# Genetic association analysis of the cardiovascular biomarker: N-terminal fragment of pro-B-type natriuretic peptide (NT-proBNP)

**DOI:** 10.1371/journal.pone.0248726

**Published:** 2021-03-15

**Authors:** Yuan Yang, Joseph M. Zmuda, Mary K. Wojczynski, Bharat Thyagarajan, Kaare Christensen, Ryan K. Cvejkus, Allison L. Kuipers

**Affiliations:** 1 Department of Epidemiology, University of Pittsburgh, Pittsburgh, PA, United States of America; 2 Department of Human Genetics, University of Pittsburgh, Pittsburgh, PA, United States of America; 3 Department of Genetics, Washington University in St Louis, St. Louis, MO, United States of America; 4 Department of Laboratory Medicine and Pathology, University of Minnesota, Minneapolis, MN, United States of America; 5 Department of Epidemiology, Biostatistics and Biodemography, Danish Aging Research Center, University of Southern Denmark, Odense C, Denmark; University of Texas School of Public Health, UNITED STATES

## Abstract

**Background:**

NT-proBNP is a biomarker of cardiovascular disease (CVD). Little is known about the heritability and genetic variants associated with NT-proBNP. Therefore, we estimated the heritability of and examined genetic associations of SNPs in the BNP gene region with circulating NT-proBNP and prevalent CVD in 4,331 participants from the Long Life Family Study (LLFS).

**Methods and results:**

Genotypes of 10 SNPs from the *NPPB* and *NPPA* regions that encode BNP and A-type natriuretic peptide, respectively, were tested for association with NT-proBNP and prevalent cardiovascular disease and risk factors. We performed analyses using the Sequential Oligogenic Linkage Analysis (SOLAR) program to account for family relatedness, and adjusted all models for age, sex, and field center. The mean age of the LLFS was 69.8 years (range 24–110) with 55.4% females. NT-proBNP was significantly heritable (h^2^ = 0.21; P = 4x10^-14^), and the minor alleles of rs632793 (p<0.001) and rs41300100 (p = 0.05) were independently associated with higher serum NT-proBNP levels. Additionally, the minor allele of rs632793 was significantly and consistently associated with lower prevalent CVD, including blood pressures, independent of NT-proBNP level (all P<0.05). Results for prevalent CVD, but not NT-proBNP levels, showed significant interaction by familial generation.

**Conclusion:**

In this family-based study of subjects with exceptional longevity, we identified several allelic variants in the BNP gene region associated with NT-pro-BNP levels and prevalent CVD.

## Introduction

Cardiovascular disease (CVD) is the leading cause of death globally [1]. B-type natriuretic peptide (BNP) has been established as a diagnostic biomarker for heart failure (HF), a subset of CVD [2]. BNP has also been associated with established risk factors for CVD including hypertension [3], obesity [4] and diabetes [5]. The natriuretic peptide family consists of three peptides: atrial natriuretic peptide (ANP), brain natriuretic peptide (BNP), and C-type natriuretic peptide. BNP is released as a pro-hormone (proBNP), and proBNP comprises 108 amino acids, which can be cleaved into two parts: N-terminal fragment proBNP (AA residues 1–76; NT-proBNP), and C-terminal BNP (AA residues77-108) [6]. NT-proBNP persists in the circulation longer, has a higher concentration in plasma, and is more stable in vitro compared to the full length or C-terminal BNP, which makes NT-proBNP a better clinical biomarker [7].

Cardiomyocyte stretch during abnormal intracardiac pressure is the primary stimulus for BNP expression [8]. As such, circulating BNP serves as a compensatory mechanism to offset the excessive vascular vasoconstriction when there is a decreased pumping capacity of the heart. NT-proBNP levels are used clinically to predict the risk of CVD, diagnose heart failure (HF), and to monitor the prognosis of other CVDs [3, 9, 10]. A diagnosis of HF can be ruled out if with age-specific low NT-proBNP levels in the non-acute setting, as established by the 2016 European Society of Cardiology guidelines [9]. Specifically, the normal range of NT-proBNP is <125pg/ml in individuals aged <75 years, and <450pg/ml in individuals aged ≥75years [11].

However, little is known about the genetic determinants of BNP with only a handful of studies evaluating its genetic regulation [12] and only a single study reporting its genetic heritability [13]. The study of heritability included 1,914 Framingham Heart Study participants from which they estimated the heritability of NT-proBNP to be 0.35 after adjustment for age, sex, and clinical variables [13]. Several single nucleotide polymorphisms (SNPs) in the *NPPB* gene region have been associated with NT-proBNP levels and CVD outcomes in population studies [14, 15] and clinical studies of patients with coronary artery disease [16].

In the current study, we aimed to replicate and extend results from a previous study conducted in the Atherosclerotic Risk in Communities (ARIC) cohort [15], of the association of prevalent CVD conditions with common genetic variation in the BNP gene region. Like previous studies, we focused on common, independent genetic variation in the *NPPA*/*NPPB* region, two genes that lie in close proximity to each other and encode for ANP and BNP, respectively. In addition, we expanded on previous findings from ARIC by conducting analyses in generally healthy adults from a unique set of families recruited for exceptional longevity, the Long Life Family Study (LLFS). Compared to ARIC, LLFS is older (69.8 ± 15.5 vs. 54.3 ± 6) with a wider age range (24–110 vs. 45–64), and has a lower prevalence of hypertension. Therefore, not only is the LLFS a sample of individuals at low risk for their age of severe CVDs like HF, but the wide age range also allows for determination of how results may differ by age–a known strong correlate of NT-proBNP levels [17].

## Methods

### Population

The Long Life Family Study (LLFS) is a family-based multi-center cohort study that enrolled 4,953 predominantly European-ancestry participants between 2006 and 2009 from 539 families with exceptional longevity [18]. Participants were recruited at four study centers: Boston, MA (Boston University), New York, NY (Columbia University), Pittsburgh, PA (University of Pittsburgh), and Denmark (University of Southern Denmark). Potential probands in the US who were at least 79 years old on January 1, 2005, and who had no end-stage renal disease or residence in hospice programs, were pre-screened for eligibility by telephone [18]. In Denmark, individuals who would be aged 90 and above during the study recruitment period were identified through the Danish National Register of Persons, which contains current information on names, addresses, place of birth, marriages, and vital status, and linked to archived parish registers to identify sibships. Eligibility at all centers was assessed by calculating a Family Longevity Selection Score (FLoSS), which was built from the birth date, gender, nation-specific cohort survival probabilities and the number of older living siblings [19]. A FloSS score of 7 or higher was required for a family to be eligible as long as they also met the following criteria: the proband and at least one living sibling and one of their living offspring were willing to participate in the LLFS interview and provide a blood sample [18].

All baseline LLFS interviews and examinations were conducted in person with portable equipment. All research assistants conducting the exams were centrally trained and certified, and used standard protocols. Copies of all LLFS forms are provided with this manuscript as Supporting Information. The current analysis was done on a subset of 4,331 LLFS participants after excluding 622 participants who were missing serum NT-proBNP measures or measures under the assay detection limit of 5pg/ml (N = 335) or was an outlier (BNP = 27950, N = 1), had current and/or a history of heart failure (N = 218), and those with missing pedigree data (N = 68). Written informed consent was obtained from each LLFS participant, and all study forms and protocols were approved by the Institutional Review Boards at Boston University, Columbia University, and the University of Pittsburgh, and by the Research Ethics Committee at the University of Southern Denmark.

### Measurement of NT-proBNP

Blood samples were collected after a ≥6-hour fast and were shipped on ice within 48 hours to the LLFS central blood laboratory at the University of Minnesota for storage at -80°C until assays were completed. Serum NT-proBNP was measured at baseline in the central laboratory by a Roche e411 Analyzer, which applies the sandwich immunoassay method to quantify NT-proBNP (Roche Diagnostics, Indianapolis, IN). The NT-proBNP inter-assay coefficient of variation (CV) was 2.7% at a mean concentration of 139.9 pg/mL and 5.1% at a mean concentration of 4,707.3 pg/mL.

### Genotyping

**Fig 1:**
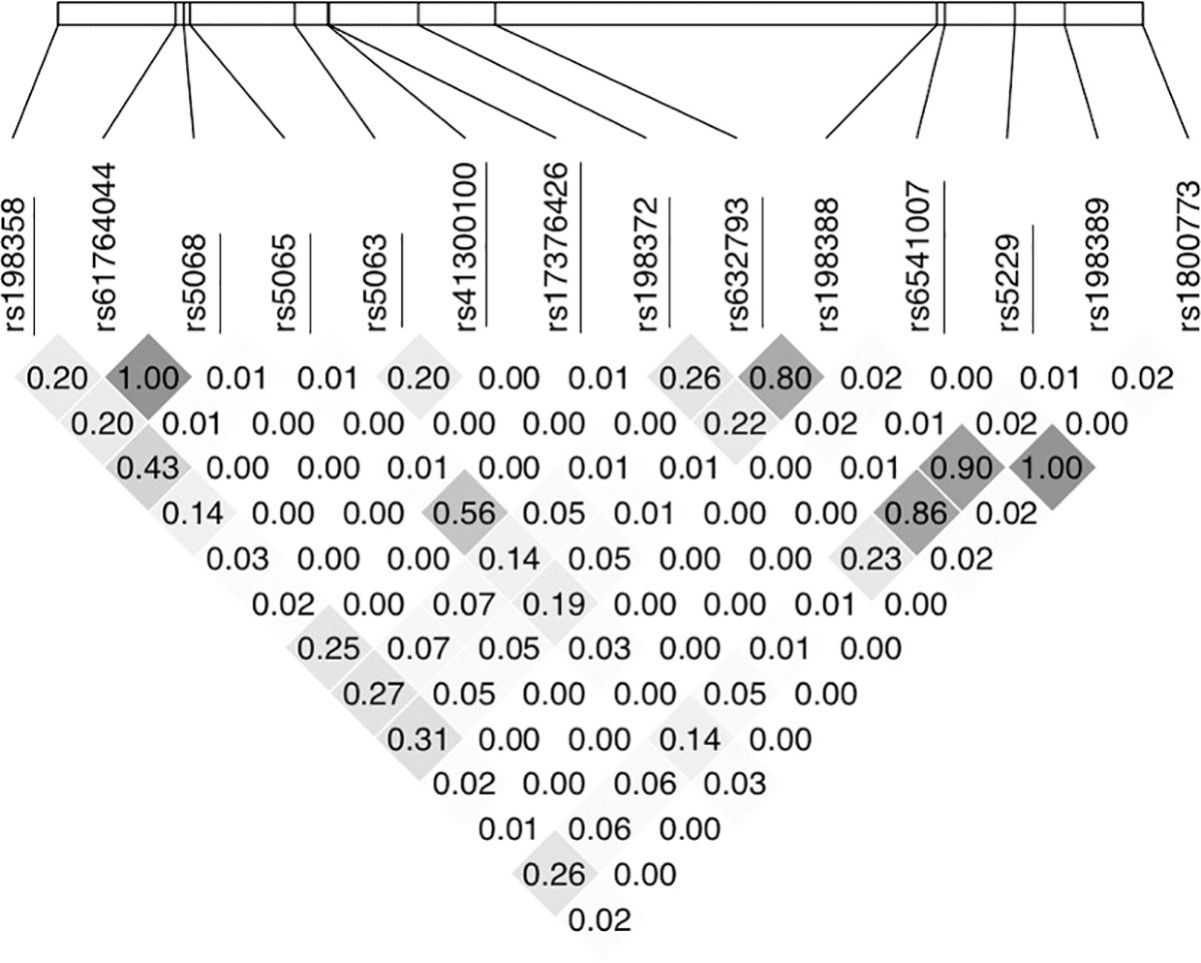
Linkage disequilibrium in the *NPPB/NPPA* gene region in the LLFS. Pairwise linkage disequilibrium (LD) in the LLFS was calculated and plotted using the gaston package in R for all genotyped SNPs in the BNP gene regions. R^2^ values are shown and depicted using a heatmap function. Independent SNPs selected for this analysis are underlined.

Genomic DNA was extracted from peripheral blood samples at the LLFS central blood laboratory at the University of Minnesota and shipped to the LLFS central genomics laboratory at the University of Washington St. Louis for genotyping. All genotype data from the LLFS were obtained from the Human Omni 2.5 v1 (Illumina, CA) genotyping chip, which was used on genomic DNA extracted from peripheral blood samples. In order to replicate previous studies and have enough power to detect associations with multiple comparisons, we focused on SNPs located within the genomic region spanning from the 5’ end of *NPPA* through the 3’ end of *NPPB*, which encode for ANP and BNP, respectively, and lie next to each other on chromosome 1. Both *NPPB* and *NPPA* regions were considered since there is evidence that these two genes share common regulatory sequences [20]. In total, 20 SNPs were directly genotyped in this region on the OMNI Chip. We excluded SNPs whose total number of heterozygotes + minor homozygotes was <10 (N = 5) and selected 1 SNP at random for each set of SNPs with linkage disequilibrium (LD)>0.8 (N = 5 excluded; Fig 1). After these exclusions, 10 SNPSs were selected for analysis. We coded the remaining SNPs as 0, 1, 2 based on the number of minor alleles. If the number of minor homozygous genotypes was <10, then we combined the minor homozygote genotype with the heterozygote genotype in order to increase power (N = 4; Table 1).

**Table 1:** Description of genotyped SNPs in the *NPPA/NPPB* gene region.

SNP	Chromosome 1 location (bp)*	Major/minor allele	Major homozygote n (%)	Heterozygote n (%)	Minor homozygote n (%)	Minor allele frequency	Model
**rs198358**	11844019	A/G	2545 (59.4)	1500 (35.0)	243 (5.7)	0.23	Additive
**rs5068**	11845917	T/C	3843 (89.4)	440 (10.2)	15 (0.4)	0.05	Additive
**rs5065**	11846011	A/G	3187 (74.1)	996 (23.2)	117 (2.7)	0.14	Additive
**rs5063**	11847591	G/A	3906 (90.8)	382 (8.9)	12 (0.3)	0.05	Additive
**rs41300100**	11848089	C/G	4218 (98.3)	74 (1.7)	1 (0.02)	0.01	Dominant
**rs17376426**	11848110	C/T	3999 (93.0)	296 (6.9)	3 (0.1)	0.04	Dominant
**rs198372**	11849457	G/A	3166 (73.6)	1029 (24.0)	105 (2.4)	0.14	Additive
**rs632793**	11850620	A/G	1488 (34.6)	2082 (48.4)	728 (16.9)	0.41	Additive
**rs6541007**	11857407	G/A	4119 (95.8)	180 (4.2)	2 (0.05)	0.02	Dominant
**rs5229**	11858462	G/A	4179 (98.1)	82 (1.9)	0 (0.0)	0.01	Dominant

*sorted in 5’ to 3’ direction based on human genome GRCh38.p13.

### Other measurements

Standing height was measured to the nearest 0.1 cm with a Handi-stat set square (Perspective Enterprises, Portage, MI) and the average of four measurements was used in analysis. Weight was measured to the nearest 0.1 kg with an electronic digital scale (SECA 841, Hanover, MD). Body mass index (BMI) was calculated from these measurements as weight (kg)/height (m)^2^. Centrally trained research assistants measured sitting blood pressure using the average of three readings from an automated blood pressure machine (BP-tru BPM 300, VMS Medtech, Coquitlam, Canada). Hypertension was defined as SBP ≥ 130 mmHg or DBP ≥ 80 mmHg during the LLFS examination or current use of anti-hypertension medication [18]. Participant history of (atrial fibrillation) AF and myocardial infarction (MI) were determined by self-report during the interview process and included collection of information regarding treatment or hospitalization, if applicable.

### Statistical analysis

All analyses were performed using the Sequential Oligogenic Linkage Analysis (SOLAR) program, which applies a variance-covariance method to adjust for family structure [21]. We defined outliers for NT-proBNP as +/- 4SD from the mean and excluded one observation (value = 27,950 pg/ml). We then log-transformed the NT-proBNP values to conform to the assumption of normal distribution as required by the SOLAR program. All analyses were adjusted for age, sex, and field center and used an alpha of 0.05 as a threshold for nominal significance, but also assessed Bonferroni-corrected alpha levels, as described below. Other potential risk factors for CVD, such as BMI, smoking, lipoprotein levels, and diabetes were considered to be potentially in the causal pathway and were not included as adjustment factors in order to not over-adjust the associations. We used multiple linear regressions to test the association of our selected SNPs (predictors) and logNT-proBNP (outcome), first as individual SNPs and then as SNPs combined into a single model in order to identify SNPs with independent associations with NT-proBNP (Bonferroni corrected alpha for 10 SNPs = 0.005).

We tested the association of SNPs that were associated with NT-proBNP level with individual CVD related measures and risk factors (Bonferroni corrected alpha for 7 SNPs = 0.007) including blood pressures, prevalent hypertension, BMI, history of AF, and history of MI. Lastly, for all significant models after Bonferroni correction, we ran additional analyses including adjustment for NT-proBNP level in order to determine if the significant genetic association was partially driven by NT-proBNP level itself. We also conducted sensitivity analyses among individuals of either generation who had NT-proBNP levels in the normal range defined as: <125pg/ml in those aged <75 years, and <450pg/ml in individuals aged ≥75years (N = 3,309) [22]. Lastly, given the wide age range and global distribution of the LLFS, we also performed stratified analyses and tested for interaction by familial generation (proband generation and offspring generation) and country (US versus Denmark).

## Results

### Characteristics of LLFS population

The 4,331 participants from the LLFS in this analysis consisted of 55.4% females and were aged 69.8 years (24–110) on average (Table 2). Of the 4,331 participants, 1,385 were in the proband generation (49–110), while 2,946 were in the offspring generation (24–88). Approximately 6% of participants had a history of AF and 5% had a history of MI. The average systolic blood pressure (SBP) and diastolic blood pressure (DBP) were 131.8 mmHg and 77.5 mmHg, respectively. 55% percent of participants had hypertension and 80% were on anti-hypertensive therapy. The median level of NT-proBNP was 86 pg/ml. After adjusting for age, sex, and field center, the residual heritability of NT-proBNP was 0.21 (p = 4 × 10^−14^).

**Table 2:** Basic characteristics of the LLFS by generation.

Characteristic		All LLFS	Proband Generation	Offspring Generation
Total Participants		4331	1385	2946
Age (mean ± SD)		69.8 ± 15.5	89.2 ± 6.9	60.6 ± 8.3
Female n (%)		2398 (55.4)	755 (54.51)	1643 (55.8)
Field Center n (%)	Denmark	1163 (26.9)	203 (14.7)	960 (32.6)
	Boston	1165 (26.9)	367 (25.5)	798 (27.1)
	New York	863 (19.9)	408 (29.5)	455 (15.4)
	Pittsburgh	1140 (26.3)	407 (29.4)	733 (24.9)
BMI (kg/m^2^) mean ± SD		27.1 ± 4.8	26.1 ± 4.2	27.5 ± 4.9
SBP (mmHg) mean ± SD		131.8 ± 22.2	139.2 ± 25.3	128.4 ± 19.7
DBP (mmHg) mean ± SD		77.5 ± 11.4	74.0 ± 11.7	79.1 ± 10.9
Hypertension n (%)		3166 (73.1)	1189 (85.9)	1977 (67.1)
Diabetes n (%)		300 (6.9)	131 (9.5)	169 (5.7)
Atrial Fibrillation n (%)		242 (5.6)	175 (12.7)	67 (2.3)
Myocardial Infarction n (%)		213 (4.9)	129 (9.3)	84 (2.9)
NT-proBNP (pg/ml) median (range)		86.0 (5–14739)	341 (5–14739)	54 (5–5310)

BMI: Body Mass Index, NT-proBNP: N-Terminal pro-BNP, SBP: Systolic Blood Pressure, DBP: Diastolic Blood Pressure.

### Associations of SNPs with NT-proBNP level

Genotype information for the 10 SNPs in the *NPPA/NPPB* region used in this analysis are listed in Table 1. Seven of these SNPs showed nominally significant associations with NT-proBNP level (all P<0.05; Table 3). Entered together into a single model, these 7 SNPs accounted for 0.85% of the variance in NT-proBNP level. However, only 2 SNPs were independently associated with NT-proBNP level: rs632792 (additive β = 0.16, p<0.001) and rs41300100 (dominant β = 0.26, p = 0.05; Table 3). On average, for each copy of the G allele at rs632793, individuals had 3.5% greater NT-proBNP than individuals with the AA genotype. Additionally, individuals with 1 or more A allele at rs41300100 had 5.7% greater NT-proBNP than those with the GG genotype.

**Table 3:** Association between SNPs and NT-proBNP level.

SNPs	Univariate		Independently Significant SNP	
	β	P-value	β	P-value
**rs198358**	0.11	<0.001*	-0.03	0.43
**rs5068**	0.12	**0.01**	0.06	0.33
**rs5065**	0.03	0.31		
**rs5063**	0.24	<0.001*	0.13	0.06
**rs41300100**	0.47	<0.001*	0.26	**0.05**
**rs17376426**	0.12	**0.04**	-0.09	0.14
**rs198372**	0.06	**0.04**	-0.02	0.6
**rs632793**	0.17	<0.001*	0.16	<0.001*
**rs6541007**	-0.05	0.55		
**rs5229**	-0.03	0.82		

NT-proBNP was modeled as the outcome and the SNPs were the predictors, adjusting for age, sex, study center. For Univariate results, each SNP was tested alone in a single model. Then SNPs that were significant in Univariate analyses were combined together into a single model to determine SNPs with independent effects on NT-proBNP level.

**BOLD** signifies P<0.05

*denotes passing Bonferroni correction threshold.

### Association of NT-proBNP with prevalent CVD measures

Associations between NT-proBNP level and CVD related measures are shown in S1 Table. NT-proBNP level was positively associated with age (p = 1 × 10^−307^), such that a 1 unit greater logNT-proBNP (~2.72 pg/ml) was associated with ~8 years greater age after adjusting for sex and study center. After adjusting for age and study center, 1 log(pg/ml) increased logNT-proBNP was associated with 20% greater odds of being male (P = 1×10^−17^). After full adjustment for age, sex, and study center, 1 unit increased NT-proBNP was associated with 0.53 mmHg decreased diastolic blood pressure (p = 0.002), 0.12 kg/m^2^ decreased BMI, 45% decreased odds of having AF history, and 22% decreased odds of MI history (all P<0.001).

### Association of SNPs with prevalent CVD measures

Associations between the 7 SNPs individually associated with NT-proBNP levels and CVD measures are shown in Table 4. The minor allele of rs632793 was nominally associated with AF, such that the presence of each G allele was associated with an additional 10% lower odds of a history of AF compared to individuals with the AA genotype (P = 0.030). Also, the minor (G) allele of rs632793 was associated with lower SBP (β = -1.3, p = 0.0095) and DBP (β = -0.6, p = 0.018), but no significant association with prevalent hypertension when defined by blood pressures or medication status (p = 0.807).

**Table 4:** Association between CVD measures and SNPs associated with BNP level.

SNP	BMI		SBP		DBP		Hypertension		AF		MI	
	β	P	β	P	β	P	β	P	β	P	β	P
rs198358	-0.04	0.15	0.14	0.8	-0.04	0.9	0.03	0.39	-0.09	0.09	-0.02	0.7
rs5068	0.02	0.64	-1.05	0.32	0.21	0.7	0.04	0.53	-0.14	0.18	0.02	1
rs5063	-0.06	0.27	0.36	0.75	-0.9	0.13	-0.01	0.9	0.05	0.69	-0.1	0.37
rs41300100	0.07	0.57	-1.08	0.67	-0.78	0.56	-0.07	0.7	-0.07	0.79	0.36	0.23
rs17376426	0.04	0.54	-0.22	0.87	0.93	0.17	-0.01	0.88	-0.01	0.93	0.02	0.98
rs198372	-0.03	0.44	-0.64	0.34	-0.4	0.25	0.03	0.55	-0.01	0.89	-0.03	0.69
rs632793	-0.02	0.36	-1.25	**0.01**	-0.59	**0.018**	0.01	0.81	-0.11	**0.03**	0	0.89

Adjusted for age, sex, and study center.

**BOLD** signifies P<0.05.

After further adjusting significant associations for NT-proBNP level, the association between the minor allele of rs632793 and AF was attenuated (β = -0.07; p = 0.180), while its associationwith lower SBP and DBP remained significant (P<0.04; S5 Table). In sensitivity analyses conducted only in individuals with NT-proBNP levels in the normal range (N = 3309), the results were largely similar for all analyses (S2–S4 Tables).

### Stratified analyses

Compared to the proband generation, the offspring generation had a greater number of significant associations between SNPs and NT-proBNP level (Table 5). In addition to rs632793, rs17376426 was significantly and independently associated with NT-proBNP levels in the offspring generation. While there was significant interaction by generation for rs198358 and rs5065 (both P_interaction_ <0.05), the effect of rs632793 on levels of NT-proBNP was similar across generations (P_interaction_ = 0.09). However, the associations of SNPs with CVD measures (Table 6) were predominantly seen in the proband generation, without any significant findings in the offspring who had less CVD burden in general (Table 2). Similar to results in all LLFS, rs632793 was inversely associated with SBP and DBP, although the association with AF was not significant in either single generation subset (Table 6). These generation-specific effects for rs632793 are suggestive of a significant interaction by generation on blood pressures (P_interaction_<0.05; S6 Table). Additionally, rs41300100 was significantly associated with hypertension (β = -4.06, p = 0.006, Table 6) in the proband generation, alone; however this effect did not reflect significant interaction by generation (S6 Table).

**Table 5:** Association of BNP variants with NT-proBNP level stratified by LLFS generation.

SNPs	Proband Generation		Offspring Generation		Interaction
	β	P-value	β	P-value	P-value
**rs198358**	0.006	0.9	0.16	**<0.001**	**0.01**
**rs5068**	0.03	0.71	0.16	**0.003**	0.25
**rs5065**	-0.08	0.15	0.08	**0.03**	**0.03**
**rs5063**	0.28	**0.004**	0.25	**<0.001**	0.84
**rs41300100**	0.45	**0.05**	0.47	**<0.001**	0.85
**rs17376426**	0.18	0.17	-0.21	0.002*	0.08
**rs198372**	-0.03	0.64	0.09	**0.007**	0.13
**rs632793**	0.1	0.01*	0.19	<0.001*	0.09
**rs6541007**	-0.06	0.7	0.04	0.62	0.41
**rs5229**	-0.08	0.66	-0.04	0.78	0.90

All models adjusted for age, sex, and study center.

* Indicates SNPs with independent effects after combining SNPs with individual P-value<0.1 together into a single model.

**Table 6:** Association of BNP variants with CVD measures stratified by LLFS generation.

NT-proBNP Associated SNP	BMI		SBP		DBP		Hypertension		AF		MI	
	β	P	β	P	β	P	β	P	β	P	β	P
**Proband **												
**rs5063**	-0.08	0.38	2.02	0.4	-1.3	0.25	-0.32	0.06	-0.09	0.57	-0.16	0.32
**rs41300100**	-0.13	0.56	0.64	0.91	-0.46	0.86	-4.06	0.006*	-0.23	0.49	0.42	0.34
**rs632793**	0.02	0.55	-2.72	0.007*	-1.42	0.003*	-0.06	0.34	-0.08	0.22	0.01	0.92
**Offspring**												
**rs198358**	-0.05	0.1	0.78	0.19	0.31	0.36	0.04	0.35	-0.14	0.11	-0.06	0.48
**rs5068**	0.06	0.31	-0.54	0.63	0.5	0.43	0.03	0.75	-0.19	0.22	-0.09	0.53
**rs5065**	-0.06	0.12	1.01	0.15	0.31	0.43	0.01	0.87	-0.17	0.08	-0.01	0.89
**rs5063**	-0.08	0.2	0.1	0.93	-0.52	0.43	0.06	0.49	0.3	0.14	-0.04	0.93
**rs41300100**	0.14	0.34	-0.62	0.82	-0.57	0.71	0.05	0.78	0.22	0.6	0.33	0.46
**rs17376426**	0.05	0.51	-0.05	0.97	0.72	0.33	-0.01	0.93	0.002	0.99	-0.04	0.84
**rs198372**	-0.04	0.33	0.06	0.93	-0	0.99	0.03	0.6	-0.11	0.29	0.04	0.72
**rs632793**	-0.04	0.16	-0.72	0.15	-0.24	0.39	0.03	0.35	-0.14	0.07	0.01	0.85

All models adjusted for age, sex, and study center.

* Indicates an association that remained significant (P<0.05) after further adjustment for NT-proBNP level.

Analyses stratified by study center (US versus Denmark) demonstrated similar population characteristics by country (S7 Table). Results were also consistent by country for the association of rs632793 and rs41300100 with NT-proBNP level (S8 Table) and showed no significant interaction effect by country on the association of BNP SNPs and CVD measures. However, the association between rs632793 and AF appears to be driven by families at the Danish study center (S8 Table).

## Discussion

We found that, in the LLFS, NT-proBNP was significantly heritable (residual heritability estimate (H^2^_r_) = 0.21) and that there were 7 SNPs in the *NPPA*/*NPPB* region associated with NT-proBNP level. The minor alleles of rs632793 and rs41300100 were independently associated with higher NT-proBNP level. Additionally, NT-proBNP was positively associated with age and male sex, but negatively associated with BMI, blood pressure, AF, and MI. The minor allele of rs632793 was inversely associated with SBP, DBP, and a history of AF. The significant association of SNPs with blood pressures was independent of NT-proBNP level, which may suggest that not all of the genetic association was explained by NT-proBNP level, though longitudinal analyses would be needed to determine this for certain. Lastly, results with prevalent CVDs also showed an interaction with age group (i.e., generation); thereby, highlighting the age-related differences in BNP function.

These results are consistent with previous findings that NT-proBNP is heritable (previous evidence H^2^_r_ = 0.34) [13] and that common SNPs in the *NPPA*/*NPPB* region are associated with circulating NT-proBNP level [15, 16, 23, 24]. Our results are also similar to previous studies showing that NT-proBNP is positively associated with age [25] and inversely associated with BMI [26]. We identified rs632793 as the most significant and consistent genetic variant associated with NT-proBNP level in the total LLFS, as well as, in the proband generation alone. This is in line with previous studies [14, 16, 27, 28], although, some other studies focused specifically and solely on rs198389 [15, 16, 24, 29–31]. These two variants are in strong LD in our study (Fig 1) and; thus, likely represent the same underlying genetic signal.

The somewhat counterintuitive association between SNPs in this region being associated with higher circulating NT-proBNP levels, but lower risk of cardiovascular conditions and risk factors even though higher NT-proBNP is associated with increased CVD risk, is consistent with previous reports [14, 16, 32–34]. The physiologic function of BNP is to relieve the heart of excess stress by reducing vascoconstriction [8, 35], so it plays a protective role in early life. But in the presence or progression of cardiac disease, the body is required to up-regulate the expression of BNP in order to attempt to relieve the symptoms of disease [15] and which is in line with associations we saw between NT-proBNP level and CVD risk factors in the current study (S1 Table). This is why greater circulating BNP is indicative of disease and can be used as a clinical marker of HF diagnosis or progression. Interestingly, our interaction analyses showed that the genetic associations did not significantly differ by generation (i.e. aging) for NT-proBNP level, but did for blood pressure and hypertension. This also supports the hypothesis that the counterintuitive direction of effect is not driven by genetic effects, but rather by the body’s regulation of NT-proBNP levels in relation to progression of cardiovascular disease.

The goal of the current study was to replicate and extend findings on BNP genetics and CVD that exist in the literature. However, it is unique to previous reports in that our families largely consist of exceptionally healthy, older adults with a low burden of CVD for their age compared to previous studies in high-risk groups. In the LLFS, we found that rs632793 which was associated with greater NT-proBNP level, was also associated with lower prevalence of cardiovascular conditions and risk factors. This relationship demonstrates the physiologic protective role of BNP across age groups and even in generally healthy adults, which suggests the relationships are not driven by a predisposition to a high risk of CVD. This idea is even more clear from the sensitivity analyses in individuals with normal NT-proBNP levels, which showed similar results to the whole LLFS cohort. This observation warrants further study of the clinical utility for CVD risk prediction of NT-proBNP in low-risk populations.

The results of the current study identified SNPs that have previously been reported to be associated with NT-proBNP and/or CVD: primarily rs632793 (in LD with rs198389), but also rs41300100 and rs17376426. While the function of rs632793 is not known, it is located in the 5’ flanking promoter region and is predicted to be associated with transcription regulation [36]. Also, rs198389 (in LD with rs632793) is located in an upstream regulatory region of *NPPB* and the minor (G) allele has been shown to be associated with 1.8-fold higher *NPPB* promoter activity [32]. rs198389 has been associated with higher NT-proBNP and BNP levels [12, 15, 16, 23, 24, 32–34, 37], lower blood pressure and hypertension [15] and a reduced rate of cardiovascular readmission [16].

In addition to rs632793, rs41300100 was associated with NT-proBNP level in all LLFS and hypertension in the proband generation, and rs17376426 was associated with NT-proBNP level independent of rs632793 in the offspring generation. rs41300100 is located in the 5 prime UTR of *NPPA*, and is predicted to lie in an enhancer region [38]. However, this particular SNP is not in LD with any other SNP in the region in the LLFS (Fig 1) and has not been reported to be associated with any traits to date. rs17376426 is located at 2KB upstream transcript variant and is predicted as a regulatory variant [39]. Therefore, the potential functional impact of rs41300100 and rs17376426 on NT-proBNP levels and/or function is unknown, and may warrant further investigation.

This study has several strengths. First, the LLFS provided a wide age range of adults to test the genetic association of BNP and is the first study to conduct these analyses in exceptionally long-lived individuals at low-risk for CVD. While the exceptional longevity and healthier than age-matched indivudals of the LLFS may make it seem like it is not generalizable, there is still substantial chronic disease prevalence and a wide variation in all measures of health within the cohort [18]. Additionally, the family study design of LLFS allowed us to calculate the heritability of NT-proBNP and is only the second study to do so. There was also a reasonably large sample size (N = 4,331) for a candidate gene approach and we used the most reliable assay for NT-proBNP measurement. We also assessed our primary results using both a nominal (P≤0.05) and Bonferroni-corrected P-value threshold given our strong *a priori* hypotheses and limited scope of work. Lastly, we looked at multiple SNPs across the *NPPA*/*NPPB* region instead of only a single candidate SNP, as many previous reports of rs198389 have done [15, 23, 24, 33, 40, 41]. This approach allowed for the identification of a number of SNPs in addition to rs198389 with significant associations to either NT-proBNP or CVD, that would have otherwise been missed.

However, there are a number of additional considerations. We do not yet have longitudinal data on NT-proBNP or incident cardiovascular disease risk in the LLFS; however, as the study continues follow-up, we will be able to assess these in the future. As age is a strong, non-linear determinant of NT-proBNP level, the inclusion of a wide-range of ages may have attenuated our associations between genetic variants and NT-proBNP given the variation in risk prediction by CVD status. However, our sensitivity analyses in individuals with normal NT-proBNP levels attempted to minimize the influence of prevalent cardiac disease. Besides, we limited our study to SNPs in the *NPPA*/*NPPB* region on chromosome 1. We did this in order to look at the association of genetic variation around BNP with circulating BNP levels and only looked at common SNPs in order to minimize the number of tests. However, SNPs in other regions, such as rs11105306 located on chromosome 12 and rs13107352 located on chromosome 4, have also been shown to be associated with the circulating NT-proBNP levels [30], which we did not assess in the current study. In addition, there are a number of factors that were not included in our analyses as they were considered to potentially be a part of the causal pathway between BNP and CVD, including BMI, smoking, lipids, and diabetes. However, it is likely that these factors are involved in the reported relationships. Lastly, while we did not include a replication cohort, we started with an *a priori* hypothesis to do a candidate gene association study and our results were in-line with previous reports; thus, our study serves as replication to this previous work.

We found that NT-proBNP level is significantly heritable in a cohort of families with exceptional aging: the LLFS. We also identified rs632793, rs41300100, and rs17376426 to be independently associated with NT-proBNP levels and related prevalent CVD conditions and risk factors. We showed that alleles associated with greater NT-proBNP were also associated with a lower prevalence of CVD conditions and risk factors, even though high NT-proBNP levels are used clinically to indicate heart failure. This seemingly counterintuitive result is consistent with previous reports and highlights the complex relationship and negative feedback loop of the BNP protein and cardiovascular conditions. As such, future research may benefit from taking into consideration genetic determinants when evaluating clinical use of BNP in the aging population.

## Supporting information

S1 Table Associations between NT-proBNP level and CVD measures. (DOCX)

S2 Table Association between SNPs and NT-proBNP level in individuals with normal NP-proBNP level. (DOCX)

S3 Table Association between significant SNPs and CVD measures in individuals with normal NP-proBNP level. (DOCX)

S4 Table Association between significant SNPs and CVD measures in individuals with normal NP-proBNP level further adjusted for NT-proBNP level. (DOCX)

S5 Table Association between significant SNPs and CVD measures further adjusted for NT-proBNP level. (DOCX)

S6 Table Effect of the interaction between significant SNP and LLFS generation on CVD measures. (DOCX)

S7 Table Basic characteristics of the LLFS by study center country. (DOCX)

S8 Table Association of significant BNP variants with CVD measures stratified by study center country. (DOCX)

S1 File Supporting information file containing all study questionnaires. (PDF)
