# Supplementary material for: Genetic association analysis of the cardiovascular biomarker: N-terminal fragment of pro-B-type natriuretic peptide (NT-proBNP)
Source: PLoS One. 2021 Mar 15;16(3):e0248726. doi: 10.1371/journal.pone.0248726 (PMC7959346; doi:10.1371/journal.pone.0248726)
Supplement: S1 Table — (DOCX) [file pone.0248726.s001.docx]

S1 Table. Associations Between NT-proBNP Level and CVD Measures

| **Outcome** | **𝛽** | **P-value** |
| --- | --- | --- |
| **Age*** | 7.86 | **<0.001** |
| **Female†** | -0.2 | **<0.001** |
| **BMI** | -0.12 | **<0.001** |
| **SBP** | -0.13 | 0.691 |
| **DBP** | -0.53 | **0.002** |
| **Prevalent Hypertension** | -0.003 | 0.195 |
| **History of Atrial Fibrillation** | -0.45 | **<0.001** |
| **History of Myocardial Infarction** | -0.22 | **<0.001** |

logNT-proBNP was modeled as the predictor per 1 log(pg/ml) unit adjusted for age, sex, and study center.

*Results for age were adjusted for sex and study center

†Results for sex were adjusted for age and study center
