# Supplementary material for: Genetic association analysis of the cardiovascular biomarker: N-terminal fragment of pro-B-type natriuretic peptide (NT-proBNP)
Source: PLoS One. 2021 Mar 15;16(3):e0248726. doi: 10.1371/journal.pone.0248726 (PMC7959346; doi:10.1371/journal.pone.0248726)
Supplement: S2 Table — (DOCX) [file pone.0248726.s002.docx]

**S2 Table. Association Between SNPs and NT-proBNP Level in Individuals with Normal NP-proBNP Level**

| **SNPs** | **Univariate** | | **Independently Significant SNP** | |
| --- | --- | --- | --- | --- |
|  | **𝛽** | **P-value** | **𝛽** | **P-value** |
| rs198358 | 0.10 | **<0.001*** | 0.002 | 0.95 |
| rs5068 | 0.08 | 0.07 |  |  |
| rs5065 | 0.06 | **0.03** | 0.01 | 0.90 |
| rs5063 | 0.19 | **<0.001*** | 0.11 | 0.07 |
| rs41300100 | 0.33 | **0.004*** | 0.12 | 0.31 |
| rs17376426 | -0.07 | 0.18 |  |  |
| rs198372 | 0.08 | **0.001*** | -0.007 | 0.87 |
| rs632793 | 0.14 | **<0.001*** | 0.12 | **<0.001*** |
| rs6541007 | -0.13 | **0.04** | -0.07 | 0.32 |
| rs5229 | -0.10 | 0.32 |  |  |

NT-proBNP was modeled as the outcome and the SNPs were the predictors, adjusting for age, sex, study center.

**BOLD** signifies P<0.05; *denotes passing Bonferroni correction threshold
