# Supplementary material for: Genetic association analysis of the cardiovascular biomarker: N-terminal fragment of pro-B-type natriuretic peptide (NT-proBNP)
Source: PLoS One. 2021 Mar 15;16(3):e0248726. doi: 10.1371/journal.pone.0248726 (PMC7959346; doi:10.1371/journal.pone.0248726)
Supplement: S3 Table — (DOCX) [file pone.0248726.s003.docx]

**S3 Table. Association Between Significant SNPs and CVD Measures in Individuals with Normal NP-proBNP Level**

| **SNP** | **BMI** | | **SBP** | | **DBP** | | **Hypertension** | | **AF** | | **MI** | | |
| --- | --- | --- | --- | --- | --- | --- | --- | --- | --- | --- | --- | --- | --- |
|  | **𝛽** | **P** | **𝛽** | **P** | **𝛽** | **P** | **𝛽** | **P** | **𝛽** | **P** | **𝛽** | **P** |  |
| rs198358 | -0.05 | 0.10 | -0.17 | 0.78 | -0.26 | 0.41 | 0.07 | 0.07 | -0.16 | 0.08 | 0.07 | 0.38 |  |
| rs5065 | -0.04 | 0.33 | -0.06 | 0.93 | -0.25 | 0.50 | 0.04 | 0.40 | -0.15 | 0.11 | 0.10 | 0.26 |  |
| rs5063 | -0.02 | 0.81 | 0.71 | 0.56 | -0.49 | 0.45 | -0.01 | 0.92 | 0.04 | 0.81 | -0.12 | 0.42 |  |
| rs41300100 | 0.19 | 0.22 | -2.37 | 0.43 | -1.70 | 0.30 | 0.02 | 0.92 | -0.09 | 0.84 | 0.23 | 0.60 |  |
| rs198372 | -0.02 | 0.67 | -1.18 | 0.09 | -0.74 | 0.05 | 0.08 | 0.10 | -0.10 | 0.31 | 0.08 | 0.35 |  |
| rs6541007 | 0.04 | 0.64 | 1.89 | 0.28 | 0.49 | 0.61 | -0.10 | 0.45 | 0.24 | 0.40 | 0.42 | 0.11 |  |
| rs632793 | -0.02 | 0.45 | -1.33 | **0.008** | -0.56 | **0.041** | 0.05 | 0.15 | -0.14 | **0.045** | 0.08 | 0.29 |  |

Adjusted for age, sex, and study center.

**BOLD** signifies P<0.05.
