# Supplementary material for: Genetic association analysis of the cardiovascular biomarker: N-terminal fragment of pro-B-type natriuretic peptide (NT-proBNP)
Source: PLoS One. 2021 Mar 15;16(3):e0248726. doi: 10.1371/journal.pone.0248726 (PMC7959346; doi:10.1371/journal.pone.0248726)
Supplement: S4 Table — (DOCX) [file pone.0248726.s004.docx]

**S4 Table. Association Between Significant SNPs and CVD Measures in Individuals with Normal NP-proBNP Level Further Adjusted for NT-proBNP Level**

| **SNP** | **SBP** | | **DBP** | | **AF** | |
| --- | --- | --- | --- | --- | --- | --- |
|  | **𝛽** | **P** | **𝛽** | **P** | **𝛽** | **P** |
| rs198372 |  |  | -0.67 | 0.08 |  |  |
| rs632793 | -1.31 | **0.009** | -0.44 | 0.11 | -0.12 | 0.11 |

Adjusted for age, sex, study center, and NT-proBNP.

**BOLD** signifies P<0.05.
