# Supplementary material for: Genetic association analysis of the cardiovascular biomarker: N-terminal fragment of pro-B-type natriuretic peptide (NT-proBNP)
Source: PLoS One. 2021 Mar 15;16(3):e0248726. doi: 10.1371/journal.pone.0248726 (PMC7959346; doi:10.1371/journal.pone.0248726)
Supplement: S5 Table — (DOCX) [file pone.0248726.s005.docx]

**S5 Table. Association Between Significant SNPs and CVD Measures Further Adjusted for NT-proBNP Level**

| **SNP** | **SBP** | | **DBP** | | **Hypertension** | | **AF** | |
| --- | --- | --- | --- | --- | --- | --- | --- | --- |
|  | **𝛽** | **P** | **𝛽** | **P** | **𝛽** | **P** | **𝛽** | **P** |
| All LLFS |  | | | | | | | |
| rs632793 | -1.25 | **0.0097** | -0.51 | **0.042** |  |  | -0.07 | 0.18 |
| Proband |  | | | | | | | |
| rs5063 |  |  |  |  | -0.28 | 0.096 |  |  |
| rs41300100 |  |  |  |  | -4.03 | **0.0096** |  |  |
| rs632793 | -2.70 | **0.007*** | -1.43 | **0.002*** |  |  |  |  |
| Offspring |  | | | | | | | |
| rs5065 |  |  |  |  |  |  | -0.12 | 0.27 |
| rs632793 |  |  |  |  |  |  | -0.09 | 0.31 |

Adjusted for age, sex, and study center.

**BOLD** signifies P<0.05; *denotes passing Bonferroni correction threshold
