# Supplementary material for: Genetic association analysis of the cardiovascular biomarker: N-terminal fragment of pro-B-type natriuretic peptide (NT-proBNP)
Source: PLoS One. 2021 Mar 15;16(3):e0248726. doi: 10.1371/journal.pone.0248726 (PMC7959346; doi:10.1371/journal.pone.0248726)
Supplement: S6 Table — (DOCX) [file pone.0248726.s006.docx]

**S6 Table. Effect of the Interaction Between Significant SNP and LLFS Generation on CVD Measures**

| **SNP*generation interaction** | **SBP** | | **DBP** | | **Hypertension** | |
| --- | --- | --- | --- | --- | --- | --- |
|  | **𝛽** | **P** | **𝛽** | **P** | **𝛽** | **P** |
| rs41300100 | 0.35 | 0.95 | 0.26 | 0.93 | 0.41 | 0.36 |
| rs632793 | 1.95 | **0.05** | 1.22 | **0.02** | 0.08 | 0.24 |

Adjusted for age, sex, and study center.

**BOLD** signifies P<0.05
