# Supplementary material for: Genetic association analysis of the cardiovascular biomarker: N-terminal fragment of pro-B-type natriuretic peptide (NT-proBNP)
Source: PLoS One. 2021 Mar 15;16(3):e0248726. doi: 10.1371/journal.pone.0248726 (PMC7959346; doi:10.1371/journal.pone.0248726)
Supplement: S7 Table — (DOCX) [file pone.0248726.s007.docx]

**S7 Table. Basic Characteristics of the LLFS by study center**

| **Characteristic** | **Denmark** | **US** |
| --- | --- | --- |
| Total Participants | 923 | 2386 |
| Age (mean ± SD) | 64.2 ± 12.4 | 67.8 ± 15.0 |
| Female n (%) | 493 (53.4) | 1307 (54.8) |
| BMI (kg/m^2^) mean ± SD | 26.5 ± 4.2 | 27.6 ± 4.9 |
| SBP (mmHg) mean ± SD | 136.1 ± 21.2 | 128.2 ± 19.9 |
| DBP (mmHg) mean ± SD | 81.5 ± 10.9 | 76.6 ± 10.5 |
| Hypertension n (%) | 706 (76.5) | 1612 (67.6) |
| Diabetes n (%) | 53 (5.7) | 171 (7.2) |
| Atrial Fibrillation n (%) | 20 (2.2) | 58 (2.4) |
| Myocardial Infarction n (%) | 27 (2.9) | 85 (3.6) |
| NT-proBNP (pg/ml) median (range) | 57 (5-443) | 60 (5-449) |

BMI: Body Mass Index, NT-proBNP: N-Terminal pro-BNP, SBP: Systolic Blood Pressure, DBP: Diastolic Blood Pressure
