# Supplementary material for: Genetic association analysis of the cardiovascular biomarker: N-terminal fragment of pro-B-type natriuretic peptide (NT-proBNP)
Source: PLoS One. 2021 Mar 15;16(3):e0248726. doi: 10.1371/journal.pone.0248726 (PMC7959346; doi:10.1371/journal.pone.0248726)
Supplement: S8 Table — (DOCX) [file pone.0248726.s008.docx]

**S8 Table. Association of Significant BNP Variants with CVD Measures Stratified by Study Center**

| **NT-proBNP Associated SNP** | **NT-proBNP** | | **BMI** | | **SBP** | | **DBP** | | **Hypertension** | | **AF** | | **MI** | | |
| --- | --- | --- | --- | --- | --- | --- | --- | --- | --- | --- | --- | --- | --- | --- | --- |
|  | **𝛽** | **P** | **𝛽** | **P** | **𝛽** | **P** | **𝛽** | **P** | **𝛽** | **P** | **𝛽** | **P** | **𝛽** | **P** |  |
| **US** |  | | | | | | | | | | | | | | |
| rs41300100 | 0.30 | 0.07 | 0.06 | 0.79 | -2.31 | 0.58 | -1.35 | 0.56 | -0.04 | 0.89 | -0.37 | 0.47 | -0.10 | 0.85 |  |
| rs632793 | 0.12 | **<0.001** | -0.02 | 0.48 | -0.98 | 0.09 | -0.38 | 0.24 | 0.03 | 0.52 | -0.08 | 0.34 | 0.04 | 0.64 |  |
| **Denmark** |  | | | | | | | | | | | | | | |
| rs41300100 | 0.34 | **0.01** | 0.36 | 0.09 | -3.06 | 0.49 | -2.17 | 0.34 | 0.09 | 0.75 | 3.03 | 0.35 | 3.09 | 0.26 |  |
| rs632793 | 0.18 | **<0.001** | -0.01 | 0.92 | -2.12 | 0.03 | -0.9 | 0.08 | 0.11 | 0.09 | -0.32 | **0.02** | 0.2 | 0.13 |  |

Adjusted for age and sex. All tests for interaction were P>0.1.

**BOLD** signifies P<0.05
