# Supplementary material for: Genetic association analysis of the cardiovascular biomarker: N-terminal fragment of pro-B-type natriuretic peptide (NT-proBNP)
Source: PLoS One. 2021 Mar 15;16(3):e0248726. doi: 10.1371/journal.pone.0248726 (PMC7959346; doi:10.1371/journal.pone.0248726)
Supplement: S1 File — (PDF) [file pone.0248726.s009.pdf]

LONG LIFE FAMILY STUDY	**(Affix Label Here)**<br>Participant ID: ____________<br>Participant Name Code: ____________	**Date Form Initiated** (e.g., 10JUN2005):<br>☐ ☐ ☐ ☐ ☐ ☐ ☐ ☐ ☐<br>d d m m m y y y y<br>**Date Form Completed** (e.g., 10JUN2005):<br>☐ ☐ ☐ ☐ ☐ ☐ ☐ ☐ ☐<br>d d m m m y y y y<br>Interviewer Code: ☐ ☐ ☐<br>Please Circle Field Center Location:<br>**BU CU DK UP**

LLFS Family Longevity Selection Score (FLoSS) Instrument – Proband (FLoSS-1 a/k/a TS1)

Interviewer Note: ***To be kept in a confidential file separate from other data forms.***

You will be calling the homes of potential participants who have been identified from Medicare (CMS) enrollment lists. However, the potential participant may not be the person who picks up the telephone. So the first task in the interview is to identify whom you are talking to.

Interviewer: *Please answer the following questions before beginning:*

How is this interview being documented or recorded?

- ☐1 ....Entry Directly Into the Computer
- ☐2 ....Completion of Paper Form

What level of Telephone Contact is this?

- ☐1 ....Screenee-Initiated – Returned Reply Card/Phone Call **Go to Script (a)**
- ☐2 ....Screenee-Initiated – PR, Media, etc. **Go to Script (a)**
- ☐3 ....Field Center Follow-up – No Reply Card Returned **Go to Script (b)**

(a) ***Interviewer Script***: *I am calling from* [insert your institution here] *about the Long Life Family Study, a family study of longevity. We hope to discover how some people and their families live long lives. I am calling because you have expressed interest in this study. May I ask who I am speaking with? Are you* [insert Screenee's name]*?* ***If not Screenee, ask*** *"May I please speak with* [insert Screenee's name]*?*

(b) ***Interviewer Script***: *I am calling from* [insert your institution here] *about the Long Life Family Study, a family study of longevity. We hope to discover how some people and their families live long lives. I am calling because we sent you some information about our study and we would like to provide you with some additional background to help you decide if you wish to participate in this study. May I ask who I am speaking with? Are you* [insert Screenee's name]*?* ***If not Screenee, ask*** *"May I please speak with* [insert Screenee's name]*?*

Participant ID: ______________________________ **Participant Name Code:** ________________________

***Interviewer**:* *Please complete the following questions before beginning:*

Are you speaking with Screenee?

- ☐[1] ....Yes **Go to Verbal Consent Script *(below)***
- ☐[0] ....No

If you are not speaking with the Screenee, then ask:

What is your relationship to ___________________________ [name of Screenee]?

- ☐[1] ....Spouse
- ☐[2] ....Sibling
- ☐[3] ....Offspring
- ☐[4] ....Grandchild
- ☐[5] ....Niece
- ☐[5] ....Nephew
- ☐[6] ....Other (Please Specify)_________________________

Interviewer: *Is it okay to speak with you now and tell you more about this project? This will take approximately 15 minutes.*

Verbal Consent Script: *We're asking* [you/Screenee] *to participate in an important international study of long life, particularly within families.* [Your/Screenee's] *name was chosen at random from a list of older Medicare users. The Centers for Medicare and Medicaid Services is cooperating with the National Institutes of Health on this study. Two other American universities* [insert names here] *and the University of Southern Denmark are also part of this study. Our goal is to find out what common traits are present in families with long-lived individuals. By sharing information with us, you can help improve the health of future generations, including your own children and grandchildren.*

Before inviting people to participate in our study, we would like to ask some questions to see if [your/Screenee's] *family is eligible. We are interested in families with long-lived individuals. You do not have to answer any questions that you do not feel comfortable with. All of the information that I receive from you, including your name and any other identifying information, will be kept strictly confidential and secured under lock and key. Please note, none of the study investigators involved, nor their staff members, will attempt to identify or contact specific family members based on the relationship information obtained during this phase of the interview. Your participation is voluntary; you do not have to answer these questions.*

1. Do I have permission to ask some questions about *[yourself/Screenee]* and *[your/his or her]* family?

- ☐[1] ....Yes
- ☐[0] ....No ***Script**: Thank you very much for speaking with me.*

Thank you. First I would like to verify some information about *[you/Screenee]*.

2a. What is *[your/Screenee's]* full name?

_________________________________ _________. _______________________________
First Name **Middle Initial** **Last Name**

2b. What is *[your/Screenee's]* home address? ____

____

____ _____________________ ____ ____ ____ ____ ____
City State Zip Code

2c. What is *[your/Screenee's]* home telephone number?

(____ ____ ____) ____ ____ ____ **-** ____ ____ ____ ____

2d. Is there another number to call that is better during the day?

(____ ____ ____) ____ ____ ____ **-** ____ ____ ____ ____

***Interviewer*: If you are not speaking with the Screenee, please obtain the following contact information for the Reporter.**

3a. What is your full name?

_________________________________ _________. _______________________________
First Name **Middle Initial** **Last Name**

3b. What is your address:

____

____ _____________________ ____ ____ ____ ____ ____
City State Zip Code

3c. What is your home telephone number?

(____ ____ ____) ____ ____ ____ **-** ____ ____ ____ ____

3d. Is there another number to call that is better during the day?

(____ ____ ____) ____ ____ ____ **-** ____ ____ ____ ____

4. How did [you/Screenee] hear about the study? (X all that apply)

☐[1] ....Brochure with Card
☐[1] ....Event
☐[1] ....Flyer
☐[1] ....Family Member
☐[1] ....Letter
☐[1] ....Don't Know
☐[1] ....Other (Please Specify)____________________________
☐[1] ....Magazine
☐[1] ....Newspaper Ad
☐[1] ....Referral
☐[1] ....Radio Ad
☐[1] ....Television Ad
☐[1] ....Refused

5a. ***Interviewer*: Is the Screenee located within an LLFS field center catchment area?**

☐[1] ....Yes
☐[0] ....No

5b. Do you have any living biological brothers or sisters?

☐[1] ....Yes **Go to Q5b**
☐[0] ....No **End Interview Using Script (a) Below**

5c. Do you have any living biological children or living biological nieces/nephews?

☐[1] ....Yes **Continue Interview Using Script (b) Below**
☐[0] ....No **End Interview Using Script (a) Below**

(a) If Interview is terminated, say the following: *"We greatly appreciate the time and interest you have shown in our study. The information you provided is very important and will help us discover how some people and their families live to a very old age."*

(b) If Interview is continuing, say the following: *"Now we would like to ask you a few questions that may help us to learn more about how some people and their families live long lives."*

6a. What is [your/Screenee's] age? ____ ____ ____ Years

6b. What is [your/Screenee's] date of birth?

Day: ____ ____ Month: ____ ____ ____ Year: ____ ____ ____ ____
(Example: ddMMMyyyy – e.g., 10JUN2005)

7. What is [your/Screenee's] gender?

☐[1] ....Male
☐[2] ....Female

---

8. *[Are you / Is Screenee]* Spanish, Hispanic or Latino?

- ☐1 ....Yes, Mexican, Mexican/Chicano
- ☐2 ....Yes, Puerto Rican
- ☐3 ....Yes, Cuban
- ☐4 ....Yes, Other Spanish/Hispanic/Latino
- ☐0 ....No, not Spanish/Hispanic/Latino
- ☐R ....Refused

9. What is *[your/Screenee's]* race? (X all that apply)

- ☐1 ....White
- ☐1 ....Black or African American
- ☐1 ....American Indian or Alaska Native
- ☐1 ....Asian
- ☐1 ....Native Hawaiian or other Pacific Islander)
- ☐1 ....Other (Please Specify ___________________________
- ☐1 ....Refused

Interviewer*:** **[Skip Q10 if Screenee is not the Proband.]** ***Does a hearing, language barrier or other problem make the Screenee unable to communicate with you?

10. ☐1 ....Yes **End Interview (Use Script Below)**
☐0 ....No

End Interview Script: *"We greatly appreciate the time and interest you have shown in our study. The information you provided is very important and will help us discover how some people and their families live to a very old age. This is all the information that we need from you. Thank you so much."*

Interviewer Script: *Now I would like to ask you some questions about* [your/Screenee's] *general health and any serious medical conditions that you may have.*

11. In general, how would you say *[your/Screenee's]* health is? Would you say it is…

- ☐5 ....Excellent
- ☐4 ....Very Good
- ☐3 ....Good
- ☐2 ....Fair
- ☐1 ....Poor
- ☐D ....Don't Know
- ☐R ....Refused

***Interviewer:* If speaking with the Screenee then complete questions 12, 13a and 13b; otherwise, skip to question 14a.**

12. Do you have advanced cancer or a serious medical condition such as one requiring oxygen or dialysis that would keep you from being able to participate in a home interview or a physical examination?

☐ 1 ....Yes, definitely physically unable
☐ 0 ....No, definitely physically able
☐ D ....Maybe

Interviewer Script: *Before we continue, I want to ask you: "What is your understanding of the purpose of the LONG LIFE Family Study?"*

13a. Key elements (concepts), "family, long lived, research study" ________________________

____

____

***Interviewer:* Does the Screenee appear to have a clear understanding of the purpose of the study?**

13b. ☐ 1 ....Yes **Go to Q15a**
☐ 0 ....No **End Interview (Use Script Below)**

If Speaking With a Reporter, ask:

14a. Will a hearing or other serious health problem such as one requiring oxygen or dialysis make it difficult for *[Screenee]* to participate in a home interview or physical examination?

☐ 1 ....Yes, definitely physically unable
☐ 0 ....No, definitely physically able
☐ D ....Maybe

14b. Do you think *[he/she]* would understand the purpose of this study in order to provide informed consent?

☐ 1 ....Yes
☐ 0 ....No **End Interview (Use Script Below)**

End Interview Script: *"We greatly appreciate the time and interest you have shown in our study. The information you provided is very important and will help us discover how some people and their families live to a very old age. This is all the information that we need from you. Thank you so much."*

Interviewer Script: *Now I would like to ask you some questions about long life in* [your/Screenee's] *family. Please feel free to tell me if you don't know the answer to a question, but please try to give your best guess. I am going to ask about* [your/Screenee's] *biological children, and* [your/Screenee's] *biological brothers and sisters. We are using the word "biological" to describe individuals that are related to* [you/Screenee] *by birth, meaning that they share a blood relationship with* [you/Screenee]. *Please note that none of the study investigators will attempt to identify, or contact specific family members based on the relationship information you provide unless you give us consent to do so at a later point in time.*

15a. How many living biological sons do *[you/he or she]* have? Remember to answer for blood relationships only. ______ Sons

15b. How many of these sons currently live within 2-3 hours of _______ Field Center *[put in your city/state]*. ______ Sons

16a. How many living biological daughters do *[you/he or she]* have? Remember to answer for blood relationships only. ______ Daughters

16b. How many of these daughters currently live within 2-3 hours of _______ Field Center *[put in your city/state]*. ______ Daughters

---

17a. How many full-brothers and sisters do *[you/he or she]* have? Include those that are living and those that are deceased. Remember, please answer for blood relationships only.

_____ Full-Brothers _____ Full-Sisters

17b. How many half-brothers and sisters do *[you/he or she]* have? Include those that are living and those that are deceased. Remember, please answer for blood relationships only.

_____ Half-Brothers _____ Half-Sisters

Interviewer Script: *Now I'm going to ask you some questions about each of [your/Screenee's] biological brothers and sisters. I'm going to ask you the questions in order of oldest to youngest. Let's begin.*

Interviewer Note: If needed to guide the interview, list the first name of each sibling in birth order on a separate piece of paper. Please destroy this page upon final determination of eligibility.

Proceed to Page 8.

18a. Is *[your/his or her]* oldest sibling a full- or half-brother or sister?

- ☐[1] ....Full-Brother
- ☐[2] ....Full-Sister
- ☐[3] ....Half-Brother
- ☐[4] ....Half-Sister

18b. Is this sibling older or younger than you?

- ☐[1] ....Older
- ☐[2] ....Younger
- ☐[3] ....Twin/Same Age

18c. Is this *[brother/sister]* still living?

- ☐[1] ....Yes
- ☐[0] ....No **Go to Q18j**
- ☐[D] ....Don't Know **Go to Q18o**

18d. What is *[his/her]* current age in years? ____ ____ ____ years **If Don't Know, enter D**

18d1. ☐ ***Interviewer: Check this box if 18d is an estimate***

18e. Where does *[he/she]* live?

City: ___________________________________ State: _____________

Country: _______________________________

18f. ***Interviewer:*** Does this person live within 2-3 hours of the New York City, Boston, MA, or Pittsburgh, PA field centers?

- ☐[1] ....Yes
- ☐[0] ....No

18g. How is *[his/her]* health?

- ☐[5] ....Excellent
- ☐[4] ....Very Good
- ☐[3] ....Good
- ☐[2] ....Fair
- ☐[1] ....Poor
- ☐[D] ....Don't Know
- ☐[R] ....Refused

18h. How many living biological children does this *[brother/sister]* have? _____ _____ children

18i. How many of these living biological children live within 2-3 hours of the New York City, NY, Boston, MA, or Pittsburgh, PA field centers? _____ _____ children

[Go to Q19a]

Interviewer*:** ***If brother or sister is deceased:

18j. What was *[his/her]* age at time of death? ____ ____ ____ years **If Don't Know, enter D**
18j1. ☐ ***Interviewer: Check this box if 18j is an estimate***

18k. What was *[his/her]* year of birth? ____ ____ ____ ____
18k1. ☐ ***Interviewer: Check this box if 18k is an estimate***

18l. Did *[he/she]* die as a result of an accident, injury or war? ***[Interviewer: If death is result of murder or suicide, answer "Yes"; if death occurred during childbirth, answer "No".]***

☐ 1 ....Yes
☐ 0 ....No
☐ D ....Don't Know
☐ R ....Refused

18m. How many living biological children does this *[brother/sister]* have? ____ ____ children

18n. How many of these living biological children live within 2-3 hours of the New York City, NY, Boston, MA, or Pittsburgh, PA field centers? _____ _____ children **[Go to Q19a]**

Interviewer*:** ***If vital status of brother or sister is unknown:

18o. What was *[his/her]* age the last time you spoke with *[him/her]*? ___ ___ __ years **If Don't Know, enter D**
18o1. ☐ ***Interviewer: Check this box if 18o is an estimate***

18p. What was *[his/her]* year of birth? ____ ____ ____ ____
18p1. ☐ ***Interviewer: Check this box if 18p is an estimate***

18q. How many living biological children does this *[brother/sister]* have? ____ ____ children

18r. How many of these living biological children live within 2-3 hours of the New York City, NY, Boston, MA, or Pittsburgh, PA field centers? _____ _____ children **[Go to Q19a]**

19a. Is *[your/his or her]* second oldest sibling a full- or half-brother or sister?

☐1 ....Full-Brother ☐3 ....Half-Brother
☐2 ....Full-Sister ☐4 ....Half-Sister

19b. Is this sibling older or younger than you?

☐1 ....Older
☐2 ....Younger
☐3 ....Twin/Same Age

19c. Is this *[brother/sister]* still living?

☐1 ....Yes
☐0 ....No **Go to Q19j**
☐D ....Don't Know **Go to Q19o**

19d. What is *[his/her]* current age in years? ____ ____ ____ years **If Don't Know, enter D**
19d1. ☐ ***Interviewer: Check this box if 19d is an estimate***

19e. Where does *[he/she]* live?

City: ________________________________ State: ____________

Country: ____________________________

19f. ***Interviewer:*** Does this person live within 2-3 hours of the New York City, Boston, MA, or Pittsburgh, PA field centers?

☐1 ....Yes
☐0 ....No

19g. How is *[his/her]* health?

☐5 ....Excellent
☐4 ....Very Good
☐3 ....Good
☐2 ....Fair
☐1 ....Poor
☐D ....Don't Know
☐R ....Refused

19h. How many living biological children does this *[brother/sister]* have? _____ _____ children

19i. How many of these living biological children live within 2-3 hours of the New York City, NY, Boston, MA, or Pittsburgh, PA field centers? _____ _____ children

[Go to Q20a]

***Interviewer*: If brother or sister is deceased:**

19j. What was *[his/her]* age at time of death? ____ ____ ____ years **If Don't Know, enter D**

19j1. ☐ ***Interviewer: Check this box if 19j is an estimate***

19k. What was *[his/her]* year of birth? ____ ____ ____ ____

19k1. ☐ ***Interviewer: Check this box if 19k is an estimate***

19l. Did *[he/she]* die as a result of an accident, injury or war? ***[Interviewer: If death is result of murder or suicide, answer "Yes"; if death occurred during childbirth, answer "No".]***

☐1 ....Yes
☐0 ....No
☐D ....Don't Know
☐R ....Refused

19m. How many living biological children does this *[brother/sister]* have? ____ ____ children

19n. How many of these living biological children live within 2-3 hours of the New York City, NY, Boston, MA, or Pittsburgh, PA field centers? _____ _____ children **[Go to Q20a]**

***Interviewer*: If vital status of brother or sister is *unknown*:**

19o. What was *[his/her]* age the last time you spoke with *[him/her]*? ___ ___ __ years **If Don't Know, enter D**

19o1. ☐ ***Interviewer: Check this box if 19o is an estimate***

19p. What was *[his/her]* year of birth? ____ ____ ____ ____

19p1. ☐ ***Interviewer: Check this box if 19p is an estimate***

19q. How many living biological children does this *[brother/sister]* have? ____ ____ children

19r. How many of these living biological children live within 2-3 hours of the New York City, NY, Boston, MA, or Pittsburgh, PA field centers? _____ _____ children **[Go to Q20a]**

20a. Is *[your/his or her]* third oldest sibling a full- or half-brother or sister?

☐1 ....Full-Brother ☐3 ....Half-Brother
☐2 ....Full-Sister ☐4 ....Half-Sister

20b. Is this sibling older or younger than you?

☐1 ....Older
☐2 ....Younger
☐3 ....Twin/Same Age

20c. Is this *[brother/sister]* still living?

☐1 ....Yes
☐0 ....No **Go to Q20j**
☐D ....Don't Know **Go to Q20o**

20d. What is *[his/her]* current age in years? ____ ____ ____ years **If Don't Know, enter D**
20d1. ☐ ***Interviewer: Check this box if 20d is an estimate***

20e. Where does *[he/she]* live?

City: ________________________________ State: ____________

Country: ____________________________

20f. ***Interviewer:*** Does this person live within 2-3 hours of the New York City, Boston, MA, or Pittsburgh, PA field centers?

☐1 ....Yes
☐0 ....No

20g. How is *[his/her]* health?

☐5 ....Excellent
☐4 ....Very Good
☐3 ....Good
☐2 ....Fair
☐1 ....Poor
☐D ....Don't Know
☐R ....Refused

20h. How many living biological children does this *[brother/sister]* have? _____ _____ children

20i. How many of these living biological children live within 2-3 hours of the New York City, NY, Boston, MA, or Pittsburgh, PA field centers? _____ _____ children

[Go to Q21a]

***Interviewer:** If brother or sister is deceased:*

20j. What was *[his/her]* age at time of death? ____ ____ ____ years **If Don't Know, enter D**

20j1. ☐ ***Interviewer:* Check this box if 20j is an estimate**

20k. What was *[his/her]* year of birth? ____ ____ ____ ____

20k1. ☐ ***Interviewer:* Check this box if 20k is an estimate**

20l. Did *[he/she]* die as a result of an accident, injury or war? ***[Interviewer:* If death is result of murder or suicide, answer "Yes"; if death occurred during childbirth, answer "No".]**

- ☐[1] ....Yes
- ☐[0] ....No
- ☐[D] ....Don't Know
- ☐[R] ....Refused

20m. How many living biological children does this *[brother/sister]* have? ____ ____ children

20n. How many of these living biological children live within 2-3 hours of the New York City, NY, Boston, MA, or Pittsburgh, PA field centers? _____ _____ children **[Go to Q21a]**

***Interviewer:** If vital status of brother or sister is unknown:*

20o. What was *[his/her]* age the last time you spoke with *[him/her]*? ___ ___ __ years **If Don't Know, enter D**

20o1. ☐ ***Interviewer:* Check this box if 20o is an estimate**

20p. What was *[his/her]* year of birth? ____ ____ ____ ____

20p1. ☐ ***Interviewer:* Check this box if 20p is an estimate**

20q. How many living biological children does this *[brother/sister]* have? ____ ____ children

20r. How many of these living biological children live within 2-3 hours of the New York City, NY, Boston, MA, or Pittsburgh, PA field centers? _____ _____ children **[Go to Q21a]**

21a. Is *[<u>your/his or her</u>]* <u>fourth oldest</u> sibling a full- or half-brother or sister?

☐[1] ....Full-Brother ☐[3] ....Half-Brother
☐[2] ....Full-Sister ☐[4] ....Half-Sister

21b. Is this sibling older or younger than you?

☐[1] ....Older
☐[2] ....Younger
☐[3] ....Twin/Same Age

21c. Is this *[<u>brother/sister</u>]* still living?

☐[1] ....Yes
☐[0] ....No **Go to Q21j**
☐[D] ....Don't Know **Go to Q21o**

21d. What is *[<u>his/her</u>]* current age in years? ____ ____ ____ years **If Don't Know, enter D**
21d1. ☐ ***Interviewer: Check this box if 21d is an estimate***

21e. Where does *[<u>he/she</u>]* live?

City: ________________________________ State: ____________

Country: ____________________________

21f. ***<u>Interviewer</u>:*** Does this person live within 2-3 hours of the New York City, Boston, MA, or Pittsburgh, PA field centers?

☐[1] ....Yes
☐[0] ....No

21g. How is *[<u>his/her</u>]* health?

☐[5] ....Excellent
☐[4] ....Very Good
☐[3] ....Good
☐[2] ....Fair
☐[1] ....Poor
☐[D] ....Don't Know
☐[R] ....Refused

21h. How many living biological children does this *[<u>brother/sister</u>]* have? _____ _____ children

21i. How many of these living biological children live within 2-3 hours of the New York City, NY, Boston, MA, or Pittsburgh, PA field centers? _____ _____ children

[Go to Q22a]

***Interviewer*: If brother or sister is deceased:**

21j. What was *[his/her]* age at time of death? ____ ____ ____ years **If Don't Know, enter D**

21j1. ☐ ***Interviewer: Check this box if 21j is an estimate***

21k. What was *[his/her]* year of birth? ____ ____ ____ ____

21k1. ☐ ***Interviewer: Check this box if 21k is an estimate***

21l. Did *[he/she]* die as a result of an accident, injury or war? ***[Interviewer: If death is result of murder or suicide, answer "Yes"; if death occurred during childbirth, answer "No".]***

- ☐ 1 ....Yes
- ☐ 0 ....No
- ☐ D ....Don't Know
- ☐ R ....Refused

21m. How many living biological children does this *[brother/sister]* have? ____ ____ children

21n. How many of these living biological children live within 2-3 hours of the New York City, NY, Boston, MA, or Pittsburgh, PA field centers? _____ _____ children **[Go to Q22a]**

***Interviewer*: If vital status of brother or sister is *unknown*:**

21o. What was *[his/her]* age the last time you spoke with *[him/her]*? ___ ___ __ years **If Don't Know, enter D**

21o1. ☐ ***Interviewer: Check this box if 21o is an estimate***

21p. What was *[his/her]* year of birth? ____ ____ ____ ____

21p1. ☐ ***Interviewer: Check this box if 21p is an estimate***

21q. How many living biological children does this *[brother/sister]* have? ____ ____ children

21r. How many of these living biological children live within 2-3 hours of the New York City, NY, Boston, MA, or Pittsburgh, PA field centers? _____ _____ children **[Go to Q22a]**

22a. Is *[your/his or her]* fifth oldest sibling a full- or half-brother or sister?

☐[1] ....Full-Brother ☐[3] ....Half-Brother
☐[2] ....Full-Sister ☐[4] ....Half-Sister

22b. Is this sibling older or younger than you?

☐[1] ....Older
☐[2] ....Younger
☐[3] ....Twin/Same Age

22c. Is this *[brother/sister]* still living?

☐[1] ....Yes
☐[0] ....No **Go to Q22j**
☐[D] ....Don't Know **Go to Q22o**

22d. What is *[his/her]* current age in years? ____ ____ ____ years **If Don't Know, enter D**
22d1. ☐ ***Interviewer: Check this box if 22d is an estimate***

22e. Where does *[he/she]* live?

City: ___________________________________ State: _____________

Country: ________________________________

22f. ***Interviewer:*** Does this person live within 2-3 hours of the New York City, Boston, MA, or Pittsburgh, PA field centers?

☐[1] ....Yes
☐[0] ....No

22g. How is *[his/her]* health?

☐[5] ....Excellent
☐[4] ....Very Good
☐[3] ....Good
☐[2] ....Fair
☐[1] ....Poor
☐[D] ....Don't Know
☐[R] ....Refused

22h. How many living biological children does this *[brother/sister]* have? _____ _____ children

22i. How many of these living biological children live within 2-3 hours of the New York City, NY, Boston, MA, or Pittsburgh, PA field centers? _____ _____ children

[Go to Q23a]

***Interviewer:* If brother or sister is deceased:**

22j. What was *[his/her]* age at time of death? ____ ____ ____ years **If Don't Know, enter D**

22j1. ☐ ***Interviewer: Check this box if 22j is an estimate***

22k. What was *[his/her]* year of birth? ____ ____ ____ ____

22k1. ☐ ***Interviewer: Check this box if 22k is an estimate***

22l. Did *[he/she]* die as a result of an accident, injury or war? ***[Interviewer: If death is result of murder or suicide, answer "Yes"; if death occurred during childbirth, answer "No".]***

- ☐ 1 ....Yes
- ☐ 0 ....No
- ☐ D ....Don't Know
- ☐ R ....Refused

22m. How many living biological children does this *[brother/sister]* have? ____ ____ children

22n. How many of these living biological children live within 2-3 hours of the New York City, NY, Boston, MA, or Pittsburgh, PA field centers? _____ _____ children **[Go to Q23a]**

***Interviewer:* If vital status of brother or sister is *unknown*:**

22o. What was *[his/her]* age the last time you spoke with *[him/her]*? ___ ___ __ years **If Don't Know, enter D**

22o1. ☐ ***Interviewer: Check this box if 22o is an estimate***

22p. What was *[his/her]* year of birth? ____ ____ ____ ____

22p1. ☐ ***Interviewer: Check this box if 22p is an estimate***

22q. How many living biological children does this *[brother/sister]* have? ____ ____ children

22r. How many of these living biological children live within 2-3 hours of the New York City, NY, Boston, MA, or Pittsburgh, PA field centers? _____ _____ children **[Go to Q23a]**

23a. Is *[your/his or her]* sixth oldest sibling a full- or half-brother or sister?

- ☐ 1 ....Full-Brother
- ☐ 2 ....Full-Sister
- ☐ 3 ....Half-Brother
- ☐ 4 ....Half-Sister

23b. Is this sibling older or younger than you?

- ☐ 1 ....Older
- ☐ 2 ....Younger
- ☐ 3 ....Twin/Same Age

23c. Is this *[brother/sister]* still living?

- ☐ 1 ....Yes
- ☐ 0 ....No **Go to Q23j**
- ☐ D ....Don't Know **Go to Q23o**

23d. What is *[his/her]* current age in years? ____ ____ ____ years **If Don't Know, enter D**

23d1. ☐ ***Interviewer: Check this box if 23d is an estimate***

23e. Where does *[he/she]* live?

City: ________________________________ State: ____________

Country: _____________________________

23f. ***Interviewer:*** Does this person live within 2-3 hours of the New York City, Boston, MA, or Pittsburgh, PA field centers?

- ☐ 1 ....Yes
- ☐ 0 ....No

23g. How is *[his/her]* health?

- ☐ 5 ....Excellent
- ☐ 4 ....Very Good
- ☐ 3 ....Good
- ☐ 2 ....Fair
- ☐ 1 ....Poor
- ☐ D ....Don't Know
- ☐ R ....Refused

23h. How many living biological children does this *[brother/sister]* have? _____ _____ children

23i. How many of these living biological children live within 2-3 hours of the New York City, NY, Boston, MA, or Pittsburgh, PA field centers? _____ _____ children

[Go to Q24a]

<u>Interviewer</u>: If brother or sister is deceased:

23j. What was *[<u>his/her</u>]* age at time of death? ____ ____ ____ years **If Don't Know, enter D**
23j1. ☐ ***Interviewer: Check this box if 23j is an estimate***

23k. What was *[<u>his/her</u>]* year of birth? ____ ____ ____ ____
23k1. ☐ ***Interviewer: Check this box if 23k is an estimate***

23l. Did *[<u>he/she</u>]* die as a result of an accident, injury or war? ***[<u>Interviewer</u>: If death is result of murder or suicide, answer "Yes"; if death occurred during childbirth, answer "No".]***

☐ 1 ....Yes
☐ 0 ....No
☐ D ....Don't Know
☐ R ....Refused

23m. How many living biological children does this *[<u>brother/sister</u>]* have? ____ ____ children

23n. How many of these living biological children live within 2-3 hours of the New York City, NY, Boston, MA, or Pittsburgh, PA field centers? _____ _____ children **[Go to Q24a]**

<u>Interviewer</u>: If vital status of brother or sister is <u>unknown</u>:

23o. What was *[<u>his/her</u>]* age the last time you spoke with *[him/her]*? ___ ___ __ years **If Don't Know, enter D**
23o1. ☐ ***Interviewer: Check this box if 23o is an estimate***

23p. What was *[<u>his/her</u>]* year of birth? ____ ____ ____ ____
23p1. ☐ ***Interviewer: Check this box if 23p is an estimate***

23q. How many living biological children does this *[<u>brother/sister</u>]* have? ____ ____ children

23r. How many of these living biological children live within 2-3 hours of the New York City, NY, Boston, MA, or Pittsburgh, PA field centers? _____ _____ children **[Go to Q24a]**

24a. Is *[your/his or her]* seventh oldest sibling a full- or half-brother or sister?

☐[1] ....Full-Brother ☐[3] ....Half-Brother
☐[2] ....Full-Sister ☐[4] ....Half-Sister

24b. Is this sibling older or younger than you?

☐[1] ....Older
☐[2] ....Younger
☐[3] ....Twin/Same Age

24c. Is this *[brother/sister]* still living?

☐[1] ....Yes
☐[0] ....No **Go to Q24j**
☐[D] ....Don't Know **Go to Q24o**

24d. What is *[his/her]* current age in years? ____ ____ ____ years **If Don't Know, enter D**
24d1. ☐ ***Interviewer: Check this box if 24d is an estimate***

24e. Where does *[he/she]* live?

City: ________________________________ State: ____________

Country: _____________________________

24f. ***Interviewer:*** Does this person live within 2-3 hours of the New York City, Boston, MA, or Pittsburgh, PA field centers?

☐[1] ....Yes
☐[0] ....No

24g. How is *[his/her]* health?

☐[5] ....Excellent
☐[4] ....Very Good
☐[3] ....Good
☐[2] ....Fair
☐[1] ....Poor
☐[D] ....Don't Know
☐[R] ....Refused

24h. How many living biological children does this *[brother/sister]* have? _____ _____ children

24i. How many of these living biological children live within 2-3 hours of the New York City, NY, Boston, MA, or Pittsburgh, PA field centers? _____ _____ children

[Go to Q25a]

***Interviewer**: If brother or sister is deceased:*

24j. What was *[his/her]* age at time of death? ____ ____ ____ years **If Don't Know, enter D**

24j1. ☐ ***Interviewer: Check this box if 24j is an estimate***

24k. What was *[his/her]* year of birth? ____ ____ ____ ____

24k1. ☐ ***Interviewer: Check this box if 24k is an estimate***

24l. Did *[he/she]* die as a result of an accident, injury or war? ***[Interviewer: If death is result of murder or suicide, answer "Yes"; if death occurred during childbirth, answer "No".]***

☐ 1 ....Yes
☐ 0 ....No
☐ D ....Don't Know
☐ R ....Refused

24m. How many living biological children does this *[brother/sister]* have? ____ ____ children

24n. How many of these living biological children live within 2-3 hours of the New York City, NY, Boston, MA, or Pittsburgh, PA field centers? _____ _____ children **[Go to Q25a]**

***Interviewer**: If vital status of brother or sister is **unknown**:*

24o. What was *[his/her]* age the last time you spoke with *[him/her]*? ___ ___ __ years **If Don't Know, enter D**

24o1. ☐ ***Interviewer: Check this box if 24o is an estimate***

24p. What was *[his/her]* year of birth? ____ ____ ____ ____

24p1. ☐ ***Interviewer: Check this box if 24p is an estimate***

24q. How many living biological children does this *[brother/sister]* have? ____ ____ children

24r. How many of these living biological children live within 2-3 hours of the New York City, NY, Boston, MA, or Pittsburgh, PA field centers? _____ _____ children **[Go to Q25a]**

25a. Is *[your/his or her]* eighth oldest sibling a full- or half-brother or sister?

☐[1] ....Full-Brother ☐[3] ....Half-Brother
☐[2] ....Full-Sister ☐[4] ....Half-Sister

25b. Is this sibling older or younger than you?

☐[1] ....Older
☐[2] ....Younger
☐[3] ....Twin/Same Age

25c. Is this *[brother/sister]* still living?

☐[1] ....Yes
☐[0] ....No **Go to Q25j**
☐[D] ....Don't Know **Go to Q25o**

25d. What is *[his/her]* current age in years? ____ ____ ____ years **If Don’t Know, enter D**
25d1. ☐ ***Interviewer: Check this box if 25d is an estimate***

25e. Where does *[he/she]* live?

City: ___________________________________ State: _____________

Country: ________________________________

25f. ***Interviewer:*** Does this person live within 2-3 hours of the New York City, Boston, MA, or Pittsburgh, PA field centers?

☐[1] ....Yes
☐[0] ....No

25g. How is *[his/her]* health?

☐[5] ....Excellent
☐[4] ....Very Good
☐[3] ....Good
☐[2] ....Fair
☐[1] ....Poor
☐[D] ....Don't Know
☐[R] ....Refused

25h. How many living biological children does this *[brother/sister]* have? _____ _____ children

25i. How many of these living biological children live within 2-3 hours of the New York City, NY, Boston, MA, or Pittsburgh, PA field centers? _____ _____ children

[Go to Q26a]

***Interviewer**: If brother or sister is deceased:*

25j. What was *[his/her]* age at time of death? ____ ____ ____ years **If Don't Know, enter D**

25j1. ☐ ***Interviewer: Check this box if 25j is an estimate***

25k. What was *[his/her]* year of birth? ____ ____ ____ ____

25k1. ☐ ***Interviewer: Check this box if 25k is an estimate***

25l. Did *[he/she]* die as a result of an accident, injury or war? ***[Interviewer: If death is result of murder or suicide, answer "Yes"; if death occurred during childbirth, answer "No".]***

- ☐ 1 ....Yes
- ☐ 0 ....No
- ☐ D ....Don't Know
- ☐ R ....Refused

25m. How many living biological children does this *[brother/sister]* have? ____ ____ children

25n. How many of these living biological children live within 2-3 hours of the New York City, NY, Boston, MA, or Pittsburgh, PA field centers? _____ _____ children **[Go to Q26a]**

***Interviewer**: If vital status of brother or sister is **unknown**:*

25o. What was *[his/her]* age the last time you spoke with *[him/her]*? ___ ___ __ years **If Don't Know, enter D**

25o1. ☐ ***Interviewer: Check this box if 25o is an estimate***

25p. What was *[his/her]* year of birth? ____ ____ ____ ____

25p1. ☐ ***Interviewer: Check this box if 25p is an estimate***

25q. How many living biological children does this *[brother/sister]* have? ____ ____ children

25r. How many of these living biological children live within 2-3 hours of the New York City, NY, Boston, MA, or Pittsburgh, PA field centers? _____ _____ children **[Go to Q26a]**

26a. Is *[your/his or her]* ninth oldest sibling a full- or half-brother or sister?

- ☐[1] ....Full-Brother
- ☐[2] ....Full-Sister
- ☐[3] ....Half-Brother
- ☐[4] ....Half-Sister

26b. Is this sibling older or younger than you?

- ☐[1] ....Older
- ☐[2] ....Younger
- ☐[3] ....Twin/Same Age

26c. Is this *[brother/sister]* still living?

- ☐[1] ....Yes
- ☐[0] ....No **Go to Q26j**
- ☐[D] ....Don't Know **Go to Q26o**

26d. What is *[his/her]* current age in years? _____ _____ _____ years **If Don't Know, enter D**

26d1. ☐ ***Interviewer: Check this box if 26d is an estimate***

26e. Where does *[he/she]* live?

City: ________________________________ State: ____________

Country: ____________________________

26f. ***Interviewer:*** Does this person live within 2-3 hours of the New York City, Boston, MA, or Pittsburgh, PA field centers?

- ☐[1] ....Yes
- ☐[0] ....No

26g. How is *[his/her]* health?

- ☐[5] ....Excellent
- ☐[4] ....Very Good
- ☐[3] ....Good
- ☐[2] ....Fair
- ☐[1] ....Poor
- ☐[D] ....Don't Know
- ☐[R] ....Refused

26h. How many living biological children does this *[brother/sister]* have? _____ _____ children

26i. How many of these living biological children live within 2-3 hours of the New York City, NY, Boston, MA, or Pittsburgh, PA field centers? _____ _____ children

[Go to Q27a]

Interviewer*:** ***If brother or sister is deceased:

26j. What was *[his/her]* age at time of death? ____ ____ ____ years **If Don't Know, enter D**

26j1. ☐ ***Interviewer: Check this box if 26j is an estimate***

26k. What was *[his/her]* year of birth? ____ ____ ____ ____

26k1. ☐ ***Interviewer: Check this box if 26k is an estimate***

26l. Did *[he/she]* die as a result of an accident, injury or war? ***[Interviewer: If death is result of murder or suicide, answer "Yes"; if death occurred during childbirth, answer "No".]***

- ☐ 1 ....Yes
- ☐ 0 ....No
- ☐ D ....Don't Know
- ☐ R ....Refused

26m. How many living biological children does this *[brother/sister]* have? ____ ____ children

26n. How many of these living biological children live within 2-3 hours of the New York City, NY, Boston, MA, or Pittsburgh, PA field centers? _____ _____ children **[Go to Q27a]**

Interviewer*:** ***If vital status of brother or sister is unknown:

26o. What was *[his/her]* age the last time you spoke with *[him/her]*? ___ ___ __ years **If Don't Know, enter D**

26o1. ☐ ***Interviewer: Check this box if 26o is an estimate***

26p. What was *[his/her]* year of birth? ____ ____ ____ ____

26p1. ☐ ***Interviewer: Check this box if 26p is an estimate***

26q. How many living biological children does this *[brother/sister]* have? ____ ____ children

26r. How many of these living biological children live within 2-3 hours of the New York City, NY, Boston, MA, or Pittsburgh, PA field centers? _____ _____ children **[Go to Q27a]**

27a. Is *[your/his or her]* tenth oldest sibling a full- or half-brother or sister?

☐1 ....Full-Brother ☐3 ....Half-Brother
☐2 ....Full-Sister ☐4 ....Half-Sister

27b. Is this sibling older or younger than you?

☐1 ....Older
☐2 ....Younger
☐3 ....Twin/Same Age

27c. Is this *[brother/sister]* still living?

☐1 ....Yes
☐0 ....No **Go to Q27j**
☐D ....Don't Know **Go to Q27o**

27d. What is *[his/her]* current age in years? ____ ____ ____ years **If Don't Know, enter D**
27d1. ☐ ***Interviewer: Check this box if 27d is an estimate***

27e. Where does *[he/she]* live?

City: ___________________________________ State: _____________

Country: _______________________________

27f. ***Interviewer:*** Does this person live within 2-3 hours of the New York City, Boston, MA, or Pittsburgh, PA field centers?

☐1 ....Yes
☐0 ....No

27g. How is *[his/her]* health?

☐5 ....Excellent
☐4 ....Very Good
☐3 ....Good
☐2 ....Fair
☐1 ....Poor
☐D ....Don't Know
☐R ....Refused

27h. How many living biological children does this *[brother/sister]* have? _____ _____ children

27i. How many of these living biological children live within 2-3 hours of the New York City, NY, Boston, MA, or Pittsburgh, PA field centers? _____ _____ children

[Go to Q28]

***Interviewer*: If brother or sister is deceased:**

27j. What was *[his/her]* age at time of death? ____ ____ ____ years **If Don't Know, enter D**
27j1. ☐ ***Interviewer: Check this box if 27j is an estimate***

27k. What was *[his/her]* year of birth? ____ ____ ____ ____
27k1. ☐ ***Interviewer: Check this box if 27k is an estimate***

27l. Did *[he/she]* die as a result of an accident, injury or war? ***[Interviewer: If death is result of murder or suicide, answer "Yes"; if death occurred during childbirth, answer "No".]***

- ☐ 1 ....Yes
- ☐ 0 ....No
- ☐ D ....Don't Know
- ☐ R ....Refused

27m. How many living biological children does this *[brother/sister]* have? ____ ____ children

27n. How many of these living biological children live within 2-3 hours of the New York City, NY, Boston, MA, or Pittsburgh, PA field centers? _____ _____ children **[Go to Q28]**

***Interviewer*: If vital status of brother or sister is *unknown*:**

27o. What was *[his/her]* age the last time you spoke with *[him/her]*? ___ ___ __ years **If Don't Know, enter D**
27o1. ☐ ***Interviewer: Check this box if 27o is an estimate***

27p. What was *[his/her]* year of birth? ____ ____ ____ ____
27p1. ☐ ***Interviewer: Check this box if 27p is an estimate***

27q. How many living biological children does this *[brother/sister]* have? ____ ____ children

27r. How many of these living biological children live within 2-3 hours of the New York City, NY, Boston, MA, or Pittsburgh, PA field centers? _____ _____ children **[Go to Q28]**

***Interviewer*: If the Screenee has more than 10 biological siblings, please use additional forms to record the information for these brothers and sisters.**

***Interviewer*: Calculate Family Longevity Selection Score (FLoSS):** ____________________________

For Interviewer Use – DO NOT ASK

Screenee Eligibility

28. Is the calculated Family Longevity Selection Score (FLoSS) ≥ ________?

☐[1] ....Yes
☐[0] ....No

29. Is the Screenee likely to be able to give informed consent?

☐[1] ....Yes
☐[0] ....No

30. Is the Screenee likely to be able to participate in the phenotype exam (no serious health condition/severe hearing loss)?

☐[1] ....Yes
☐[0] ....No
☐[D] ....Maybe

Family Eligibility

31. Are there at least two (2) recruitable siblings in this family (i.e. proband/sib, sib/sib)?

☐[1] ....Yes
☐[0] ....No

32. Does at least one (1) member of the core family (i.e. proband, sib or offspring) live within three (3) hours of one of the study field centers in New York City, NY, Boston, MA or Pittsburgh, PA?

☐[1] ....Yes
☐[0] ....No

Script 1*: *If yes for Questions 28-32, then continue with: [Your/Screenee's] *family is unusually long-lived compared to others we have screened. We are interested in finding out more about* [you/Screenee] *and* [your/his or her] *family so that we can discover how some people and their families live to a very old age. In this next part of the study, we would like to ask* [you/him or her] *and several members of* [your/his or her] *family to participate so that we can get a more complete picture of* [your/his or her] *family's structure, health and functioning. The study will collect information about* [your/his or her] *family's medical history and about* [your/his or her] *family members' physical activity and mental functions, such as memory. We will also ask* [you and your / Screenee and his/her] *family members for a blood sample so that we can look for factors in the blood that may increase chances for a long life and successful aging.*

(a) *Do you think that* [you/Screenee] *and other members of* [your/Screenee's] *family would be interested in participating in this next phase of the study, including providing a blood sample?*

☐1 ....Yes **Go to (b)**
☐0 ....No **Go to (j)**
☐D ....Don't Know (Pending) Specify: ________________ **End Interview/Call Back**

(b) *Do* [you/Screenee] *have a living spouse?*

☐1 ....Yes **Go to (c)**
☐0 ....No **Go to (h)**

(c) *Is* [he/she] *the biological* [father/mother] *of any of* [your/Screenee's] *children?*

☐1 ....Yes **Go to (d)**
☐0 ....No **Go to (h)**

(d) *What is* [his/her] *age?* ____ ____ ____ Years

(e) *Does* [he/she] *live with* [you/Screenee]*?*

☐1 ....Yes
☐0 ....No

(f) *Does* [your/Screenee's] *spouse have any living siblings age 85 and older?*

☐1 ....Yes
☐0 ....No

***Interviewer Note*: This question is to identify whether it is worthwhile to screen the spouse as a potential Proband.**

(g) *Would you be willing to ask [him/her] if we can contact them to discuss participation in this Study?*

☐1 ....Yes **Go to TS2; Record Contact Information; Go to (h)**
☐0 ....No **Go to (h)**

(h) *Before we move forward, would* [you/he or she] *also be willing to contact at least two family members (one sibling and one offspring) and ask them if we can contact them to discuss the study and answer any questions that they may have?*

☐1 ....Yes **Go to (i)**
☐0 ....No **Go to (j)**

(i) *May I ask you who you plan to contact; first names only?* ***[Interviewer: Please write down the first names of these individuals, in order of age, in the appropriate section on the Family Contact Information Form (TS2).]*** *We will call you back in one week to confirm with you that these family members are interested in speaking with us. Thank you.*

When is the best time to call you back? _____________ AM/PM _____________ Day

If you or your family member would like to contact us, we can be reached toll-free at XXX-XXX-XXXX.

(j) *Could you please indicate your reason(s)?* ***(Please X all that apply; then End Interview using Script #3 on following page).***

- ☐ 1 ....Not Interested
- ☐ 1 ....Not Enough Time
- ☐ 1 ....Unwilling to Provide Blood Sample
- ☐ 1 ....Not Well
- ☐ 1 ....Concern about Ability to Complete Examination
- ☐ 1 ....Privacy Issue/Concern
- ☐ 1 ....Unwilling to Contact Family Members
- ☐ 1 ....Lack of "Family" Interest
- ☐ 1 ....Other, Please Specify: ______________________________

Script 2: If NO to Family Longevity Selection Score (FLoSS) _________, but to all other questions YES, or meets all eligibility criteria except siblings and/or offspring are out of area, then say: *Thank you for your time. The information you provided is very important to us. It will help us discover how some people and their families live to a very old age. At this point in our study, we have all the information that we need for now. However, it is possible that as the study continues, we may need to speak with you again. Therefore, we would like to keep* [your/Screenee's] *contact information on file so that we may contact* [you/Screenee]*, if necessary.*

Interviewer: Please indicate . . .

- ☐ 1 ....Okay to Contact
- ☐ 0 ....Objects to Future Contact

We greatly appreciate the time and interest you have shown in our study.

Script 3 -- End Interview Script: **Regardless of Family Longevity Score, if answer was NO to either Screenee Eligibility Questions 29 or 30 (on Pages 20-21), or NO to Family Eligibility Questions 31 or 32, or NO to Q(a) or (b) above, then say:** *Thank you for your time. The information you provided is very important to us. It will help us discover how some people and their families live to a very old age. We greatly appreciate the time and interest you have shown in our study.*

LONG LIFE FAMILY STUDY

(Affix Label Here)

Participant ID: ____________________

Participant Name Code: ____________

Date Form Initiatied (e.g., 10JUN2005):

☐ ☐ ☐ ☐ ☐ ☐ ☐ ☐ ☐
d d m m m y y y y

Date Form Completed (e.g., 10JUN2005):

☐ ☐ ☐ ☐ ☐ ☐ ☐ ☐ ☐
d d m m m y y y y

Interviewer Code: ☐ ☐ ☐

Please Circle Field Center Location:
BU **CU** **DK** **UP**

LLFS Proband Relative Contact Information Form (TS2)

Interviewer Notes*:** ***Recommended Resources: Proband's completed TS1 Interview (or summary report).

This form is to be used to recontact the Proband, to identify his/her siblings and to record information on additional relatives who are willing to be contacted about participation in LLFS. Section 2 of this form is a continuous process and should be updated whenever additional family members are identified and have agreed to be contacted.

1. *Section 1: The first objective of this form is to identify the Proband's complete sibship (including full- or half-siblings). This is the first step in identifying a family Pedigree. It is important that this section be completed during this telephone contact.*

2. *Section 2: In this section, names of any other family members that have been identified and contacted by Proband or other family members, are to be recorded. If this person has agreed to be contacted, all contact information is to be obtained and documented.*

   ***Important Note*:** *Prior to initiating contact with any individual identified at this time, LLFS staff must query the existing database for each name obtained to determine if a Study ID had been previously assigned (i.e. via CMS list). This should also be done for those individuals who do not provide consent. If an ID has been assigned, that number is to be recorded in the space provided. For those who are not in the DES system, an ID will be assigned according to the protocol outlined in Chapter 4 of the LLFS MOP. The number should then be documented in the space provided on this form, as well as on the TS1a. Upon obtaining consent to contact, this ID number will be transferred to the TS1a; the TS1a will be entered using the individual's ID number, not the Proband's.*

3. *Section 3: The purpose of this section is to close the interview. The interviewer is to inform the Proband that additional questions regarding their parents, spouse(s) and offspring, will need to be ascertained at a later time (once the family is enrolled). Efforts should be made to obtain the information from the Proband, however, if they indicate they would prefer another family member be contacted to do this, the contact information should be documented.*

Note to Interviewer*:** ***If you need additional sheets for the following section, please Xerox the appropriate sheets and append to this form

Participant ID: ______________________________ **Participant Name Code:** _______________

Name of Proband *(Last, First, Middle; if Female, please include Maiden Name):* ____

Section 1 – Proband's Sibling(s): (Start from eldest to youngest, indicating Proband's place within this sibship; include full- or half-sibs)

Sibling #___

(a) Sibling's Name

Last: ______________________________

First: ______________________________

Middle: ______________________________

Maiden: ______________________________

(b) Relationship

☐[1] Full-Brother ☐[3] Half-Brother
☐[2] Full-Sister ☐[4] Half-Sister

(c) Indicate Vital Status

☐[0] Deceased **Go to (h)**
☐[1] Alive **Go to (d)**

(d) Have you told this sibling about the Study?

☐[1] Yes **Go to (f)**
☐[0] No **Go to (e)**

(e) Can you provide a reason why you have not yet contacted this family member about the study? ***[X all that apply]***

☐[1] He/She is cognitively impaired
☐[1] He/she is too ill to participate
☐[1] I am no longer in contact with him/her
☐[1] I do not believe he/she will be interested
☐[1] I do not believe he/she has time
☐[1] He/she lives out of the area
☐[1] I have not had time to contact him/her

(f) Is this individual willing to be contacted?

☐[1] Yes **Go to (i)**
☐[0] No **Go to (g)**
☐[D] Haven't Contacted/Undecided

(g) Can you provide a reason why this family member is not interested in our Study? ***[X all that apply]***

☐[1] He/She is cognitively impaired
☐[1] He/she is too ill to participate
☐[1] I am no longer in contact with him/her
☐[1] He/She is not interested in participating
☐[1] He/she does not have the time
☐[1] He/she lives out of the area
☐[1] I have not had time to contact him/her

(h) To your knowledge, did ____ ever suffer from any of the following conditions? ***[X all that apply]***

☐[1] Heart Disease
☐[1] Stroke
☐[1] Diabetes
☐[1] Hypertension
☐[1] Cancer
☐[1] Alzheimers/Dementia
☐[1] Other_______________

(i) Contact Information

Address: ____

____

Phone: __________________________ (☐ Home ☐ Work)

Best day/time to call: ______________________ E-Mail: ________________________________

Family Member Telephone Screener Completed? ☐ Yes ☐ No

Individual Eligible to Participate in Study? ☐ Yes ☐ No

Date Scheduled: ___ ___ / ___ ___ ___ / ___ ___ ___ ___

Date Completed: ___ ___ / ___ ___ ___ / ___ ___ ___ ___

LLFS Participant ID #: ___ ___ ___ ___ ___ ___ ___ ___

Participant ID: ____________________ **Participant Name Code:** ____________

Name of Proband *(Last, First, Middle; if Female, please include Maiden Name):* ______________________________

Section 1 – Proband's Sibling(s) - Continued: (Start from eldest to youngest, indicating Proband's place within this sibship)

Sibling #___

(a) Sibling's Name

Last: ____________________

First: ____________________

Middle: ____________________

Maiden: ____________________

(e) Can you provide a reason why you have not yet contacted this family member about the study? ***[X all that apply]***

- ☐[1] He/She is cognitively impaired
- ☐[1] He/she is too ill to participate
- ☐[1] I am no longer in contact with him/her
- ☐[1] I do not believe he/she will be interested
- ☐[1] I do not believe he/she has time
- ☐[1] He/she lives out of the area
- ☐[1] I have not had time to contact him/her

(g) Can you provide a reason why this family member is not interested in our Study? ***[X all that apply]***

- ☐[1] He/She is cognitively impaired
- ☐[1] He/she is too ill to participate
- ☐[1] I am no longer in contact with him/her
- ☐[1] He/She is not interested in participating
- ☐[1] He/she does not have the time
- ☐[1] He/she lives out of the area
- ☐[1] I have not had time to contact him/her

(b) Relationship

- ☐[1] Full-Brother
- ☐[2] Full-Sister
- ☐[3] Half-Brother
- ☐[4] Half-Sister

(c) Indicate Vital Status

- ☐[0] Deceased **Go to (h)**
- ☐[1] Alive **Go to (d)**

(f) Is this individual willing to be contacted?

- ☐[1] Yes **Go to (i)**
- ☐[0] No **Go to (g)**
- ☐[D] Haven't Contacted/Undecided

(h) To your knowledge, did ____ ever suffer from any of the following conditions? ***[X all that apply]***

- ☐[1] Heart Disease
- ☐[1] Stroke
- ☐[1] Diabetes
- ☐[1] Hypertension
- ☐[1] Cancer
- ☐[1] Alzheimers/Dementia
- ☐[1] Other______________

(d) Have you told this sibling about the Study?

- ☐[1] Yes **Go to (f)**
- ☐[0] No **Go to (e)**

(i) Contact Information

Address: ____

____

Phone: ____________________ (☐ Home ☐ Work)

Best day/time to call: ____________________ E-Mail: ____________________

Family Member Telephone Screener Completed? ☐ Yes ☐ No

Individual Eligible to Participate in Study? ☐ Yes ☐ No

Date Scheduled: ___ ___ / ___ ___ ___ / ___ ___ ___ ___

Date Completed: ___ ___ / ___ ___ ___ / ___ ___ ___ ___

LLFS Participant ID #: ___ ___ ___ ___ ___ ___ ___ ___

Participant ID: ___________________________ **Participant Name Code:** ___________

Name of Proband *(Last, First, Middle; if Female, please include Maiden Name):* ______________________________

Section 1 – Proband's Sibling(s) - Continued: (Start from eldest to youngest, indicating Proband's place within this sibship)

Sibling #___

(a) Sibling's Name

Last: ______________________

First: ______________________

Middle: ______________________

Maiden: ______________________

(e) Can you provide a reason why you have not yet contacted this family member about the study? ***[X all that apply]***

- ☐[1] He/She is cognitively impaired
- ☐[1] He/she is too ill to participate
- ☐[1] I am no longer in contact with him/her
- ☐[1] I do not believe he/she will be interested
- ☐[1] I do not believe he/she has time
- ☐[1] He/she lives out of the area
- ☐[1] I have not had time to contact him/her

(g) Can you provide a reason why this family member is not interested in our Study? ***[X all that apply]***

- ☐[1] He/She is cognitively impaired
- ☐[1] He/she is too ill to participate
- ☐[1] I am no longer in contact with him/her
- ☐[1] He/She is not interested in participating
- ☐[1] He/she does not have the time
- ☐[1] He/she lives out of the area
- ☐[1] I have not had time to contact him/her

(b) Relationship

- ☐[1] Full-Brother ☐[3] Half-Brother
- ☐[2] Full-Sister ☐[4] Half-Sister

(c) Indicate Vital Status

- ☐[0] Deceased **Go to (h)**
- ☐[1] Alive **Go to (d)**

(f) Is this individual willing to be contacted?

- ☐[1] Yes **Go to (i)**
- ☐[0] No **Go to (g)**
- ☐[D] Haven't Contacted/Undecided

(h) To your knowledge, did ____ ever suffer from any of the following conditions? ***[X all that apply]***

- ☐[1] Heart Disease
- ☐[1] Stroke
- ☐[1] Diabetes
- ☐[1] Hypertension
- ☐[1] Cancer
- ☐[1] Alzheimers/Dementia
- ☐[1] Other______________

(d) Have you told this sibling about the Study?

- ☐[1] Yes **Go to (f)**
- ☐[0] No **Go to (e)**

(i) Contact Information

Address: ____

____

Phone: ______________________ (☐ Home ☐ Work)

Best day/time to call: __________________ E-Mail: ______________________

Family Member Telephone Screener Completed? ☐ Yes ☐ No

Individual Eligible to Participate in Study? ☐ Yes ☐ No

Date Scheduled: ___ ___ / ___ ___ ___ / ___ ___ ___ ___

Date Completed: ___ ___ / ___ ___ ___ / ___ ___ ___ ___

LLFS Participant ID #: ___ ___ ___ ___ ___ ___ ___ ___

Participant ID: ______________________________ **Participant Name Code:** ____________

Name of Proband *(Last, First, Middle; if Female, please include Maiden Name):* ______________________________

Section 1 – Proband's Sibling(s) - Continued: (Start from eldest to youngest, indicating Proband's place within this sibship)

Sibling #___

(a) Sibling's Name

Last: ______________________

First: ______________________

Middle: ______________________

Maiden: ______________________

(b) Relationship

☐[1] Full-Brother ☐[3] Half-Brother
☐[2] Full-Sister ☐[4] Half-Sister

(c) Indicate Vital Status

☐[0] Deceased **Go to (h)**
☐[1] Alive **Go to (d)**

(d) Have you told this sibling about the Study?

☐[1] Yes **Go to (f)**
☐[0] No **Go to (e)**

(e) Can you provide a reason why you have not yet contacted this family member about the study? ***[X all that apply]***

☐[1] He/She is cognitively impaired
☐[1] He/she is too ill to participate
☐[1] I am no longer in contact with him/her
☐[1] I do not believe he/she will be interested
☐[1] I do not believe he/she has time
☐[1] He/she lives out of the area
☐[1] I have not had time to contact him/her

(f) Is this individual willing to be contacted?

☐[1] Yes **Go to (i)**
☐[0] No **Go to (g)**
☐[D] Haven't Contacted/Undecided

(g) Can you provide a reason why this family member is not interested in our Study? ***[X all that apply]***

☐[1] He/She is cognitively impaired
☐[1] He/she is too ill to participate
☐[1] I am no longer in contact with him/her
☐[1] He/She is not interested in participating
☐[1] He/she does not have the time
☐[1] He/she lives out of the area
☐[1] I have not had time to contact him/her

(h) To your knowledge, did ____ ever suffer from any of the following conditions? ***[X all that apply]***

☐[1] Heart Disease
☐[1] Stroke
☐[1] Diabetes
☐[1] Hypertension
☐[1] Cancer
☐[1] Alzheimers/Dementia
☐[1] Other______________

(i) Contact Information

Address: ____

____

Phone: ______________________ (☐ Home ☐ Work)

Best day/time to call: ____________________ E-Mail: ______________________

Family Member Telephone Screener Completed? ☐ Yes ☐ No

Individual Eligible to Participate in Study? ☐ Yes ☐ No

Date Scheduled: ___ ___ / ___ ___ ___ / ___ ___ ___ ___

Date Completed: ___ ___ / ___ ___ ___ / ___ ___ ___ ___

LLFS Participant ID #: ___ ___ ___ ___ ___ ___ ___ ___

Participant ID: ________________________________ **Participant Name Code:** ____________

Name of Proband *(Last, First, Middle; if Female, please include Maiden Name):* ____

Section 2 – Proband's Additional Relatives: (Do not document information for relatives beyond the third generation (i.e. grandchildren of Proband/Index Person generation)

Relative #___

(a) Relative's Name

Last: ______________________

First: ______________________

Middle: ______________________

Maiden: ______________________

(d) Can you provide a reason why you have not yet contacted this family member about the study? ***[X all that apply]***

- ☐[1] He/She is cognitively impaired
- ☐[1] He/she is too ill to participate
- ☐[1] I am no longer in contact with him/her
- ☐[1] I do not believe he/she will be interested
- ☐[1] I do not believe he/she has time
- ☐[1] He/she lives out of the area
- ☐[1] I have not had time to contact him/her

(f) Can you provide a reason why this family member is not interested in our Study? ***[X all that apply]***

- ☐[1] He/She is cognitively impaired
- ☐[1] He/she is too ill to participate
- ☐[1] I am no longer in contact with him/her
- ☐[1] He/She is not interested in participating
- ☐[1] He/she does not have the time
- ☐[1] He/she lives out of the area
- ☐[1] I have not had time to contact him/her

(b) Relationship (refer to List in MOP)

____

Related by which Sibling (Index Person)?

____

(d) Have you or another family member told this person about the research Study?

- ☐[1] Yes **Go to (e)**
- ☐[0] No **Go to (d)**

(e) Is this individual willing to be contacted?

- ☐[1] Yes **Go to (g)**
- ☐[0] No **(go to (f)**
- ☐[D] Haven't Contacted/Undecided

Notes:

(g) Contact Information

Address: ____

____

Phone: ______________________ (☐ Home ☐ Work)

Best day/time to call: ______________________

E-Mail: ______________________________

Family Member Telephone Screener Completed? ☐ Yes ☐ No Individual

Eligible to Participate in Study? ☐ Yes ☐ No

Date Scheduled: ___ ___ / ___ ___ ___ / ___ ___ ___ ___

Date Completed: ___ ___ / ___ ___ ___ / ___ ___ ___ ___

LLFS Participant ID #: ___ ___ ___ ___ ___ ___ ___ ___

Participant ID: ______________________________ **Participant Name Code:** ____________

Name of Proband *(Last, First, Middle; if Female, please include Maiden Name):* ______________________________

Section 2 – Proband's Additional Relatives – Continued: (Do not document information for relatives beyond the third generation (i.e. grandchildren of Proband/Index Person generation)

Relative #___

(a) Relative's Name

Last: ______________________

First: ______________________

Middle: ______________________

Maiden: ______________________

(d) Can you provide a reason why you have not yet contacted this family member about the study? ***[X all that apply]***

- ☐1 He/She is cognitively impaired
- ☐1 He/she is too ill to participate
- ☐1 I am no longer in contact with him/her
- ☐1 I do not believe he/she will be interested
- ☐1 I do not believe he/she has time
- ☐1 He/she lives out of the area
- ☐1 I have not had time to contact him/her

(f) Can you provide a reason why this family member is not interested in our Study? ***[X all that apply]***

- ☐1 He/She is cognitively impaired
- ☐1 He/she is too ill to participate
- ☐1 I am no longer in contact with him/her
- ☐1 He/She is not interested in participating
- ☐1 He/she does not have the time
- ☐1 He/she lives out of the area
- ☐1 I have not had time to contact him/her

(b) Relationship (refer to List in MOP)

____

Related by which Sibling (Index Person)?

____

(d) Have you or another family member told this person about the research Study?

- ☐1 Yes **Go to (e)**
- ☐0 No **Go to (d)**

(e) Is this individual willing to be contacted?

- ☐1 Yes **Go to (g)**
- ☐0 No **(go to (f)**
- ☐D Haven't Contacted/Undecided

Notes:

(g) Contact Information

Address: ____

____

Phone: ______________________ (☐ Home ☐ Work)

Best day/time to call: ______________________

E-Mail: ______________________________

Family Member Telephone Screener Completed? ☐ Yes ☐ No Individual

Eligible to Participate in Study? ☐ Yes ☐ No

Date Scheduled: ___ ___ / ___ ___ ___ / ___ ___ ___ ___

Date Completed: ___ ___ / ___ ___ ___ / ___ ___ ___ ___

LLFS Participant ID #: ___ ___ ___ ___ ___ ___ ___ ___

Participant ID: ______________________ **Participant Name Code:** ____________

Name of Proband *(Last, First, Middle; if Female, please include Maiden Name):* ______________________

Section 2 – Proband's Additional Relatives – Continued: (Do not document information for relatives beyond the third generation (i.e. grandchildren of Proband/Index Person generation)

Relative #___

(a) Relative's Name

Last: ____________

First: ____________

Middle: ____________

Maiden: ____________

(d) Can you provide a reason why you have not yet contacted this family member about the study? ***[X all that apply]***

- ☐1 He/She is cognitively impaired
- ☐1 He/she is too ill to participate
- ☐1 I am no longer in contact with him/her
- ☐1 I do not believe he/she will be interested
- ☐1 I do not believe he/she has time
- ☐1 He/she lives out of the area
- ☐1 I have not had time to contact him/her

(f) Can you provide a reason why this family member is not interested in our Study? ***[X all that apply]***

- ☐1 He/She is cognitively impaired
- ☐1 He/she is too ill to participate
- ☐1 I am no longer in contact with him/her
- ☐1 He/She is not interested in participating
- ☐1 He/she does not have the time
- ☐1 He/she lives out of the area
- ☐1 I have not had time to contact him/her

(b) Relationship (refer to List in MOP)

____

Related by which Sibling (Index Person)?

____

(d) Have you or another family member told this person about the research Study?

- ☐1 Yes **Go to (e)**
- ☐0 No **Go to (d)**

(e) Is this individual willing to be contacted?

- ☐1 Yes **Go to (g)**
- ☐0 No **(go to (f)**
- ☐D Haven't Contacted/Undecided

Notes:

(g) Contact Information

Address: ______________________

____

Phone: ____________ (☐ Home ☐ Work)

Best day/time to call: ____________

E-Mail: ____________

Family Member Telephone Screener Completed? ☐ Yes ☐ No Individual

Eligible to Participate in Study? ☐ Yes ☐ No

Date Scheduled: ___ ___ / ___ ___ ___ / ___ ___ ___ ___

Date Completed: ___ ___ / ___ ___ ___ / ___ ___ ___ ___

LLFS Participant ID #: ___ ___ ___ ___ ___ ___ ___ ___

Name of Proband *(Last, First, Middle; if Female, please include Maiden Name):* ____

Section 2 – Proband's Additional Relatives – Continued: (Do not document information for relatives beyond the third generation (i.e. grandchildren of Proband/Index Person generation)

Relative #___

(a) Relative's Name

Last: ____________________________

First: ____________________________

Middle: ____________________________

Maiden: ____________________________

(d) Can you provide a reason why you have not yet contacted this family member about the study? ***[X all that apply]***

- ☐[1] He/She is cognitively impaired
- ☐[1] He/she is too ill to participate
- ☐[1] I am no longer in contact with him/her
- ☐[1] I do not believe he/she will be interested
- ☐[1] I do not believe he/she has time
- ☐[1] He/she lives out of the area
- ☐[1] I have not had time to contact him/her

(f) Can you provide a reason why this family member is not interested in our Study? ***[X all that apply]***

- ☐[1] He/She is cognitively impaired
- ☐[1] He/she is too ill to participate
- ☐[1] I am no longer in contact with him/her
- ☐[1] He/She is not interested in participating
- ☐[1] He/she does not have the time
- ☐[1] He/she lives out of the area
- ☐[1] I have not had time to contact him/her

(b) Relationship (refer to List in MOP)

____

Related by which Sibling (Index Person)?

____

(d) Have you or another family member told this person about the research Study?

- ☐[1] Yes **Go to (e)**
- ☐[0] No **Go to (d)**

(e) Is this individual willing to be contacted?

- ☐[1] Yes **Go to (g)**
- ☐[0] No **(go to (f)**
- ☐[D] Haven't Contacted/Undecided

Notes:

(g) Contact Information

Address: ____

____

Phone: __________________________ (☐ Home ☐ Work)

Best day/time to call: ____________________________

E-Mail: ______________________________________

Family Member Telephone Screener Completed? ☐ Yes ☐ No Individual

Eligible to Participate in Study? ☐ Yes ☐ No

Date Scheduled: ___ ___ / ___ ___ ___ / ___ ___ ___ ___

Date Completed: ___ ___ / ___ ___ ___ / ___ ___ ___ ___

LLFS Participant ID #: ___ ___ ___ ___ ___ ___ ___ ___

Participant ID: ______________________________ **Participant Name Code:** ____________

Name of Proband *(Last, First, Middle; if Female, please include Maiden Name):* ______________________________

Section 2 – Proband's Additional Relatives – Continued: (Do not document information for relatives beyond the third generation (i.e. grandchildren of Proband/Index Person generation)

Relative #___

(a) Relative's Name

Last: ______________________

First: ______________________

Middle: ______________________

Maiden: ______________________

(d) Can you provide a reason why you have not yet contacted this family member about the study? ***[X all that apply]***

- ☐1 He/She is cognitively impaired
- ☐1 He/she is too ill to participate
- ☐1 I am no longer in contact with him/her
- ☐1 I do not believe he/she will be interested
- ☐1 I do not believe he/she has time
- ☐1 He/she lives out of the area
- ☐1 I have not had time to contact him/her

(f) Can you provide a reason why this family member is not interested in our Study? ***[X all that apply]***

- ☐1 He/She is cognitively impaired
- ☐1 He/she is too ill to participate
- ☐1 I am no longer in contact with him/her
- ☐1 He/She is not interested in participating
- ☐1 He/she does not have the time
- ☐1 He/she lives out of the area
- ☐1 I have not had time to contact him/her

(b) Relationship (refer to List in MOP)

____

Related by which Sibling (Index Person)?

____

(d) Have you or another family member told this person about the research Study?

- ☐1 Yes **Go to (e)**
- ☐0 No **Go to (d)**

(e) Is this individual willing to be contacted?

- ☐1 Yes **Go to (g)**
- ☐0 No **(go to (f)**
- ☐D Haven't Contacted/Undecided

Notes:

(g) Contact Information

Address: ______________________________

____

Phone: ______________________ (☐ Home ☐ Work)

Best day/time to call: ______________________

E-Mail: ______________________________

Family Member Telephone Screener Completed? ☐ Yes ☐ No Individual

Eligible to Participate in Study? ☐ Yes ☐ No

Date Scheduled: ___ ___ / ___ ___ ___ / ___ ___ ___ ___

Date Completed: ___ ___ / ___ ___ ___ / ___ ___ ___ ___

LLFS Participant ID #: ___ ___ ___ ___ ___ ___ ___ ___

Participant ID: ___________________________________ **Participant Name Code:** ______________

Name of Proband *(Last, First, Middle; if Female, please include Maiden Name):* ____

Section 2 – Proband's Additional Relatives – Continued: (Do not document information for relatives beyond the third generation (i.e. grandchildren of Proband/Index Person generation)

Relative #___

(a) Relative's Name

Last: ________________________________

First: ________________________________

Middle: ______________________________

Maiden: ______________________________

(d) Can you provide a reason why you have not yet contacted this family member about the study? ***[X all that apply]***

- ☐[1] He/She is cognitively impaired
- ☐[1] He/she is too ill to participate
- ☐[1] I am no longer in contact with him/her
- ☐[1] I do not believe he/she will be interested
- ☐[1] I do not believe he/she has time
- ☐[1] He/she lives out of the area
- ☐[1] I have not had time to contact him/her

(f) Can you provide a reason why this family member is not interested in our Study? ***[X all that apply]***

- ☐[1] He/She is cognitively impaired
- ☐[1] He/she is too ill to participate
- ☐[1] I am no longer in contact with him/her
- ☐[1] He/She is not interested in participating
- ☐[1] He/she does not have the time
- ☐[1] He/she lives out of the area
- ☐[1] I have not had time to contact him/her

(b) Relationship (refer to List in MOP)

____

Related by which Sibling (Index Person)?

____

(d) Have you or another family member told this person about the research Study?

- ☐[1] Yes **Go to (e)**
- ☐[0] No **Go to (d)**

(e) Is this individual willing to be contacted?

- ☐[1] Yes **Go to (g)**
- ☐[0] No **(go to (f)**
- ☐[D] Haven't Contacted/Undecided

Notes:

(g) Contact Information

Address: ____

____

Phone: ___________________________ (☐ Home ☐ Work)

Best day/time to call: ________________________________

E-Mail: ____

Family Member Telephone Screener Completed? ☐ Yes ☐ No Individual

Eligible to Participate in Study? ☐ Yes ☐ No

Date Scheduled: ___ ___ / ___ ___ ___ / ___ ___ ___ ___

Date Completed: ___ ___ / ___ ___ ___ / ___ ___ ___ ___

LLFS Participant ID #: ___ ___ ___ ___ ___ ___ ___ ___

Section 3 – Conclusion of Interview:

(a) ***Interviewer Script*:** *Thank you so much for providing us with this information; we greatly appreciate your time and interest in our study. Our next step will be to contact your siblings and determine their interest in participating. If your family is enrolled in the Study, we will need to obtain additional information about your family structure, including your parents, spouse(s) and children, and would like to call you back. Is this agreeable to you?*

☐1 .... Yes **Go to Script (b)**
☐0 .... No **Go to Script (c)**

***Interviewer Note*: At the discretion of the Field Center, you may proceed to the PIF Form with Proband if it is clear that this family will be enrolled and the Proband is interested in doing so.**

(b) ***Interviewer Script*:** *Is there a preferred day and/or time that you would like us to call back?*

☐1 .... Yes If Yes, which day ____________________________ and/or time _____________________ **End Interview**
☐0 .... No

(c) ***Interviewer Script*:** *Is there someone else in your family that we can contact to provide this information?*

☐1 .... Yes **Proceed with completion of a box on the following page**
☐0 .... No

(d) ***Interviewer Note*:** *If any of Proband's siblings are deceased, the Study would still like to obtain Pedigree information for this Index Person's family (i.e. children and spouse(s)). Therefore, it is important to identify a Family Reporter for this Index Person's branch.*

Is there someone we can contact to obtain information about your [brother/sister]'s _______[Name]_________ family?

☐1 .... Yes **Proceed with completion of a box on the following page**
☐0 .... No

***Interviewer Note*: It is important that you make all attempts to either obtain the information directly from Proband or obtain a designated Reporter. Without the PIF information, the value of the family to the Study will be compromised.**

Reporter Information: **Please complete the information below for the individual who will provide information for the PIF, if not Proband.**

Has the Proband informed this person about the LLFS study and what this will involve? ☐[1] Yes ☐[0] No
Is this individual willing to be contacted by our research group about participation? ☐[1] Yes ☐[0] No

Relationship to Proband: ______________________________

Name: ______________________________

Address: ______________________________

Phone: ______________________ (☐ Home ☐Work) Best day/time to call: ______________________

E-Mail Address: ______________________ LLFS Proband (Family) ID #: ___ ___ ___ ___ ___ ___ ___ ___

Reporter Information: **Please complete the information below for the individual who will provide information for the PIF, if not Proband.**

Has the Proband informed this person about the LLFS study and what this will involve? ☐[1] Yes ☐[0] No
Is this individual willing to be contacted by our research group about participation? ☐[1] Yes ☐[0] No

Relationship to Proband: ______________________________

Name: ______________________________

Address: ______________________________

Phone: ______________________ (☐ Home ☐Work) Best day/time to call: ______________________

E-Mail Address: ______________________ LLFS Proband (Family) ID #: ___ ___ ___ ___ ___ ___ ___ ___

Interviewer*:** ***If you need additional space to record Reporter information, please Xerox this page and attach.

LONG LIFE FAMILY STUDY	**(Affix Label Here)**<br>Participant ID: ____________<br>Participant Name Code: ________	**Date Form Initiated** (e.g., 10JUN2005):<br>☐☐☐☐☐☐☐☐☐<br>d d m m m y y y y<br>**Date Form Completed** (e.g., 10JUN2005):<br>☐☐☐☐☐☐☐☐☐<br>d d m m m y y y y<br>Interviewer Code: ☐☐☐<br>Please Circle Field Center Location:<br>**BU CU DK UP**

LLFS Telephone Screener
Family Member (TS1a)

***Interviewer Note**: To be kept in a confidential file separate from other data forms. Recommended Resources: TS2 and TS3-PIF Form*

This interview entails calling a family member that has been identified and consented by the Proband or another previously screened family member. Before you begin, please take note as to whether the minimum family size has been met for scheduling (refer to the Relative Contact Information Worksheet (TS2)). You will need this information to deliver the correct eligibility script at the end of this interview. Refer to the notes at the End of Interview which will help guide you.

Has the Minimum Family Size been met?

☐[0] ....No **If Screenee is eligible at the end of interview, go to Script (C)**
☐[1] ....Yes **If Screenee is eligible at the end of interview, go to Script (D)**

***Interviewer Script**: Hi, my name is* [insert your name here] *and I am calling from* [insert your institution here] *about the LONG LIFE Family Study. We are attempting to learn why some families have more relatives living to a very old age than some other families.* [Insert family member's name] *told us that they spoke with you and that you are interested in learning more about participating in this family study.* [Insert Proband's name] *has lived a long life. That is why we are asking you to participate with [him/her] in an important international study of longevity. In addition to our university, this study is being conducted at two other American universities* [insert names here], *as well as at the University of Southern Denmark. Our goal is to find out what families with histories of long-lived individuals have in common. By participating in this study with your family members, you may have an opportunity to help improve the health of future generations.*

We have already spoken with [insert Proband's name] *about your family, but if it is okay with you, we have some questions we would like to ask you. Based on the telephone interview, some family members will be invited to participate in a more detailed part of this study. This would involve seeing you in person. You do not have to answer any questions that you do not want to. All information that I receive from you, including your name and any other identifying information, will be strictly confidential and kept in secure files. Your participation is voluntary; you do not have to answer these questions. This will take approximately 5 minutes. Is it okay to speak with you now?*

☐[1] ....Yes
☐[0] ....No

Interviewer: ***If no, then ask:*** *When would be a good day and time for me to call you back and discuss this family study?*

Day/Date: ____ Time: _________________ AM/PM

---

1. Do I have permission to ask you some questions about yourself?

   ☐ 1 ....Yes
   ☐ 0 ....No ***Script:*** ***Thank you very much for speaking with me.***

Interviewer: *Now I would like to verify your contact information:* ***(Pre-fill from TS2 when possible)***

2a. Name: ______________________________ ________. ______________________________
First Name **Middle Initial** **Last Name**

2b. Home Address: ____

____

______________________________________ _____________________ ____ ____ ____ ____ ____
City State Zip Code

2c. What is your home telephone number?

(____ ____ ____) ____ ____ ____ - ____ ____ ____ ____

2d. Is there another number to call that is better during the day? [If no, check here ☐.]

(____ ____ ____) ____ ____ ____ - ____ ____ ____ ____

3. What is your age? ____ ____ ____ Years

4. What is your date of birth?

Day: ____ ____ Month: ____ ____ ____ Year: ____ ____ ____ ____
(Example: June 6, 1904 should appear as 06 / Jun / 1904)

5. What is your gender?

   ☐ 1 ....Male
   ☐ 2 ....Female

***Interviewer*: Be sure to determine the precise biological relationship; use the "other" category, as needed, for "half" relationships.**

6. What is your relationship to ____________________________ *[name of Proband]*?

☐[1] ....Spouse
☐[2] ....Sibling
☐[3] ....Offspring
☐[4] ....Grandchild
☐[5] ....Niece
If Niece, daughter of which Index Person? ____________________________
☐[6] ....Nephew
If Nephew, son of which Index Person? ___________________________
☐[7] ....Other (Please Specify)_________________________

***Interviewer Script*:** *Now I would like to ask you some questions about your general health and any serious medical conditions that you may have.*

7. In general, how would you say your health is? Would you say it is…

☐[5] ....Excellent
☐[4] ....Very Good
☐[3] ....Good
☐[2] ....Fair
☐[1] ....Poor
☐[D] ....Don't Know
☐[R] ....Refused

8. Do you have advanced cancer or a serious medical condition, such as one which requires oxygen or dialysis that would keep you from being able to participate in a home interview or a physical examination?

☐[1] ....Yes, definitely physically unable **End Interview *(Script A below)***
☐[0] ....No, definitely physically able **Continue with Interview**
☐[D] ....Maybe, Call back **Date/Time:** _____________________________

Script A – End Interview*:** *"Thank you so much for this information. We greatly appreciate the time and interest you have shown in our study. The information you provided is very important and will help us discover how some people and their families live to a very old age."* ***END OF INTERVIEW

Interviewer Script – for those continuing: *Before we continue, I want to ask you what is your understanding of the purpose of the LONG LIFE Family Study?*

9a. Key elements (concepts), "family, long lived, research study" ___________________________

____

____

9b. ***Interviewer:*** *Does the individual have a clear understanding of the purpose of the study?*

☐ 1 ....Yes **Go to Q9c**
☐ 0 ....No **End Interview (Use Script B Below)**

9c. ***Interviewer:*** *Does a hearing, language barrier or other problem make the Screenee unable to communicate with you?*

☐ 1 ....Yes **End Interview (Use Script B Below)**
☐ 0 ....No

Script B – End Interview: *"Thank you so much for this information. We greatly appreciate the time and interest you have shown in our study. The information you provided is very important and will help us discover how some people and their families live to a very old age."* ***END OF INTERVIEW***

Note to Interviewer: ***If eligible, refer to page 1 of form and determine if the minimum family size has been met.***

- **If "No", proceed to *Script C***
- **If "Yes", proceed to *Script D***

Script C – Minimum Family Size has* NOT *been met: *Thank you for answering our questions. We still need to gather just a bit more information from your family members to determine whether we can schedule you and your family for the next phase of this study. In that phase, we are interested in studying families over two generations – with at least 2 family members in the oldest generation and one from the offspring generation). If enough people in your family agree to participate, you and your family members will be invited to take part in an in-person visit. This interview can be scheduled at your convenience either in your home or at our clinic. During the visit, a trained clinical staff member will obtain medical and personal information about you. You will be asked to answer questions related to your current and past medical history, medication use, daily living activities, physical activity as well as your health habits. You will also be asked questions such as how many years of education you have had and where you were born and your occupation. Other questionnaires will include paper and pencil tests of your ability to process and recall information and a mood/personality assessment. With your permission, we will obtain measurements of your weight, height, waist circumference, heart rate, blood pressure and lung function. You will also be asked to perform some simple physical tasks such as standing up from of a chair, gripping an object to measure hand strength and walking a short distance to assess your physical function. Additionally, you will be asked to perform a series of movements to test your balance. You will be asked if we can collect a small blood sample. This examination can be completed in approximately 3 hours. We will use all this information to determine the different ways in which families can achieve long lives and successful aging. You may refuse to participate in any portion of the study.*

While we are in the process of contacting your family members already identified by other relatives, would you be willing to ask your [brothers/sisters, aunts/uncles, nieces/nephews or cousins] *if we can contact them to discuss the study and answer any questions they may have? May I ask who you plan to contact? First names only please and their relationship to* [Insert Proband's Name Here]. **Note to Interviewer: Record this information on TS2.** *When I call you back in a week or so regarding your family's eligibility, I will be confirming that you spoke with these family members and they are interested in speaking with us.*

What is a good day and time to call you back to let you know about your family's eligibility?

Day/Date: ____ Time: _________________ AM/PM

Thank you very much. We will be in touch soon to let you know and, if eligible, to schedule your visit. If you have any questions, I can be reached at XXX-XXX-XXXX. ***END OF INTERVIEW.***

Note to Interviewer: ***Once a family has been deemed eligible, contact all family members and continue with questionnaire TS1a, beginning at Q10a.***

Script D – Minimum Family Size Met: *Thank you for answering our questions. The information you provided is very helpful. At this point, we would like to invite you to participate in an in-person visit so that we can gather more information about your health and find out what common traits are present in families with long-lived individuals. This interview can be scheduled at your convenience either in your home or at our clinic. During the visit, a trained clinical staff member will obtain medical and personal information about you. You will be asked to answer questions related to your current and past medical history, medication use, daily living activities, physical activity as well as your health habits. You will also be asked questions such as how many years of education you have had and where you were born and your occupation. Other questionnaires will include paper and pencil tests of your ability to process and recall information and a mood/personality assessment. With your permission, we will obtain measurements of your weight, height, waist circumference, heart rate, blood pressure and lung function. You will also be asked to perform some simple physical tasks such as standing up from of a chair, gripping an object to measure hand strength and walking a short distance to assess your physical function. Additionally, you will be asked to perform a series of movements to test your balance. You will be asked if we can collect a small blood sample. This examination can be completed in approximately 3 hours. We will use all this information to determine the different ways in which families can achieve long lives and successful aging. You may refuse to participate in any portion of the study.*

Complete this Section when Family Eligibility Has Been Met:

10a. Are you interested in participating in this study, including providing a blood sample?

☐ 1 ....Yes **Go to Q10c**
☐ 0 ....No **Go to Q10b**
☐ D ....Don't Know (Pending) Specify: _________________ **Call Back**

10b. Could you please indicate your reason(s)? ***(Please X all that apply; then End Interview using "End Interview Script" below).***

- ☐[1] ....Not Interested
- ☐[1] ....Not Enough Time
- ☐[1] ....Unwilling to Provide Blood Sample
- ☐[1] ....Not Well
- ☐[1] ....Concern about Ability to Complete Examination
- ☐[1] ....Privacy Issue/Concern
- ☐[1] ....Unwilling to Contact Family Members
- ☐[1] ....Lack of "Family" Interest
- ☐[1] ....Other, Please Specify: ______________________________

<u>End Interview Script:</u> *"Thank you so much for the information you have provided. We greatly appreciate the time and interest you have shown in our study. If you decide at a later date that you would like to participate in this family study, please contact me at XXX-XXX-XXXX. Thank you and good-bye."* ***END OF INTERVIEW***

10c.Where would you like this visit to be conducted?

- ☐[1] ....Home Visit **Schedule appointment for in-person visit**
- ☐[2] ....Clinic Visit **Schedule appointment for in-person visit**
- ☐[3] ....Other (Please Specify)_________________________
    - **If applicable, schedule appointment for in-person visit.**
    - **If "other" (i.e. distant visit), then read the following script: "*We will follow-up with you in a few months, as we have yet to begin conducting distant visits."***
    - **If phone visit is the only option, Go to Q10d.**

10d. Do you think you would be interested in participating in the study via a telephone visit?

- ☐[1] ....Yes **Schedule Telephone Visit**
- ☐[0] ....No **Answer Q10e; Read End Interview Script**

10e. **Interviewer:** If no, why not?____

<u>End Interview Script:</u> *"Thank you so much for the information you have provided. We greatly appreciate the time and interest you have shown in our study. If you decide at a later date that you would like to participate in this family study, please contact me at XXX-XXX-XXXX. Thank you and good-bye."* ***END OF INTERVIEW***

<u>For Proband Generation Only</u>:

11a. Do you have a living spouse?

- ☐[1] ....Yes **Go to Q11b**
- ☐[0] ....No **Go to *End Interview Script***

11b. Is *[he/she]* the biological *[father/mother]* of any of your children?

☐[1] ....Yes **Go to Q11c**
☐[0] ....No **Go to *End Interview Script***

11c. What is *[his/her]* age? ____ ____ ____ Years **Go to 11d**

11d. Does *[he/she]* live with you?

☐[1] ....Yes **Go to 11e**
☐[0] ....No **Go to 11e**

Interviewer Only for 11e:
11e. Does spouse live within 2-3 hours of the New York City, Boston, MA or Pittsburgh, PA field centers?

☐[1] ....Yes **Go to Q11f**
☐[0] ....No **Go to Q11f**

11f. Does your spouse have any living siblings over the age of 85?

☐[1] ....Yes **Go to Q11g**
☐[0] ....No **Go to Q11g**

11g. Would you be willing to ask your spouse if we can contact *[him/her]* to discuss participation in the study?

☐[1] ....Yes **Go to TS2, Section 2; Record Contact Information; read *End Interview Script* then proceed to Q13**
☐[0] ....No **Go to *End Interview Script*; Remind of Appointment Details then proceed to Q13**

End Interview Script: *Would you be willing to ask your* [brothers/sisters, aunts/uncles, nieces/nephews or cousins] *if we can contact them to discuss the study and answer any questions they may have? May I ask who you plan to contact? First names only, please, and their relationship to* [Insert Proband's Name Here]. **Note to Interviewer: Record this information on TS2.** *I will call you back in a week or so to confirm that you spoke with these family members and they are interested in speaking with us.*

For Offspring Generation Only:

12a. Do you have a living spouse?

☐[1] ....Yes **Go to Q12b**
☐[0] ....No **Go to *End Interview Script***

12b. What is *[his/her]* age? ____ ____ ____ Years **Go to Q12c**

12c. Does *[he/she]* live with you?

☐[1] ....Yes **Go to Q12d**
☐[0] ....No **Go to Q12d**

Interviewer Only for 12d:
12d. Does spouse live within 2-3 hours of the New York City, Boston, MA or Pittsburgh, PA field centers?

☐[1] ....Yes **Go to Q12e**
☐[0] ....No **Go to Q12e**

12e. Would you be willing to ask your spouse if we can contact *[him/her]* to discuss participation in the study?

☐[1] ....Yes **Go to TS2, Section 2; Record Contact Information; read *End Interview Script* then proceed to Q13**
☐[0] ....No **Go to *End Interview Script*; Remind of Appointment Details then proceed to Q13**

End Interview Script: *"Thank you so much for the information you have provided. We greatly appreciate the time and interest you have shown in our study. If you decide at a later date that you would like to participate in this family study, please contact me at XXX-XXX-XXXX. Thank you and good-bye."* ***END OF INTERVIEW***

Complete This Section for Index Persons Only (Sibs of Proband):

13a. Now we will be needing additional information from you about your family structure, including parents, spouse(s) and children. Would you like to continue with answering these questions at this time?

☐[1] ....Yes **Go to TS3-PIF Form**
☐[0] ....No **Go to Q13b**

13b. Is there a preferred day or time you would like for us to call back?

☐[1] ....Yes If Yes, Day? _____________ Time? _________________ **End Interview and Call Back to Conduct PIF**
☐[0] ....No **Go to Script Below**

Interviewer Script: *"In order to conduct in-person visits on your family, we will need to have you or someone you designate, provide some additional information about your family structure.*

13c. Is there someone else in your family that we can contact to provide this information?

☐[1] ....Yes **Complete Box on Page 8**
☐[0] ....No **End Interview Using Script Below**

End Interview Script: *"Thank you so much for the information you have provided. We greatly appreciate the time and interest you have shown in our study. If you decide at a later date that you would like to participate in this family study, please contact me at XXX-XXX-XXXX. Thank you and good-bye."* ***END OF INTERVIEW***

Interviewer Note: It is important that as the interviewer, you make all attempts to either obtain the information directly from Proband or obtain a designated Reporter. Without the PIF information, the value of the family to the Study will be compromised.

Reporter Information: Please complete the information below for the individual who will provide information for the PIF, if not Index Person.

Have you informed this person about the LLFS study and what this will involve? ☐[1] Yes ☐[0] No
Is this individual willing to be contacted by our research group about participation? ☐[1] Yes ☐[0] No
Relationship to Index Person: ____

Name: ____

Address: ____

Phone: ________________________ (☐ Home ☐Work) Best day/time to call: _________

E-Mail Address: ________________________

LLFS Proband (Family) ID #: ___ ___ ___ ___ ___ ___ ___ ___

LONG LIFE FAMILY STUDY

(Affix Label Here)

Index Person ID: ______________

Index Person Name Code: _______

Date Form Initiated (e.g., 10JUN2005):

d	d	m	m	m	y	y	y	y

Date Form Completed (e.g., 10JUN2005):

d	d	m	m	m	y	y	y	y

Interviewer Code: ☐ ☐ ☐

Please Circle Field Center Location:
BU CU DK UP

LLFS Pedigree Information Form (TS3-PIF)

***Interviewer Notes*: This form is to be completed during a Telephone Interview, to be conducted with an Index Person [hereafter defined as Proband or each of his/her Siblings] or the Index Person's Designated Reporter. The Reporter, identified during the TS2 (pg. 9) or the TS1a, is to be contacted *only* if the Index Person does not feel able or comfortable providing the information.**

The primary purpose of this form is to collect the necessary information to construct and draw a Family Pedigree by identifying a specific set of relatives (parents, spouse(s) and children) for each Index Person in this family. These individual Pedigrees will be subsequently verified with the corresponding Reporter who provided the information, during Phase III of the Study, and combined into one complete Family Pedigree.

Upon completion of Phase I (TS1, TS1a, and TS2), Proband's complete Sibship will have been identified and, if needed, a Reporter for each Index Person identified. Therefore, prior to conducting this Interview, please review the information from the previous calls, generating a PIF for each Index Person, including Proband. Where prior information is available, please verify this information with the Reporter to avoid redundancy.

If additional pages are needed for large families, append additional section pages, as needed, to this Form.

Prior to conducting the interview, please identify the following:

Index Person (Proband or his/her Sibling): ____

Name of Reporter (if not Index Person): ____

If this interview is being done via a Designated Reporter who is not an enrolled participant in LLFS (eg. a Grandchild of Index Person), you may refer to Appendix A on page 7 of this form for to obtain verbal consent.

Index Person ID: ______________________________ **Index Person Name Code:** ______________

Name of Index Person *(Last, First, Middle; if Female, please include Maiden Name):* ____

Section 1 – Index Person's Biological Parents:

	**Parent #1**	**Parent #2**		
**(a)** **Name**	Last: ______________________<br>First: ______________________<br>Middle: ____________________<br>Maiden: ___________________	Last: ______________________<br>First: ______________________<br>Middle: ____________________<br>Maiden: ___________________		
**(b)** **Relationship**	☐1 Father<br>☐2 Mother	☐1 Father<br>☐2 Mother		
**(c) Date of Birth**	__ __ / __ __ __ / __ __ __ __	__ __ / __ __ __ / __ __ __ __		
**(d)** **Indicate Vital Status**	☐0 Deceased **Go to (e)**<br>☐1 Alive **Go to Section 2**	☐0 Deceased **Go to (e)**<br>☐1 Alive **Go to Section 2**		
**(e) Date of Death**	__ __ / __ __ __ / __ __ __ __	__ __ / __ __ __ / __ __ __ __		
**(f)** **Did ____ ever suffer from any of the following conditions?** ***[X all that apply]***	☐1 Heart Disease<br>☐2 Stroke<br>☐3 Diabetes<br>☐4 Hypertension<br>☐5 Cancer<br>☐6 Alzheimers/Dementia<br>☐7 Other________________	☐1 Heart Disease<br>☐2 Stroke<br>☐3 Diabetes<br>☐4 Hypertension<br>☐5 Cancer<br>☐6 Alzheimers/Dementia<br>☐7 Other________________		

Notes:

Index Person ID: ______________________________ **Index Person Name Code:** ______________

Name of Index Person *(Last, First, Middle; if Female, please include Maiden Name):* ____

Section 2 – Index Person's Spouse(s):

	Spouse/Partner #1	Spouse/Partner #2	Spouse/Partner #3	Spouse/Partner #4
**(a)** **Spouse's Name**	Last: ______________________<br>First: ______________________<br>Middle: ____________________<br>Maiden: ___________________	Last: ______________________<br>First: ______________________<br>Middle: ____________________<br>Maiden: ___________________	Last: ___________________<br>First: ___________________<br>Middle: _________________<br>Maiden: _________________	Last: _____________________<br>First: _____________________<br>Middle: ___________________<br>Maiden: __________________
**(b)** **Relationship**	☐ 1 Husband (Male Partner)<br>☐ 2 Wife (Female Partner)	☐ 1 Husband (Male Partner)<br>☐ 2 Wife (Female Partner)	☐ 1 Husband (Male Partner)<br>☐ 2 Wife (Female Partner)	☐ 1 Husband (Male Partner)<br>☐ 2 Wife (Female Partner)
**(c) Date of Birth**	__ __ / __ __ __ / __ __ __ __	__ __ / __ __ __ / __ __ __ __	__ __ / __ __ __ / __ __ __ __	__ __ / __ __ __ / __ __ __ __
**(d)** **Indicate Vital Status**	☐ 0 Deceased **Go to (e)**<br>☐ 1 Alive **Go to (g)**	☐ 0 Deceased **Go to (e)**<br>☐ 1 Alive **Go to (g)**	☐ 0 Deceased **Go to (e)**<br>☐ 1 Alive **Go to (g)**	☐ 0 Deceased **Go to (e)**<br>☐ 1 Alive **Go to (g)**
**(e) Date of Death**	__ __ / __ __ __ / __ __ __ __	__ __ / __ __ __ / __ __ __ __	__ __ / __ __ __ / __ __ __ __	__ __ / __ __ __ / __ __ __ __
**(f)** **Did ____ ever suffer from any of the following conditions?** ***[X all that apply]***	☐ 1 Heart Disease<br>☐ 2 Stroke<br>☐ 3 Diabetes<br>☐ 4 Hypertension<br>☐ 5 Cancer<br>☐ 6 Alzheimers/Dementia<br>☐ 7 Other________________	☐ 1 Heart Disease<br>☐ 2 Stroke<br>☐ 3 Diabetes<br>☐ 4 Hypertension<br>☐ 5 Cancer<br>☐ 6 Alzheimers/Dementia<br>☐ 7 Other________________	☐ 1 Heart Disease<br>☐ 2 Stroke<br>☐ 3 Diabetes<br>☐ 4 Hypertension<br>☐ 5 Cancer<br>☐ 6 Alzheimers/Dementia<br>☐ 7 Other______________	☐ 1 Heart Disease<br>☐ 2 Stroke<br>☐ 3 Diabetes<br>☐ 4 Hypertension<br>☐ 5 Cancer<br>☐ 6 Alzheimers/Dementia<br>☐ 7 Other________________
**(g)** **Did you and ____ ever have any children?**	☐ 1 Yes **Go to Section 3**<br>☐ 0 No	☐ 1 Yes **Go to Section 3**<br>☐ 0 No	☐ 1 Yes **Go to Section 3**<br>☐ 0 No	☐ 1 Yes **Go to Section 3**<br>☐ 0 No

Notes:

Index Person ID: ____________________________ **Index Person Name Code:** _____________

Name of Index Person *(Last, First, Middle; if Female, please include Maiden Name):* ____

Section 3 – Index Person's Biological Children (Start from eldest to youngest; complete for each Spouse noted in Section 2 above):

Children of Spouse/Partner #____ ; Name *(Last, First, Middle; if Female, please include Maiden Name):* __________________________________

	**Child #___**	**Child #___**	**Child #___**	**Child #___**
**(a) Child's Name**	Last: ____________<br>First: ____________<br>Middle: ____________<br>Maiden: ____________	Last: ____________<br>First: ____________<br>Middle: ____________<br>Maiden: ____________	Last: ____________<br>First: ____________<br>Middle: ____________<br>Maiden: ____________	Last: ____________<br>First: ____________<br>Middle: ____________<br>Maiden: ____________
**(b) Relationship**	☐[1] Son<br>☐[2] Daughter	☐[1] Son<br>☐[2] Daughter	☐[1] Son<br>☐[2] Daughter	☐[1] Son<br>☐[2] Daughter
**(c) Date of Birth**	__ __ / __ __ __ / __ __ __ __	__ __ / __ __ __ / __ __ __ __	__ __ / __ __ __ / __ __ __ __	__ __ / __ __ __ / __ __ __ __
**(d) Indicate Vital Status**	☐[0] Deceased **Go to (e)**<br>☐[1] Alive **Go to (g)**	☐[0] Deceased **Go to (e)**<br>☐[1] Alive **Go to (g)**	☐[0] Deceased **Go to (e)**<br>☐[1] Alive **Go to (g)**	☐[0] Deceased **Go to (e)**<br>☐[1] Alive **Go to (g)**
**(e) Date of Death**	__ __ / __ __ __ / __ __ __ __	__ __ / __ __ __ / __ __ __ __	__ __ / __ __ __ / __ __ __ __	__ __ / __ __ __ / __ __ __ __
**(f) Did ____ ever suffer from any of the following conditions? *[X all that apply]***	☐[1] Heart Disease<br>☐[2] Stroke<br>☐[3] Diabetes<br>☐[4] Hypertension<br>☐[5] Cancer<br>☐[6] Alzheimers/Dementia<br>☐[7] Other__________	☐[1] Heart Disease<br>☐[2] Stroke<br>☐[3] Diabetes<br>☐[4] Hypertension<br>☐[5] Cancer<br>☐[6] Alzheimers/Dementia<br>☐[7] Other__________	☐[1] Heart Disease<br>☐[2] Stroke<br>☐[3] Diabetes<br>☐[4] Hypertension<br>☐[5] Cancer<br>☐[6] Alzheimers/Dementia<br>☐[7] Other__________	☐[1] Heart Disease<br>☐[2] Stroke<br>☐[3] Diabetes<br>☐[4] Hypertension<br>☐[5] Cancer<br>☐[6] Alzheimers/Dementia<br>☐[7] Other__________
**(g) # of Biological Children?**	_____ Biological Children	_____ Biological Children	_____ Biological Children	_____ Biological Children

**(h) Did you have another spouse?**	☐[1] Yes **Return to Section 2**<br>☐[0] No **Go to (i)**	**(i) Are there any other Partners with whom you have biological children?**	☐[1] Yes **Return to Section 2**<br>☐[0] No **End**

Index Person's Children – Continued from Page 5

Section 3 – Index Person's Biological Children (Start from eldest to youngest; complete for each Spouse noted in Section 2 above):

Children of Spouse/Partner #____ ; Name *(Last, First, Middle; if Female, please include Maiden Name):* ___________________________________

	**Child #___**	**Child #___**	**Child #___**	**Child #___**
**(a) Child's Name**	Last: ______<br>First: ______<br>Middle: ______<br>Maiden: ______	Last: ______<br>First: ______<br>Middle: ______<br>Maiden: ______	Last: ______<br>First: ______<br>Middle: ______<br>Maiden: ______	Last: ______<br>First: ______<br>Middle: ______<br>Maiden: ______
**(b) Relationship**	☐1 Son<br>☐2 Daughter	☐1 Son<br>☐2 Daughter	☐1 Son<br>☐2 Daughter	☐1 Son<br>☐2 Daughter
**(c) Date of Birth**	__ __ / __ __ __ / __ __ __ __	__ __ / __ __ __ / __ __ __ __	__ __ / __ __ __ / __ __ __ __	__ __ / __ __ __ / __ __ __ __
**(d) Indicate Vital Status**	☐0 Deceased **Go to (e)**<br>☐1 Alive **Go to (g)**	☐0 Deceased **Go to (e)**<br>☐1 Alive **Go to (g)**	☐0 Deceased **Go to (e)**<br>☐1 Alive **Go to (g)**	☐0 Deceased **Go to (e)**<br>☐1 Alive **Go to (g)**
**(e) Date of Death**	__ __ / __ __ __ / __ __ __ __	__ __ / __ __ __ / __ __ __ __	__ __ / __ __ __ / __ __ __ __	__ __ / __ __ __ / __ __ __ __
**(f) Did ____ ever suffer from any of the following conditions? *[X all that apply]***	☐1 Heart Disease<br>☐2 Stroke<br>☐3 Diabetes<br>☐4 Hypertension<br>☐5 Cancer<br>☐6 Alzheimers/Dementia<br>☐7 Other______	☐1 Heart Disease<br>☐2 Stroke<br>☐3 Diabetes<br>☐4 Hypertension<br>☐5 Cancer<br>☐6 Alzheimers/Dementia<br>☐7 Other______	☐1 Heart Disease<br>☐2 Stroke<br>☐3 Diabetes<br>☐4 Hypertension<br>☐5 Cancer<br>☐6 Alzheimers/Dementia<br>☐7 Other______	☐1 Heart Disease<br>☐2 Stroke<br>☐3 Diabetes<br>☐4 Hypertension<br>☐5 Cancer<br>☐6 Alzheimers/Dementia<br>☐7 Other______
**(g) # of Biological Children?**	______ Biological Children	______ Biological Children	______ Biological Children	______ Biological Children
**(h) Did you have another spouse?**	☐1 Yes **Return to Section 2**<br>☐0 No **Go to (i)**	**(i) Are there any other Partners with whom you have biological children?**	☐1 Yes **Return to Section 2**<br>☐0 No **End**	

Index Person's Children – Continued from Page 6

Section 3 – Index Person's Biological Children (Start from eldest to youngest; complete for each Spouse noted in Section 2 above):

Children of Spouse/Partner #____ ; Name *(Last, First, Middle; if Female, please include Maiden Name):* ____________________________________

	**Child #___**	**Child #___**	**Child #___**	**Child #___**
**(a) Child's Name**	Last: ____________________<br>First: ____________________<br>Middle: __________________<br>Maiden: _________________	Last: ____________________<br>First: ____________________<br>Middle: __________________<br>Maiden: _________________	Last: ____________________<br>First: ____________________<br>Middle: __________________<br>Maiden: _________________	Last: ____________________<br>First: ____________________<br>Middle: __________________<br>Maiden: _________________
**(b) Relationship**	☐1 Son<br>☐2 Daughter	☐1 Son<br>☐2 Daughter	☐1 Son<br>☐2 Daughter	☐1 Son<br>☐2 Daughter
**(c) Date of Birth**	__ __ / __ __ __ / __ __ __ __	__ __ / __ __ __ / __ __ __ __	__ __ / __ __ __ / __ __ __ __	__ __ / __ __ __ / __ __ __ __
**(d) Indicate Vital Status**	☐0 Deceased **Go to (e)**<br>☐1 Alive **Go to (g)**	☐0 Deceased **Go to (e)**<br>☐1 Alive **Go to (g)**	☐0 Deceased **Go to (e)**<br>☐1 Alive **Go to (g)**	☐0 Deceased **Go to (e)**<br>☐1 Alive **Go to (g)**
**(e) Date of Death**	__ __ / __ __ __ / __ __ __ __	__ __ / __ __ __ / __ __ __ __	__ __ / __ __ __ / __ __ __ __	__ __ / __ __ __ / __ __ __ __
**(f) Did ____ ever suffer from any of the following conditions? *[X all that apply]***	☐1 Heart Disease<br>☐2 Stroke<br>☐3 Diabetes<br>☐4 Hypertension<br>☐5 Cancer<br>☐6 Alzheimers/Dementia<br>☐7 Other_______________	☐1 Heart Disease<br>☐2 Stroke<br>☐3 Diabetes<br>☐4 Hypertension<br>☐5 Cancer<br>☐6 Alzheimers/Dementia<br>☐7 Other_______________	☐1 Heart Disease<br>☐2 Stroke<br>☐3 Diabetes<br>☐4 Hypertension<br>☐5 Cancer<br>☐6 Alzheimers/Dementia<br>☐7 Other_______________	☐1 Heart Disease<br>☐2 Stroke<br>☐3 Diabetes<br>☐4 Hypertension<br>☐5 Cancer<br>☐6 Alzheimers/Dementia<br>☐7 Other_______________
**(g) # of Biological Children?**	______ Biological Children	______ Biological Children	______ Biological Children	______ Biological Children

**(h) Did you have another spouse?**	☐1 Yes **Return to Section 2**<br>☐0 No **Go to (i)**	**(i) Are there any other Partners with whom you have biological children?**	☐1 Yes **Return to Section 2**<br>☐0 No **End**

APPENDIX A
Pedigree Information Form

Following is a Script with which to obtain verbal consent to ask the following information for individuals who are participating in the role of a Designated Reporter and who will not be/are not enrolled as study participants:

Hello, my name is ________________ and I am calling from [insert name of institution] about the LONG LIFE Family Study. We are attempting to learn why some families have more relatives living to a very old age than some other families. Your [insert relative's relation and name] told us they spoke with you, and that you are willing to provide us with additional information about your family structure, including your parents, spouse(s) and children.

We are requesting this information as part of this study because it may possibly help us to identify the common traits present in some families that have helped these individuals live to a very old age.

I'd first like to provide you with some information about the background and purpose of this study. Your [insert reference to relative] was chosen at random from a list of older Medicare users. The Centers for Medicare and Medicaid Services is cooperating with the National Institutes of Health (NIH) on this study. Two other American Universities [insert collaborating university names], along with the University of Southern Denmark, are working together with us to possibly learn more about the secrets to a long life.

By sharing some information about your relatives with us, we will be able to construct a family tree of your family. This information may help efforts to improve the health of future generations, including many of your own younger family members.

With your permission, I would like to begin this interview by asking you to provide some brief information about [insert reference to relative] parents, spouse(s), and children. You do not have to answer any questions that you do not feel comfortable answering. All of the information you provide, including your name and other identifying information, will be kept strictly confidential and maintained in secure closets in our research department. Your participation is completely voluntary; you do not have to answer these questions.

Do I have your permission to ask you some questions about the family members I mentioned above?

☐ 1 ....Yes **Begin Interview**
☐ 0 ....No ***Thank you for speaking with me*.**

[If No:] Try to obtain the name of an alternative reporter; if unsuccessful, try to go back to the proband to obtain this information.

LONG LIFE FAMILY STUDY	**(Affix Label Here)**<br><br>Participant ID: ____________<br><br>Participant Name Code: __________	**Date Form Initiated** (e.g., 10JUN2005):<br>☐☐☐☐☐☐☐☐☐<br>d d m m m y y y y<br>**Date Form Completed** (e.g., 10JUN2005):<br>☐☐☐☐☐☐☐☐☐<br>d d m m m y y y y<br>Interviewer Code: ☐☐☐<br>Please Circle Field Center Location:<br>**BU CU DK UP**

LLFS Telephone Screener
Control Subjects (TS1a-c)

Interviewer Note: ***To be kept in a confidential file separate from other data forms***

This interview entails calling a spouse of an enrolled family member that has been identified and consented by the previously screened family member.

Interviewer Script: *Hi, my name is* [insert your name here] *and I am calling from* [insert your institution here] *about the LONG LIFE Family Study. We are attempting to learn why some families have more relatives living to a very old age than some other families. Your* [husband / wife] [Insert spouse's name] *told us that they spoke with you and that you are interested in learning more about participating in this study. Your* [husband / wife] *belongs to a family we believe has had the good fortune to have many member living long and healthy lives. Because your* [husband / wife] *is participating in this important international study of longevity, we would like to invite you to participate as well. In addition to our university, this study is being conducted at two other American universities* [insert names here], *as well as at the University of Southern Denmark. Our goal is to find out what families with histories of long-lived individuals have in common. By participating in this study, you may make an important contribution to our efforts to help improve the health of future generations.*

If you choose to participate in our study, we would arrange to see you in person. For now, we have some questions we would like to ask you. You do not have to answer any questions that you do not want to. All information that I receive from you, including your name and any other identifying information, will be strictly confidential and kept under lock and key. Your participation is voluntary; you do not have to answer these questions. This will take approximately 15 minutes. Is it okay to speak with you now?

☐ 1 ....Yes
☐ 0 ....No

Interviewer: ***If no, then ask:*** *When would be a good day and time for me to call you back and discuss this study?*

Day/Date: ____ Time: __________________ AM/PM

1. Do I have permission to ask you some questions about yourself?

☐ 1 ....Yes
☐ 0 ....No   ***Script*:** ***Thank you very much for speaking with me.***

***Interviewer*:** *Now I would like to verify your contact information:*

2a. Name: ______________________________ ________. ______________________________
First Name **Middle Initial** **Last Name**

2b. Home Address: ____

____

________________________ ________________ ____ ____ ____ ____ ____
City State Zip Code

2c. Home telephone number?

(____ ____ ____) ____ ____ ____ - ____ ____ ____ ____

2d. Is there another number to call that is better during the day? [If no, check here ☐.]

(____ ____ ____) ____ ____ ____ - ____ ____ ____ ____

3. Age? ____ ____ ____ Years

4. Date of birth?

Day: ____ ____ Month: ____ ____ ____ Year: ____ ____ ____ ____
(Example: June 6, 1904 should appear as 06 / Jun / 1904)

5. Gender?

☐ 1 ....Male
☐ 2 ....Female

***Interviewer Script*:** *Now I would like to ask you some questions about your general health and any serious medical conditions that you may have.*

6. In general, how would you say your health is? Would you say it is…

- ☐ 5 ....Excellent
- ☐ 4 ....Very Good
- ☐ 3 ....Good
- ☐ 2 ....Fair
- ☐ 1 ....Poor
- ☐ D ....Don't Know
- ☐ R ....Refused

7. Do you have advanced cancer or a serious medical condition, such as one which requires oxygen or dialysis that would keep you from being able to participate in a home interview or a physical examination?

- ☐ 1 ....Yes, definitely physically unable **End Interview (Script A below)**
- ☐ 0 ....No, definitely physically able **Continue with Interview**
- ☐ D ....Maybe, Call back **Date/Time:** ________________________

Script A – End Interview: *"Thank you so much for this information. We greatly appreciate the time and interest you have shown in our study. The information you provided is very important. Take care."* ***END OF INTERVIEW***

Interviewer Script – for those continuing: *Before we continue, I want to ask you what is your understanding of the purpose of the LONG LIFE Family Study?*

8a. Key elements (concepts), "family, long lived, research study" ________________________

____

____

Interviewer: ***Does the individual have a clear understanding of the purpose of the study?***

8b.
- ☐ 1 ....Yes
- ☐ 0 ....No **End Interview (Use Script B Below)**

Interviewer: ***Does a hearing, language barrier or other problem make the Screenee unable to communicate with you?***

8c.
- ☐ 1 ....Yes **End Interview (Use Script B Below)**
- ☐ 0 ....No

Script B – End Interview: *"Thank you so much for this information. We greatly appreciate the time and interest you have shown in our study. The information you provided is very important. Take care."* ***END OF INTERVIEW***

For Controls Residing within the 3 U.S. Catchment Areas: Proceed with Q9a-9c below.

Interviewer: *Thank you for answering our questions. The information you provided is very helpful. At this point, we would like to invite you to participate in an in-person visit with your husband / wife so that we can gather more information about your health. This interview can be scheduled at your convenience either in your home or at our clinic. We can conduct this visit at the same time as we see your spouse. During the visit, a trained clinical staff member will obtain medical and personal information about you. You will be asked to answer questions related to your current and past medical history, medication use, daily living activities, physical activity as well as your health habits. You will also be asked questions such as how many years of education you have had and where you were born and your occupation. Other questionnaires will include paper and pencil tests of your ability to process and recall information and a mood/personality assessment. With your permission, we will obtain measurements of your weight, height, waist circumference, heart rate, blood pressure and lung function. You will also be asked to perform some simple physical tasks such as standing up from of a chair, gripping an object to measure hand strength and walking a short distance to assess your physical function. Additionally, you will be asked to perform a series of movements to test your balance. You will be asked if we can collect a small blood sample. This examination can be completed in approximately 3 hours. We will use all this information to determine the different ways in which families can achieve long lives and successful aging. You may refuse to participate in any portion of the study.*

9a. Are you interested in participating in an in-person visit, including providing a blood sample?

☐ 1 ....Yes **Go to 9b**
☐ 0 ....No **Go to Q9c**
☐ D ....Don't Know (Pending) Specify: ________ **Call Back**

9b. Where would you like this visit to be conducted?

☐ 1 ....Home Visit **Schedule appointment for in-person visit**
☐ 2 ....Clinic Visit **Schedule appointment for in-person visit**

9c. If "No", could you please indicate your reason(s)? ***(Please X all that apply; then End Interview using Script B below).***

☐ 1 ....Just Not Interested
☐ 1 ....Not Enough Time
☐ 1 ....Unwilling to Provide Blood Sample
☐ 1 ....Not Well
☐ 1 ....Concern about Ability to Complete Examination
☐ 1 ....Privacy Issue/Concern
☐ 1 ....Unwilling to Contact Family Members
☐ 1 ....Lack of "Family" Interest
☐ 1 ....Other, Please Specify: ______________________________

Script B – End Interview: *"Thank you so much for this information. We greatly appreciate the time and interest you have shown in our study. The information you provided is very important and will help us discover how some people and their families live to a very old age. If you decide at a later date that you would like to participate in this family study, please contact me at XXX-XXX-XXXX. Thank you and good-bye."* ***END OF INTERVIEW***

For Controls Residing Outside of the 3 U.S. Catchment Areas: Proceed with Q10a-10b below.

Interviewer: *Thank you for answering our questions. The information you provided is very helpful. At this time, we do not have plans to conduct in person visits in your area. However, if we do visit your area at a later time, we would like to invite you to participate in an in-person visit with your husband/wife so that we can gather more information about your health. This interview can be scheduled at your convenience. We can conduct this visit at the same time as we see your spouse. During the visit, a trained clinical staff member will obtain medical and personal information about you. You will be asked to answer questions related to your current and past medical history, medication use, daily living activities, physical activity as well as your health habits. You will also be asked questions such as how many years of education you have had and where you were born and your occupation. Other questionnaires will include paper and pencil tests of your ability to process and recall information and a mood/personality assessment. With your permission, we will obtain measurements of your weight, height, waist circumference, heart rate, blood pressure and lung function. You will also be asked to perform some simple physical tasks such as standing up from of a chair, gripping an object to measure hand strength and walking a short distance to assess your physical function. Additionally, you will be asked to perform a series of movements to test your balance. You will be asked if we can collect a small blood sample. This examination can be completed in approximately 3 hours. We will use all this information to determine the different ways in which families can achieve long lives and successful aging. You may refuse to participate in any portion of the study.*

10a. Are you interested in participating in an in-person visit, including providing a blood sample, if we come to your area?

☐ 1 ....Yes **Go to *End Interview Script A Below***
☐ 0 ....No **Go to Q10b**
☐ D ....Don't Know (Pending) Specify: ________ **Call Back**

Script A – End Interview: *"Thank you so much for this information. We greatly appreciate the time and interest you have shown in our study. We will be in touch with you regarding participation if we are able to schedule a visit in your area. The information you provided is very important. Thank you and good-bye."* ***END OF INTERVIEW***

10b. If "No", could you please indicate your reason(s)? ***(Please X all that apply; then End Interview using Script B below).***

- ☐[1] ....Just Not Interested
- ☐[1] ....Not Enough Time
- ☐[1] ....Unwilling to Provide Blood Sample
- ☐[1] ....Not Well
- ☐[1] ....Concern about Ability to Complete Examination
- ☐[1] ....Privacy Issue/Concern
- ☐[1] ....Unwilling to Contact Family Members
- ☐[1] ....Lack of "Family" Interest
- ☐[1] ....Other, Please Specify: ______________________________

Script B – End Interview: *"Thank you so much for this information. We greatly appreciate the time and interest you have shown in our study. We will be in touch with you regarding participation if we are able to schedule a visit to your area. The information you provided is very important."* ***END OF INTERVIEW***

LONG LIFE FAMILY STUDY

(Affix Label Here)

Participant ID: ____________________

Participant Name Code: _____________

Date Form Initiated (e.g., 10JUN2005):

☐ ☐ ☐ ☐ ☐ ☐ ☐ ☐ ☐
d d m m m y y y y

Date Form Completed (e.g., 10JUN2005):

☐ ☐ ☐ ☐ ☐ ☐ ☐ ☐ ☐
d d m m m y y y y

Interviewer Code: ☐ ☐ ☐

Please Circle Field Center Location:
BU CU DK UP

LLFS Telephone Screener
Control Subjects (TS1a-c)

Interviewer Note: ***To be kept in a confidential file separate from other data forms***

This interview entails calling a spouse of an enrolled family member that has been identified and consented by the previously screened family member.

Interviewer Script: *Hi, my name is* [insert your name here] *and I am calling from* [insert your institution here] *about the LONG LIFE Family Study. We are attempting to learn why some families have more relatives living to a very old age than some other families. Your* [husband / wife] [Insert spouse's name] *told us that they spoke with you and that you are interested in learning more about participating in this study. Your* [husband / wife] *belongs to a family we believe has had the good fortune to have many member living long and healthy lives. Because your* [husband / wife] *is participating in this important international study of longevity, we would like to invite you to participate as well. In addition to our university, this study is being conducted at two other American universities* [insert names here], *as well as at the University of Southern Denmark. Our goal is to find out what families with histories of long-lived individuals have in common. By participating in this study, you may make an important contribution to our efforts to help improve the health of future generations.*

If you choose to participate in our study, we would arrange to see you in person. For now, we have some questions we would like to ask you. You do not have to answer any questions that you do not want to. All information that I receive from you, including your name and any other identifying information, will be strictly confidential and kept under lock and key. Your participation is voluntary; you do not have to answer these questions. This will take approximately 15 minutes. Is it okay to speak with you now?

☐ 1 ....Yes
☐ 0 ....No

Interviewer: ***If no, then ask:*** *When would be a good day and time for me to call you back and discuss this study?*

Day/Date: ____ Time: __________________ AM/PM

1. Do I have permission to ask you some questions about yourself?

☐ 1 ....Yes
☐ 0 ....No ***Script:*** ***Thank you very much for speaking with me.***

Interviewer: *Now I would like to verify your contact information:*

2a. Name: ______________________________ ________. ______________________________
First Name **Middle Initial** **Last Name**

2b. Home Address: ____

____

__________________________________ ____________________ ____ ____ ____ ____ ____
City State Zip Code

2c. Home telephone number?

(____ ____ ____) ____ ____ ____ - ____ ____ ____ ____

2d. Is there another number to call that is better during the day? [If no, check here ☐.]

(____ ____ ____) ____ ____ ____ - ____ ____ ____ ____

3. Age? ____ ____ ____ Years

4. Date of birth?

Day: ____ ____ Month: ____ ____ ____ Year: ____ ____ ____ ____
(Example: June 6, 1904 should appear as 06 / Jun / 1904)

5. Gender?

☐ 1 ....Male
☐ 2 ....Female

Interviewer Script: *Now I would like to ask you some questions about your general health and any serious medical conditions that you may have.*

6. In general, how would you say your health is? Would you say it is…

☐ 5 ....Excellent
☐ 4 ....Very Good
☐ 3 ....Good
☐ 2 ....Fair
☐ 1 ....Poor
☐ D ....Don't Know
☐ R ....Refused

7. Do you have advanced cancer or a serious medical condition, such as one which requires oxygen or dialysis that would keep you from being able to participate in a home interview or a physical examination?

☐ 1 ....Yes, definitely physically unable **End Interview (Script A below)**
☐ 0 ....No, definitely physically able **Continue with Interview**
☐ D ....Maybe, Call back **Date/Time:** ________________________

<u>Script A – End Interview</u>: *"Thank you so much for this information. We greatly appreciate the time and interest you have shown in our study. The information you provided is very important. Take care."* ***END OF INTERVIEW***

<u>Interviewer Script – for those continuing</u>: *Before we continue, I want to ask you what is your understanding of the purpose of the LONG LIFE Family Study?*

8a. Key elements (concepts), "family, long lived, research study" ________________________

____

____

***<u>Interviewer</u>:* Does the individual have a clear understanding of the purpose of the study?**

8b. ☐ 1 ....Yes
☐ 0 ....No **End Interview (Use Script B Below)**

***<u>Interviewer</u>:* Does a hearing, language barrier or other problem make the Screenee unable to communicate with you?**

8c. ☐ 1 ....Yes **End Interview (Use Script B Below)**
☐ 0 ....No

<u>Script B – End Interview</u>: *"Thank you so much for this information. We greatly appreciate the time and interest you have shown in our study. The information you provided is very important. Take care."* ***END OF INTERVIEW***

For Controls Residing within the 3 U.S. Catchment Areas: Proceed with Q9a-9c below.

Interviewer: *Thank you for answering our questions. The information you provided is very helpful. At this point, we would like to invite you to participate in an in-person visit with your husband / wife so that we can gather more information about your health. This interview can be scheduled at your convenience either in your home or at our clinic. We can conduct this visit at the same time as we see your spouse. During the visit, a trained clinical staff member will obtain medical and personal information about you. You will be asked to answer questions related to your current and past medical history, medication use, daily living activities, physical activity as well as your health habits. You will also be asked questions such as how many years of education you have had and where you were born and your occupation. Other questionnaires will include paper and pencil tests of your ability to process and recall information and a mood/personality assessment. With your permission, we will obtain measurements of your weight, height, waist circumference, heart rate, blood pressure and lung function. You will also be asked to perform some simple physical tasks such as standing up from of a chair, gripping an object to measure hand strength and walking a short distance to assess your physical function. Additionally, you will be asked to perform a series of movements to test your balance. You will be asked if we can collect a small blood sample. This examination can be completed in approximately 3 hours. We will use all this information to determine the different ways in which families can achieve long lives and successful aging. You may refuse to participate in any portion of the study.*

9a. Are you interested in participating in an in-person visit, including providing a blood sample?

☐ 1 ....Yes **Go to 9b**
☐ 0 ....No **Go to Q9c**
☐ D ....Don't Know (Pending) Specify: ________ **Call Back**

9b. Where would you like this visit to be conducted?

☐ 1 ....Home Visit **Schedule appointment for in-person visit**
☐ 2 ....Clinic Visit **Schedule appointment for in-person visit**

9c. If "No", could you please indicate your reason(s)? ***(Please X all that apply; then End Interview using Script B below).***

☐ 1 ....Just Not Interested
☐ 1 ....Not Enough Time
☐ 1 ....Unwilling to Provide Blood Sample
☐ 1 ....Not Well
☐ 1 ....Concern about Ability to Complete Examination
☐ 1 ....Privacy Issue/Concern
☐ 1 ....Unwilling to Contact Family Members
☐ 1 ....Lack of "Family" Interest
☐ 1 ....Other, Please Specify: ______________________________

Script B – End Interview: *"Thank you so much for this information. We greatly appreciate the time and interest you have shown in our study. The information you provided is very important and will help us discover how some people and their families live to a very old age. If you decide at a later date that you would like to participate in this family study, please contact me at XXX-XXX-XXXX. Thank you and good-bye."* ***END OF INTERVIEW***

For Controls Residing Outside of the 3 U.S. Catchment Areas: Proceed with Q10a-10b below.

Interviewer: *Thank you for answering our questions. The information you provided is very helpful. At this time, we do not have plans to conduct in person visits in your area. However, if we do visit your area at a later time, we would like to invite you to participate in an in-person visit with your husband/wife so that we can gather more information about your health. This interview can be scheduled at your convenience. We can conduct this visit at the same time as we see your spouse. During the visit, a trained clinical staff member will obtain medical and personal information about you. You will be asked to answer questions related to your current and past medical history, medication use, daily living activities, physical activity as well as your health habits. You will also be asked questions such as how many years of education you have had and where you were born and your occupation. Other questionnaires will include paper and pencil tests of your ability to process and recall information and a mood/personality assessment. With your permission, we will obtain measurements of your weight, height, waist circumference, heart rate, blood pressure and lung function. You will also be asked to perform some simple physical tasks such as standing up from of a chair, gripping an object to measure hand strength and walking a short distance to assess your physical function. Additionally, you will be asked to perform a series of movements to test your balance. You will be asked if we can collect a small blood sample. This examination can be completed in approximately 3 hours. We will use all this information to determine the different ways in which families can achieve long lives and successful aging. You may refuse to participate in any portion of the study.*

10a. Are you interested in participating in an in-person visit, including providing a blood sample, if we come to your area?

☐[1] ....Yes **Go to *End Interview Script A Below***
☐[0] ....No **Go to Q10b**
☐[D] ....Don't Know (Pending) Specify: ________ **Call Back**

Script A – End Interview: *"Thank you so much for this information. We greatly appreciate the time and interest you have shown in our study. We will be in touch with you regarding participation if we are able to schedule a visit in your area. The information you provided is very important. Thank you and good-bye."* ***END OF INTERVIEW***

10b. If "No", could you please indicate your reason(s)? ***(Please X all that apply; then End Interview using Script B below).***

- ☐[1] ....Just Not Interested
- ☐[1] ....Not Enough Time
- ☐[1] ....Unwilling to Provide Blood Sample
- ☐[1] ....Not Well
- ☐[1] ....Concern about Ability to Complete Examination
- ☐[1] ....Privacy Issue/Concern
- ☐[1] ....Unwilling to Contact Family Members
- ☐[1] ....Lack of "Family" Interest
- ☐[1] ....Other, Please Specify: ________________________________

Script B – End Interview: *"Thank you so much for this information. We greatly appreciate the time and interest you have shown in our study. We will be in touch with you regarding participation if we are able to schedule a visit to your area. The information you provided is very important."* ***END OF INTERVIEW***

LONG LIFE FAMILY STUDY	**(Affix Label Here)**<br>Participant ID: ______________<br>Participant Name Code: __________	**Date Form Initiated** (e.g., 10JUN2005):<br>☐ ☐ ☐ ☐ ☐ ☐ ☐ ☐ ☐<br>d d m m m y y y y<br>**Date Form Completed** (e.g., 10JUN2005):<br>☐ ☐ ☐ ☐ ☐ ☐ ☐ ☐ ☐<br>d d m m m y y y y<br>Interviewer Code: ☐ ☐ ☐<br>Please Circle Field Center Location:<br>**BU CU DK UP**

LLFS Family Longevity Score (FLoSS) Instrument
Control Subjects (FLSI-c)
[UP & BU Pilot Only]

Interviewer Script: *Now I would like to ask you some questions about family. Please feel free to tell me if you don't know the answer to a question, but please try to answer to the best of your knowledge. I am going to ask about your brothers and sisters, your biological parents, your biological grand parents. We are using the word "biological" to mean individuals that are related to you by birth, that is, they share a blood relationship with you. Please note that none of the study investigators will attempt to identify or contact specific family members based on the relationship information you provide.*

1a. How many full-brothers and sisters do *[you/he or she]* have? Include those that are living and those that are deceased. Remember, please answer for blood relationships only.

_____ Full-Brothers _____ Full-Sisters

1b. How many half-brothers and sisters do *[you/he or she]* have? Include those that are living and those that are deceased. Remember, please answer for blood relationships only.

_____ Half-Brothers _____ Half-Sisters

Interviewer Script: *First, I'm going to ask you some questions about each of your biological brothers and sisters. I'm going to ask you the questions in order of oldest to youngest. Let's begin.*

2a. Is your oldest sibling a full- or half-brother or sister?

☐ 1 ....Full Brother ☐ 3 ....Half Brother
☐ 2 ....Full Sister ☐ 4 ....Half Sister

2b. Is this *[brother/sister]* still living?

☐ 1 ....Yes
☐ 0 ....No **Go to Q2d**
☐ D ....Don't Know **Go to Q3a**

2c. What is *[his/her]* current age in years? ____ ____ ____ years **if Don't Know, enter 999**

***Interviewer Note*: If brother or sister is deceased:**

2d. What was *[his/her]* age at time of death? ____ ____ ____ years **If Don't Know, enter 999**

2e. What was *[his/her]* year of birth? ____ ____ ____ ____

2f. Did *[he/she]* die as a result of an accident, injury or war? ***[Interviewer*: If death is result of murder or suicide, answer "Yes"; if death occurred during childbirth, answer "No".]**

- ☐ 1 ....Yes
- ☐ 0 ....No
- ☐ D ....Don't Know
- ☐ R ....Refused

3a. Is your second oldest sibling a full- or half-brother or sister?

- ☐ 1 ....Full Brother
- ☐ 2 ....Full Sister
- ☐ 3 ....Half Brother
- ☐ 4 ....Half Sister

3b. Is this *[brother/sister]* still living?

- ☐ 1 ....Yes
- ☐ 0 ....No **Go to Q3d**
- ☐ D ....Don't Know **Go to Q4a**

3c. What is *[his/her]* current age in years? ____ ____ ____ years i**f Don't Know, enter 999**

***Interviewer*: If brother or sister is deceased:**

3d. What was *[his/her]* age at time of death? ____ ____ ____ years **If Don't Know, enter 999**

3e. What was *[his/her]* year of birth? ____ ____ ____ ____

3f. Did *[he/she]* die as a result of an accident, injury or war? ***[Interviewer*: If death is result of murder or suicide, answer "Yes"; if death occurred during childbirth, answer "No".]**

- ☐ 1 ....Yes
- ☐ 0 ....No
- ☐ D ....Don't Know
- ☐ R ....Refused

4a. Is your third oldest sibling a full- or half-brother or sister?

☐[1] ....Full Brother ☐[3] ....Half Brother
☐[2] ....Full Sister ☐[4] ....Half Sister

4b. Is this *[brother/sister]* still living?

☐[1] ....Yes
☐[0] ....No **Go to Q4d**
☐[D] ....Don't Know **Go to Q5a**

4c. What is *[his/her]* current age in years? ____ ____ ____ years i**f Don't Know, enter 999**

***Interviewer**: If brother or sister is deceased:*

4d. What was *[his/her]* age at time of death? ____ ____ ____ years **If Don't Know, enter 999**

4e. What was *[his/her]* year of birth? ____ ____ ____ ____

4f. Did *[he/she]* die as a result of an accident, injury or war? ***[Interviewer**: If death is result of murder or suicide, answer "Yes"; if death occurred during childbirth, answer "No".]*

☐[1] ....Yes
☐[0] ....No
☐[D] ....Don't Know
☐[R] ....Refused

5a. Is your fourth oldest sibling a full- or half-brother or sister?

☐[1] ....Full Brother ☐[3] ....Half Brother
☐[2] ....Full Sister ☐[4] ....Half Sister

5b. Is this *[brother/sister]* still living?

☐[1] ....Yes
☐[0] ....No **Go to Q5d**
☐[D] ....Don't Know **Go to Q6a**

5c. What is *[his/her]* current age in years? ____ ____ ____ years i**f Don't Know, enter 999**

***Interviewer**: If brother or sister is deceased:*

5d. What was *[his/her]* age at time of death? ____ ____ ____ years **If Don't Know, enter 999**

5e. What was *[his/her]* year of birth? ____ ____ ____ ____

5f. Did *[he/she]* die as a result of an accident, injury or war? ***[Interviewer: If death is result of murder or suicide, answer "Yes"; if death occurred during childbirth, answer "No".]***

☐1 ....Yes
☐0 ....No
☐D ....Don't Know
☐R ....Refused

6a. Is your fifth oldest sibling a full- or half-brother or sister?

☐1 ....Full Brother ☐3 ....Half Brother
☐2 ....Full Sister ☐4 ....Half Sister

6b. Is this *[brother/sister]* still living?

☐1 ....Yes
☐0 ....No **Go to Q6d**
☐D ....Don't Know **Go to Q7a**

6c. What is *[his/her]* current age in years? ____ ____ ____ years **if Don't Know, enter 999**

Interviewer: If brother or sister is deceased:

6d. What was *[his/her]* age at time of death? ____ ____ ____ years **If Don't Know, enter 999**

6e. What was *[his/her]* year of birth? ____ ____ ____ ____

6f. Did *[he/she]* die as a result of an accident, injury or war? ***[Interviewer: If death is result of murder or suicide, answer "Yes"; if death occurred during childbirth, answer "No".]***

☐1 ....Yes
☐0 ....No
☐D ....Don't Know
☐R ....Refused

7a. Is your sixth oldest sibling a full- or half-brother or sister?

☐1 ....Full Brother ☐3 ....Half Brother
☐2 ....Full Sister ☐4 ....Half Sister

7b. Is this *[brother/sister]* still living?

☐1 ....Yes
☐0 ....No **Go to Q7d**
☐D ....Don't Know **Go to Q8a**

7c. What is [his/her] current age in years? ____ ____ ____ years **if Don't Know, enter 999**

***Interviewer**: If brother or sister is deceased:*

7d. What was [his/her] age at time of death? ____ ____ ____ years **If Don't Know, enter 999**

7e. What was [his/her] year of birth? ____ ____ ____ ____

7f. Did [he/she] die as a result of an accident, injury or war? ***[Interviewer**: If death is result of murder or suicide, answer "Yes"; if death occurred during childbirth, answer "No".]*

- ☐ 1 ....Yes
- ☐ 0 ....No
- ☐ D ....Don't Know
- ☐ R ....Refused

8a. Is your seventh oldest sibling a full- or half-brother or sister?

- ☐ 1 ....Full Brother
- ☐ 2 ....Full Sister
- ☐ 3 ....Half Brother
- ☐ 4 ....Half Sister

8b. Is this [brother/sister] still living?

- ☐ 1 ....Yes
- ☐ 0 ....No **Go to Q8d**
- ☐ D ....Don't Know **Go to Q9a**

8c. What is [his/her] current age in years? ____ ____ ____ years **if Don't Know, enter 999**

***Interviewer**: If brother or sister is deceased:*

8d. What was [his/her] age at time of death? ____ ____ ____ years **If Don't Know, enter 999**

8e. What was [his/her] year of birth? ____ ____ ____ ____

8f. Did [he/she] die as a result of an accident, injury or war? ***[Interviewer**: If death is result of murder or suicide, answer "Yes"; if death occurred during childbirth, answer "No".]*

- ☐ 1 ....Yes
- ☐ 0 ....No
- ☐ D ....Don't Know
- ☐ R ....Refused

9a. Is your eighth oldest sibling a full- or half-brother or sister?

☐1 ....Full Brother ☐3 ....Half Brother
☐2 ....Full Sister ☐4 ....Half Sister

9b. Is this *[brother/sister]* still living?

☐1 ....Yes
☐0 ....No **Go to Q9d**
☐D ....Don't Know **Go to Q10a**

9c. What is *[his/her]* current age in years? ____ ____ ____ years **if Don't Know, enter 999**

***Interviewer*: If brother or sister is deceased:**

9d. What was *[his/her]* age at time of death? ____ ____ ____ years **If Don't Know, enter 999**

9e. What was *[his/her]* year of birth? ____ ____ ____ ____

9f. Did *[he/she]* die as a result of an accident, injury or war? ***[Interviewer*: If death is result of murder or suicide, answer "Yes"; if death occurred during childbirth, answer "No".]**

☐1 ....Yes
☐0 ....No
☐D ....Don't Know
☐R ....Refused

10a. Is your ninth oldest sibling a full- or half-brother or sister?

☐1 ....Full Brother ☐3 ....Half Brother
☐2 ....Full Sister ☐4 ....Half Sister

10b. Is this *[brother/sister]* still living?

☐1 ....Yes
☐0 ....No **Go to Q10d**
☐D ....Don't Know **Go to Q11a**

10c. What is *[his/her]* current age in years? ____ ____ ____ years **if Don't Know, enter 999**

***Interviewer*: If brother or sister is deceased:**

10d. What was *[his/her]* age at time of death? ____ ____ ____ years **If Don't Know, enter 999**

10e. What was *[his/her]* year of birth? ____ ____ ____ ____

10f. Did [he/she] die as a result of an accident, injury or war? ***[Interviewer: If death is result of murder or suicide, answer "Yes"; if death occurred during childbirth, answer "No".]***

- ☐[1] ....Yes
- ☐[0] ....No
- ☐[D] ....Don't Know
- ☐[R] ....Refused

11a. Is your tenth oldest sibling a full- or half-brother or sister?

- ☐[1] ....Full Brother
- ☐[2] ....Full Sister
- ☐[3] ....Half Brother
- ☐[4] ....Half Sister

11b. Is this [brother/sister] still living?

- ☐[1] ....Yes
- ☐[0] ....No **Go to Q11d**
- ☐[D] ....Don't Know **Go to Q12a**

11c. What is [his/her] current age in years? ____ ____ ____ years **if Don't Know, enter 999**

Interviewer: If brother or sister is deceased:

11d. What was [his/her] age at time of death? ____ ____ ____ years **If Don't Know, enter 999**

11e. What was [his/her] year of birth? ____ ____ ____ ____

11f. Did [he/she] die as a result of an accident, injury or war? ***[Interviewer: If death is result of murder or suicide, answer "Yes"; if death occurred during childbirth, answer "No".]***

- ☐[1] ....Yes
- ☐[0] ....No
- ☐[D] ....Don't Know
- ☐[R] ....Refused

Interviewer: If the Screenee has more than 10 biological siblings, please use additional forms to record the information for these brothers and sisters.

Interviewer Script: *Now, I'm going to ask you some questions about your biological mother, your biological mother's brothers and sisters, and your biological father's parents (your maternal grandparents).*

12a. Has your biological mother (the woman that gave birth you) passed away?

- ☐[1] ....Yes
- ☐[0] ....No **If still living, please Go to Q12e**
- ☐[D] ....Don't Know **Go to Q13a**

12b. What was her age at time of death? ____ ____ ____ years **If Don't Know, enter 999**

12c. What was her year of death? ____ ____ ____ ____

12d. Did she die as a result of an accident, injury or war? ***[Interviewer: If death is result of murder or suicide, answer "Yes"; if death occurred during childbirth, answer "No".]***

☐1 ....Yes
☐0 ....No
☐D ....Don't Know
☐R ....Refused

***Interviewer: If biological mother is still living*:**

12e. What is her current age in years? ____ ____ ____ years **If Don't Know, enter 999**

12f. What was her year of birth? ____ ____ ____ ____

Interviewer Script: *Now, I'd like to ask you about your biological mother's brothers and sisters, that is, your maternal aunts and uncles. Remember: Please feel free to tell me if you don't know the answer to a question, but please try to give your best guess.*

13a. Is your mother's oldest sibling a full- or half-brother or sister?

☐1 ....Full Brother ☐3 ....Half Brother
☐2 ....Full Sister ☐4 ....Half Sister

13b. Is this *[brother/sister]* still living?

☐1 ....Yes
☐0 ....No **Go to Q13d**
☐D ....Don't Know **Go to Q14a**

13c. What is *[his/her]* current age in years? ____ ____ ____ years **if Don't Know, enter 999**

Interviewer Note*: *If mother's brother or sister is deceased:

13d. What was *[his/her]* age at time of death? ____ ____ ____ years **If Don't Know, enter 999**

13e. What was *[his/her]* year of birth? ____ ____ ____ ____

13f. Did *[he/she]* die as a result of an accident, injury or war? ***[Interviewer: If death is result of murder or suicide, answer "Yes"; if death occurred during childbirth, answer "No".]***

☐1 ....Yes
☐0 ....No
☐D ....Don't Know
☐R ....Refused

14a. Is your mother's second oldest sibling a full- or half-brother or sister?

☐1 ....Full Brother ☐3 ....Half Brother
☐2 ....Full Sister ☐4 ....Half Sister

14b. Is this *[brother/sister]* still living?

☐1 ....Yes
☐0 ....No **Go to Q14d**
☐D ....Don't Know **Go to Q15a**

14c. What is *[his/her]* current age in years? ____ ____ ____ years i**f Don't Know, enter 999**

Interviewer: If mother's brother or sister is deceased:

14d. What was *[his/her]* age at time of death? ____ ____ ____ years **If Don't Know, enter 999**

14e. What was *[his/her]* year of birth? ____ ____ ____ ____

14f. Did *[he/she]* die as a result of an accident, injury or war? ***[Interviewer: If death is result of murder or suicide, answer "Yes"; if death occurred during childbirth, answer "No".]***

☐1 ....Yes
☐0 ....No
☐D ....Don't Know
☐R ....Refused

15a. Is your mother's third oldest sibling a full- or half-brother or sister?

☐1 ....Full Brother ☐3 ....Half Brother
☐2 ....Full Sister ☐4 ....Half Sister

15b. Is this *[brother/sister]* still living?

☐1 ....Yes
☐0 ....No **Go to Q15d**
☐D ....Don't Know **Go to Q16a**

15c. What is *[his/her]* current age in years? ____ ____ ____ years i**f Don't Know, enter 999**

Interviewer: If mother's brother or sister is deceased:

15d. What was *[his/her]* age at time of death? ____ ____ ____ years **If Don't Know, enter 999**

15e. What was *[his/her]* year of birth? ____ ____ ____ ____

15f. Did *[he/she]* die as a result of an accident, injury or war? ***[Interviewer: If death is result of murder or suicide, answer "Yes"; if death occurred during childbirth, answer "No".]***

☐ 1 ....Yes
☐ 0 ....No
☐ D ....Don't Know
☐ R ....Refused

16a. Is your mother's fourth oldest sibling a full- or half-brother or sister?

☐ 1 ....Full Brother ☐ 3 ....Half Brother
☐ 2 ....Full Sister ☐ 4 ....Half Sister

16b. Is this *[brother/sister]* still living?

☐ 1 ....Yes
☐ 0 ....No **Go to Q16d**
☐ D ....Don't Know **Go to Q17a**

16c. What is *[his/her]* current age in years? ____ ____ ____ years **if Don't Know, enter 999**

Interviewer: If mother's brother or sister is deceased:

16d. What was *[his/her]* age at time of death? ____ ____ ____ years **If Don't Know, enter 999**

16e. What was *[his/her]* year of birth? ____ ____ ____ ____

16f. Did *[he/she]* die as a result of an accident, injury or war? ***[Interviewer: If death is result of murder or suicide, answer "Yes"; if death occurred during childbirth, answer "No".]***

☐ 1 ....Yes
☐ 0 ....No
☐ D ....Don't Know
☐ R ....Refused

17a. Is your mother's fifth oldest sibling a full- or half-brother or sister?

☐ 1 ....Full Brother ☐ 3 ....Half Brother
☐ 2 ....Full Sister ☐ 4 ....Half Sister

17b. Is this *[brother/sister]* still living?

☐ 1 ....Yes
☐ 0 ....No **Go to Q17d**
☐ D ....Don't Know **Go to Q18a**

17c. What is *[his/her]* current age in years? ____ ____ ____ years **if Don't Know, enter 999**

***Interviewer**: If mother's brother or sister is deceased:*

17d. What was *[his/her]* age at time of death? ____ ____ ____ years **If Don't Know, enter 999**

17e. What was *[his/her]* year of birth? ____ ____ ____ ____

17f. Did *[he/she]* die as a result of an accident, injury or war? ***[Interviewer**: If death is result of murder or suicide, answer "Yes"; if death occurred during childbirth, answer "No".]*

- ☐1 ....Yes
- ☐0 ....No
- ☐D ....Don't Know
- ☐R ....Refused

18a. Is your mother's sixth oldest sibling a full- or half-brother or sister?

- ☐1 ....Full Brother
- ☐2 ....Full Sister
- ☐3 ....Half Brother
- ☐4 ....Half Sister

18b. Is this *[brother/sister]* still living?

- ☐1 ....Yes
- ☐0 ....No **Go to Q18d**
- ☐D ....Don't Know **Go to Q19a**

18c. What is *[his/her]* current age in years? ____ ____ ____ years i**f Don't Know, enter 999**

***Interviewer**: If mother's brother or sister is deceased:*

18d. What was *[his/her]* age at time of death? ____ ____ ____ years **If Don't Know, enter 999**

18e. What was *[his/her]* year of birth? ____ ____ ____ ____

18f. Did *[he/she]* die as a result of an accident, injury or war? ***[Interviewer**: If death is result of murder or suicide, answer "Yes"; if death occurred during childbirth, answer "No".]*

- ☐1 ....Yes
- ☐0 ....No
- ☐D ....Don't Know
- ☐R ....Refused

19a. Is your mother's seventh oldest sibling a full- or half-brother or sister?

- ☐1 ....Full Brother
- ☐2 ....Full Sister
- ☐3 ....Half Brother
- ☐4 ....Half Sister

19b. Is this *[brother/sister]* still living?

☐[1] ....Yes
☐[0] ....No **Go to Q19d**
☐[D] ....Don't Know **Go to Q20a**

19c. What is *[his/her]* current age in years? ____ ____ ____ years **if Don't Know, enter 999**

***Interviewer*: If mother's brother or sister is deceased:**

19d. What was *[his/her]* age at time of death? ____ ____ ____ years **If Don't Know, enter 999**

19e. What was *[his/her]* year of birth? ____ ____ ____ ____

19f. Did *[he/she]* die as a result of an accident, injury or war? ***[Interviewer*: If death is result of murder or suicide, answer "Yes"; if death occurred during childbirth, answer "No".]**

☐[1] ....Yes
☐[0] ....No
☐[D] ....Don't Know
☐[R] ....Refused

20a. Is your mother's eighth oldest sibling a full- or half-brother or sister?

☐[1] ....Full Brother ☐[3] ....Half Brother
☐[2] ....Full Sister ☐[4] ....Half Sister

20b. Is this *[brother/sister]* still living?

☐[1] ....Yes
☐[0] ....No **Go to Q20d**
☐[D] ....Don't Know **Go to Q21a**

20c. What is *[his/her]* current age in years? ____ ____ ____ years **if Don't Know, enter 999**

***Interviewer*: If mother's brother or sister is deceased:**

20d. What was *[his/her]* age at time of death? ____ ____ ____ years **If Don't Know, enter 999**

20e. What was *[his/her]* year of birth? ____ ____ ____ ____

20f. Did *[he/she]* die as a result of an accident, injury or war? ***[Interviewer: If death is result of murder or suicide, answer "Yes"; if death occurred during childbirth, answer "No".]***

- ☐1 ....Yes
- ☐0 ....No
- ☐D ....Don't Know
- ☐R ....Refused

21a. Is your mother's ninth oldest sibling a full- or half-brother or sister?

- ☐1 ....Full Brother
- ☐2 ....Full Sister
- ☐3 ....Half Brother
- ☐4 ....Half Sister

21b. Is this *[brother/sister]* still living?

- ☐1 ....Yes
- ☐0 ....No **Go to Q21d**
- ☐D ....Don't Know **Go to Q22a**

21c. What is *[his/her]* current age in years? ____ ____ ____ years **if Don't Know, enter 999**

Interviewer: If mother's brother or sister is deceased:

21d. What was *[his/her]* age at time of death? ____ ____ ____ years **If Don't Know, enter 999**

21e. What was *[his/her]* year of birth? ____ ____ ____ ____

21f. Did *[he/she]* die as a result of an accident, injury or war? ***[Interviewer: If death is result of murder or suicide, answer "Yes"; if death occurred during childbirth, answer "No".]***

- ☐1 ....Yes
- ☐0 ....No
- ☐D ....Don't Know
- ☐R ....Refused

22a. Is your mother's tenth oldest sibling a full- or half-brother or sister?

- ☐1 ....Full Brother
- ☐2 ....Full Sister
- ☐3 ....Half Brother
- ☐4 ....Half Sister

22b. Is this *[brother/sister]* still living?

- ☐1 ....Yes
- ☐0 ....No **Go to Q22d**
- ☐D ....Don't Know **Go to Q23a**

22c. What is *[his/her]* current age in years? ____ ____ ____ years **if Don't Know, enter 999**

***Interviewer:* If mother's brother or sister is deceased:**

22d. What was *[his/her]* age at time of death? ____ ____ ____ years **If Don't Know, enter 999**

22e. What was *[his/her]* year of birth? ____ ____ ____ ____

22f. Did *[he/she]* die as a result of an accident, injury or war? ***[Interviewer:* If death is result of murder or suicide, answer "Yes"; if death occurred during childbirth, answer "No".]**

☐ 1 ....Yes
☐ 0 ....No
☐ D ....Don't Know
☐ R ....Refused

***Interviewer:* If the participant's mother has more than 10 biological siblings, please use additional forms to record the information for these brothers and sisters.**

Interviewer Script: *Now, I'd like to ask you about your biological mother's parents, that is, your maternal grandmother and grandfather. Remember: Please feel free to tell me if you don't know the answer to a question, but please try to give your best guess.*

23a. Has your biological maternal grandmother (your mother's natural mother) passed away?

☐ 1 ....Yes
☐ 0 ....No **If still living, please Go to Q23e**
☐ D ....Don't Know **Go to Q24a**

23b. What was her age at time of death? ____ ____ ____ years **If Don't Know, enter 999**

23c. What was her year of death? ____ ____ ____ ____

23d. Did she die as a result of an accident, injury or war? ***[Interviewer:* If death is result of murder or suicide, answer "Yes"; if death occurred during childbirth, answer "No".]**

☐ 1 ....Yes
☐ 0 ....No
☐ D ....Don't Know
☐ R ....Refused

***Interviewer:* If biological maternal grandmother is still living:**

23e. What is her current age in years? ____ ____ ____ years **If Don't Know, enter 999**

23f. What was her year of birth? ____ ____ ____ ____

24a. Has your biological maternal grandfather (your mother's natural father) passed away?

☐[1] ....Yes
☐[0] ....No **If still living, please Go to Q24e**
☐[D] ....Don't Know **Go to Q25a**

24b. What was his age at time of death? ____ ____ ____ years **If Don't Know, enter 999**

24c. What was his year of death? ____ ____ ____ ____

24d. Did he die as a result of an accident, injury or war? ***[<u>Interviewer</u>: If death is result of murder or suicide, answer "Yes"; if death occurred during childbirth, answer "No".]***

☐[1] ....Yes
☐[0] ....No
☐[D] ....Don't Know
☐[R] ....Refused

<u>Interviewer</u>: If biological maternal grandfather is still living:

24e. What is his current age in years? ____ ____ ____ years **If Don't Know, enter 999**

24f. What was his year of birth? ____ ____ ____ ____

<u>Interviewer Script</u>: *Finally, I'm going to ask you some questions about your biological father, your biological father's brothers and sisters and your biological father's parents (your paternal grandparents).*

25a. Has your biological father passed away? Remember, please answer for blood relationships only, not a stepfather.

☐[1] ....Yes
☐[0] ....No **If still living, please Go to Q25e**
☐[D] ....Don't Know **Go to Q26a**

25b. What was his age at time of death? ____ ____ ____ years **If Don't Know, enter 999**

25c. What was his year of death? ____ ____ ____ ____

25d. Did he die as a result of an accident, injury or war? ***[<u>Interviewer</u>: If death is result of murder or suicide, answer "Yes".]***

☐[1] ....Yes
☐[0] ....No
☐[D] ....Don't Know
☐[R] ....Refused

***Interviewer*: *If biological father is still living*:**

25e. What is his current age in years? ____ ____ ____ years **If Don't Know, enter 999**

25f. What was his year of birth? ____ ____ ____ ____

***Interviewer Script*:** *Now, I'd like to ask you about your biological father's brothers and sisters, that is, your paternal aunts and uncles. Remember: Please feel free to tell me if you don't know the answer to a question, but please try to give your best guess.*

26a. Is your father's oldest sibling a full- or half-brother or sister?

☐1 ....Full Brother ☐3 ....Half Brother
☐2 ....Full Sister ☐4 ....Half Sister

26b. Is this *[brother/sister]* still living?

☐1 ....Yes
☐0 ....No **Go to Q26d**
☐D ....Don't Know **Go to Q27a**

26c. What is *[his/her]* current age in years? ____ ____ ____ years **if Don't Know, enter 999**

Interviewer Note*: *If father's brother or sister is deceased:

26d. What was *[his/her]* age at time of death? ____ ____ ____ years **If Don't Know, enter 999**

26e. What was *[his/her]* year of birth? ____ ____ ____ ____

26f. Did *[he/she]* die as a result of an accident, injury or war? ***[Interviewer*: *If death is result of murder or suicide, answer "Yes"; if death occurred during childbirth, answer "No".]***

☐1 ....Yes
☐0 ....No
☐D ....Don't Know
☐R ....Refused

27a. Is your father's second oldest sibling a full- or half-brother or sister?

☐1 ....Full Brother ☐3 ....Half Brother
☐2 ....Full Sister ☐4 ....Half Sister

27b. Is this *[brother/sister]* still living?

☐1 ....Yes
☐0 ....No **Go to Q27d**
☐D ....Don't Know **Go to Q28a**

27c. What is *[his/her]* current age in years? ____ ____ ____ years **if Don't Know, enter 999**

***Interviewer*: If father's brother or sister is deceased:**

27d. What was *[his/her]* age at time of death? ____ ____ ____ years **If Don't Know, enter 999**

27e. What was *[his/her]* year of birth? ____ ____ ____ ____

27f. Did *[he/she]* die as a result of an accident, injury or war? ***[Interviewer*: If death is result of murder or suicide, answer "Yes"; if death occurred during childbirth, answer "No".]**

- ☐ 1 ....Yes
- ☐ 0 ....No
- ☐ D ....Don't Know
- ☐ R ....Refused

28a. Is your father's third oldest sibling a full- or half-brother or sister?

- ☐ 1 ....Full Brother
- ☐ 3 ....Half Brother
- ☐ 2 ....Full Sister
- ☐ 4 ....Half Sister

28b. Is this *[brother/sister]* still living?

- ☐ 1 ....Yes
- ☐ 0 ....No **Go to Q28d**
- ☐ D ....Don't Know **Go to Q29a**

28c. What is *[his/her]* current age in years? ____ ____ ____ years i**f Don't Know, enter 999**

***Interviewer*: If father's brother or sister is deceased:**

28d. What was *[his/her]* age at time of death? ____ ____ ____ years **If Don't Know, enter 999**

28e. What was *[his/her]* year of birth? ____ ____ ____ ____

28f. Did *[he/she]* die as a result of an accident, injury or war? ***[Interviewer*: If death is result of murder or suicide, answer "Yes"; if death occurred during childbirth, answer "No".]**

- ☐ 1 ....Yes
- ☐ 0 ....No
- ☐ D ....Don't Know
- ☐ R ....Refused

29a. Is your father's fourth oldest sibling a full- or half-brother or sister?

- ☐ 1 ....Full Brother
- ☐ 3 ....Half Brother
- ☐ 2 ....Full Sister
- ☐ 4 ....Half Sister

29b. Is this *[brother/sister]* still living?

- ☐ 1 ....Yes
- ☐ 0 ....No **Go to Q29d**
- ☐ D ....Don't Know **Go to Q30a**

29c. What is *[his/her]* current age in years? ____ ____ ____ years if **Don't Know, enter 999**

Interviewer: If father's brother or sister is deceased:

29d. What was *[his/her]* age at time of death? ____ ____ ____ years **If Don't Know, enter 999**

29e. What was *[his/her]* year of birth? ____ ____ ____ ____

29f. Did *[he/she]* die as a result of an accident, injury or war? ***[Interviewer: If death is result of murder or suicide, answer "Yes"; if death occurred during childbirth, answer "No".]***

- ☐ 1 ....Yes
- ☐ 0 ....No
- ☐ D ....Don't Know
- ☐ R ....Refused

30a. Is your father's fifth oldest sibling a full- or half-brother or sister?

- ☐ 1 ....Full Brother
- ☐ 3 ....Half Brother
- ☐ 2 ....Full Sister
- ☐ 4 ....Half Sister

30b. Is this *[brother/sister]* still living?

- ☐ 1 ....Yes
- ☐ 0 ....No **Go to Q30d**
- ☐ D ....Don't Know **Go to Q31a**

30c. What is *[his/her]* current age in years? ____ ____ ____ years if **Don't Know, enter 999**

Interviewer: If father's brother or sister is deceased:

30d. What was *[his/her]* age at time of death? ____ ____ ____ years **If Don't Know, enter 999**

30e. What was *[his/her]* year of birth? ____ ____ ____ ____

30f. Did *[he/she]* die as a result of an accident, injury or war? ***[Interviewer: If death is result of murder or suicide, answer "Yes"; if death occurred during childbirth, answer "No".]***

- ☐ 1 ....Yes
- ☐ 0 ....No
- ☐ D ....Don't Know
- ☐ R ....Refused

31a. Is your father's sixth oldest sibling a full- or half-brother or sister?

- ☐ 1 ....Full Brother
- ☐ 2 ....Full Sister
- ☐ 3 ....Half Brother
- ☐ 4 ....Half Sister

31b. Is this *[brother/sister]* still living?

- ☐ 1 ....Yes
- ☐ 0 ....No **Go to Q31d**
- ☐ D ....Don't Know **Go to Q32a**

31c. What is *[his/her]* current age in years? ____ ____ ____ years **if Don't Know, enter 999**

Interviewer: If father's brother or sister is deceased:

31d. What was *[his/her]* age at time of death? ____ ____ ____ years **If Don't Know, enter 999**

31e. What was *[his/her]* year of birth? ____ ____ ____ ____

31f. Did *[he/she]* die as a result of an accident, injury or war? ***[Interviewer: If death is result of murder or suicide, answer "Yes"; if death occurred during childbirth, answer "No".]***

- ☐ 1 ....Yes
- ☐ 0 ....No
- ☐ D ....Don't Know
- ☐ R ....Refused

32a. Is your father's seventh oldest sibling a full- or half-brother or sister?

- ☐ 1 ....Full Brother
- ☐ 2 ....Full Sister
- ☐ 3 ....Half Brother
- ☐ 4 ....Half Sister

32b. Is this *[brother/sister]* still living?

- ☐ 1 ....Yes
- ☐ 0 ....No **Go to Q32d**
- ☐ D ....Don't Know **Go to Q33a**

32c. What is *[his/her]* current age in years? ____ ____ ____ years **if Don't Know, enter 999**

***Interviewer*: If father's brother or sister is deceased:**

32d. What was *[his/her]* age at time of death? ____ ____ ____ years **If Don't Know, enter 999**

32e. What was *[his/her]* year of birth? ____ ____ ____ ____

32f. Did *[he/she]* die as a result of an accident, injury or war? ***[Interviewer*: If death is result of murder or suicide, answer "Yes"; if death occurred during childbirth, answer "No".]**

- ☐ 1 ....Yes
- ☐ 0 ....No
- ☐ D ....Don't Know
- ☐ R ....Refused

33a. Is your father's eighth oldest sibling a full- or half-brother or sister?

- ☐ 1 ....Full Brother
- ☐ 2 ....Full Sister
- ☐ 3 ....Half Brother
- ☐ 4 ....Half Sister

33b. Is this *[brother/sister]* still living?

- ☐ 1 ....Yes
- ☐ 0 ....No **Go to Q33d**
- ☐ D ....Don't Know **Go to Q34a**

33c. What is *[his/her]* current age in years? ____ ____ ____ years i**f Don't Know, enter 999**

***Interviewer*: If father's brother or sister is deceased:**

33d. What was *[his/her]* age at time of death? ____ ____ ____ years **If Don't Know, enter 999**

33e. What was *[his/her]* year of birth? ____ ____ ____ ____

33f. Did *[he/she]* die as a result of an accident, injury or war? ***[Interviewer*: If death is result of murder or suicide, answer "Yes"; if death occurred during childbirth, answer "No".]**

- ☐ 1 ....Yes
- ☐ 0 ....No
- ☐ D ....Don't Know
- ☐ R ....Refused

34a. Is your father's ninth oldest sibling a full- or half-brother or sister?

- ☐ 1 ....Full Brother
- ☐ 2 ....Full Sister
- ☐ 3 ....Half Brother
- ☐ 4 ....Half Sister

34b. Is this *[brother/sister]* still living?

☐[1] ....Yes
☐[0] ....No **Go to Q34d**
☐[D] ....Don't Know **Go to Q35a**

34c. What is *[his/her]* current age in years? ____ ____ ____ years if **Don't Know, enter 999**

***Interviewer*: If father's brother or sister is deceased:**

34d. What was *[his/her]* age at time of death? ____ ____ ____ years **If Don't Know, enter 999**

34e. What was *[his/her]* year of birth? ____ ____ ____ ____

34f. Did *[he/she]* die as a result of an accident, injury or war? ***[Interviewer*: If death is result of murder or suicide, answer "Yes"; if death occurred during childbirth, answer "No".]**

☐[1] ....Yes
☐[0] ....No
☐[D] ....Don't Know
☐[R] ....Refused

35a. Is your father's tenth oldest sibling a full- or half-brother or sister?

☐[1] ....Full Brother ☐[3] ....Half Brother
☐[2] ....Full Sister ☐[4] ....Half Sister

35b. Is this *[brother/sister]* still living?

☐[1] ....Yes
☐[0] ....No **Go to Q35d**
☐[D] ....Don't Know **Go to Q36a**

35c. What is *[his/her]* current age in years? ____ ____ ____ years if **Don't Know, enter 999**

***Interviewer*: If father's brother or sister is deceased:**

35d. What was *[his/her]* age at time of death? ____ ____ ____ years **If Don't Know, enter 999**

35e. What was *[his/her]* year of birth? ____ ____ ____ ____

35f. Did *[he/she]* die as a result of an accident, injury or war? ***[Interviewer*: If death is result of murder or suicide, answer "Yes"; if death occurred during childbirth, answer "No".]**

☐[1] ....Yes
☐[0] ....No
☐[D] ....Don't Know
☐[R] ....Refused

***Interviewer*: If the participant's father has more than 10 biological siblings, please use additional forms to record the information for these brothers and sisters.**

***Interviewer Script*:** *Now, I'd like to ask you about your biological father's parents, that is, your paternal grandmother and grandfather. Remember: Please feel free to tell me if you don't know the answer to a question, but please try to give your best guess.*

36a. Has your biological paternal grandmother (your father's natural mother) passed away?

- ☐[1] ....Yes
- ☐[0] ....No **If still living, please Go to Q36e**
- ☐[D] ....Don't Know **Go to Q37a**

36b. What was her age at time of death? ____ ____ ____ years **If Don't Know, enter 999**

36c. What was her year of birth? ____ ____ ____ ____

36d. Did she die as a result of an accident, injury or war? ***[Interviewer*: If death is result of murder or suicide, answer "Yes"; if death occurred during childbirth, answer "No".]**

- ☐[1] ....Yes
- ☐[0] ....No
- ☐[D] ....Don't Know
- ☐[R] ....Refused

***Interviewer*: If biological paternal grandmother is still living**:

36e. What is her current age in years? ____ ____ ____ years **If Don't Know, enter 999**

36f. What was her year of birth? ____ ____ ____ ____

37a. Has your biological paternal grandfather (your father's natural father) passed away?

- ☐[1] ....Yes
- ☐[0] ....No **If still living, please Go to Q37e**
- ☐[D] ....Don't Know **End Interview**

37b. What was his age at time of death? ____ ____ ____ years **If Don't Know, enter 999**

37c. What was his year of birth? ____ ____ ____ ____

37d. Did he die as a result of an accident, injury or war? ***[Interviewer*: If death is result of murder or suicide, answer "Yes"; if death occurred during childbirth, answer "No".]**

- ☐[1] ....Yes
- ☐[0] ....No
- ☐[D] ....Don't Know
- ☐[R] ....Refused

***<u>Interviewer</u>:* *If biological paternal grandfather is still living*:**

37e. What is his current age in years? ____ ____ ____ years **If Don't Know, enter 999**

37f. What was his year of birth? ____ ____ ____ ____

REPLY FORM – The LONG LIFE Family Study

Please, only complete and return this form
if you have <u>at least one living</u> brother or sister

☐ **Yes, I am interested <u>and</u> I have at least one living brother or sister. Please contact me with more information!**

Name: ____

Address: ____

City: ______________________ State: __________ Zip: ___________

Phone # (   ) __________________ Date of Birth: _____ / _____ / _____

Best day and time to call me: ______________________________

Because we study longevity in families, we would like to know the ages of your brothers and/or sisters. Please check the appropriate box and indicate either their current age or age at which they passed away (you can estimate). Thank you.

Brother? ☐ or Sister? ☐ Age:_____ Still living? Yes ☐ No ☐

Brother? ☐ or Sister? ☐ Age:_____ Still living? Yes ☐ No ☐

Brother? ☐ or Sister? ☐ Age:_____ Still living? Yes ☐ No ☐

Brother? ☐ or Sister? ☐ Age:_____ Still living? Yes ☐ No ☐

Brother? ☐ or Sister? ☐ Age:_____ Still living? Yes ☐ No ☐

If you prefer we contact a family member or caregiver to discuss our study, please list the name and contact information below.

Contact's Name: ____

Contact's Address: ______________________________________

____

Contact's Phone # (   ) ____________________________

*****Thank you for your interest in The LONG LIFE Family Study!*****

LONG LIFE FAMILY STUDY	**(Affix Label Here)**<br>Participant ID: ____________________<br>Participant Name Code: ____________	Date Form Filled Out:<br>☐☐☐☐☐☐☐☐☐<br>d d M M M y y y y<br>(e.g., 10JUN2005)<br>Interviewer Code: ☐☐☐<br>Circle Field Center Location:<br>BU CU DK UP

Consent Tracking and Interview Feasibility

Please Mark the Appropriate Box Below:

☐ 1 ....This Form was Administered In-Person by Study Personnel
☐ 2 ....This Form was Administered via Telephone by Study Personnel

Informed Consent

1. Verify that informed consent is being provided by the Participant:

☐ 1 ....Yes
☐ 0 ....No

2a. Date Participant signed LLFS Consent Form: ☐☐/☐☐☐/☐☐☐☐
d d / m m m / y y y y

2b. Version Number ☐☐

or

Version Date ☐☐/☐☐☐/☐☐☐☐
d d / m m m / y y y y

2c. Consent form documents that participant allows blinded data/samples to be shared with other investigators:

☐ 1 ....Yes
☐ 0 ....No

2d. Consent form documents that participant allows samples to be stored for future research:

☐ 1 ....Yes
☐ 0 ....No

3. Date Participant signed HIPAA Authorization *(not applicable to BU or UP Field Centers; see Q2a)*:

☐☐/☐☐☐/☐☐☐☐
d d / m m m / y y y y

Interview Feasibility

Is the Respondent able to . . .	Yes, Without Any Difficulty	Yes, with Little Difficulty	Yes, with Great Difficulty	No	N/A (Mark for Phone Visits)
4a. See?	☐ 3	☐ 2	☐ 1	☐ 0	☐ N
4b. Hear?	☐ 3	☐ 2	☐ 1	☐ 0	
4c. Understand?	☐ 3	☐ 2	☐ 1	☐ 0	
4d. Speak?	☐ 3	☐ 2	☐ 1	☐ 0	

***Interviewer**: After completing this section, please use your best judgment to determine whether any visual, auditory or cognitive impairments will make it impossible for the participant to participate in this study. If you reach this conclusion, please check the appropriate box below and write down which impairment(s) are severe enough to warrant a discontinuation of this study visit.*

4e. Is the examination feasible?

☐ 1 ....Yes
☐ 0 ....No Reason: ____

---

5. Is the participant confined to *[his/her]* bed? *(Only out of bed when going to the toilet and taking a bath)*

☐ 1 ....Yes
☐ 0 ....No
☐ N ....Not Applicable (Participating in Phone Visit)

LONG LIFE FAMILY STUDY	(Affix Label Here)	Date Form Filled Out:
	Participant ID: ____________________	☐☐☐☐☐☐☐☐☐
		d d M M M y y y y
		(e.g., 10JUN2005)
	Participant Name Code: _____________	Interviewer Code: ☐☐☐
		Circle Field Center Location:
		BU CU DK UP

Assessment for Capacity
To Provide Informed Consent

After reviewing the consent form, please read the following:

Interviewer Script: *"I'm going to ask you a couple of short questions. Please feel free to refer to the consent form we just reviewed."*

1. In your own words, please explain the general purpose of this study. __________________________
____
____

2. In your own words, please explain the potential risks and benefits of participating in this study. ___
____
____

3. In your own words, please describe the activities you will participate in during this study. ________
____
____

4. In your own words, please explain your options if you do not want to participate in the study, or in certain parts of the study. ____
____
____

***Interviewer Note:* Based on the participant's responses to the above questions, the interviewer determines whether or not the person has sufficient understanding to provide informed consent. It is important to note that this is not a memory test, and participants can use the consent form to help them answer these questions and/or request clarification from the examiner.**

Based on the responses to the questions above, is it your opinion that this participant is adequately able to provide informed consent to participate in this study?

☐ 1 .... Yes
☐ 0 .... No

LONG LIFE FAMILY STUDY	**(Affix Label Here)**<br>Participant ID: ___________________<br>Participant Name Code: ____________	Date Form Filled Out: ☐☐☐☐☐☐☐☐☐<br>d d M M M y y y y<br>(e.g., 10JUN2005)<br>Interviewer Code: ☐☐☐<br>Circle Field Center Location:<br>**BU CU DK UP**

Criteria for Proxy-Based Interviews

Interviewer Note: The following table outlines four scenarios based on the initial cognitive screen (MMSE) and the examiner's impression of the participant's self-report. Indicate the scenario that applies by checking the appropriate box in the left column. Proceed as indicated in the right column. Note that these categories are mutually exclusive, so only one scenario should be marked as "Yes" and all others should be marked as "No".

	**Results of Cognitive Screen**	**Administration Procedure**
☐1 Yes<br>☐0 No	Participant scored above 24 on the Mini-Mental State Examination and there is no significant concern regarding the participant's ability to provide accurate self-report.	Proceed with interview. Proxy-based interview not necessary.
☐1 Yes<br>☐0 No	Participant scored above 24 on the Mini-Mental State Examination but there is significant concern regarding the participant's ability to provide accurate self-report.	Administer proxy-based interviews.
☐1 Yes<br>☐0 No	Participant scored 24 or below on the Mini-Mental State Examination and this impaired score is not due to a sensory impairment.	Administer proxy-based interviews.
☐1 Yes<br>☐0 No	Participant scored 24 or below on the Mini-Mental State Examination; however, this impaired score is due primarily to a sensory impairment; cognitive abilities appear intact.	Proceed with interview. Proxy-based interview not necessary.

LONG LIFE FAMILY STUDY	**(Affix Label Here)**<br><br>Participant ID: ____________________<br><br>Participant Name Code: _____________	Date Form Filled Out:<br>☐☐☐☐☐☐☐☐☐<br>d d M M M y y y y<br>(e.g., 10JUN2005)<br><br>Interviewer Code: ☐☐☐<br><br>Circle Field Center Location:<br>**BU CU DK UP**

Socio-Demographic Information
(Danish Version)

***For Internal Use Only* – Please Mark the Appropriate Box Below:**

☐1 ....This Form was Administered via a DFR/Proxy
☐2 ....This Form was Administered In-Person by Study Personnel
☐3 ....This Form was Administered via Telephone by Study Personnel
☐4 ....This Form was Mailed and Self-Administered by Participant
☐5 ....This Form was Administered by Other: ____________________

Interviewer:* *Please ask participant for verification of their date of birth and complete Questions 1 and 2.

***P**1. Date of Birth: Day: ____ ____ Month: ____ ____ ____ Year: ____ ____ ____ ____
(Example: 10 JUN 2005)

***P**2. Which item was used to provide proof of age?

☐1 ....Birth certificate
☐2 ....Church record
☐3 ....Family bible
☐4 ....Military record
☐5 ....Census record
☐6 ....Passport
☐7 ....Driver's license
☐8 ....No official source
☐9 ....CPR-Register
☐10 ....Other (Please Specify)_________________________

***P**2a. Date of birth verified?

☐1 ....Yes
☐0 ....No, reason ___________________________________

***P**3a. Were you born in Denmark?

- ☐[1] ....Yes
- ☐[0] ....No **Go to Q4a**

P3b. In what city/town, county and state were you born?

City/Town: ____

County: ____

State: ____ **Go to Q5**

P4a. In what country were you born? ______________________________________

P4b. When did you come to Denmark?

Year: ___ ___ ___ ___ **OR** Age: ___ ___ ___

5. Where did you live the majority of your childhood, prior to reaching the age of 16 years?

City/Town: ____

County: ____

State: ____

Country: ____

***P**6a. What is your current housing situation?

- ☐[1] ....House, including Townhouse and Farm **Go to Q6b**
- ☐[2] ....Apartment/Co-op/Condominium **Go to Q6b**
- ☐[3] ....Assisted Living/Other Special Housing for Older Adults **Go to Q7**
- ☐[4] ....Nursing Home **Go to Q7**
- ☐[5] ....Other (Please Specify) _________________________ **Go to Q7, if group dwelling**

P6b. How many people are living in your home apart from yourself? __________ people **If 0, Go to Q7**

***P**6c. Do you live together with? (X all that apply)

- ☐[1] ....Spouse/Partner
- ☐[1] ....Sisters/Brothers
- ☐[1] ....Child (children)
- ☐[1] ....Grandchild
- ☐[1] ....Other Relatives
- ☐[1] ....Close friends/friends
- ☐[1] ....Other (Please Specify) ___________________

***P**7. What is your gender?

- ☐[1] ....Male
- ☐[2] ....Female

8. Are you Spanish/Hispanic/Latino? ***[Interviewer: Leave Blank]***

- ☐[1] ....Yes, Mexican, Mexican American/Chicano
- ☐[2] ....Yes, Puerto Rican
- ☐[3] ....Yes, Cuban
- ☐[4] ....Yes, Other Spanish/Hispanic Latino
- ☐[0] ....No, not Spanish/Hispanic/Latino

9. What is your race? (X all that apply) ***[Interviewer: Leave Blank]***

- ☐[1] ....White
- ☐[1] ....Black or African American
- ☐[1] ....American Indian or Alaska Native
- ☐[1] ....Asian
- ☐[1] ....Native Hawaiian or other Pacific Islander
- ☐[1] ....Other (Please Specify)_________________________

***P**10. What is the highest degree or level of school that you have completed?

- ☐[0] ....Never went to school
- ☐[1] ....No schooling completed
- ☐[2] ....Nursery school to 4th grade
- ☐[3] ....5th grade or 6th grade
- ☐[4] ....7th grade or 8th grade
- ☐[5] ....9th grade
- ☐[6] ....10th grade
- ☐[7] ....11th grade
- ☐[8] ....12th grade – No high school diploma or GED
- ☐[9] ....High school graduate – Diploma or GED
- ☐[10] ....Vocational Training *How Many Years?* __________
  (technical, agricultural trade or craft)
- ☐[11] ....Some college credit but less than one year
- ☐[12] ....1 or more years of college – no degree
- ☐[13] ....Associate degree (AA, AS)
- ☐[14] ....Bachelor's degree (BA, AB, BS)
- ☐[15] ....Master's degree (MA, MS, Meng, Med, MSW, MBA)
- ☐[16] ....Professional degree (MD, DDS, DVM, LLB, JD)
- ☐[17] ....Doctorate degree (PhD, EdD)
- ☐[D] ....Do Not Know
- ☐[R] ....Refused

Now I would like to ask about your MAIN occupation.

11a. What was your main occupation for most of your life? Include unpaid work that you may have done on a farm, in a business or as a homemaker. ***Interviewer Note*: *If [he/she] never worked, check the box below and skip to Q12a. If participant is currently working, check the box for "Currently Working" and note the participant's main occupation, despite [his/her] current role. Since we are interested in the participant's main occupation, [his/her] current role (if still working), may not be [his/her] main occupation, especially if the participant is working part-time or on a 'side job'.***

(Examples of occupations include the following: janitor, farm laborer, bus driver, postal clerk, registered nurse, teacher, auto mechanic, lawyer, doctor, accountant, housewife, unpaid work on a farm, etc.) ____

____

☐[N] Never worked **Go to Q12a**
☐[R] Refused **Go to Q12a**
☐[1] Currently Working **Go to Q11b and Read Instructions Carefully Before Answering**

11b. How many subordinates did you have when you stopped working? ***Interviewer Note*: *If the participant is still working at [his/her] main occupation, you may enter the current number of subordinates. If the participant is currently unemployed or working in a job outside of [his/her] main occupation (i.e. part-time, 'side job', etc.), enter the number of subordinates the participant had when [he/she] stopped working from [his/her] main occupation.*** ____ ____ ____

***P**12a. What is your current marital status?

☐[1] ....Married, indicate age of spouse: _________years
☐[2] ....Separated, indicate year: ___ ___ ___ ___
☐[3] ....Divorced, indicate year: ___ ___ ___ ___
☐[4] ....Widowed, indicate year: ___ ___ ___ ___
☐[0] ....Never married **Go to Q15a**

12b. In what year or what age were you when your first marriage began?

Year: ___ ___ ___ ___ **OR** Age: ___ ___ ___

12c. How many times have you been married? ____ ____ Times

P13. What is the highest degree or level of school that your spouse completed? If you were married more than once, answer this question for the spouse to which you were married the longest. ***(Please Use Response Form in Appendix B)***

- ☐ 0 ....Never went to school
- ☐ 1 ....No schooling completed
- ☐ 2 ....Nursery school to 4th grade
- ☐ 3 ....5th grade or 6th grade
- ☐ 4 ....7th grade or 8th grade
- ☐ 5 ....9th grade
- ☐ 6 ....10th grade
- ☐ 7 ....11th grade
- ☐ 8 ....12th grade – No high school diploma or GED
- ☐ 9 ....High school graduate – Diploma or GED
- ☐ 10 ....Vocational Training *How Many Years?* _________
  (technical, agricultural trade or craft)
- ☐ 11 ....Some college credit but less than one year
- ☐ 12 ....1 or more years of college – no degree
- ☐ 13 ....Associate degree (AA, AS)
- ☐ 14 ....Bachelor's degree (BA, AB, BS)
- ☐ 15 ....Master's degree (MA, MS, Meng, Med, MSW, MBA)
- ☐ 16 ....Professional degree (MD, DDS, DVM, LLB, JD)
- ☐ 17 ....Doctorate degree (PhD, EdD)
- ☐ D ....Do Not Know
- ☐ R ....Refused

Now I would like to ask about your spouse's MAIN occupation.

14a. What was the main occupation of your spouse for most of his/her life? Include unpaid work that he/she may have done on a farm, in a business or as a homemaker. If you have been married more than once, answer this question for the spouse with whom you were married the longest. **Interviewer Note: If he/she never worked, check the box and skip to Q15.**

(Examples of occupations include the following: janitor, farm laborer, bus driver, postal clerk, registered nurse, teacher, auto mechanic, lawyer, doctor, accountant, housewife, unpaid work on a farm, etc.) ____

____

- ☐ N Never worked **Go to Q15a**
- ☐ R Refused **Go to Q15a**

14b. How many subordinates did your spouse have when [*he/she*] stopped working? ____ ____ ____

---

15. During most of your life, how hard was it for you (and your family) to pay for the very basics like food, clothing and housing? (***Please use Response Form in Appendix B***)

☐5 ....Very Easy
☐4 ....Easy
☐3 ....OK
☐2 ....Hard
☐1 ....Very Hard
☐D ....Don't Know
☐R ....Refused

***P**16. Do you currently own or do you rent your principal place of residence, or the place where you usually live?

☐1 ....Own
☐2 ....Rent
☐3 ....Other: ___________________________
☐D ....Don't Know
☐R ....Refused

LONG LIFE FAMILY STUDY	(Affix Label Here)<br><br>Participant ID: ________________<br><br>Participant Name Code: __________	Date Form Filled Out:<br>☐☐☐☐☐☐☐☐☐<br>d d M M M y y y y<br>(e.g., 10JUN2005)<br><br>Interviewer Code: ☐☐☐<br><br>Circle Field Center Location:<br>BU CU DK UP

Physical Function and Activity

Please Mark the Appropriate Box Below:

☐1 ....This Form was Administered via a DFR/Proxy
☐2 ....This Form was Administered In-Person by Study Personnel
☐3 ....This Form was Administered via Telephone by Study Personnel
☐4 ....This Form was Mailed and Self-Administered by Participant
☐5 ....This Form was Administered by Other: ____________________

P1. In a typical week, how often do you get together with friends, neighbors, your children or other relatives, other than those you live with? ***(Please Use Response Form in Appendix B.)***

☐5 ....Daily
☐4 ....4 to 6 Times per Week
☐3 ....2 to 3 Times per Week
☐2 ....Once per Week
☐1 ....Less than Once per Week

P2. In a typical week, how often do you spend the entire day alone?

☐5 ....Daily
☐4 ....4 to 6 Times per Week
☐3 ....2 to 3 Times per Week
☐2 ....Once per Week
☐1 ....Less than Once per Week

---

***P**3a. Do you have any difficulty getting in and out of bed or chairs without help from another person or special equipment?

☐1 ....Yes
☐0 ....No **Go to Q4a**
☐D ....Don't Know **Go to Q4a**
☐R ....Refused **Go to Q4a**

P**3b. How much difficulty would you say you have? Would you say . . . ***(Please Use Response Form in Appendix B.)

- ☐[1] ....A little difficulty
- ☐[2] ....Some difficulty
- ☐[3] ....A lot of difficulty
- ☐[0] ....I am unable to do it
- ☐[D] ....Don't Know

***P**3c. Do you usually receive help from another person getting in and out of bed or chairs?

- ☐[1] ....Yes
- ☐[0] ....No

***P**4a. Do you have any difficulty bathing or showering without help from another person or special equipment?

- ☐[1] ....Yes
- ☐[0] ....No **Go to Q5a**
- ☐[D] ....Don't Know **Go to Q5a**
- ☐[R] ....Refused **Go to Q5a**

P**4b. How much difficulty would you say you have? Would you say . . . ***(Please Use Response Form in Appendix B.)

- ☐[1] ....A little difficulty
- ☐[2] ....Some difficulty
- ☐[3] ....A lot of difficulty
- ☐[0] ....I am unable to do it
- ☐[D] ....Don't Know

***P**4c. Do you usually receive help from another person bathing or showering?

- ☐[1] ....Yes
- ☐[0] ....No

***P**5a. Do you have any difficulty walking across a small room without help from another person or special equipment?

- ☐[1] ....Yes
- ☐[0] ....No **Go to Q6a**
- ☐[D] ....Don't Know **Go to Q6a**
- ☐[R] ....Refused **Go to Q6a**

P**5b. How much difficulty would you say you have? Would you say . . . ***(Please Use Response Form in Appendix B.)

- ☐[1] ....A little difficulty
- ☐[2] ....Some difficulty
- ☐[3] ....A lot of difficulty
- ☐[0] ....I am unable to do it
- ☐[D] ....Don't Know

***P**5c. Do you usually receive help from another person walking across a small room?

- ☐[1] ....Yes
- ☐[0] ....No

***P**6a. Because of a health or physical problem, do you have any difficulty walking a quarter of a mile (2-3 blocks)?

- ☐[1] ....Yes
- ☐[0] ....No **Go to Q6d**

P**6b. How much difficulty would you say you have? Would you say . . . ***(Please Use Response Form in Appendix B.)

- ☐[1] ....A little difficulty
- ☐[2] ....Some difficulty
- ☐[3] ....A lot of difficulty
- ☐[0] ....I am unable to do it on my own
- ☐[D] ....Don't Know

***P**6c. Do you usually receive help from another person to walk a quarter of a mile (2-3 blocks)?

- ☐[1] ....Yes **Go to Q7a**
- ☐[0] ....No **Go to Q7a**
- ☐[D] ....Doesn't Do **Go to Q8a**

***P**6d. How easy is it for you to walk for a quarter of a mile (2-3 blocks)? Would you say . . .

- ☐[1] ....Very easy
- ☐[2] ....Somewhat easy
- ☐[3] ....Not that easy
- ☐[D] ....Don't Know

***P**7a. Because of a health or physical problem, do you have any difficulty walking a distance of one mile, that is about 8 to 12 blocks.

- ☐[1] ....Yes **Go to Q8a**
- ☐[0] ....No **Go to Q7b**

***P**7b. How easy is it for you to walk one mile (about 8 to 12 blocks)? Would you say . . .

- ☐[1] ....Very easy
- ☐[2] ....Somewhat easy
- ☐[3] ....Not that easy
- ☐[D] ....Don't Know

***P**8a. Because of a health or physical problem, do you have any difficulty walking up one flight of stairs (about 10 steps) without resting?

- ☐[1] ....Yes
- ☐[0] ....No **Go to Q8d**

P**8b. If yes, how much difficulty would you say you have? Would you say . . . ***(Please Use Response Form in Appendix B.)

- ☐[1] ....A little difficulty
- ☐[2] ....Some difficulty
- ☐[3] ....A lot of difficulty
- ☐[0] ....I am unable to do it on my own
- ☐[D] ....Don't Know

***P**8c. Do you usually receive help from another person to walk up one flight of stairs (about 10 steps)?

- ☐[1] ....Yes **Go to Q9a**
- ☐[0] ....No **Go to Q9a**
- ☐[D] ....Doesn't Do **Go to Q10a**

P**8d. How easy is it for you to walk up one flight of stairs (about 10 steps)? Would you say . . . ***(Please Use Response Form in Appendix B.)

- ☐[1] ....Very easy
- ☐[2] ....Somewhat easy
- ☐[3] ....Not that easy
- ☐[D] ....Don't Know

***P**9a. Because of a health or physical problem, do you have any difficulty walking up two flights of stairs (about 20 steps) without resting?

- ☐[1] ....Yes **Go to Q10a**
- ☐[0] ....No **Go to Q9d**

P**9d. How easy is it for you to walk up two flights of stairs (about 20 steps)? Would you say . . . ***(Please Use Response Form in Appendix B.)

- ☐[1] ....Very easy
- ☐[2] ....Somewhat easy
- ☐[3] ....Not that easy
- ☐[D] ....Don't Know

P10a. Do you have glasses or contact lenses?

- ☐[1] ....Yes
- ☐[0] ....No

P10b. How would you rate your current eyesight (with glasses or contacts, if you wear them)?

- ☐[5] ....Excellent
- ☐[4] ....Good
- ☐[3] ....Fair
- ☐[2] ....Poor
- ☐[1] ....Very Poor
- ☐[0] ....Unable to See/Blind

P11a. Do you wear a hearing aid?

- ☐[1] ....Yes
- ☐[0] ....No

P11b. How would you rate your current hearing ability (with a hearing aid, if used)?

- ☐[5] ....Excellent
- ☐[4] ....Good
- ☐[3] ....Fair
- ☐[2] ....Poor
- ☐[1] ....Very Poor
- ☐[0] ....Unable to Hear/Deaf

---

***For the Interviewer*: These questions (10a, 10b and 10c) are intended to evaluate what the respondent ACTUALLY DOES and not what he/she is able to do.**

P12a. In the past two weeks, did you do any walking (outside of your home)?

☐[1] ....Yes
☐[0] ....No **Go to Q12c**

P12b. On how many days did you go walking in the past two weeks?

☐[5] ....Everyday **Go to Q13a**
☐[4] ....10 to 13 days **Go to Q13a**
☐[3] ....6 to 9 days **Go to Q13a**
☐[2] ....2 to 5 days **Go to Q13a**
☐[1] ....Only one day **Go to Q13a**

12c. What is the main reason you did not do any walking in the past 2 weeks?

☐[1] ....Illness or Injury
☐[2] ....Social-environmental Factors
☐[3] ....Other (Please Specify)________________________

Interviewer: *Now I'm going to ask you about your physical activity and exercise habits when you were around 50 years old . . . [if current age is < 50 years old, substitute [his/her] current age for "age 50"].*

P13a. In a typical week, did you do any regular walking – for exercise, to get to work, while at work, to walk the dog – for at least one hour per week?

☐[1] ....Yes
☐[0] ....No **Go to Q14a**
☐[D] ....Don't Know **Go to Q14a**
☐[R] ....Refused **Go to Q14a**

P13b. Did you do regular walking for at least three hours per week?

☐[1] ....Yes
☐[0] ....No
☐[D] ....Don't Know
☐[R] ....Refused

P14a. In a typical week, did you participate in any vigorous exercise or sports, such as bicycling, swimming, jogging, or racquet sports, for at least one hour per week?

- ☐ 1 ....Yes
- ☐ 0 ....No **Interview Completed**
- ☐ D ....Don't Know **Interview Completed**

P14b. Did you participate in any vigorous exercise or sports for at least three hours per week?

- ☐ 1 ....Yes
- ☐ 0 ....No
- ☐ D ....Don't Know

LONG LIFE FAMILY STUDY	**(Affix Label Here)**<br><br>Participant ID: ___________________<br><br>Participant Name Code: ____________	Date Form Filled Out: ☐☐☐☐☐☐☐☐☐<br>d d M M M y y y y<br>(e.g., 10JUN2005)<br><br>Interviewer Code: ☐☐☐<br><br>Circle Field Center Location:<br>**BU CU DK UP**

Personal History

Please Mark the Appropriate Box Below:

☐1 ....This Form was Administered via a DFR/Proxy
☐2 ....This Form was Administered In-Person by Study Personnel
☐3 ....This Form was Administered via Telephone by Study Personnel
☐4 ....This Form was Mailed and Self-Administered by Participant
☐5 ....This Form was Administered by Other: ____________________

Interviewer: *Now I would like to ask you some questions about any smoking and/or drinking that you have done over the course of your lifetime.*

P1a. Have you smoked more than 100 cigarettes in your entire life?

☐1 ....Yes
☐0 ....No **Go to Q2a**
☐D ....Don't Know
☐R ....Refused

P1b. In what year or how old were you when you started smoking cigarettes on a regular basis?

Year: ___ ___ ___ ___ **OR** Age: ___ ___ ___

P1c. Do you now smoke cigarettes?

☐1 ....Yes **Go to Q1e**
☐0 ....No
☐D ....Don't Know
☐R ....Refused

P1d. In what year or how old were you when you quit smoking cigarettes?

Year: ___ ___ ___ ___ **OR** Age: ___ ___ ___

P1e. On average, how many cigarettes per day *[do you or did you]* usually smoke? ___ ___ ___

P2a. Have you smoked cigars or a pipe on a regular basis?

- ☐[1] ....Yes
- ☐[0] ....No **Go to Q3a**
- ☐[D] ....Don't Know
- ☐[R] ....Refused

P2b. In what year or how old were you when you first started smoking cigars or a pipe?

Year: ___ ___ ___ ___ **OR** Age: ___ ___ ___

P2c. Do you currently smoke cigars or a pipe on a regular basis?

- ☐[1] ....Yes **Go to Q2e**
- ☐[0] ....No
- ☐[D] ....Don't Know
- ☐[R] ....Refused

P2d. In what year or how old were you when you quit smoking cigars or a pipe?

Year: ___ ___ ___ ___ **OR** Age: ___ ___ ___

P2e. On average, how many cigars or pipe bowls per day do/did you smoke? ___ ___ ___

Interviewer: *These next questions are about drinking alcoholic beverages. Alcoholic beverages include beer, ale, wine, wine coolers, liquor such as whiskey, gin, rum or vodka, and cocktails and mixed drinks containing liquor, such as Manhattans and martinis, and any other drink that contains alcohol.*

Please answer for all types of alcoholic beverages together. Let's consider one drink to be equal to . . .

- One 12 oz. can of beer
- One 5 oz. glass of wine (a full glass)
- A drink containing a "shot", a "jigger", or a "finger of liquor" (approximately 1 ¼ oz.)

3a. During the past 12 months, how many drinks did you have in a typical week? If you are unsure, please make your best guess. ***(Please Use Response Form in Appendix B)***

Interviewer Note: **Do NOT read response categories.**

- ☐[1] ....None, do not drink alcohol at all **Go to Q3b**
- ☐[2] ....An occasional drink, but less than one per week **Go to Q3b**
- ☐[3] ....1-3 drinks per week **Go to Q3c**
- ☐[4] ....4-7 drinks per week **Go to Q3c**
- ☐[5] ....8-14 drinks per week **Go to Q3c**
- ☐[6] ....15-21 drinks per week **Go to Q3c**
- ☐[7] ....22-27 drinks per week **Go to Q3c**
- ☐[8] ....28 or more drinks per week **Go to Q3c**
- ☐[D] ....Don't Know **Go to Q3c**
- ☐[R] ....Refused **Go to Q3c**

3b. If the answer to Q3a above was "none" or "an occasional drink":

What is your primary reason for not drinking very much? ***(Please Use Response Form in Appendix B)***

<u>Interviewer Note:</u> **Do NOT read response options. Please check only one answer.**

- ☐[1] ....No need or not necessary
- ☐[2] ....Don't care for it or dislike it
- ☐[3] ....Medical or health reasons
- ☐[4] ....Religious or moral reasons
- ☐[5] ....Recovering alcoholic
- ☐[6] ....Family member an alcoholic or problem drinker
- ☐[7] ....Costs too much
- ☐[8] ....Other Reasons (Please Specify) ____________________________________

3c. Did you ever drink more than you do now?

- ☐[1] ....Yes
- ☐[0] ....No
- ☐[D] ....Don't Know
- ☐[R] ....Refused

3d. Was there ever a time in your life when you drank 5 or more drinks of any kind of alcoholic beverage almost every day?

- ☐[1] ....Yes **Go to Q3e**
- ☐[0] ....No **End Interview**
- ☐[D] ....Don't Know
- ☐[R] ....Refused

3e. <u>If Yes</u>, during the past 12 months, have you had 5 or more drinks almost every day?

- ☐[1] ....Yes
- ☐[0] ....No

LONG LIFE FAMILY STUDY

(Affix Label Here)

Participant ID: ____________________

Participant Name Code: ____________

Date Form Filled Out: ☐☐☐☐☐☐☐☐☐
d d M M M y y y y
(e.g., 10JUN2005)

Interviewer Code: ☐☐☐

Circle Field Center Location:

BU **CU** **DK** **UP**

Medical History

Please Mark the Appropriate Box Below:

- ☐ 1 .... This Form was Administered via a DFR/Proxy
- ☐ 2 .... This Form was Administered In-Person by Study Personnel
- ☐ 3 .... This Form was Administered via Telephone by Study Personnel
- ☐ 4 .... This Form was Mailed and Self-Administered by Participant
- ☐ 5 .... This Form was Administered by Other: ____________________

1. In general, how would you describe your health over the course of your lifetime?

- ☐ 5 .... Excellent
- ☐ 4 .... Very Good
- ☐ 3 .... Good
- ☐ 2 .... Fair
- ☐ 1 .... Poor
- ☐ D .... Don't Know
- ☐ R .... Refused

***P**2. "I'm going to read to you a list of conditions. Please respond 'yes' or 'no' if you have EVER been told by a doctor that you had this condition."

<u>Interviewer:</u> If participant responds "YES", ask at what age they were first told they had the condition and whether or not they currently have the condition, before moving on to next condition. If they don't know if they ever had the condition or refused to answer, please mark the appropriate box. If they don't know the age they were first told, please mark the appropriate box.

Complete Medical History Questions on Page 2.

Participant ID: ____________________________ Participant Name Code: ________________________

	Yes[1]	No[0]	Refused[R]	Don't Know[D]	Age you were first told	Current Condition?
Myocardial Infarction or Heart Attack						**Yes / No**
Coronary Angioplasty or Coronary Artery Bypass Grafting (CABG)						**Yes / No**
Heart Failure or Congestive Heart Failure						**Yes / No**
Atrial Fibrillation/Pacemaker						**Yes / No**
Deep Vein Thrombosis (or blood clots in legs) or Pulmonary Embolism (blood clot in lung)						**Yes / No**
Rheumatic Fever or Heart Valve Problems						**Yes / No**
High Blood Pressure						**Yes / No**
**b. Stroke**						
Stroke or Cerebrovascular Accident						**Yes / No**
Transient Ischemic Attack (TIA) or Mini-Stroke						**Yes / No**
**c. Lung Disease**						
Asthma						**Yes / No**
Chronic Bronchitis						**Yes / No**
Emphysema or Chronic Obstructive Pulmonary Disease (COPD)						**Yes / No**
Pneumonia						**Yes / No**
Pulmonary Fibrosis						**Yes / No**
Chest Surgery<br>If yes, specify: ____________________						**Yes / No**
**d. Arthritis**						
Arthritis of the Knees, Hips or Spine						**Yes / No**
**e. Endocrine/GI/Kidney**						
Diabetes						**Yes / No**
Thyroid Disease						**Yes / No**
Osteoporosis						**Yes / No**
Chronic Liver Disease, Cirrhosis, or Hepatitis						**Yes / No**
Kidney (Renal) Disease or Failure						**Yes / No**
**f. Neurological**						
Alzheimer's Disease or Dementia						**Yes / No**
Parkinson's Disease						**Yes / No**
Depression or Anxiety						**Yes / No**
**g. Cancer**						
Breast Cancer						**Yes / No**
Blood Cancer, Leukemia, or Lymphoma						**Yes / No**
Colon (Bowel) or Rectal Cancer						**Yes / No**

	Yes[1]	No[0]	Refused[R]	Don't Know[D]	Age you were first told	Current Condition?
Lung Cancer						**Yes / No**
Malignant Melanoma						**Yes / No**
Other Skin Cancer						**Yes / No**
Esophageal Cancer						**Yes / No**
Pancreatic Cancer						**Yes / No**
Other Cancer, specify: _____________						**Yes / No**
**For Men Only:**						
Prostate Cancer						**Yes / No**
Enlarged Prostate, not cancer						**Yes / No**
**h. Hearing**						
Use Hearing Aid(s)						**Yes / No**
**i. Vision**						
Cataract Surgery Both Eyes						**Yes / No**
Cataract Surgery One Eye						**Yes / No**
Macular Degeneration						**Yes / No**
Glaucoma						**Yes / No**
**j. Fractures**						
Hip						**Yes / No**
Wrist or Forearm						**Yes / No**
Spine						**Yes / No**
Other: Specify: ______________________						**Yes / No**
**k. Other Illnesses**						
Specify: _____________________________						**Yes / No**
Specify: _____________________________						**Yes / No**
Specify: _____________________________						**Yes / No**
Specify: _____________________________						**Yes / No**
Specify: _____________________________						**Yes / No**

3a. Have you fallen within the last year?

☐ 1 ....Yes
☐ 0 ....No **Go to Q4a**

3b. If yes, how many times? ____ ____

3c. Did any of these falls require medical attention?

☐[1] ....Yes
☐[0] ....No
☐[D] ....Don't Know
☐[R] ....Refused

P4a. Have you been hospitalized within the last year?

☐[1] ....Yes
☐[0] ....No **Go to Q5**
☐[D] ....Don't Know **Go to Q5**
☐[R] ....Refused **Go to Q5**

P4b. How many times have you been hospitalized in the past year? ___ ___

---

5. How much do you currently weigh? If you are unsure, please make your best guess.

___ ___ ___ lbs **OR** ___ ___ ___ kg

6a. Since this time last year, has your weight changed by 5 or more pounds *[or 2.27 or more kilograms]*?

☐[1] ....Yes
☐[0] ....No **Go to Q7**

6b. Did you experience a gain or loss in your weight during this time?

☐[1] ....Gain
☐[2] ....Loss
☐[3] ....Both

6c. Were you trying to *[gain/lose]* weight?

☐[1] ....Yes
☐[0] ....No

6d. How many pounds (or kilograms) did you *[gain/lose]* overall since this time last year?

___ ___ ___ lbs **OR** ___ ___ ___kg

7. What was your usual weight at about age 50? If you don't remember exactly, please make your best guess.

___ ___ ___ lbs **OR** ___ ___ ___ kg

☐D ....Don't Know
☐R ....Refused

Interviewer: The following section is to be asked of women participants only; if participant is a man, conclude the interview at this time.

P8a. Have you ever been pregnant?

☐1 ....Yes
☐0 ....No **Go to Q9**
☐D ....Don't Know **Go to Q9**
☐R ....Refused **Go to Q9**

P8b. How many of your pregnancies resulted in the birth of a live child?

____ ____ pregnancies **If 0, Go to Q9**

P8c. How old were you when your first child was born? Do not include adopted children.

____ ____ years old

P8d. How old were you when your last child was born? Do not include adopted children.

____ ____ years old

P9. How old were you when you first started getting your period? If you are unsure, please make your best guess. ____ ____ years old

P10a. Have you reached menopause?

☐1 ....Yes
☐0 ....No **Go to Q11**
☐D ....Don't Know **Go to Q11**
☐R ....Refused **Go to Q11**

P10b. In what year, or how old were you, when you reached menopause (complete cessation of period for one year)?

Year: ___ ___ ___ ___ **OR** Age: ___ ___ years old **OR**

Interviewer: If unable to provide exact age or year in which menopause began, ask participant to take his/her best guess by choosing one of the categories below for age at which menopause was reached.

Please choose one:
- ☐[1] ....≤ 45 years
- ☐[2] ....46-47 years
- ☐[3] ....48-49 years
- ☐[4] ....50-51 years
- ☐[5] ....≥ 52 years

P10c. Was the onset of your menopause a result of:

- ☐[1] ....Natural Causes
- ☐[2] ....Surgery
- ☐[3] ....Radiation Treatment
- ☐[4] ....Chemotherapy
- ☐[5] ....Other (Please Specify)_________________________

P11. Have you ever had one or both ovaries removed?

- ☐[1] ....Yes
- ☐[0] ....No
- ☐[D] ....Don't Know
- ☐[R] ....Refused

P12a. Have you ever had a hysterectomy (surgery to remove your uterus or womb)?

- ☐[1] ....Yes
- ☐[0] ....No **Go to Q13a**
- ☐[D] ....Don't Know **Go to Q13a**
- ☐[R] ....Refused **Go to Q13a**

P12b. When did you have this surgery?

____ ____ ____ Age OR ___ ___ ___ ___ Year

P12c. Have you taken estrogen or female hormone pills after you had a hysterectomy?

- ☐[1] ....Yes
- ☐[0] ....No **If No, End Interview Here**
- ☐[D] ....Don't Know
- ☐[R] ....Refused

P12d. When did you start taking estrogen or female hormone pills? If you are unsure, please make your best guess.

____ ____ ____Age **OR** ___ ___ ___ ___ Year

P12e. If you took estrogen or female hormone pills, for how many years did you take estrogen or female

hormone pills every day or nearly everyday? If you are unsure, please make your best guess.

____ ____Years **If Answered, End Interview Here**

P13a. Since menopause, have you taken estrogen or female hormone pills?

- ☐[1] ....Yes
- ☐[0] ....No **If No, End Interview Here**
- ☐[D] ....Don't Know
- ☐[R] ....Refused
- ☐[N] ....Not Applicable **If Not Applicable, End Interview Here**

P13b. When did you start taking estrogen or female hormone pills? If you are unsure, please make your best guess.

____ ____ ____ Age **OR** ___ ___ ___ ___ Year

P13c. If you took estrogen or female hormone pills, for how many years did you take estrogen or female hormone pills every day or nearly everyday? If you are unsure, please make your best guess.

____ ____ Years

LONG LIFE FAMILY STUDY

(Affix Label Here)

Participant ID: ____________________

Participant Name Code: ____________

Date Form Filled Out:

☐☐☐☐☐☐☐☐☐

d d M M M y y y y

(e.g., 10JUN2005)

Interviewer Code: ☐☐☐

Circle Field Center Location:

BU **CU** **DK** **UP**

Medication Inventory

Please Mark the Appropriate Box Below:

☐1 ....This Form was Administered via a DFR/Proxy

☐2 ....This Form was Administered In-Person by Study Personnel

☐3 ....This Form was Administered via Telephone by Study Personnel

Section A - Medication Reception

Record on the Medication Inventory Form all prescription and over-the-counter medications (including pills, dermal patches, eye drops, creams, salves, and injections) used in the previous two weeks. If possible, record the complete drug name exactly as written on the container label. Confirm strength and units.

"We are interested in all the prescription and over-the-counter medications that you took during the past 2 weeks. We are also interested in drugs not usually prescribed by a doctor, such as supplements, vitamins, pain medications, laxatives or bowel medicines, cold and cough medications, antacids or stomach medicines, and ointments or salves. Please tell me about any other medications, prescribed by a doctor, that you have not brought with you today."

Did the participant take any prescription or non-prescription medications in the past 2 weeks?

☐1 ....Yes

☐0 ....No

☐D ....Don't Know

☐R ....Refused

Section B - Prescription Medication and/or Over-the-Counter Medications & Supplements

Copy the name of the prescription medication and the strength in milligrams (mg) or other units. Multivitamins and herbal preparations should be coded as "N". In addition, record the formulation code.

***Formulation Codes** - 0=unidentifiable, 1=oral tablet, 2=oral capsule, 3=oral liquid, 4=oral chew, 5=topical cream, lotion, or ointment, 6=other liquid, 7=ophthalmic, 8=rectal or vaginal, 9=inhaled or nasal, 10=injected, 11=transdermal patch, 12=powder, 13=other, D=missing*

Please turn to the Medication Inventory Forms on Pages 2-3

Participant ID: ___________________________ Participant Name Code: ___________________

	Medication Name (Generic Name or Trade Name)	Strength	Units	Formulation Code	Container Seen? Yes or No	Other Notes
1.					Y N	
2.					Y N	
3.					Y N	
4.					Y N	
5.					Y N	
6.					Y N	
7.					Y N	
8.					Y N	
9.					Y N	
10.					Y N	
11.					Y N	
12.					Y N	
13.					Y N	
14.					Y N	
15.					Y N	
16.					Y N	
17.					Y N	
18.					Y N	
19.					Y N	
20.					Y N	
21.					Y N	
22.					Y N	
23.					Y N	
24.					Y N	
25.					Y N	

Participant ID: ____________________________ Participant Name Code: ____________________

	Medication Name (Generic Name or Trade Name)	Strength	Units	Formulation Code	Container Seen? Yes or No	Other Notes
26.					Y N	
27.					Y N	
28.					Y N	
29.					Y N	
30.					Y N	
31.					Y N	
32.					Y N	
33.					Y N	
34.					Y N	
35.					Y N	
36.					Y N	
37.					Y N	
38.					Y N	
39.					Y N	
40.					Y N	
41.					Y N	
42.					Y N	
43.					Y N	
44.					Y N	
45.					Y N	
46.					Y N	
47.					Y N	
48.					Y N	
49.					Y N	

Interviewer: Attach Additional Pages As Needed

NACC
National Alzheimer's Coordinating Center
Department of Epidemiology, School of Public Health and Community Medicine, University of Washington

4311 11th Avenue NE #300
Seattle, WA 98105
phone: (206) 543-8637; fax: (206) 616-5927
e-mail: naccmail@u.washington.edu
website: https://www.alz.washington.edu

NACC Uniform Data Set (UDS)

APPENDIX

(Version 1.1, September 2005)

TABLE of CONTENTS

General Principles of Assessment/Testing 1

Mini Mental State Examination (MMSE) 2

Logical Memory IA – Immediate 8

Digit Span Forward 11

Digit Span Backward 13

Category Fluency 17

Trail Making Test 21

WAIS-R Digit Symbol 27

Logical Memory IIA – Delayed 29

Boston Naming Test (30 odd-numbered items) 31

NACC UDS Neuropsychological Battery

GENERAL PRINCIPLES OF ASSESSMENT/TESTING

This initial section is intended to assist technicians and other non-clinicians to adopt test "etiquette".

1. Establish rapport: This is essential to the interview in general and the assessment of cognitive function in particular. The participants are volunteers who will typically have little experience with cognitive assessment procedures. Thu, the cognitive testing has the potential to be an uncomfortable or even threatening experience. The following considerations will help to establish and maintain rapport.

   a. It is important to convey a genuine sense of appreciation for the time and effort being volunteered. Remember that the participants are people (not sources of data) who are donating their time. It is incumbent upon the interviewer to reciprocate with a style that conveys interest and attentiveness to the interview process, sensitivity to the needs and/or concerns of the respondent, and the appropriate respect for the participant.

   b. The participant will respond to the interviewer's emotional state. If the interviewer is anxious, unsure, disinterested, etc., the participant will often respond in kind. Therefore, it is critical that the examiner is thoroughly trained and confident regarding procedures of test administration. Participants will be maximally at ease and cooperative with an interviewer who is secure in his/her role and who has a firm command of test administration (so that, for example, instructions are delivered in a fluent, practiced manner with proper inflections).

   c. Avoid subtle coaching or "cheerleading". Your job is to administer each item as specified and obtain a response that can be scored.

2. Provide uniformity in data collection (and scoring): This is essential for all portions of the interview. To this end:

   a. Strictly adhere to the wording of instructions and items.

   b. It is vital to gain and hold the participant's attention as items are administered.

   c. Do not assume that part answers or misstatements are correct. When in doubt about the acceptability of a response, refer to the manual and/or your supervisor.

3. Missing data: Every reasonable attempt should be made to administer all performance items/tests. Note that if an item is administered and the correct response is not made, the response should be scored as an error.

   If an item/test is not administered, it may be due to a physical problem (coded as 95 or 995), cognitive/behavioral problem (96 or 996), other problem (97 or 997), or refusal (98 or 998). Select the appropriate reason and enter the code in the space provided. If more than one reason applies, enter the lowest numbered reason.

It is intended that the tests to be administered in the order in which they appear in the NACC UDS manual. This is necessary in order to standardize among Centers the delay intervals for testing memory, and also to eliminate any differences due to the order of test administration. It is therefore suggested that the UDS be administered in its entirety either before or after the administration of other tests commonly used by the Center.

NACC UDS Neuropsychological Battery

MINI MENTAL STATE EXAMINATION (MMSE)

Description:
The Mini Mental State Examination is a screening scale that evaluates orientation to place, orientation to time, registration (immediate repetition of three words), attention and concentration (spelling D-L-R-O-W), recall (recalling the previously repeated three words), language (naming, repetition, reading, writing, comprehension), and visual construction (copy two intersecting pentagons). The MMSE is scored as the number of correctly completed items, with lower scores indicative of poorer performance and greater cognitive impairment.

Source:
Reproduced by special permission of the publisher, Psychological Assessment Resources, Inc., 16204 North Florida Avenue, Lutz, FL 33549, from the Mini Mental State Examination by Marshal Folstein and Susan Folstein, copyright 1975, 1998, 2001 by Mini Mental LLC, Inc. Published 2001 by Psychological Assessment Resources, Inc. Further reproduction is prohibited without permission of PAR, Inc. The MMSE can be purchased from PAR, Inc. by calling (813) 968-3003.

Administration:
Words in boldface type should be read aloud clearly and slowly to the examinee. Item substitutions appear in parentheses. Administration should be conducted privately and in the examinee's primary language. Begin by asking the following two questions:

[SAY]: "Do you have any trouble with your memory? May I ask you some questions about your memory?"

[Follow the detailed instructions (*Individual Item Instructions* below) for each item on the MMSE.]

Scoring:
Scoring is deferred until after the examination, and is done from the written record, which must be recorded in a legible and decipherable manner. On the *Worksheet for Mini Mental State Examination (MMSE)* (master form provided in the tabbed section entitled "UDS Npsych Test Forms"), circle '0' for an incorrect response, or '1' if a response is correct. On NACC UDS Form C1, in the space provided for item 1b.1, enter the subscale score for Orientation to Time (0–5 points). In the space for item 1b.2, enter the subscale score for Orientation to Place (0–5 points). Then sum all item scores on the worksheet and enter that total score (0-30) in the space provided for item 1c.

Individual Item Instructions:
<u>Orientation to Time</u>:

Each question should be asked separately, as specified on the test form.

If the subject gives only the last two digits of the year, prompt by saying, "What is the full year?" When a year or season is near transition, the correct answer can be prompted with "Are you sure?" If the subject gives the date when prompted for the day, give credit for date if the response is correct. All other items require exact answers.

Write the subject's response to each question in the space provided. Score one point for each correct response. No partial credit is given.

Orientation to Place:

Each question should be asked separately, as indicated on the test form.

For "building", any correct name is acceptable except generic references such as 'hospital' or 'medical center'. All other items require exact answers.

Write the subject's response to each question in the space provided. Score one point for each correct response.

Registration:

[SAY]: "Listen carefully. I am going to say three words. You say them back after I stop. Ready? Here they are...APPLE [pause], PENNY [pause], TABLE [pause]. Now repeat those words back to me." Repeat up to 5 times, but score only the first trial.

Write the subject's response to each question in the space provided. At the end of the trial, give the following instructions:

[SAY]: "Now keep those words in mind. I am going to ask you to say them again in a few minutes."

Write the subject's response to each question in the space provided. Score one point for each correct response.

Note: The *Recall* test below should be administered no sooner than three minutes following this test. If the intervening test, *Attention and Concentration*, is completed in less than three minutes, perform a non-verbal portion of the MMSE (e.g., *Comprehension*, *Reading*, *Writing*, or *Drawing*) until three minutes have elapsed and then return to the *Recall* test.

Attention:

[SAY]: "The word WORLD is spelled W-O-R-L-D. Spell WORLD backwards."

Repeat the instructions if the subject asks for a repetition or appears confused. As clarification, the examiner may say "Start from the end and go to the beginning." Allow additional trials if the subject requests them.

Record the letters of the subject's final response. One point is given for each correct letter (for example, "D-L-O-W" would receive two points for the correct placement of the "D" and the "L").

Recall:

[**Note:** This test should be administered no sooner than three minutes after the *Registration* test above. If the previous test, *Attention*, is completed in less than three minutes, perform a non-verbal portion of the MMSE (e.g., *Comprehension*, *Reading*, *Writing*, or *Drawing*) until three minutes have elapsed and then return to this test.]

After a 3-minute delay following *Registration*, ask the subject:

[SAY]: "What were those three objects I asked you to remember?"

If needed, explain the source of the three objects by saying "I asked you to repeat three words earlier and to remember them. Can you recall them now?"

Write the subject's response to each question in the space provided. One point is given for each correct response. If the subject is unable to recall an item, the item should be scored as incorrect.

Naming:

Show the subject a pencil or pen and say, "What is this?" If the subject gives a function (e.g., "You can write with it"), say "Yes, but what is this called?" No other clues should be provided. Repeat for wristwatch.

Write the subject's response to each question in the space provided. One point is given for each correct response.

Repetition:

Engage the subject's attention and clearly say, "Now I am going to ask you to repeat what I say. Ready? 'No ifs, ands, or buts.' Now you say that." Repeat up to 5 times, but score only the first trial.

Write the subject's response in the space provided. One point is given for an exact repetition of the phrase.

Use the 3-segment page for the remaining items (cut the page along the dotted lines). Use the upper portion of the page (blank) for the *Comprehension*, *Writing*, and *Drawing* items that follow. Use the middle portion of the page ("CLOSE YOUR EYES") as a stimulus form for the *Reading* item. Use the lower portion (intersecting pentagons) for the *Drawing* item.

Comprehension:

[SAY]: "Listen carefully because I am going to ask you to do something." Present the blank piece of paper at the subject's mid-line and say "Take the paper in your right hand, fold it in half and put it on the floor [or table]". One repetition is permitted at the subject's request, but the entire command must be repeated verbatim. The subject is allowed to fold the paper using both hands. Record the subject's performance for each of the three segments indicated on the form.

Reading:

Show the stimulus card ("CLOSE YOUR EYES") to the subject and say "Read this and do what it says." The subject can be reminded not just to read the sentence, but to perform the action. If the subject is unable to read, read the sentence out loud.

Record the subject's performance. One point is given if the instructions are completely and correctly followed.

Writing:

Give the subject a pencil or pen and the blank piece of paper and say "Write a sentence." If needed, prompt the subject by saying "Write a sentence about the weather."

One point is given for any complete sentence (even if not about the weather). Grammar, spelling, and punctuation are not scored, but the subject must write a sensible sentence that contains a subject and a verb. A sentence with an implied subject (e.g., close the door) is acceptable. If the sentence is illegible, ask the subject to read it aloud for scoring.

Drawing:

Give the subject a pencil or pen and the paper with the overlapping pentagons and say "Please copy this design." Do not allow erasures. If the subject appears dissatisfied with his/her drawing or requests a second attempt, allow the subject to re-draw the figures. Clearly label the first and second attempt. The best drawing should be scored.

One point is given if the subject draws two intersecting pentagons (5-sided figures), the intersection results in a 4-sided figure, and all ten angles are present with two of them intersecting.

Worksheet for MINI-MENTAL STATE EXAMINATION (MMSE)

SAMPLE (actual form at back of manual)

Instructions: Words in boldface type should be read aloud clearly and slowly to the examinee. Item substitutions appear in parentheses. Administration should be conducted privately and in the examinee's primary language. Circle "0" if the response is incorrect, or "1" if the response is correct. Begin by asking the following two questions:
Do you have any trouble with your memory? May I ask you some questions about your memory?

ORIENTATION TO TIME	RESPONSE	SCORE (circle one)	
**What is the... year?**	______	0	1
**season?**	______	0	1
**month of the year?**	______	0	1
**day of the week?**	______	0	1
**date?**	______	0	1

ORIENTATION TO PLACE
(Alternative place words that are appropriate for the setting and increasingly precise may be substituted and noted.)
Where are we now? What is the...

**state** (province)?	______	0	1
**county** (city/town)?	______	0	1
**city/town** (or part of city/neighborhood)?	______	0	1
**building** (name or type)?	______	0	1
**floor of the building**(room number or address)?	______	0	1

REGISTRATION
(Alternative word sets [e.g., PONY, QUARTER, ORANGE] may be substituted and noted when retesting an examinee.)
Listen carefully. I am going to say three words. You say them back after I stop. Ready?
Here they are... APPLE [pause], **PENNY** [pause], **TABLE** [pause]. **Now repeat those words back to me.**
[*Repeat up to 5 times, but score only the first trial.*]

APPLE	______	0	1
PENNY	______	0	1
TABLE	______	0	1

Now keep those words in mind. I am going to ask you to say them again in a few minutes.

ATTENTION AND CONCENTRATION
The word WORLD is spelled W-O-R-L-D.
Spell WORLD backwards.
[*Allow additional trials if requested.*] ____ (D=1) ____ (L=1) ____ (R=1) ____ (O=1) ____ (W=1) ____ (0 to 5)

(continued) →

Psychological Assessment Resources, Inc. • 16204 N. Florida Avenue • Lutz, FL 33549 • (800) 331-8378 • www.parinc.com

Reproduced by special permission of the publisher, Psychological Assessment Resources, Inc., 16204 North Florida Avenue, Lutz, FL 33549, from the Mini Mental State Examination by Marshal Folstein and Susan Folstein, copyright 1975, 1998, 2001 by Mini Mental LLC, Inc. Published 2001 by Psychological Assessment Resources, Inc. Further reproduction is prohibited without permission of PAR, Inc. The MMSE can be purchased from PAR, Inc. by calling (813) 968-3003.

SAMPLE (actual form at back of manual)

**RECALL**			
**What were those words I asked you to remember?** [*Do not offer any hints.*]			
APPLE	__________	0	1
PENNY	__________	0	1
TABLE	__________	0	1
**NAMING**	RESPONSE	SCORE (circle one)	
**What is this?** [*Point to a pencil or pen.*]	__________	0	1
**What is this?** [*Point to a watch.*]	__________	0	1
(Alternative common objects [e.g., eyeglasses, chair, keys] may be substituted and noted.)			
**REPETITION**			
**Now I am going to ask you to repeat what I say. Ready? "NO IFS, ANDS, OR BUTS." Now you say that.** [*Repeat up to 5 times, but score only the first trial.*]			
NO IFS, ANDS, OR BUTS	__________	0	1

Use the following 3-segment page for the remaining items (cut the page along the dotted lines). Use the upper portion of the page (blank) for the *Comprehension*, *Writing*, and *Drawing* items that follow. Use the middle portion of the page ("CLOSE YOUR EYES") as a stimulus form for the *Reading* item. Use the lower portion (intersecting pentagons) for the *Drawing* item.

**COMPREHENSION**			
**Listen carefully because I am going to ask you to do something.** **Take this paper in your right hand** [pause], **fold it in half** [pause], **and put it on the floor** (*or* **table).**			
TAKE IN RIGHT HAND	__________	0	1
FOLD IN HALF	__________	0	1
PUT ON FLOOR (*or* TABLE)	__________	0	1
**READING**			
**Please read this and do what it says.** [Show examinee the words on the stimulus form.].			
CLOSE YOUR EYES	__________	0	1
**WRITING**			
(Place the blank piece of paper (unfolded) in front of the subject and provide a pen or pencil.)			
**Please write a sentence.** [If examine does not respond, say: **Write about the weather.**]		0	1
Score 1 point if the sentence is comprehensible and contains a subject and a verb. Ignore errors in grammar or spelling.			
**DRAWING**			
**Please copy this design.** [Display the intersecting pentagons on the stimulus form.]		0	1
Score 1 point if the drawing consists of two 5-sided figures that intersect to form a 4-sided figure.			

Assessment of level of consciousness.

Alert/ Responsive	Drowsy	Stuporous	Comatose/ Unresponsive

Total Score = ________
(Sum all item scores) (30 points max.)

SAMPLE (actual form at back of manual)

CLOSE YOUR EYES

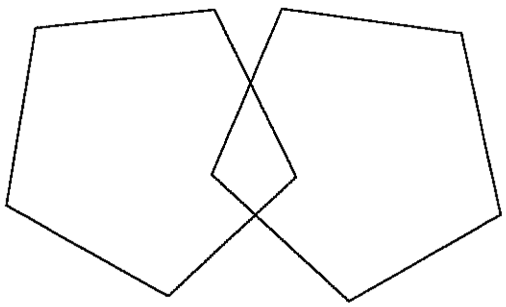

NACC UDS Neuropsychological Battery

LOGICAL MEMORY IA – IMMEDIATE

Description:
Logical Memory is a subtest of the Wechsler Memory Scale-Revised and among the most widely used clinical measures of memory. For UDS purposes, only the first (story A) of the two stories will be administered. This test assesses the ability to recall a short passage or story. The examiner reads the story to the subject in a clear voice. Immediately after hearing the story, the subject is asked to retell the story from memory. The story should be read with adequate volume and clarity for the subject to understand during the presentation. No repetitions are permitted.

It is important for the examiner to get a sense of the subject's hearing acuity, and modulate their voice accordingly. Of note, for the hard of hearing, it is not necessarily helpful to merely increase the volume, but rather change the pitch; a lower pitched voice sometimes is more audible than a loud, high-pitched voice. As with all neuropsychological testing, it is best if the examiner projects his/her voice at the subject, rather than down toward their clipboard.

Source:
Wechsler Memory Scale® – Revised. Copyright© 1945, renewed 1974, 1987 by Harcourt Assessment, Inc. Reproduced with permission. All rights reserved. *"Wechsler Memory Scale"* and *"WMS"* are trademarks of Harcourt Assessment, Inc., registered in the United States of America and other jurisdictions.

Administration:
The following instructions are printed on each worksheet and are to be read verbatim:

[SAY]: "I am going to read you a little story of just a few lines. Listen carefully and try to remember it just the way I say it, as close to the same words as you can remember. When I am through, I want you to tell me everything I read to you. You should tell me all you can remember, even if you are not sure. Are you ready?"

When the examiner has finished reading the story:

[SAY]: "Now what did I read to you? Tell me everything and begin at the beginning."

Always permit the subject to include additional information by prompting with "Anything else?" Record any additional "bits of information". After the subject appears to be able to recall no more of the story:

[SAY] "Later on I will ask you to tell me this story again, so try not to forget it."

Logical Memory IIA–Delayed should be administered at least 30 minutes, and no more than 40 minutes, after this test. The examiner should complete other cognitive testing during the interval between the Immediate and Delayed tests. **Note: if the 30–40 minute delay period has elapsed and another test is being administered, interrupt that test and administer the *Logical Memory IIA-Delayed.* Once the Logical Memory II has been fully completed, resume the interrupted test.**

Record the subject's responses on the *Worksheet for Logical Memory IA–Immediate* (master form provided in the tabbed section entitled "UDS Npsych Test Forms") between the lines of the text. To simplify the process of recording, underline each word or unit that is reported verbatim, and write above the text any units that are reported but not verbatim. The examiner may prefer to tape record the subject's response and then transcribe the results after the session; this is acceptable if appropriate consent has been obtained.

Scoring:
Scoring is deferred until after the examination. The phrases and words separated by diagonal lines in the passages are treated as items or units, and each unit correctly repeated is worth one point, for a total of 25 points. Non-verbatim responses that are acceptable and receive full credit are listed in the Weschler Memory Scale-Revised manual.

Record on the *Worksheet for Logical Memory IA–Immediate* (master form provided in the tabbed section entitled "UDS Npsych Test Forms") each unit that is successfully recalled. Then total the units recalled and enter that score (00-25) in the space provided. This is the number to be entered on NACC UDS Form C1, item 3c.

Worksheet for LOGICAL MEMORY IA – IMMEDIATE

SAMPLE (actual form at back of manual)

Read aloud the follow instructions verbatim:

[SAY]: "I am going to read you a little story of just a few lines. Listen carefully and try to remember it just the way I say it, as close to the same words as you can remember. When I am through, I want you to tell me everything I read to you. You should tell me all you can remember, even if you are not sure. Are you ready?"

[Record the subject's responses between the lines of the text below. To simplify the process of recording, underline each word or unit that is reported verbatim, and write above the text any units that are reported but not verbatim. The examiner may prefer to tape record the subject's response and then transcribe the results after the session; this is acceptable if appropriate consent has been obtained.]

**Story A – Immediate**	**Score**
Anna / Thompson / of South / Boston /, employed / as a cook /	___ (0–6)
in a school / cafeteria /, reported / at the City Hall /Station /	___ (0–5)
that she had been held up / on State Street / the night before /	___ (0–3)
and robbed / of fifty-six dollars /. She had four /	___ (0–3)
small children /, the rent was due /, and they had not eaten /	___ (0–3)
for two days /. The police /, touched by the woman's story /,	___ (0–3)
took up a collection / for her /.	___ (0–2)

Total number of story units recalled: ___ ___

[SAY]: "Now what did I read to you? Tell me everything and begin at the beginning."

Always permit the subject to include additional information by prompting with "Anything else?" Record any additional "bits of information". After the subject appears to be able to recall no more of the story:

[SAY] "Later on I will ask you to tell me this story again, so try not to forget it."

Logical Memory IIA–Delayed should be administered at least 30 minutes, and no more than 40 minutes, after this test. The examiner should complete other cognitive testing during the interval between the Immediate and Delayed tests. **Note: if the 30–40 minute delay period has elapsed and another test is being administered, interrupt that test and administer the *Logical Memory IIA-Delayed.***

NACC UDS Neuropsychological Battery

DIGIT SPAN FORWARD

Description:
This is a widely-used test of working memory (or attention) in which the subject is read number sequences of increasing length and asked to repeat them. The total score is the number of sequences correctly repeated.

Source:
Wechsler Memory Scale® – Revised. Copyright© 1945, renewed 1974, 1987 by Harcourt Assessment, Inc. Reproduced with permission. All rights reserved. *"Wechsler Memory Scale"* and *"WMS"* are trademarks of Harcourt Assessment, Inc., registered in the United States of America and other jurisdictions.

Administration:
Begin with item 1a and read aloud the following instructions:

[SAY]: "I am going to say some numbers. Listen carefully, and when I am through, say them right after me. Ready?"

Read digits clearly at the rate of one per second, letting voice pitch drop on last digit. If the subject begins repeating the numbers before you've said them all, say "I'll be saying several numbers. I'd like you to wait until I've finished saying them all before you respond." Record the subject's response verbatim, then proceed to the next item.

Subsequent items are preceded by "Here is another." Again, read digits clearly at the rate of one per second, letting voice pitch drop on last digit. On each item, record the subject's response verbatim.

Each digit sequence is presented only once. If the subject asks for a repetition, say, "Just tell me what you can remember". The examiner may <u>not</u> repeat a number sequence for the subject. If the subject cannot respond, say "Let's try another one." Spontaneous changes of the response are permitted. The procedure is discontinued after two consecutive errors at the same item length.

Scoring is deferred until after the examination and is done from the written record, which must be written in a legible, decipherable manner.

Scoring:
Each response is scored as an error (0) or correct (1) in the space provided on the *Worksheet for Digit Span Forward* (master form provided in the tabbed section entitled "UDS Npsych Test Forms"). The Total Correct score is the number of correct responses prior to two consecutive errors at the same digit length. This is the number to be entered on NACC UDS Form C1, item 4a.

The Digit Span Forward Length is the length of the highest digit sequence the subject was able to repeat correctly. This number should be entered on NACC UDS Form C1, item 4b.

Worksheet for DIGIT SPAN FORWARD

SAMPLE (actual form at back of manual)

Read aloud the follow instructions verbatim:

[SAY]: "I am going to say some numbers. Listen carefully, and when I am through, say them right after me. Ready?"

Read digits clearly at the rate of one per second, letting voice pitch drop on last digit. If the subject begins repeating the numbers before you've said them all, say "I'll be saying several numbers. I'd like you to wait until I've finished saying them all before you respond." Record the subject's response verbatim on the worksheet below, then proceed to the next item.

Subsequent items are preceded by "Here is another." Again, read digits clearly at the rate of one per second, letting voice pitch drop on last digit. On each item, record the subject's response verbatim.

Each digit sequence is presented only once. If the subject asks for a repetition, say, "Just tell me what you can remember". The examiner may not repeat a number sequence for the subject. If the subject cannot respond, say "Let's try another one." Spontaneous changes of the response are permitted. The procedure is discontinued after two consecutive errors at the same item length.

Item	Response Code (0 or 1)
1a. "Ready? 6-2-9."	___
1b. "Here is another. 3-7-5."	___
2a. "Here is another. 5-4-7-1"	___
2b. "Here is another. 8-3-9-6."	___
3a. "Here is another. 3-6-9-2-5."	___
3b. "Here is another. 6-9-4-7-1."	___
4a. "Here is another. 9-1-8-4-2-7."	___
4b. "Here is another. 6-3-5-4-8-2."	___
5a. "Here is another: 1-2-8-5-3-4-6."	___
5b. "Here is another: 2-8-1-4-9-7-5."	___
6a. "Here is another: 3-8-2-9-5-1-7-4."	___
6b. "Here is another: 5-9-1-8-2-6-4-7."	___

Total Correct: ___

Digit Span Forward Length: ___

NACC UDS Neuropsychological Battery

DIGIT SPAN BACKWARD

Description:
This is a widely used measure of working memory (or attention) in which the subject is read number sequences of increasing length and then asked to repeat each sequence backward. The primary measure of performance is the number of digit sequences correctly reversed.

Source:
Wechsler Memory Scale® – Revised. Copyright© 1945, renewed 1974, 1987 by Harcourt Assessment, Inc. Reproduced with permission. All rights reserved. *"Wechsler Memory Scale"* and *"WMS"* are trademarks of Harcourt Assessment, Inc., registered in the United States of America and other jurisdictions.

Administration:
This task immediately follows Digit Span Forward and begins with practice to orient the subject to the task. Read aloud the initial instruction on the *Worksheet for Digit Span Backward* (master form provided in the tabbed section entitled "UDS Npsych Test Forms"):

[SAY]: "I am going to say some more numbers. When I stop, I want you to say them backward. Ready?"

Present practice item P1 by saying "Try this one: 2-8-3."

Pause for the subject to respond, and encourage a guess if need be. Record the response code (0 = error; 1 = correct) for P1 in the space provided on the worksheet.

If the subject is <u>correct</u>, read the appropriate instruction branch:

[SAY]: "That's right. Now I have some more numbers. Remember, you are to say them backward." Proceed to the first test item (1a).

If the subject is <u>incorrect</u>, read the appropriate instruction branch:

[SAY]: "No, I said 2-8-3, so to say these backward, you would need to say 3-8-2." Now try these numbers; remember, you are to say them backward. Ready?" Administer the next practice item (P2), record the response code on the worksheet. Whether the subject passes or fails practice item 2, proceed to the first test item (1a).

[SAY]: "I am going to say some more numbers When I stop, I want you to say them backwards.

Make sure you have the subject's attention before presenting each item. Do this by saying "Ready?" before each item. Subsequent items are preceded by "Here is another." Read digits clearly at the rate of one per second, letting voice pitch drop on last digit. Each digit sequence is presented only once. If the subject asks for a repetition, say "Just tell me what you can remember."

Spontaneous changes of the response are permitted. The procedure is discontinued after two consecutive errors <u>at the same item length</u>. If the subject asks for reinstruction, say, "After I say the numbers, you try to say them backward."

If the subject repeats the numbers forward on either of the first two test trials, you may cue them to say the numbers in the backward order, but do not repeat the number sequence. Reinstruct the subject by saying "Remember, after I say the numbers, you are to say them backward. Ready?" Score as correct if the subject is then able to give the correct backward order. Only one unrequested reinstruction is permitted, so subsequent forward orders are simply scored as errors. Do not cue for any of the remaining backward sequences.

Scoring:
Each response is scored as an error (0) or correct (1) in the space provided on the *Worksheet for Digit Span Backward*. The Total Correct score is the number of correct responses prior to two consecutive errors at the same digit length. This is the number to be entered on NACC UDS Form C1, item 5a.

The Digit Span Backward Length is the highest digit sequence the subject was able to reverse. This number should be entered on NACC UDS Form C1, item 5b.

Worksheet for DIGIT SPAN BACKWARD

SAMPLE (actual form at back of manual)

This task immediately follows Digit Span Forward and begins with practice to orient the subject to the task. Read aloud the initial instruction:

[SAY]: "I am going to say some more numbers. When I stop, I want you to say them backward. Ready?"

Present practice item P1 on the worksheet below by saying **"Try this one: 2-8-3."** Pause for the subject to respond, and encourage a guess if need be. Record the response code (0 = error; 1 = correct) for P1 in the space provided on the worksheet.

Practice Items	Response Code (0 or 1)	Instruction
P1. "Try this one: 2-8-3."	___	[If 3-8-2]: "That's right. Now I have some more numbers. Remember, you are to say them backward." [Go to test item 1a]<br>[If any other response]: "No, I said 2-8-3, so to say these backward, you would need to say 3-8-2. Now try these numbers; remember, you are to say them backward. [Go to practice item P2]
P2. "Ready? 1-5-8."	___	[If 8-5-1]: "That's right." [Go to test item 1a]<br>[If any other response]: "No, I said 1-5-8, so to say them backward, you would need to say 8-5-1." [Go to test item 1a].

[SAY]: "I am going to say some more numbers When I stop, I want you to say them backward."

Test Items	Response Code (0 or 1)
1a. "Ready? 5-1"	___
1b. "Here is another: 3-8."	___
2a. "Here is another: 4-9-3."	___
2b. "Here is another: 5-2-6."	___
3a. "Here is another: 3-8-1-4."	___
3b. "Here is another: 1-7-9-5."	___

(continued)

Worksheet for DIGIT SPAN BACKWARD
(continued)

SAMPLE (actual form at back of manual)

4a.	"Here is another: 6-2-9-7-2."	___
4b.	"Here is another: 4-8-5-2-7."	___
5a.	"Here is another: 7-1-5-2-8-6."	___
5b.	"Here is another: 8-3-1-9-6-4."	___
6a.	"Here is another: 4-7-3-9-1-2-8."	___
6b.	"Here is another: 8-1-2-9-3-6-5."	___

Total Correct: ___

Digit Span Backward Length: ___

NACC UDS Neuropsychological Battery

CATEGORY FLUENCY

Description:
This is a widely used measure of semantic memory (verbal fluency, language). The subject is asked to name different exemplars of a given semantic category, and the number of unique exemplars named is scored.

Source:
The procedure is adapted from CERAD administration and scoring procedures for Verbal Fluency (Morris et al., 1989).

Administration:
Read the initial instruction:

[SAY]: "I am going to give you a category and I want you to name, as fast as you can, all of the things that belong in that category. For example, if I say 'articles of clothing', you could say 'shirt', 'tie', or 'hat'. Can you think of other articles of clothing?"

Allow up to 20 seconds for the subject to produce two responses. Circle the number corresponding to the subject's responses, and read the associated instruction.

Response Code	Instruction
0 (No response)	"You could have said 'shoes' or 'coat' since they are articles of clothing."
1 (One or more incorrect responses, no correct response)	"No, ____is (are) not an article (s) of clothing. You could have said 'shoes' or 'coat' since they are articles of clothing."
2 (One or more correct response, no incorrect responses)	"That's right. You also could have said 'shoes' or 'coat'."
3 (One or more correct responses, one or more incorrect responses)	"______is (are) correct, but _______ is (are) not an article of clothing. You also could have said 'shoes' or 'coat'."
4 (Two or more correct responses)	"That's right."

Next, read the instructions for the Animals category:

[SAY] "Now I want you to name things that belong to another category: Animals. You will have one minute. I want you to tell me all the animals you can think of in one minute. Ready? Begin."

Start timer as you say "Begin". Write actual responses as legibly as possible on the *Worksheet for Category Fluency–Animals* (master form provided in the tabbed section entitled "UDS Npsych Test Forms"). Stop the procedure at 60 seconds. One prompt ("Tell me all the animals you can think of.") is permitted if the participant makes no response for 15 seconds or expresses incapacity (e.g., "I can't think of any more."). It is also permissible to repeat the instruction or category if the subject specifically requests it.

Next, read the instructions for the Vegetables category:

[SAY] "Now I want you to name things that belong to another category: Vegetables. You will have one minute. I want you to tell me all the vegetables you can think of in one minute. Ready? Begin."

Start timer as you say "Begin". Write actual responses as legibly as possible on the *Worksheet for Category Fluency–Vegetables* (master form provided in the tabbed section entitled "UDS Npsych Test Forms"). Stop the procedure at 60 seconds. One prompt ("Tell me all the vegetables you can think of.") is permitted if the participant makes no response for 15 seconds or expresses incapacity (e.g., "I can't think of any more."). It is also permissible to repeat the instruction or category if the participant specifically requests it.

Scoring:
Defer scoring until after all test administration is finished.

The Animal Total score on the *Worksheet for Category Fluency–Animals* is the number of correct unique animal names produced within the one-minute time limit.

- CREDIT: breeds (e.g., terriers); male, female, and infant names of a species (e.g., bull, cow, calf); both superordinate and subordinate examples of a species (e.g., both dog and terrier are credited); birds; fish; reptiles, insects.
- DO NOT CREDIT: Repetitions, mythical animals.

The Animal Total score (0–77) should be entered on NACC UDS Form C1, item 6a.

The Vegetable Total score on the *Worksheet for Category Fluency–Vegetables* is the total number of correct unique names of vegetables produced within the one-minute time limit.

- CREDIT: Both superordinate and subordinate responses (e.g., peppers and jalapenos are credited); less specific names (e.g., greens); nuts (e.g., peanuts, acorns); and grains such as corn or rice.
  - ⇨ Names of vegetables found in other cultures but perhaps unfamiliar to you (e.g., jicama) are acceptable only if they can be verified in the dictionary. After completion of the task, ask the subject to spell the word if you are unsure of the correct spelling.
  - ⇨ Grains (e.g., rice, wheat, oats, etc.), gourds, sugarcane, herbs and seaweed are counted as acceptable 'vegetable' responses.
  - ⇨ Tomato, avocado, and pumpkin are acceptable responses.
  - ⇨ Prepared vegetable products are not acceptable responses (e.g., pickles, tomato sauce, ketchup, etc.)
- DO NOT CREDIT: Repetitions.

The Vegetable Total score (0–77) should be entered on NACC UDS Form C1, item 6b.

Worksheet for CATEGORY FLUENCY–ANIMALS

SAMPLE (actual form at back of manual)

1. ____________________
2. ____________________
3. ____________________
4. ____________________
5. ____________________
6. ____________________
7. ____________________
8. ____________________
9. ____________________
10. ____________________
11. ____________________
12. ____________________
13. ____________________
14. ____________________
15. ____________________
16. ____________________
17. ____________________
18. ____________________
19. ____________________
20. ____________________
21. ____________________
22. ____________________
23. ____________________
24. ____________________
25. ____________________
26. ____________________
27. ____________________
28. ____________________
29. ____________________
30. ____________________
31. ____________________
32. ____________________
33. ____________________
34. ____________________
35. ____________________
36. ____________________
37. ____________________
38. ____________________
39. ____________________
40. ____________________
41. ____________________
42. ____________________
43. ____________________
44. ____________________
45. ____________________
46. ____________________
47. ____________________
48. ____________________
49. ____________________
50. ____________________
51. ____________________
52. ____________________
53. ____________________
54. ____________________
55. ____________________
56. ____________________
57. ____________________
58. ____________________
59. ____________________
60. ____________________
61. ____________________
62. ____________________
63. ____________________
64. ____________________
65. ____________________
66. ____________________
67. ____________________
68. ____________________
69. ____________________
70. ____________________
71. ____________________
72. ____________________
73. ____________________
74. ____________________
75. ____________________
76. ____________________
77. ____________________

Animal Total _____

Worksheet for CATEGORY FLUENCY–VEGETABLES

SAMPLE (actual form at back of manual)

1. ______	27. ______	53. ______
2. ______	28. ______	54. ______
3. ______	29. ______	55. ______
4. ______	30. ______	56. ______
5. ______	31. ______	57. ______
6. ______	32. ______	58. ______
7. ______	33. ______	59. ______
8. ______	34. ______	60. ______
9. ______	35. ______	61. ______
10. ______	36. ______	62. ______
11. ______	37. ______	63. ______
12. ______	38. ______	64. ______
13. ______	39. ______	65. ______
14. ______	40. ______	66. ______
15. ______	41. ______	67. ______
16. ______	42. ______	68. ______
17. ______	43. ______	69. ______
18. ______	44. ______	70. ______
19. ______	45. ______	71. ______
20. ______	46. ______	72. ______
21. ______	47. ______	73. ______
22. ______	48. ______	74. ______
23. ______	49. ______	75. ______
24. ______	50. ______	76. ______
25. ______	51. ______	77. ______
26. ______	52. ______	**Vegetable Total** ______

NACC UDS Neuropsychological Battery

TRAIL MAKING TEST

Description:

This is a test of processing speed and executive function. Although both Parts A and B depend on visuomotor and perceptual-scanning skills, Part B also requires considerable cognitive flexibility in shifting from number to letter sets under time pressure.

Part A consists of 25 circles numbered 1 through 25 distributed over a white sheet of 8½" x 11" paper. The subject is instructed to connect the circles with a drawn line as quickly as possible in ascending numerical order.

Part B also consists of 25 circles, but these circles contain either numbers (1 through 13) or letters (A through L). The subject must connect the circles while alternating between numbers and letters in an ascending order (e.g., A to 1; 1 to B; B to 2; 2 to C).

The subject's performance is judged in terms of the time, in seconds, required to complete each Trail. The time to complete Part A (150-second maximum) and Part B (300-second maximum) will be the primary measure of interest (testing is stopped if the maximum time is reached). Both parts of the Trail Making Test are available in multiple forms of equal difficulty for purposes of repeated evaluation.

Source:

This test, originally called Partington's Pathways[1], was a component of the Army Individual Test Battery. It was popularized by Reitan and colleagues[2] and subsequently became part of the Halstead-Reitan Neuropsychological Battery[3]. The instructions for administration are adapted from Spreen and Strauss[4].

Administration:

Part A: Place the form for Sample A in front of the subject (master form provided in the tabbed section entitled "UDS Npsych Test Forms"). Read aloud the instructions:

[SAY]: "There are numbers in circles on this page. Please take the pencil and draw a line from one number to the next, in order. Start at 1 (point to the number), then go to 2 (point to the number), then go to 3 (point to the number) and so on. Please try not to lift the pencil as you move from one number to the next. Work as quickly as you can."

If the subject makes an error, mark through the line and go back to the point at which the error was made and say, for example, "You were at number two. What is the next number?" Wait for the subject's response and say "Please start here and continue."

If the subject completes the sample correctly, go to Test A (master form provided in the tabbed section entitled "UDS Npsych Test Forms"). Repeat the instructions given for the sample. Start timing as soon as the instruction is given to begin. Stop timing when the Trail is completed, or stop subject when the maximum time is reached. Allow a maximum of 150 seconds for the test.

---

[1] Partington JE, Leiter RG. Partington's Pathway Test. The Psychological Service Center Bulletin. 1949;1:9-20.

[2] Reitan RM. Validity of the Trail-Making Test as an indication of organic brain damage. Perceptual Motor Skills. 1958;8:271-276.

[3] Reitan R, Wolfson D. The Halstead-Reitan Neuropsychological Test Battery. Tucson: Neuropsychology Press; 1985.

[4] Spreen O, Strauss E. A compendium of neuropsychological tests. New York: Oxford University Press; 1998.

Part B: Place the form for Sample B in front of the subject (master form provided in the tabbed section entitled "UDS Npsych Test Forms"). Read aloud the instructions:

[SAY]: "There are numbers and letters in circles on this page. Please take the pencil and draw a line, alternating in order between the numbers and letters. Start at number 1 (point to the number), then go to the first letter, A (point to the letter), then go to the next number, 2 (point to the number), and then the next letter, B (point to the letter), and so on. Please try not to lift the pencil as you move from one number or letter to the next. Work as quickly as you can."

If the subject makes an error, mark through the line and go back to the point at which the error was made and say, for example, "You were at number two. What is the next letter?" Wait for subject's response and say "Please start here and continue."

If the subject completes the sample correctly, go to Test B (master form provided in the tabbed section entitled "UDS Npsych Test Forms"). Repeat the instructions given for the sample. Start timing as soon as the instruction is given to begin. Stop timing when the Trail is completed, or stop subject when the maximum time is reached. Allow a maximum of 300 seconds for the test.

Scoring:

Record the total number of seconds to complete Part A, up to a maximum of 150 seconds. If the subject is not finished by 150 seconds, the score is 150. Enter the score for Part A on NACC UDS Form C1, item 7a.

Record the total number of seconds to complete Part B, up to a maximum of 300 seconds. If the subject is not finished by 300 seconds, the score is 300. Enter the score for Part B on NACC UDS Form C1, item 7b.

For both Part A and Part B, record errors of commission and omission as described below:

"Errors of commission" are defined as errors occurring when the subject connects two circles in the incorrect sequence. Each time this occurs, one error commission is scored.

"Errors of commission" are defined as errors occurring because the subject failed to draw a connecting line to a given circle in the correct sequence. This only occurs when the subject is very slow and does not complete the task in the time allotted.

- ⇨ One incorrectly sequenced number/letter may, in rare instances, be counted as both an error of commission and an error of omission (e.g., if the subject incorrectly draws a line to the letter K, but then never makes it back to that letter in the correct sequence).
- ⇨ If it is clear that the subject intended to touch a circle but barely missed it, do not count it as an omission. However, when this is first observed, caution the subject to touch circles.
- ⇨ If the subject draws a line through another circle while clearly on the way to the next circle in the sequence, caution the subject to avoid touching circles other than the ones intended, and make a note of what occurred on the raw data form, but do not count as an error of commission.

The subject may be unable to complete this test due to physical problems (e.g., tremor, dystonia). In that event, refer to KEY 2 on NACC UDS Form C1 and indicate the reason for incomplete data (i.e., physical problem, cognitive/ behavioral problem, other problem, verbal refusal) by entering the appropriate key code in the space provided for items 7a and 7b.

SAMPLE (actual form at back of manual)

Sample A

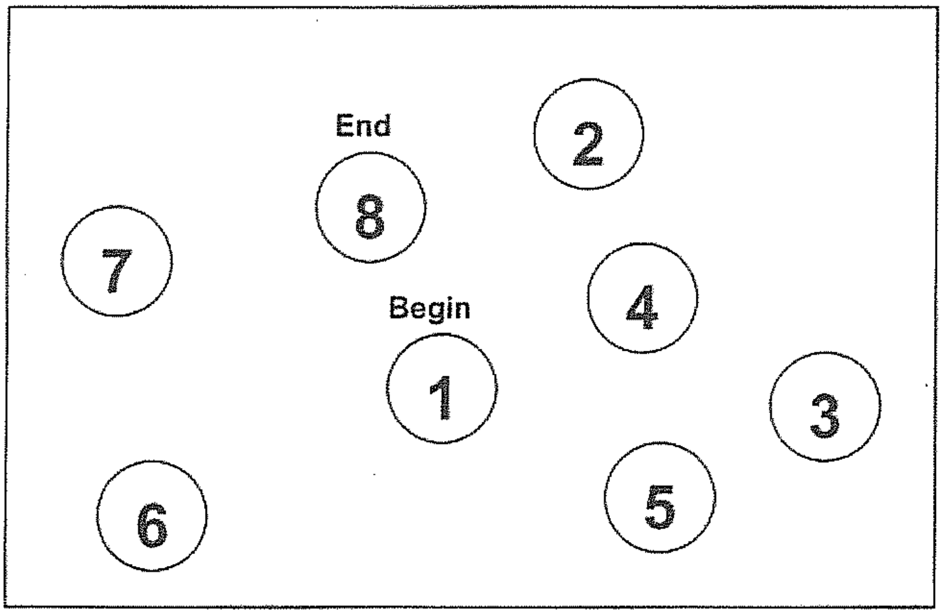

Test A

SAMPLE (actual form at back of manual)

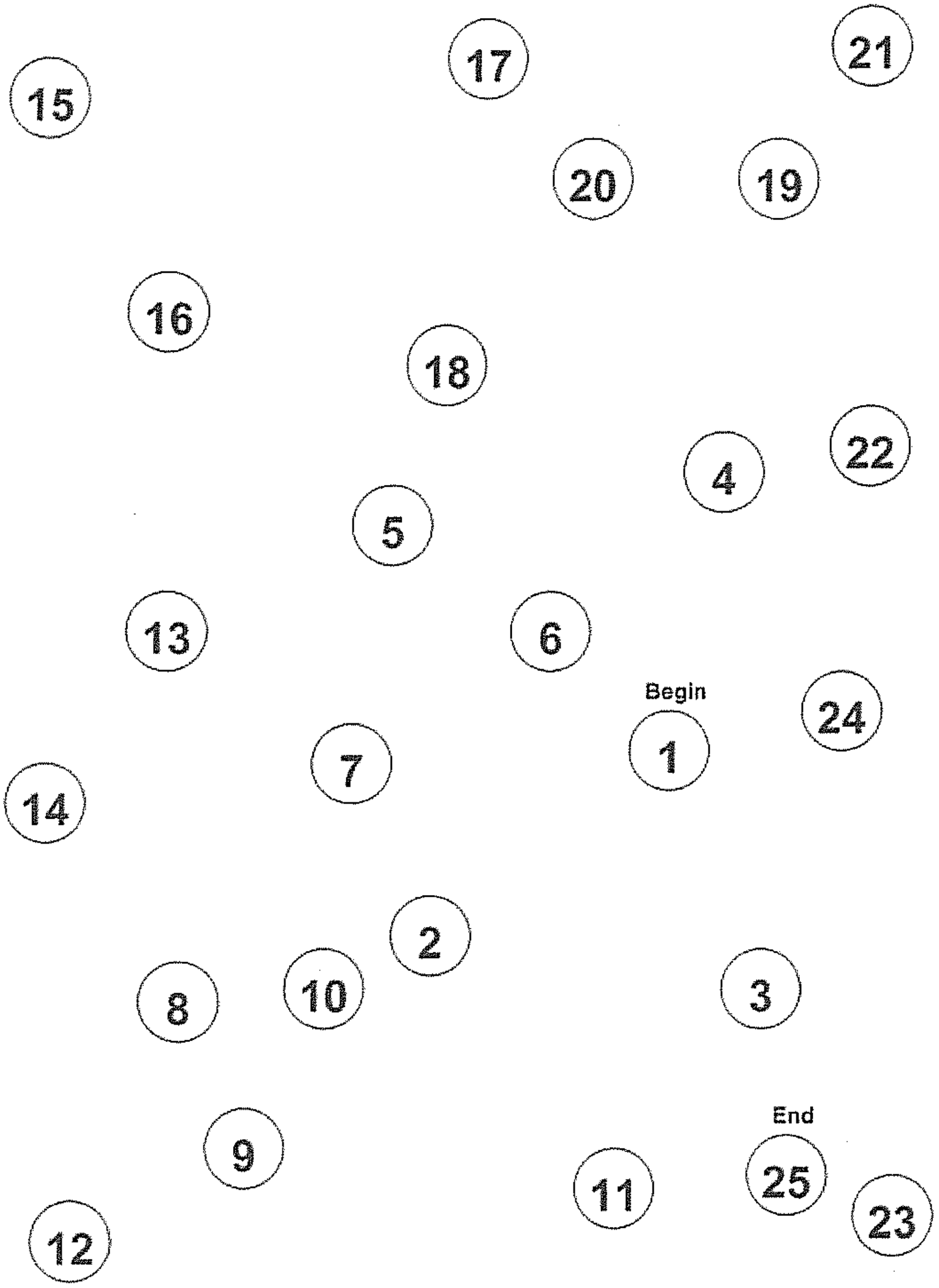

SAMPLE (actual form at back of manual)

Sample B

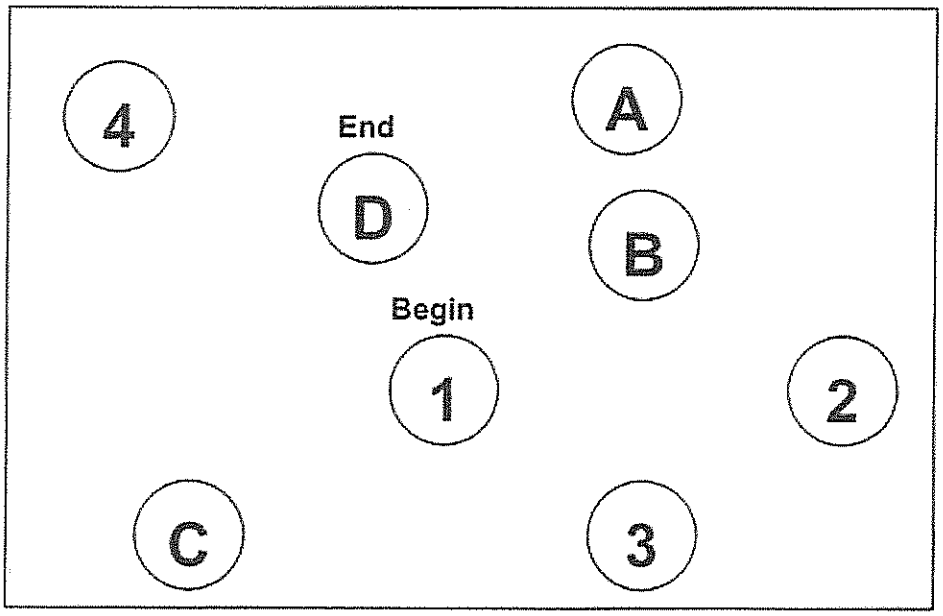

Test B

SAMPLE (actual form at back of manual)

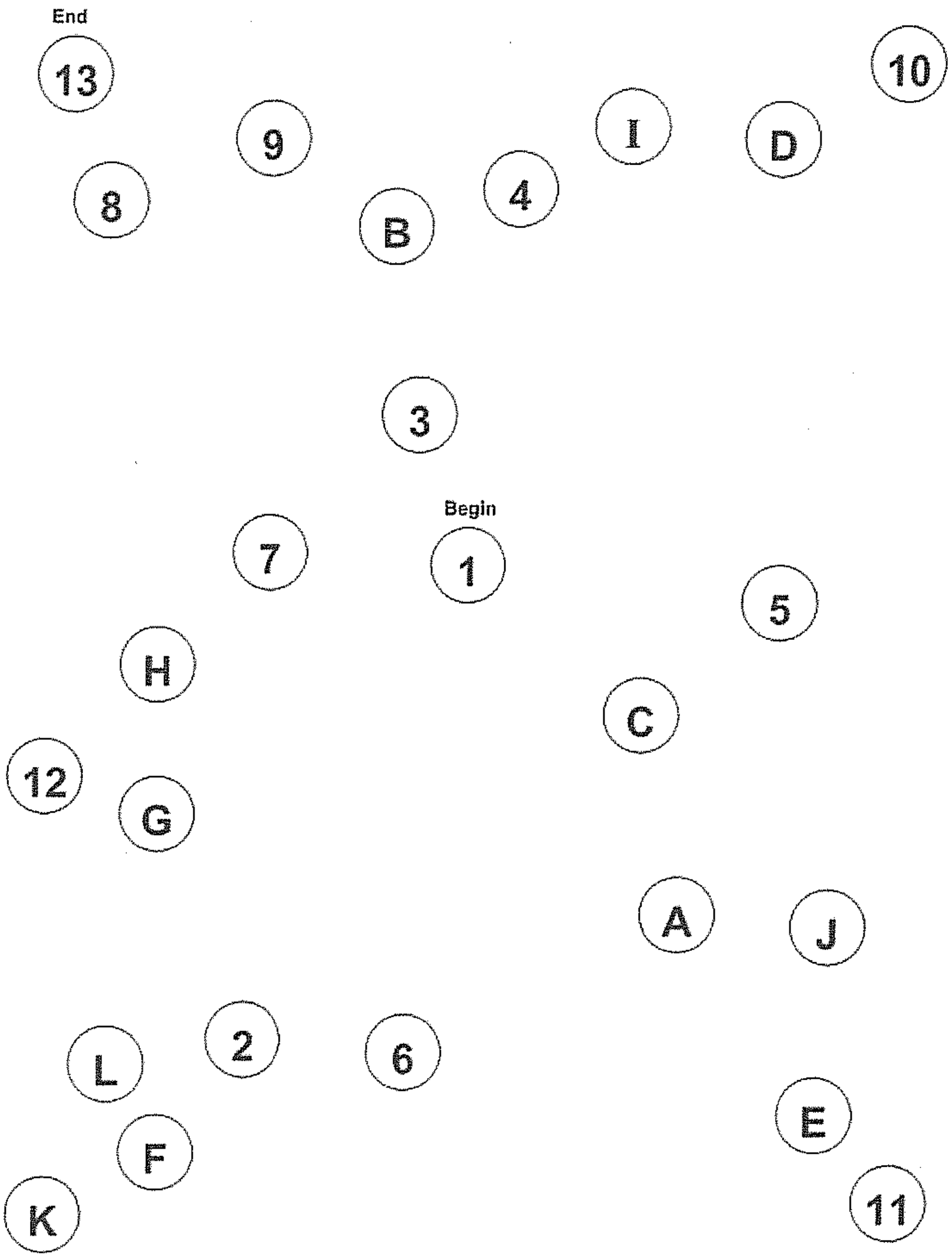

NACC UDS Neuropsychological Battery Worksheet

WAIS-R DIGIT SYMBOL

Description:
This subtest of the WAIS-R engages multiple cognitive abilities, including attention, psychomotor speed, complex scanning, visual tracking, and immediate memory. The test consists a series of small blank squares presented in rows, each randomly paired with one of nine numbers (1 to 9) printed directly above it. At the top of the page is a printed "key" that pairs each of the numbers 1 through 9 with an unfamiliar symbol. Following a short series of practice trials, the subject must use the key to fill in the blank squares in order (working left to right across the rows) with the symbol that is paired with the number above it, working as quickly as possible for 90 seconds. The number of blank squares filled in correctly within the time limit is the measure of interest.

Source:
Wechsler Adult Intelligence Scale® – Revised. Copyright© 1981, 1955 by Harcourt Assessment, Inc. Reproduced with permission. All rights reserved. *"Wechsler Adult Intelligence Scale"* and *"WAIS"* are trademarks of Harcourt Assessment, Inc., registered in the United States of America and other jurisdictions.

Administration:
Place the test form on table in front of subject (master form provided in the tabbed section entitled "UDS Npsych Test Forms").

[SAY]: "Look at these boxes. Notice that each has a number in the upper part and a special mark in the lower part. Each number has its own mark. Now look down here where the boxes have numbers in the top part but the squares at the bottom are empty." [*Point to the sample items.*]

[SAY]: "You are to put in each of the empty squares the mark should go there, like this: Here is a 2; the 2 has this mark, so I put it in this square like this. Here is a 1; the 1 has this mark, so I put it in this square. This number is 3; the 3 has this mark, so I put it in this square." [*Examiner fills in the first three boxes to demonstrate.*]

[SAY]: "Now you fill in the squares up to this heavy line." [*If subject makes errors, continue to help until all sample items are filled in correctly.*] "Yes, now you know how to do them."

[SAY]: "When I tell you to start, you do the rest of them. Begin here and fill in as many squares as you can, one after the other, without skipping any. Keep working until I tell you to stop. Work as quickly as you can without making any mistakes. When you finish this line, go on to this one. Ready? Begin." [*Allow 90 seconds.*]

- ⇨ If the subject skips an item, immediately give a reminder to go in order and not skip over any; point out the skipped item and direct the subject to continue from the last item completed successfully in order.
- ⇨ Do not intervene if the subject fills in a wrong symbol, but if the subject has clearly lost focus as to what to do (e.g., starts filling in something other than symbols), you may give the following reminder: "Remember, you are to fill in the symbol that goes below each number, as indicated in the key up above."
- ⇨ If the subject pauses at the end of a row, say "Good, keep going." [*Point to the beginning of the next row, if necessary.*]

Score:
Credit 1 point for each item filled in correctly within the 90-second time limit. The seven sample items are not included in the total score. Calculate the total number of points received (maximum possible = 93), and enter this score on NACC UDS Form C1, item 8a.

SAMPLE (actual form at back of manual)

DIGIT	1	2	3	4	5	6	7	8	9	SCORE
SYMBOL	—	⊥	⊐	L	⊔	O	Λ	X	=	

SAMPLES

2	1	3	7	2	4	8	2	1	3	2	1	4	2	3	5	2	3	1	4	5	6	3	1	4

1	5	4	2	7	6	3	5	7	2	8	5	4	6	3	7	2	8	1	9	5	8	4	7	3

6	2	5	1	9	2	8	3	7	4	6	5	9	4	8	3	7	2	6	1	5	4	6	3	7

9	2	8	1	7	9	4	6	8	5	9	7	1	8	5	2	9	4	8	6	3	7	9	8	6

NACC UDS Neuropsychological Battery Worksheet

LOGICAL MEMORY IIA – DELAYED

Description:
This is a measure of delayed recall (episodic memory) of the story read to the participant at the beginning of the testing session (Logical Memory IA–Immediate).

Source:
Wechsler Memory Scale® – Revised. Copyright© 1945, renewed 1974, 1987 by Harcourt Assessment, Inc. Reproduced with permission. All rights reserved. *"Wechsler Memory Scale"* and *"WMS"* are trademarks of Harcourt Assessment, Inc., registered in the United States of America and other jurisdictions.

Administration:
Administer this test at least 30 minutes and no more than 40 minutes after *Logical Memory IA–Immediate.* The examiner should complete other cognitive testing during the interval between the Immediate and Delayed tests. (**Note: if the 30–40 minute delay period has elapsed and another test is being administered, interrupt the other test and administer this test.**) The following instructions are to be read verbatim:

[SAY]: "Do you remember the little story I read to you a few minutes ago? Now I want you to tell me the story again. Tell me everything; begin at the beginning."

If the subject does not recall the story, it is permissible to offer the following reminder: "The story was about a woman who was robbed." Do not give any further help other than general encouragement. Make a note if the reminder is given, and do not then credit the subject for that item (i.e., "robbed") when scoring.

After the subject has recalled the story, prompt with "Anything else?" Record any additional information recalled and score appropriately.

Record the subject's responses on the *Worksheet for Logical Memory IIA–Delayed* (master form provided in the tabbed section entitled "UDS Npsych Test Forms") between the lines of the text. To simplify the process of recording, underline each word or unit that is reported verbatim, and write above the text any units that are reported but not verbatim. The examiner may prefer to tape record the subject's response and then transcribe the results after the session; this is acceptable if appropriate consent has been obtained.

Scoring:
Scoring is deferred until after the examination. Use the same scoring procedure as for Logical Memory IA, treating the phrases and words separated by diagonal lines in the passages as items or units, and scoring one point for each unit correctly repeated, for a total of 25 points. Non-verbatim responses that are acceptable and receive full credit are listed in the Weschler Memory Scale-Revised manual.

Record on the worksheet each unit that is successfully recalled, then total the units recalled and enter that score (00-25) in the space provided. This is the number to be entered on NACC UDS Form C1, item 9a.

Calculate the amount of time, in minutes, that has elapsed since the administration of the first test, *Logical Memory IA–Immediate*, and enter the total time on the worksheet and on NACC UDS Form C1, item 9b.

Worksheet for LOGICAL MEMORY IIA–DELAYED

SAMPLE (actual form at back of manual)

Administer this test at least 30 minutes and no more than 40 minutes after *Logical Memory IA–Immediate*. The examiner should complete other cognitive testing during the interval between the Immediate and Delayed tests. **Note: if the 30–40 minute delay period has elapsed and another test is being administered, interrupt that test and administer this test.**

The following instructions are to be read verbatim:

[SAY]: "Do you remember the little story I read to you a few minutes ago? Now I want you to tell me the story again. Tell me everything; begin at the beginning."

If the subject does not recall the story, it is permissible to offer the following reminder: "The story was about a woman who was robbed." Do not give any further help other than general encouragement. Make a note if the reminder is given, and then do not credit the subject for that item (i.e., "robbed") when scoring.

[Record the subject's responses between the lines of the text below. To simplify the process of recording, underline each word or unit that is reported verbatim, and write above the text any units that are reported but not verbatim. The examiner may prefer to tape record the subject's response and then transcribe the results after the session; this is acceptable if appropriate consent has been obtained.]

After the subject has recalled the story, prompt with "Anything else?". Record any additional information recalled and score appropriately.

**Story A – Delayed**	**Score**
Anna / Thompson / of South / Boston /, employed / as a cook /	___ (0–6)
in a school / cafeteria /, reported / at the City Hall /Station /	___ (0–5)
that she had been held up / on State Street / the night before /	___ (0–3)
and robbed / of fifty-six dollars /. She had four /	___ (0–3)
small children /, the rent was due /, and they had not eaten /	___ (0–3)
for two days /. The police /, touched by the woman's story /,	___ (0–3)
took up a collection / for her /.	___ (0–2)

Total number of story units recalled: ___ ___

Time elapsed since Logical Memory IA – Immediate: ___ ___ (minutes)

NACC UDS Neuropsychological Battery Worksheet

BOSTON NAMING TEST (30 Odd-numbered items)

Description:
This reduced version of the Boston Naming Test is a measure of the ability to orally label (name) 30 line drawings of objects. The objects are presented in order of frequency, from most frequent (i.e., bed) to least frequent (i.e., abacus). This test is sensitive to aphasia and also to object recognition deficits. For the purposes of the UDS, only the odd-numbered items[1] from the full test will be administered.

Source:
Boston Naming Test, second edition. Kaplan E, Goodglass H, Weintraub S. Philadelphia: Lea and Febiger; 1983. Adapted by special permission of the publisher, PRO-ED Inc., 8700 Shoal Creek Blvd., Austin TX 78757-6897 (800-897-3202; www.proedinc.com). Copyright© 2001.

Administration:
[SAY]: "I am going to show you some pictures, one at a time. I would like you to tell me the name of the object that you see." Display the first picture and say, "Tell me what this is called."

Begin at item 1 and present all 30 items in order. Allow 20 seconds for each response, unless the subject says that s/he doesn't know the word before 20 seconds has elapsed. If the answer is correct, put a check mark (✓) in the column titled "*Uncued–Correct*" on the *Worksheet for Boston Naming Test (30 Odd-numbered items)* (master form provided in the tabbed section entitled "UDS Npsych Test Forms"); record any response other than the correct one in the column titled "Incorrect Response". Enter the latency in seconds, if desired.

If the subject has given a response that indicates misperception of the picture, give the *stimulus cue*, which is printed in parentheses after the item name. The subject is allowed up to 20 seconds to name the picture after the stimulus cue is given. If the item is named correctly within that time, check the column entitled "*Stimulus Cue–Correct*"; otherwise, check "*Stimulus Cue–Incorrect*" and record the response verbatim in the previous column entitled "*Incorrect Response*". The stimulus cue is presented only when the subject's response reflects misperception.

If the response following the stimulus cue is incorrect, move on to the *phonemic cue.* The phonemic cue is also given after every failure to respond or after any incorrect response. Provide the first sound in the name of the item (indicated in underlined bold text on the test form). If the subject succeeds with a phonemic cue, place a check mark in the column entitled "*Phonemic Cue–Correct*"; if the subject fails, place a check mark in the column entitled "*Phonemic Cue–Incorrect*". The number of items correct following phonemic cues is of clinical interest but is <u>not</u> included in the total score.

- ⇨ Record the subject's responses verbatim if incorrect or circumlocutory, and write "DK" only if the subject actually says s/he doesn't know.
- ⇨ Mispronunciations are treated as incorrect unless they clearly reflect a regional or dialect-specific pronunciation (e.g., someone from the Boston area may omit the 'r' sound in 'dart'). If a mispronunciation does not reflect such a regionalism (e.g., 'esculator'), the answer is incorrect and cueing proceeds as appropriate.

---

[1] Mack WJ, Freed DM, Williams BW, Henderson VW. Boston Naming Test: shortened versions for use in Alzheimer's disease. *J Gerontol.* 1992;47(3):P154-158.

⇨ If the subject gives a more general or circumlocutory response (e.g., 'boat' or 'it floats on water' for the word 'canoe'), say "Can you think of a more specific name for it?"

⇨ If the subject gives a more specific response (e.g., 'daisy' for 'flower'), say "Can you think of a more general name for it?"

⇨ If the subject gives the correct name, but says that it is not that object (e.g., "Well, it's not a canoe"), the response is considered incorrect and cueing proceeds if appropriate.

Discontinue testing after 6 consecutive failures (i.e., failure to name correctly either without assistance or with a stimulus cue).

Scoring:
The total score is the number of items that are named correctly without assistance PLUS the number of items named correctly following a stimulus cue if one had to be given (maximum total score = 30). Enter the total score in the space provided on the worksheet and on NACC UDS Form C1, item 10a.

Worksheet for BOSTON NAMING TEST (30 Odd-numbered items)

SAMPLE (actual form at back of manual)

Items	Incorrect Response	Uncued	Stimulus Cue		Score	Latency	Phonemic Cue	
		Correct	Correct	Incorrect	1 or 0	(seconds)	Correct	Incorrect
1. bed (a piece of furniture)								
3. pencil (used for writing)								
5. whistle (used for blowing)								
7. comb (used for fixing hair)								
9. saw (used by a carpenter)								
11. helicopter (used for air travel)								
13. octopus (an ocean animal)								
15. hanger (found in a closet)								
17. camel (an animal)								
19. pretzel (something to eat)								
21. racquet (used for sports)								
23. volcano (a kind of mountain)								
25. dart (you throw it)								
27. globe (a kind of map)								
29. beaver (an animal)								
31. rhinoceros (an animal)								
33. igloo (type of house)								
35. dominoes (a game)								
37. escalator (you go up on it)								
39. hammock (you lie on it)								
41. pelican (a bird)								
43. pyramid (found in Egypt)								
45. unicorn (mythical animal)								
47. accordion (a musical instrument)								
49. asparagus (something to eat)								
51. latch (part of a door)								
53. scroll (a document)								
55. sphinx (it's found in Egypt)								
57. trellis (used in a garden)								
59. protractor (measures angles)								

Scoring:

a. Total correct without a cue: ____

b. Total stimulus cues given: ____

c. Total correct with a stimulus cue: ____

d. Total phonemic cues given: ____ (*clinical use only; not to be used in scoring*)

e. Total correct with a phonemic cue: ____ (*clinical use only; not to be used in scoring*)

f. Total correct: ____ **sum of a+c above (30 maximum)**

Worksheet for BOSTON NAMING TEST (30 Odd-numbered items)

SAMPLE (actual forms at back of manual)

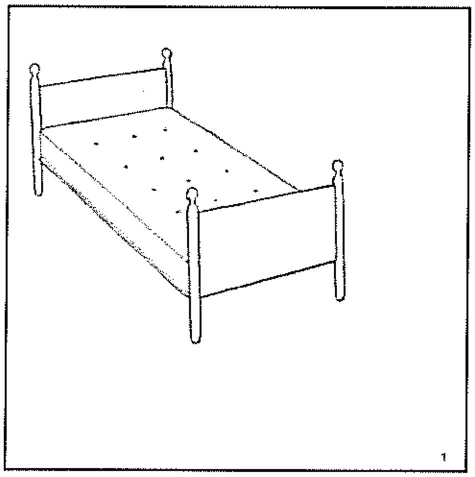

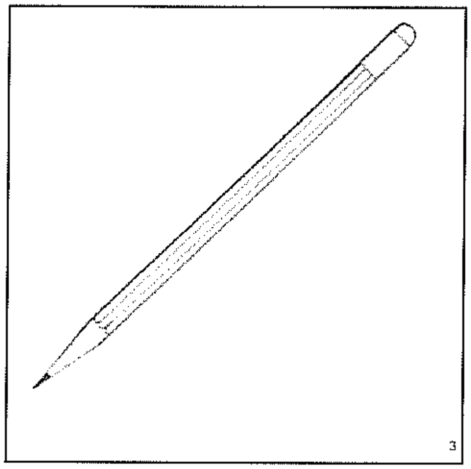

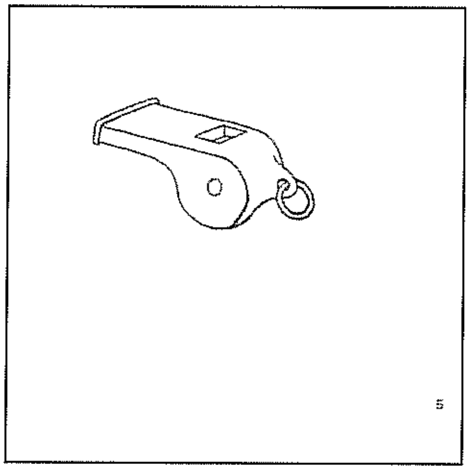

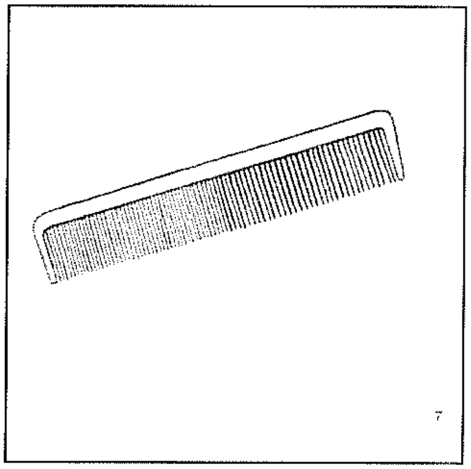

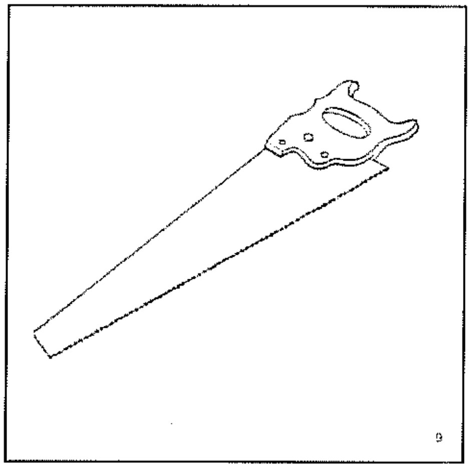

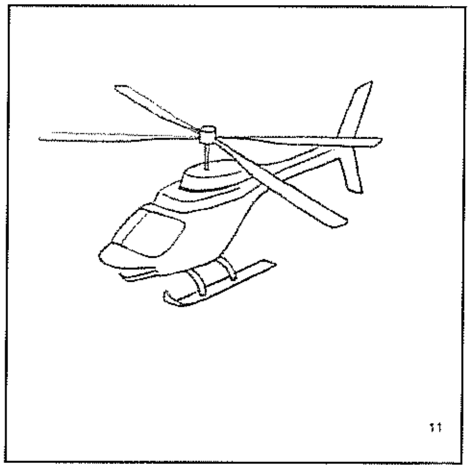

Worksheet for BOSTON NAMING TEST (30 Odd-numbered items)

SAMPLE (actual forms at back of manual)

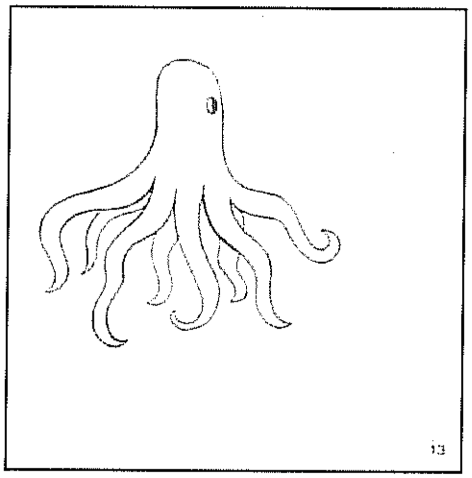

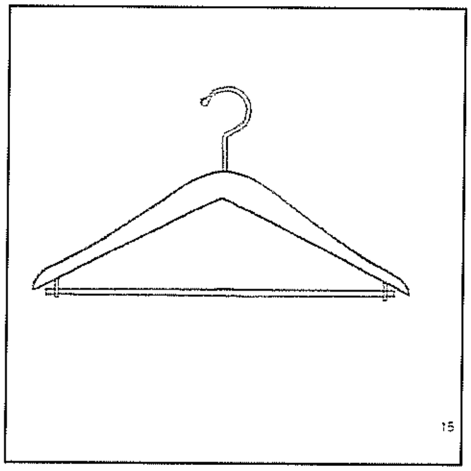

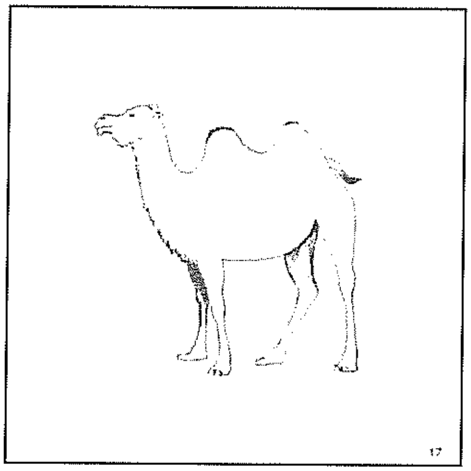

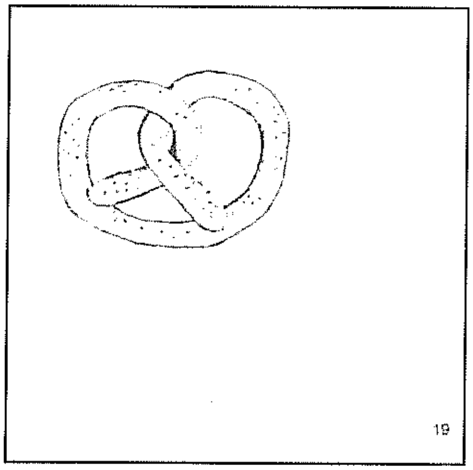

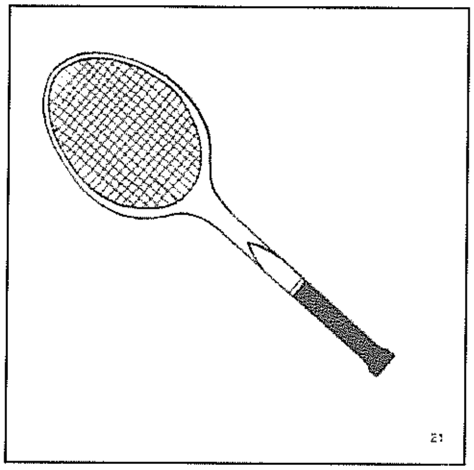

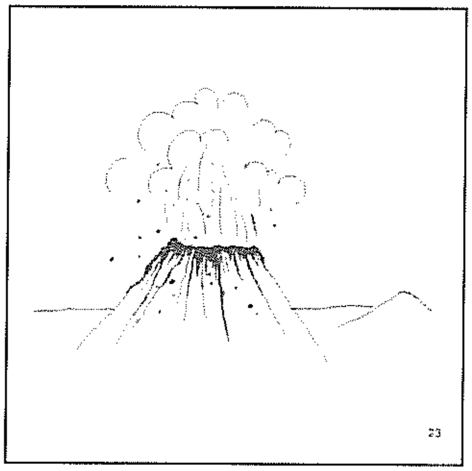

Worksheet for BOSTON NAMING TEST (30 Odd-numbered items)

SAMPLE (actual forms at back of manual)

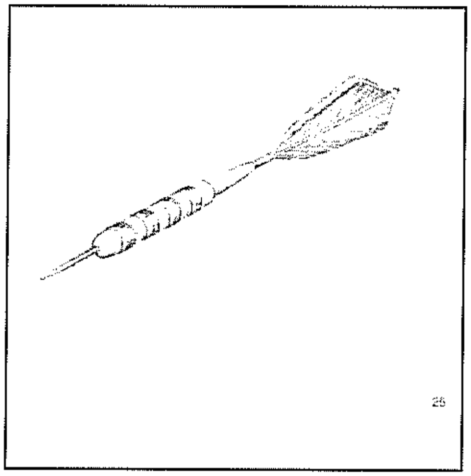

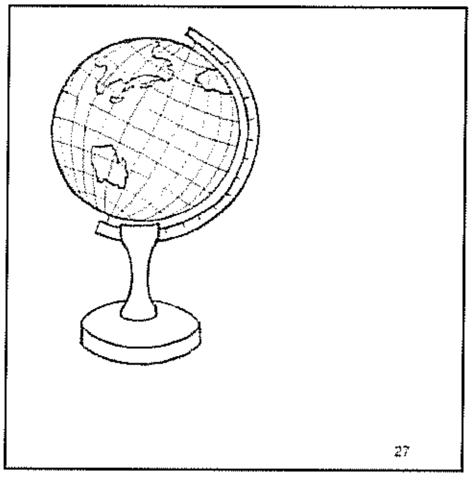

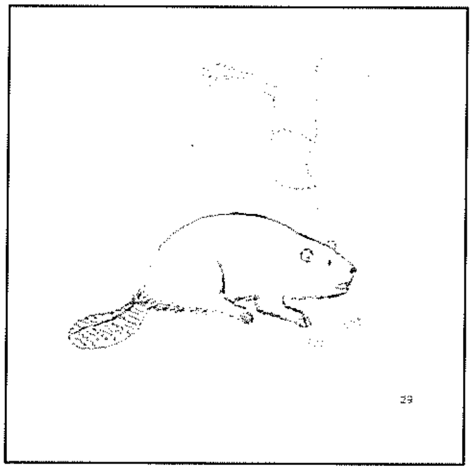

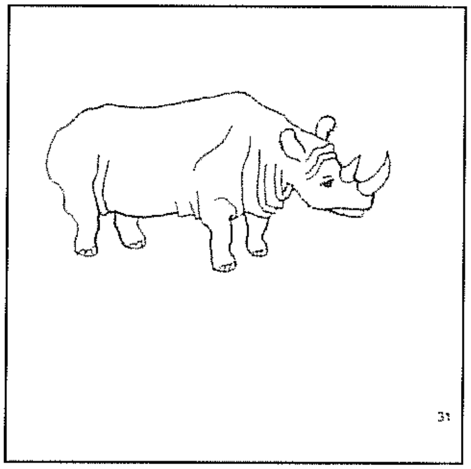

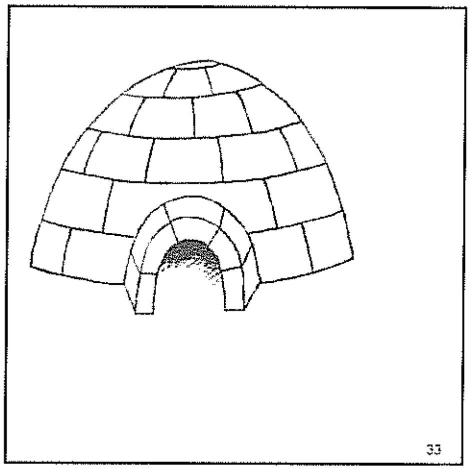

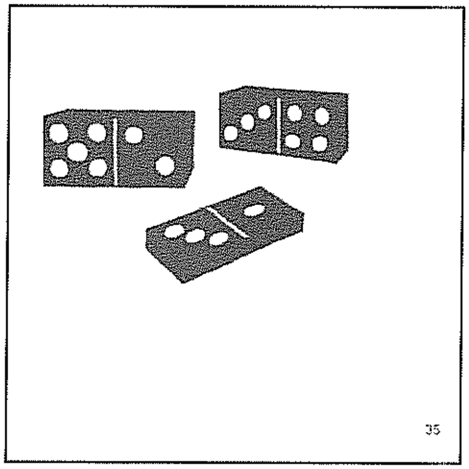

Worksheet for BOSTON NAMING TEST (30 Odd-numbered items)

SAMPLE (actual forms at back of manual)

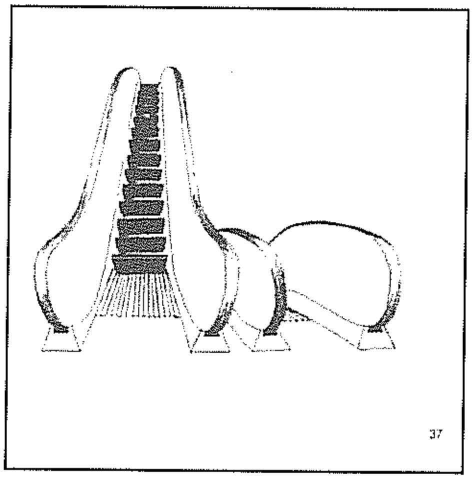

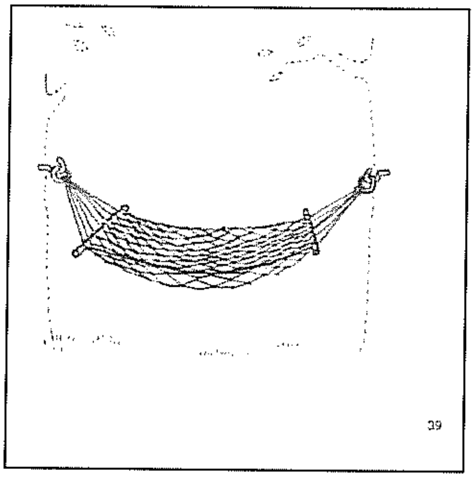

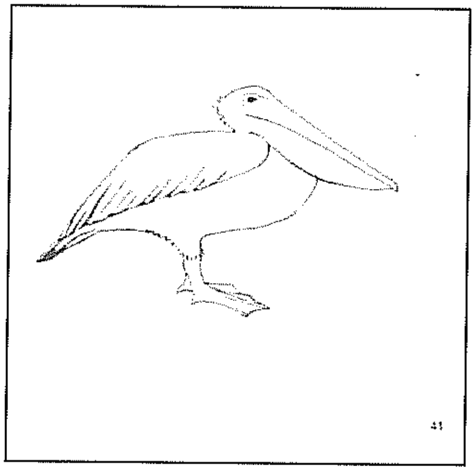

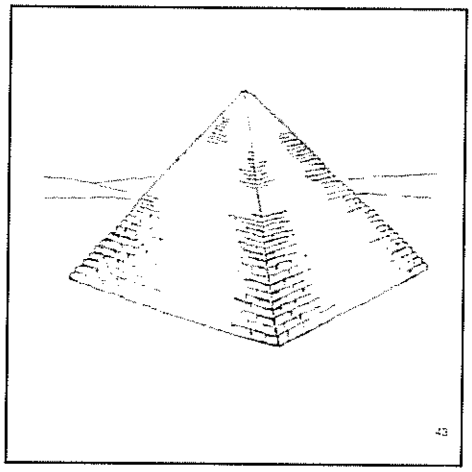

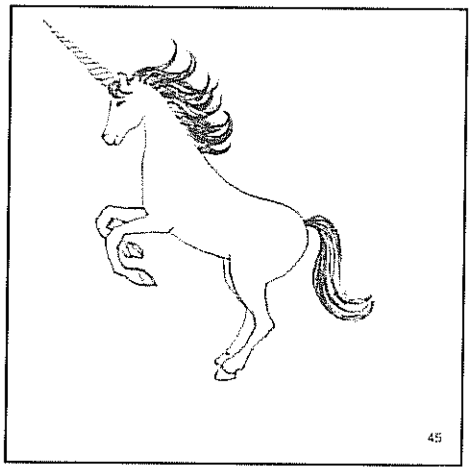

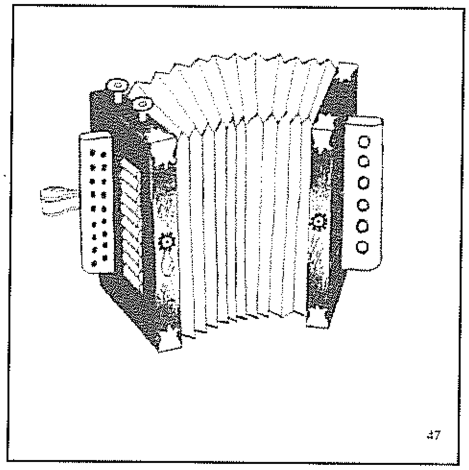

Worksheet for BOSTON NAMING TEST (30 Odd-numbered items)

SAMPLE (actual forms at back of manual)

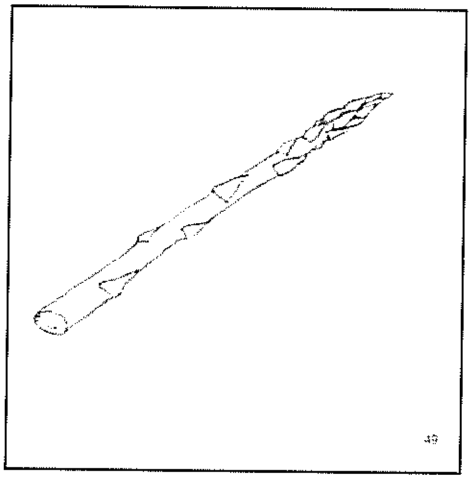

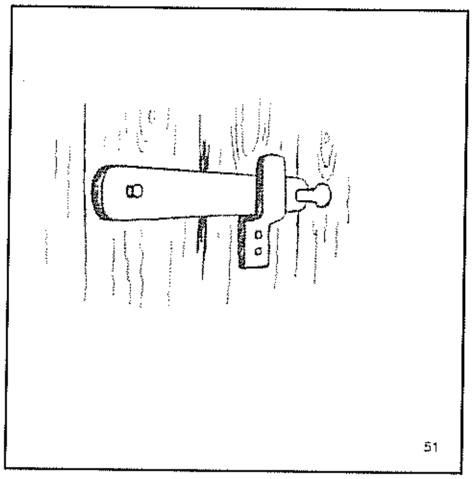

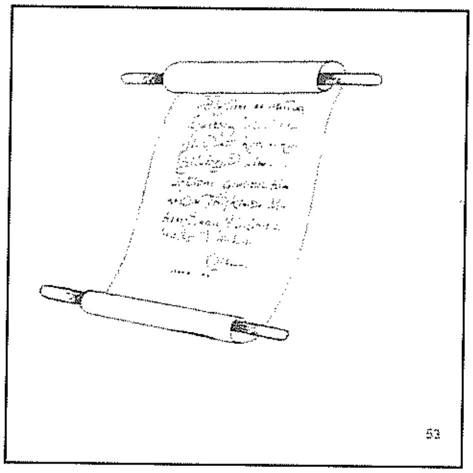

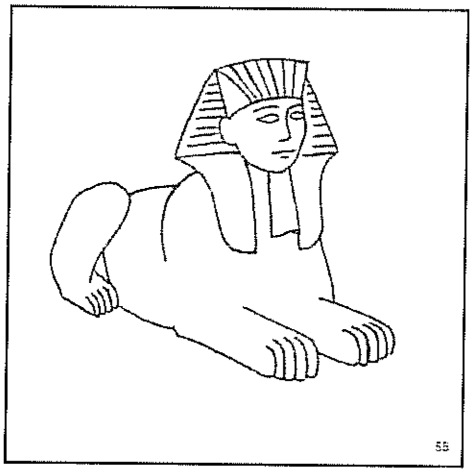

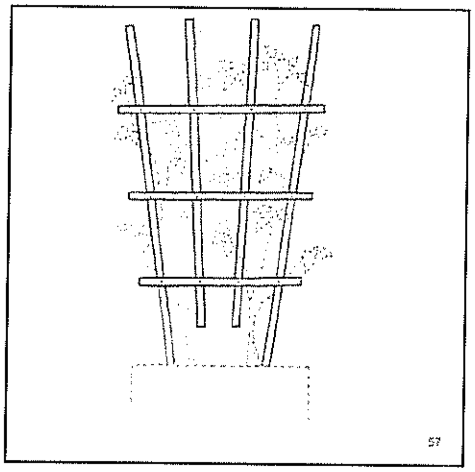

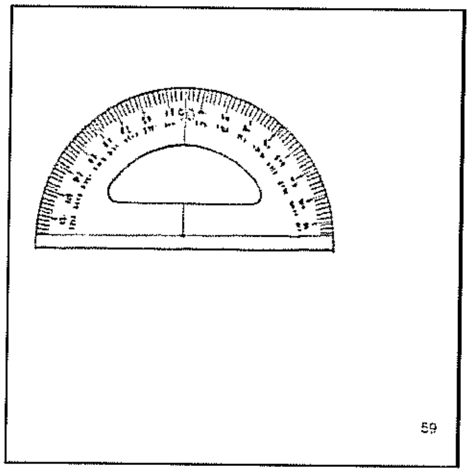

LONG LIFE FAMILY STUDY	**(Affix Label Here)**<br>Participant ID: ____________<br>Participant Name Code: ____________	Date Form Filled Out: ☐☐☐☐☐☐☐☐☐<br>d d M M M y y y y<br>(e.g., 10JUN2005)<br>Interviewer Code: ☐☐☐<br>Circle Field Center Location:<br>BU CU DK UP

Cognitive Assessments

Please Mark the Appropriate Box Below:

☐[1] ....These Forms were Administered In-Person by Study Personnel
☐[2] ....These Forms were Administered via Telephone by Study Personnel

---

Please use NACC UDS Forms which follow:

- **Part A: Folstein Mini-Mental Exam**
- **Part B: Logical Memory 1A – Immediate**
- **Part C: Digit Span Forward**
- **Part D: Digit Span Backward**
- **Part E: Category Fluency – Animals**
- **Part F: Category Fluency – Vegetables**
- **Part G: Digit-Symbol Substitution Task**
- **Part H: Logical Memory IIA – Delayed**

***Interviewer Note*: Please refer to Amendments to these panels on Page 11-1, Chapter 11 of the LLFS MOP.**

Interviewer Note: Please complete the following section upon completion of the Cognitive Battery testing.

Cognitive Battery Validity Ratings

The following variables were judged to affect test performance:

		Yes	No
1.	Visual Impairment	_____	_____
2.	Hearing Impairment	_____	_____
3.	Environmental Conditions (interruptions, noise, etc.)	_____	_____
4.	Dispositional factors (cooperativeness, attentiveness, etc.)	_____	_____
5.	Other factors	_____	_____

Please rate the **overall validity** of the cognitive battery according to the following scale ***(circle one)***:

1 All Tests Invalid

2 Most Tests Completely Invalid *__OR__* All Tests Possibly Invalid

3 1-2 Tests Completely Invalid *__OR__* A Few Tests Possibly Invalid

4 All Tests Valid

LONG LIFE FAMILY STUDY	**(Affix Label Here)**<br>Participant ID: ________________<br>Participant Name Code: __________	Date Form Filled Out: ☐☐☐☐☐☐☐☐☐<br>d d M M M y y y y<br>(e.g., 10JUN2005)<br>Interviewer Code: ☐☐☐<br>Circle Field Center Location:<br>**BU CU DK UP**

Telephone Interview For Cognitive Status (TICS)

Starting date/time of this Interview:

Date: ____ ____ / ____ ____ ____ / ____ ____ ____ ____

Time: ___ ___ **:** ___ ___ **AM / PM (circle one)**

Question	Response		Score
1. Tell me your full name.	First: ______________________<br>Last: ______________________		0-2 _______
2. What is today's date?	Month: ______________________<br>Date: ______________________<br>Year: ___ ___ ___ ___<br>Day:<br>______________________<br>_<br>Season: ______________________		0-1 _______<br>0-1 _______<br>0-1 _______<br>0-1 _______<br>0-1 _______
3. Where are you right now?	House #: ______________________<br>Street: ______________________<br>City: ______________________<br>State: ______________________<br>Zip Code: ______________________		0-1 _______<br>0-1 _______<br>0-1 _______<br>0-1 _______<br>0-1 _______
4. Count backward from 20 to 1.			0-2 _______
5. I'm going to read you a list of 10 words. Please listen carefully and try to remember them. When I am done, tell me as many words as you can, in any order. Ready? The words are: **Cabin; Pipe; Elephant; Chest; Silk; Theater; Watch; Whip; Pillow; Giant.** Now tell me all the words you can remember.	Cabin _______<br>Pipe _______<br>Elephant _______<br>Chest _______<br>Silk _______	Theater _______<br>Watch _______<br>Whip _______<br>Pillow _______<br>Giant _______	0-10 _______

Question	Response	Score
6. 100 minus 7 equals what? And 7 from that? Etc.<br>And 7 from that?<br>And 7 from that?<br>And 7 from that?		0-5 _______
7.<br>• What do people usually use to cut paper?<br>• How many things are in a dozen?<br>• What do you call the prickly green plant that lives in the desert?<br>• What animal does wool come from?	________________<br>________________<br>________________<br>________________	0-1 _______<br>0-1 _______<br>0-1 _______<br>0-1 _______
8.<br>• Say this: "No ifs, ands or buts."<br>• Say this: "Methodist Episcopal".	________________<br>________________	0-1 _______<br>0-1 _______
9.<br>• Who is the President of the United States right now?<br>• Who is the Vice-President?	________________<br>________________	0-1 _______<br>0-1 _______
10. With your finger, tap five times on the part of the phone that you speak into.	________________	0-2 _______
11. I'm going to give you a word and I want you to give me its opposite. For Example, the opposite of hot is cold.<br>• What is the opposite of "West"?<br>• What is the opposite of "Generous"?	________________<br>________________	0-1 _______<br>0-1 _______
12. Earlier I read you a long list of words that I asked you to remember. I would like you to try to tell me as many of those words as you can remember now.	Cabin _______ Theater _______<br>Pipe _______ Watch _______<br>Elephant _______ Whip _______<br>Chest _______ Pillow _______<br>Silk _______ Giant _______	0-10 _______
**13. TICS Total Score (do not include Q12)**		0-42 _______
**14. Revised TICS Total Score (including Q12)**		0-52 _______

LONG LIFE FAMILY STUDY	**(Affix Label Here)**<br>Participant ID: ___________<br>Participant Name Code: ___________	Date Form Filled Out: ☐☐☐☐☐☐☐☐☐<br>d d M M M y y y y<br>(e.g., 10JUN2005)<br>Interviewer Code: ☐☐☐<br>Circle Field Center Location:<br>**BU CU DK UP**

Informant-Based Date of Onset Interview

1. What is your relationship with ______________________ *[insert name of participant]*?

   ☐1 .... Spouse
   ☐2 .... Child
   ☐3 .... Friend/Companion
   ☐4 .... Other ___________________________ *(please specify)*

2. Do you live with ______________________ *[insert name of participant]*?

   ☐1 .... Yes
   ☐0 .... No

3. In the past year, how often have you seen *[insert name of participant]*? (***Interviewer Note:* Any interval can be used to establish this date. The following may be helpful to establish the date. "About how many times per week?" OR "How many times per month?"**)

   Frequency of contact with the patient during the last year? Days _____________ / 365

4. In the last 5 years, how often have you seen *[insert name of participant]*? (***Interviewer Note:* The interval may be described year by year and summed.**)

   Frequency of contact with the patient over 5 years? Days _____________ / 1200

5. ***Interviewer Script:*** *I am going to ask you several questions about the very first symptoms or problems you noticed in [insert name of participant]. You may be aware of many problems but this interview will focus on the first or earliest ones.*

   Have you noticed *[Problem]*? When did you first notice that? When was this not present? ***(Interviewer Note:* Repeat for items A-H in the table below.)**

   Use the following page to record detailed descriptions of specific events that illustrate the earliest problems. A date must be set when the problem was definitely present and definitely absent. If necessary, use additional questions to clarify the timeline (page 2).

   In some cases, the informant will describe an episode that does not appear to meet criteria for the category that is being queried (see page 3 for category descriptions). Record this response in the correct category regardless of the label given by the informant.

Participant ID: ____________________________ Participant Name Code: ______________________

Have you noticed that [Insert Participant's Name] has had:		When did you first notice that ?		When was this not present?	
		Month	Year	Month	Year
5a. Memory Problems	☐Yes ☐No				
5b. Performance Changes	☐Yes ☐No				
5c. Language Problems	☐Yes ☐No				
5d. Trouble with orientation (knowing time or place)	☐Yes ☐No				
5e. Depression	☐Yes ☐No				
5f. Personality Changes	☐Yes ☐No				
5g. Behavior Changes	☐Yes ☐No				
5h. Hallucinations, delusions, or paranoid ideas	☐Yes ☐No				

Note to Interviewer: ***The questions provided below are suggestions to help you better establish answers to Q5a-Q5h above.***

The following questions may be useful to help establish when the problem was definitely there:

"Do you remember this occurring during any events such as birthdays, anniversaries, or holidays?"
"Do you recall what year it was, or what season of the year it was?"
"Do you remember where you were or where the patient was when you first noticed the problem?"

The following additional questions may be useful to establish when the problem was definitely absent:

"When was the last time you think *[insert participant's name]* was not having this kind of problem?"
"When was his/her *[Specific Area]* about the same as yours?"
"When was his/her *[Specific Area]* as good as other people his/her the same age?"

Record detailed description of problems: ____

____

____

____

____

____

____

____

____

6. Interviewer, describe your impression of the quality of onset information:

☐5 .... Very Good
☐4 .... Good
☐3 .... Unsure
☐2 .... Poor
☐1 .... Very Poor

Memory

Difficulty with recalling things (e.g., names, all or important parts of conversations, or lists of things); forgetting details, appointments, or messages; losing or misplacing items.

Performance

Problems with carrying on occupational and recreational activities; trouble remembering "how to do" a previously well known skill.

Language

Word-finding problems and misnaming of things; difficulty with understanding conversations.

Disorientation

Confusion about the time (including date) and place.

Personality

Intensification of a pre-existing personality trait; notable new trait or a marked change, e.g., paranoia (pervasive and unwarranted suspiciousness and mistrust of people, does not include other delusions), apathy (socially withdrawn, loss of interest in usual activities), egocentricity (selfishness or unawareness of significant others), dependency (passively allowing others to assume responsibility for major areas of life because of inability to function independently).

Depressed Mood

Persistent and severe depressed mood; vegetative signs are not required.

Behavior

Physical aggression or verbal abuse; lack of adequate personal grooming; sexual indiscretion, including verbal behavior; rigidity or stubbornness; emotional ability (e.g., laughing or crying inappropriately).

Psychosis

Delusion (persistent false beliefs that cannot be removed by contradictory evidence); hallucination (a visual, auditory, or olfactory perception of something that does not exist).

Adapted from Sano et al. (1995). A standardized technique for establishing onset and duration of symptoms of Alzheimer's disease. Archives of Neurology, 52, 961-966.

LONG LIFE FAMILY STUDY	**(Affix Label Here)**<br>Participant ID: ____________________<br>Participant Name Code: _____________	Date Form Filled Out:<br>☐☐☐☐☐☐☐☐☐<br>**d d M M M y y y y**<br>(e.g., 10JUN2005)<br>Interviewer Code: ☐☐☐<br>Circle Field Center Location:<br>**BU CU DK UP**

Performance Measures

***Examiner*: Where is this test being performed?**

☐[1] ....This Form was Administered by Study Personnel in the Clinic
☐[2] ....This Form was Administered by Study Personnel in the Home
☐[3] ....Other (Please Specify)_________________________

A. SHORT PHYSICAL PERFORMANCE BATTERY (SPPB)

Interviewer: *All of the tests should be performed in the same order as they are presented in this protocol. Instructions to the participant are shown in bold italic and should be given exactly as they are written in this script.*

Now let's begin the evaluation. I would now like you to try to move your body in different movements. I will first describe and show each movement to you. Then I'd like you to try to do it. If you cannot do a particular movement, or if you feel it would be unsafe to try to do it, tell me and we'll move on to the next one. Let me emphasize that I do not want you to try to do any movement that you feel might be unsafe.

Do you have any questions before we begin?

Measure: Balance Tests

Interviewer: *The participant must be able to stand unassisted without the use of a cane or walker. You may help the participant to get up.*

Side-by-Side Stand

Interviewer Script: ***Now I will show you the first movement.*** *(Demonstrate)* ***I want you to try to stand with your feet together, side-by-side, for about 10 seconds. You may use your arms, bend your knees, or move your body to maintain your balance, but try not to move your feet. Try to hold this position until I tell you to stop.***

Stand next to the participant to help him/her into the side-by-side position.

- *Supply just enough support to the participant's arm to prevent loss of balance.*
- *When the participant has his/her feet in position, ask "**Are you ready?**"*
- *Then let go and begin timing as you say, "**Ready, begin.**"*
- *Stop the stopwatch and say "**Stop**" after 10 seconds or when the participant steps out of position or grabs your arm.*
- *If participant is unable to hold the position for 10 seconds, record result and go to the gait speed test.*

---

1. Is the participant able to do a side-by-side stand (balance unaided) for 10 seconds?

☐[1] ....Yes, held for 10 seconds
☐[0] ....No, not held for 10 seconds **Record Result in Q2; End Balance Test; Go to Gait Speed Test**
☐[R] ....Not attempted/Refused **End Balance Test/Go to Q3**

2. Number of seconds held, if less than 10: ____ . ____ ____

3. If participant did not attempt test, indicate reason with an **"X".** **End Balance Test; Go to Gait Speed Test**

☐[1] ....Tried, but unable
☐[2] ....Participant could not hold position unassisted
☐[3] ....Not attempted, interviewer felt unsafe
☐[4] ....Not attempted, participant felt unsafe
☐[5] ....Participant unable to understand instructions
☐[6] ....Other (Please Specify)_________________________
☐[R] ....Participant Refused

Semi-Tandem Stand

Interviewer Script: ***Now I will show you the second movement.*** *(Demonstrate)* ***I want you to try to stand with the side of the heel of one foot touching the big toe of the other foot for about 10 seconds. You may put either foot in front, whichever is more comfortable for you. You may use your arms, bend your knees, or move your body to maintain your balance, but try not to move your feet. Try to hold this position until I tell you to stop.***

- *Stand next to the participant to help him/her into the semi-tandem position.*
- *Supply just enough support to the participant's arm to prevent loss of balance.*
- *When the participant has his/her feet in position, ask "**Are you ready?**"*
- *Then let go and begin timing as you say, "**Ready, begin.**"*
- *Stop the stopwatch and say "**Stop**" after 10 seconds or when the participant steps out of position or grabs your arm.*
- *If participant is unable to hold the position for 10 seconds, record result and go to the gait speed test.*

---

4. Is the participant able to do a semi-tandem stand (balance unaided) for 10 seconds?

- ☐ 1 ....Yes, held for 10 seconds
- ☐ 0 ....No, not held for 10 seconds **Record Result in Q5; End Balance Test; Go to Gait Speed Test**
- ☐ R ....Not attempted/Refused **End Balance Test; Go to Q6**

5. Number of seconds held, if less than 10: ____ . ____ ____ **Go to Gait Speed Test**

6. If participant did not attempt test, indicate reason with an **"X".** **End Balance Test; Go to Gait Speed Test**

- ☐ 1 ....Tried, but unable
- ☐ 2 ....Participant could not hold position unassisted
- ☐ 3 ....Not attempted, interviewer felt unsafe
- ☐ 4 ....Not attempted, participant felt unsafe
- ☐ 5 ....Participant unable to understand instructions
- ☐ 6 ....Other (Please Specify)_________________________
- ☐ R ....Participant Refused

Tandem Stand

Interviewer Script: ***Now I will show you the third movement.*** *(Demonstrate)* ***I want you to try to stand with the heel of one foot in front of and touching the toes of the other foot for about 10 seconds. You may put either foot in front, whichever is more comfortable for you. You may use your arms, bend your knees, or move your body to maintain your balance, but try not to move your feet. Try to hold this position until I tell you to stop.***

- *Stand next to the participant to help him/her into the tandem position.*
- *Supply just enough support to the participant's arm to prevent loss of balance.*
- *When the participant has his/her feet in position, ask "**Are you ready?**"*
- *Then let go and begin timing as you say, "**Ready, begin.**"*
- *Stop the stopwatch and say "**Stop**" after 10 seconds or when the participant steps out of position or grabs your arm.*
- *If participant is unable to hold the position for 10 seconds, record result and go to the gait speed test.*

---

7. Is the participant able to do a tandem stand (balance unaided) for 10 seconds?

- ☐ 1 ....Yes, held for 10 seconds
- ☐ 0 ....No, not held for 10 seconds **Record Result in Q8; End Balance Test; Go to Gait Speed Test**
- ☐ R ....Not attempted/Refused **End Balance Test; Go to Q9**

8. Number of seconds held, if less than 10: ____ . ____ ____ **Go to Gait Speed Test**

9. If participant did not attempt test, indicate reason with an **"X"**. **End Balance Test; Go to Gait Speed Test**

☐[1] ....Tried, but unable
☐[2] ....Participant could not hold position unassisted
☐[3] ....Not attempted, interviewer felt unsafe
☐[4] ....Not attempted, participant felt unsafe
☐[5] ....Participant unable to understand instructions
☐[6] ....Other (Please Specify)_________________________
☐[R] ....Participant Refused

Measure: Gait Speed Test

First Gait Speed

Interviewer Script: ***Now I am going to observe how you normally walk. If you use a cane or other walking aid and you feel you need it to walk a short distance, then you may use it.***

This is our walking course. I want you to walk to the other end of the course at your usual speed, just as if you were walking down the street to go to the store. *(Demonstrate the walk for the participant.)*

Walk all the way past the other end of the tape before you stop. I will walk with you. Do you feel this would be safe? *(Have the participant stand with both feet touching the starting line.)*

When I want you to start, I will say: "Ready, begin." *When the participant acknowledges this instruction say **"Ready, begin."***

- *Press the start/stop button to start the stopwatch when the participant steps over the starting line.*
- *Walk behind and to the side of the participant.*
- *Stop timing when one of the participant's feet is completely across the end line.*

---

10. Length of walk test course:

☐[1] ....4 Meters
☐[2] ....3 Meters (if 4 meter course is unavailable)

11. Time for 3 or 4 meters: ____ ____ . ____ ____ Seconds

12. If participant did not attempt test, indicate reason with an **"X":**

☐1 ....Tried, but unable
☐2 ....Participant could not walk unassisted
☐3 ....Not attempted, interviewer felt unsafe
☐4 ....Not attempted, participant felt unsafe
☐5 ....Participant unable to understand instructions
☐6 ....Other (Please Specify)__________________________
☐R ....Participant Refused

13. Aids used for first walk:

☐0 ....None
☐1 ....Cane
☐2 ....Other (Please Specify)___________________

Second Gait Speed

Interviewer Script: ***Now I want you to repeat the walk. Remember to walk at your usual pace, and go all the way past the other end of the course.***

Have the participant stand with both feet touching the starting line.

When I want you to start, I will say: "Ready, begin." *When the participant acknowledges this instruction say* ***"Ready, begin."***

- *Press the start/stop button to start the stopwatch when the participant steps over the starting line.*
- *Walk behind and to the side of the participant.*
- *Stop timing when one of the participant's feet is completely across the end line.*

---

14. Time for 3 or 4 meters: ____ ____ **.** ____ ____ Seconds

15. If participant did not attempt test, indicate reason with an **"X":**

☐1 ....Tried, but unable
☐2 ....Participant could not walk unassisted
☐3 ....Not attempted, interviewer felt unsafe
☐4 ....Not attempted, participant felt unsafe
☐5 ....Participant unable to understand instructions
☐6 ....Other (Please Specify)__________________________
☐R ....Participant Refused

16. Aids used for Second walk:

☐[0] ....None
☐[1] ....Cane
☐[2] ....Other (Please Specify)__________________

17. What is the time for the faster of the two walks? **(Record the shorter of the two times; if only one walk done, record that time)** ____ ____ . ____ ____

Measure: Chair Stand Test

<u>Single Chair Stand</u>

<u>Interviewer Script</u>: ***Let's do the last movement test. Do you think it would be safe for you to try to stand up from a chair?***

The next test measures the strength in your legs. *(Demonstrate and explain the procedure.)*

First, fold your arms across your chest and sit so that your feet are on the floor; then stand up keeping your arms folded across your chest.

Please stand up keeping your arms folded across your chest. *(Record result)*

If the participant cannot rise without using arms, say ***"Okay, try to stand up using your arms."*** *This is the end of the test. Record result onto the scoring page.*

---

18. Safe to stand without help?

☐[1] ....Yes
☐[0] ....No

19. Results:

☐[1] ....Participant stood without using arms **Go to Repeated Chair Stand Test**
☐[2] ....Participant used arms to stand **End Test; Proceed to Section B**
☐[0] ....Test not completed (0 points) **End Test; Go to Q20**

20. If participant did not attempt test, indicate reason with an **"X":**

- ☐[1] ....Tried, but unable
- ☐[2] ....Participant could not stand unassisted
- ☐[3] ....Not attempted, interviewer felt unsafe
- ☐[4] ....Not attempted, participant felt unsafe
- ☐[5] ....Participant unable to understand instructions
- ☐[6] ....Other (Please Specify)________________________
- ☐[R] ....Participant Refused

Interviewer: Skip to Q24

Repeated Chair Stand

Interviewer Script: ***Do you think it would be safe for you to try to stand up from a chair five times without using your arms?*** *(Demonstrate and explain the procedure.)*

Please stand up straight as QUICKLY as you can five times, without stopping in between. After standing up each time, sit down and then stand up again. Keep your arms folded across your chest. I'll be timing you with a stopwatch.

- *When the participant is properly seated, say: "**Ready? Stand.**" and begin timing.*
- *Count out loud as the participant rises each time, up to five times.*
- *Stop if participant becomes tired or short of breath during repeated chair stands.*
- *Stop the stopwatch when he/she has straightened up completely for the fifth time.*
- *Also stop:*
  - *If participant uses his/her arms.*
  - *After 1 minute, if participant has not completed all 5 rises*
  - *At your discretion, if concerned for participant's safety.*

If the participant stops and appears to be fatigued before completing the five stands, confirm this by asking ***"Can you continue?"***

---

21. Safe to stand five times?

- ☐[1] ....Yes
- ☐[0] ....No **Go to Q23**

22. Time to complete five stands? **(Only if participant completes 5 stands)**

____ ____ **.** ____ ____ Seconds

23. If participant did not attempt/complete test, indicate reason with an **"X":**

- ☐ 1 ....Tried, but unable
- ☐ 2 ....Participant could not stand unassisted
- ☐ 3 ....Not attempted, interviewer felt unsafe
- ☐ 4 ....Not attempted, participant felt unsafe
- ☐ 5 ....Participant unable to understand instructions
- ☐ 6 ....Other (Please Specify)_________________________
- ☐ R ....Participant Refused

B. GRIP STRENGTH

***Interviewer Script*: In this exercise, I am going to use this instrument to measure the strength in your hand. I am going to ask you some questions to determine which hand would be better to test.**

24. Are you right or left-handed?

- ☐ 1 ....Right
- ☐ 2 ....Left
- ☐ 3 ....Both

25. Which is your stronger hand?

- ☐ 1 ....Right **Determine safety for right hand; Go to Q26a**
- ☐ 2 ....Left **Determine safety for left hand; Go to Q27a**
- ☐ 3 ....Same **Determine safety for dominant hand (See Q24; Proceed to Q26a for right or Q27a for left). If neither hand is dominant, determine safety for right hand, Go to Q26a.**

If determining safety for right hand:

26a. Do you have severe pain or severe arthritis in your **right hand**?

- ☐ 1 ....Yes
- ☐ 0 ....No

26b. Have you had any surgery on your **right hand** or wrist in the past three months?

- ☐ 1 ....Yes
- ☐ 0 ....No

26c. Do you think you could safely squeeze this with your **right hand**, as hard as you can?

☐ 1 ....Yes **Test right hand; Go to Q28**
☐ 0 ....No **Do not test right hand, Go to Q27a to evaluate left hand**

If determining safety for left hand:

27a. Do you have severe pain or severe arthritis in your **left hand**?

☐ 1 ....Yes
☐ 0 ....No

27b. Have you had any surgery on your **left hand** or wrist in the past three months?

☐ 1 ....Yes **Do not test left hand, Go to Q26a to evaluate right hand, if not already done. If right hand not eligible, Go to Q28.**
☐ 0 ....No

27c. Do you think you could safely squeeze this with your **left hand**, as hard as you can?

☐ 1 ....Yes **Test left hand; Go to Q28**
☐ 0 ....No **Do not test left hand, Go to Q26a to evaluate right hand, if not already done. If right hand not eligible, Go to Q28.**

---

28. Which hand was tested?

☐ 1 ....Right
☐ 2 ....Left
☐ 3 ....Neither **END TEST**

Interviewer Script: ***I'd like you to sit down and take your right/left arm and please place it next to your body and bend your elbow at a 90° angle. Grip the two bars in your hand, like this. You need to slowly squeeze the bars as hard as you can."*** *(Hand the Dynamometer to the participant. Adjust if needed.)*

Now try it once just to get the feel for it. For this practice, just squeeze gently. It won't feel like the bars are moving, but your strength will be recorded. Are the bars the right distance apart for a comfortable grip?

Show dial to participant.

29. Dynamometer Setting:

☐[1] ....One
☐[2] ....Two
☐[3] ....Three
☐[4] ....Four
☐[5] ....Five

We'll do this twice. This time it counts, so when I say "Squeeze", squeeze as hard as you can. Ready? Squeeze! Squeeze! Squeeze! Okay now, STOP.

30. Trial 1:

____ ____ kg

☐[U] ....Unable to test/exclusion
☐[C] ....Participant unable to understand instructions
☐[R] ....Refused

Examiner Note: Wait 10 seconds before second trial.

Now, one more time. Squeeze as hard as you can. Ready? Squeeze! Squeeze! Squeeze! Okay now, STOP.

31. Trial 2:

____ ____ kg

☐[U] ....Unable to test/exclusion
☐[C] ....Participant unable to understand instructions
☐[R] ....Refused

LONG LIFE FAMILY STUDY	**(Affix Label Here)**<br>Participant ID: ____________________<br>Participant Name Code: _____________	Date Form Filled Out:<br>☐☐☐☐☐☐☐☐☐<br>d d M M M y y y y<br>(e.g., 10JUN2005)<br>Interviewer Code: ☐☐☐<br>Circle Field Center Location:<br>**BU CU DK UP**

Blood Pressure, Heart Rate, Height, Weight and Waist Circumference

***Interviewer*: Set equipment at 1 minute intervals, allowing for a 1 minute break between measurements.**

MEASURE: Blood Pressure

1a. Record BPTru 300 machine number: ____ ____ ____

1b. Arm Circumference: ____ ____ . ____ cm

1c. Cuff Size:

☐1 ....Child (13-17.99 cm)
☐2 ....Small (18-25.99 cm)
☐3 ....Regular (26-33.99 cm)
☐4 ....Large (34-42.99 cm)
☐5 ....X-Large (Thigh) (43-52 cm)

2a. Which arm was used?

☐1 ....Right
☐2 ....Left

2b. Cuff Placement:

☐1 ....Upper Arm
☐2 ....Forearm

2c. ***Interviewer***: If right arm was not used, please explain why the right arm was not used for this measurement: ____

____

Participant ID: ______________________________ **Participant Name Code:** ____________________

Sitting Blood Pressure Measurement #1:

3a. **Systolic**: ___ ___ ___ mmHg 3c. **Pulse Rate:** ____ ____ ____

3b. **Diastolic**: ___ ___ ___ mmHg

3d. Comments required for missing or unusual values: ____

____

Sitting Blood Pressure Measurement #2:

4a. **Systolic**: ___ ___ ___ mmHg 4c. **Pulse Rate:** ____ ____ ____

4b. **Diastolic**: ___ ___ ___ mmHg

4d. Comments required for missing or unusual values: ____

____

Sitting Blood Pressure Measurement #3:

5a. **Systolic**: ___ ___ ___ mmHg 5c. **Pulse Rate:** ____ ____ ____

5b. **Diastolic**: ___ ___ ___ mmHg

5d. Comments required for missing or unusual values: ____

____

MEASURE: Ankle-Arm Blood Pressure

6a. Blood Pressure: Right Arm: ____ ____ ____ (from 1st Sitting Systolic Blood Pressure Reading)

Left Arm: ____ ____ ____ (take 1 Reading)

If greater than 10mmHg difference, use the arm with the higher Blood Pressure.

6b. Which arm was used?

☐1 ....Right
☐2 ....Left

Systolic Measurement #1:

7a. Brachial (Arm): ___ ___ ___ mmHg

7b. Right Posterior Tibial: ___ ___ ___ mmHg

7c. Left Posterior Tibial: ___ ___ ___ mmHg

Systolic Measurement #2:

8a. Left Posterior Tibial: ___ ___ ___ mmHg

8b. Right Posterior Tibial: ___ ___ ___ mmHg

8c. Brachial (Arm): ___ ___ ___ mmHg

9a. Was the dosalis pedis pulse used?

☐ 1 ....Yes **Go to Q9b**
☐ 0 ....No **Go to Q10a**

9b. If yes, in which leg?

☐ 1 ....Right
☐ 2 ....Left
☐ 3 ....Both

10a. Was the ankle-arm blood pressure measurement completed successfully?

☐ 1 ....Yes **Go to 10b**
☐ 0 ....No **Go to Q10g**

10b. Average Brachial Systolic Pressure:

Brachial-1: ___ ___ ___ + Brachial-2 ___ ___ ___ = ___ ___ ___ / 2 = ___ ___ ___

10c. Average Right Posterior Tibial Systolic Blood Presure:

R_Posterior Tibial-1 ___ ___ ___ + R_Posterior Tibial-2 ___ ___ ___ = ___ ___ ___ / 2

10d. Average Left Posterior Tibial Systolic Blood Presure:

L_Posterior Tibial-1 ___ ___ ___ + L_Posterior Tibial-2 ___ ___ ___ = ___ ___ ___ / 2

10e. Ankle-Arm Blood Pressure Ratio for Right Side:

Average R_Posterior Tibial / Average Brachial = ___ ___ ___

10f. Ankle-Arm Blood Pressure Ratio for Left Side:

Average L_Posterior Tibial / Average Brachial = ___ ___ ___

10g. If No, why wasn't the procedure completed? **(Please "X" all that apply)**

Left Leg:

- ☐ 1 ....Unable to Occlude
- ☐ 1 ....Ulceration
- ☐ 1 ....Amputation
- ☐ 1 ....Unable to Locate Distal Pulse
- ☐ 1 ....Too Painful
- ☐ 1 ....Unable to Lie in Semi-Recumbent Position
- ☐ 1 ....Participant Refused
- ☐ 1 ....Unable to Follow Instructions
- ☐ 1 ....Other, Please Specify: ___________________________

Right Leg:

- ☐ 1 ....Unable to Occlude
- ☐ 1 ....Ulceration
- ☐ 1 ....Amputation
- ☐ 1 ....Unable to Locate Distal Pulse
- ☐ 1 ....Too Painful
- ☐ 1 ....Unable to Lie in Semi-Recumbent Position
- ☐ 1 ....Participant Refused
- ☐ 1 ....Unable to Follow Instructions
- ☐ 1 ....Other, Please Specify: ___________________________

MEASURE: Anthropometry

Examiner Script: *Now I am going to measure your standing height. Please remove your shoes. Stand with your feet flat on the floor, heels together, with heels, hips, shoulders directly against the wall."*

Standing Height:

If participant is unable to sufficiently follow instructions to complete this measurement, please enter "C" for measurement 1 and proceed to question 12a.

11a. Measurement #1: ___ ___ ___ . ___ cm

11b. Measurement #2: ___ ___ ___ . ___ cm

11c. Please calculate the difference between Q11a and Q11b: ___ ___ ___ . ___ cm

If difference between Measurement #1 and #2 is ≥ 0.4 cm, proceed with Measurements #3 and #4.

11e. Measurement #3: ___ ___ ___ . ___ cm

11f. Measurement #4: ___ ___ ___ . ___ cm

11g. Is Participant standing sideways due to kyphosis (stooped posture)?

☐ 1 ....Yes
☐ 0 ....No

Arm Span:

Examiner Script: *In this test I will measure the length of your arm span from fingertip to fingertip. Please stand with your back to the wall and fully extend your right/left arm at shoulder height, just until your fingertip touches the corner of the wall. Now extend your other arm also at shoulder height. I will place a piece of tape at the outstretched fingertip and mark the tape.*

If participant is unable to sufficiently follow instructions to complete this measurement, please enter "C" for measurement 1 and proceed to question 13a.

12a. Measurement: ___ ___ ___ . ___ cm

12b. How tall were you as a young adult, that is, in your mid-twenties?

___ feet ___ ___ inches or ___ ___ ___ . ___ cm

Sitting Height:

Examiner Script: *Please sit on this seat with your knees facing forward. Place your hand on your thighs in a cross-handed position. Sit up as straight as possible with your buttocks and back touching the backboard. Do not support your body weight on your feet. All your weight should be on the buttocks. Relax the muscles of your legs and buttocks.*

If participant is unable to sufficiently follow instructions to complete this measurement, please enter "C" for measurement 1 and proceed to question 13g.

13a. Measurement #1: ___ ___ ___ . ___ cm

13b. Measurement #2: ___ ___ ___ . ___ cm

13c. Please calculate the difference between Q13a and Q13b: ___ ___ ___ . ___ cm

If difference between Measurement #1 and #2 is ≥ 0.4 cm, proceed with Measurements #3 and #4.

13e. Measurement #3: ___ ___ ___ . ___ cm

13f. Measurement #4: ___ ___ ___ . ___ cm

13g. Is Participant sitting sideways due to kyphosis (stooped posture)?

☐ 1 ....Yes
☐ 0 ....No

13h. Was the designated seat used?

☐ 1 ....Yes **Go to Q14a below**
☐ 0 ....No **Go to Q13i below**

13i. Seat Height? ___ ___ ___ . ___ cm

Knee Height:

Examiner Script: *Please remove your shoes and socks from your right foot and roll up your pant leg past your knee. In this test, we will measure the length of your leg from heel to knee. Place the heel of your right foot on this measuring caliper and this other arm will rest on your knee.*

If participant is unable to sufficiently follow instructions to complete this measurement, please enter "C" for measurement 1 and proceed to question 15.

14a. ☐ 1 ....Right Knee **Test Right Knee if able. If not, test Left Knee**
☐ 2 ....Left Knee
☐ 3 ....Unable to bend either Knee
☐ 4 ....Other (Please Specify)__________________
☐ R ....Refused

14b. Measurement #1: ___ ___ ___ . ___ cm

14c. Measurement #2: ___ ___ ___ . ___ cm

Examiner Script: *In order to measure your weight, please remove your shoes and heavy jewelry, and empty your pockets. Please step forward onto the center of the scale.*

If participant is unable to sufficiently follow instructions to complete this measurement, please enter "C" for weight.

15. Weight: ___ ___ ___ . ___ kg

<u>Abdominal Circumference</u>:

Examiner Script: *I'd like to take a measurement around your middle at your bellybutton. I may need to move some of your clothing out of the way. Breathe normally. Don't hold your stomach in, just relax.*

If participant is unable to sufficiently follow instructions to complete this measurement, please enter "C" for measurement 1 and exit the form.

16a. Measurement #1: ___ ___ ___ . ___ cm

16b. Measurement #2: ___ ___ ___ . ___ cm

16c. Please calculate the difference between Q11a and Q11b: ___ ___ ___ . ___ cm

If difference between Measurement #1 and #2 is > 1cm, proceed with Measurements #3 and #4.

16d. Measurement #3: ___ ___ ___ . ___ cm

16e. Measurement #4: ___ ___ ___ . ___ cm

16f. Was circumference at the umbilicus obstructed?

☐ 1 ....Yes **Go to Q16h**
☐ 0 ....No **END**

16g. If Yes, please explain: ____

LONG LIFE FAMILY STUDY

(Affix Label Here)

Participant ID: ____________________

Participant Name Code: _____________

Date Form Filled Out: ☐☐☐☐☐☐☐☐☐
d d M M M y y y y
(e.g., 10JUN2005)

Interviewer Code: ☐☐☐

Circle Field Center Location:

BU **CU** **DK** **UP**

Spirometry

Please Mark the Appropriate Box Below:

☐1 ....This Form was Administered In-Person by Study Personnel
☐2 ....This Form was Administered via Telephone by Study Personnel

---

1a. Do you usually have a cough, on most days, for 3 or more months during the year?

☐1 ....Yes
☐0 ....No **Go to Q2**
☐D ....Don't Know **Go to Q2**
☐R ....Refused **Go to Q2**

1b. For how many years have you had this cough? ____ ____ Years

---

2a. Do you usually bring up phlegm from your chest, on most days, for 3 or more months during the year?

☐1 ....Yes
☐0 ....No **Go to Q3**
☐D ....Don't Know **Go to Q3**
☐R ....Refused **Go to Q3**

2b. For how many years have you brough up phlegm from your chest like this? ____ ____ Years

---

3a. Do you ever use oxygen therapy at home?

☐1 ....Yes
☐0 ....No **Go to Q4**
☐D ....Don't Know **Go to Q4**
☐R ....Refused **Go to Q4**

3b. When do you use it?

☐[1] ....Most of the Time
☐[2] ....Only at Night
☐[3] ....Only with Exercise

Please answer the following:

4a. Does your chest ever sound wheezy or whistling when you have a cold?

☐[1] ....Yes
☐[0] ....No
☐[D] ....Don't Know
☐[R] ....Refused

4b. Does your chest ever sound wheezy or whistling apart from colds?

☐[1] ....Yes
☐[0] ....No
☐[D] ....Don't Know
☐[R] ....Refused

4c. Does your chest sound wheezy or whistling most days or nights?

☐[1] ....Yes
☐[0] ....No
☐[D] ....Don't Know
☐[R] ....Refused

***Interviewer*: If participant answered "YES" to ONE or MORE PARTS (4a, 4b, 4c) of this question, complete Q4d below.**

4d. For how many years has this been present? ____ ____ Years

---

5. Is the participant's systolic blood pressure greater than 210 mm HG or diastolic blood pressure greater than 120 mm HG?

☐[1] ....Yes **Do Not Test**
☐[0] ....No

---

6. Have you been told that you had a heart attack or stroke in the last three months, or have you had eye, chest, or stomach surgery in the last three months?

- ☐ 1 ....Yes **Do Not Test**
- ☐ 0 ....No
- ☐ D ....Don't Know **Do Not Test**
- ☐ R ....Refused **Do Not Test**

---

7. Have you had any significant problems doing Spirometry in the past?

- ☐ 1 ....Yes **Inquire about problems. Do Not Test if problems significant (see MOP for definition of significant); proceed with testing if problems not significant.**
- ☐ 0 ....No
- ☐ D ....Don't Know
- ☐ R ....Refused

---

8. Have you had a respiratory infection in the past 2 weeks, for instance, a cold, flu, bronchitis, or pneumonia? ******Note This Question Is For Informational Purposes Only And Is Not An Exclusion Criteria.***

- ☐ 1 ....Yes
- ☐ 0 ....No
- ☐ D ....Don't Know
- ☐ R ....Refused

9a. Have you taken any inhalers, "puffers" or inhaled corticosteroids in the last three days (for example, albuterol [Ventolin, Proventil], salmetrol [Serevent], ipratropium [Atrovent, Combivent], tiotropium [Spiriva], Advair, Aerobid, Azmacort, Beclovent, Flovent, Pulmicort, or Vanceril?

- ☐ 1 ....Yes
- ☐ 0 ....No **Go to Q10**
- ☐ D ....Don't Know **Go to Q10**
- ☐ R ....Refused **Go to Q10**

9b. Please complete the table below:

	Last Taken			Time last taken								
**Name of Medication**	**Day before yesterday**	**Yesterday**	**Today**									
						:			:		M	
						:			:		M	
						:			:		M	
						:			:		M	

10. Did you have any caffeinated coffee, tea, or cola, or other caffeinated drink, in the past 2 hours?

☐[1] ....Yes
☐[0] ....No
☐[D] ....Don't Know
☐[R] ....Refused

11. Did you smoke a cigarette, pipe or cigar during the last hour?

☐[1] ....Yes
☐[0] ....No
☐[D] ....Don't Know
☐[R] ....Refused

***Interviewer**: At this time, please conduct Spirometry testing on eligibile participants.*

Interviewer Script: *Place the Spirette on the top of your tongue, seal it with your lips, but don't bite down on it. Take a great big deep breath of air as far as you can inhale. BLAST your air into the tube as hard and fast as you can. Keep blosing out until I tell you to stop. Pretend to blow out all the candles on a birthday cake with one breath.*

12a. Was Spirometry completed?

☐[1] ....Yes **Go to Q12b**
☐[0] ....No **Go to Q12c**

12b. Record results of Spirometry*:

**FEV6 Best Value**	______________ liters
**FEV6 % Predicted**	______________ %
**FEV_1 Best Value**	______________ liters
**FEV_1 % Predicted**	______________ %
**% FEV_1**	______________ %

* *If FEV6% predicted and FEV_1% predicted do not appear on spirometer, still record other three numbers.*

12c. Specify the reasons(s) why Spirometry was not completed. Select one or more from the provided options:

- ☐ 1 ....Physically Unable
- ☐ 2 ....Cognitively Unable
- ☐ 3 ....Equipment Problem
- ☐ 4 ....Other (Please Specify)_________________________
- ☐ C ....Unable to Follow Instructions
- ☐ R ....Refused

LONG LIFE FAMILY STUDY

(Affix Label Here)

Participant ID: ____________________

Participant Name Code: ____________

Date Form Filled Out: ☐☐☐☐☐☐☐☐☐

d d M M M y y y y

(e.g., 10JUN2005)

Interviewer Code: ☐☐☐

Circle Field Center Location:

BU CU DK UP

Mood/Personality

CES-D/NEO Five-Factor Inventory

Please Mark the Appropriate Box Below:

- ☐1 ....This Form was Administered via a DFR/Proxy
- ☐2 ....This Form was Administered In-Person by Study Personnel
- ☐3 ....This Form was Administered via Telephone by Study Personnel
- ☐4 ....This Form was Mailed and Self-Administered by Participant
- ☐5 ....This Form was Administered by Other: ____________________

CES-D: Participant Version

I am going to read you a list of ways you might have felt or behaved during the past week or past seven days. After I read an item, please look at this card and tell me how often you have felt this way during the past week.

CES-D: Proxy Version

I am going to read you a list of ways [insert Name Here] *might have felt or behaved during the past week or past seven days. After I read an item, please look at this card and tell me how often* [insert Name Here] *has felt this way during the past week. For all questions below, use the following format:* "During the past week, was "JOHN" bothered by things that usually don't bother him. How often did he feel that way?"

Refer to Card in Appendix B

P1. During the past week, I was bothered by things that usually don't bother me. How often did you feel this way?

***Interviewer*: Read Responses.**

- ☐0 ....Rarely or none of the time (less than 1 day)
- ☐1 ....Some or a little of the time (1 to 2 days)
- ☐2 ....A moderate amount of time (3 to 4 days)
- ☐3 ....Most of the time
- ☐D ....Don't Know
- ☐R ....Refused

P2. I had trouble keeping my mind on what I was doing:

- ☐ 0 ....Rarely or none of the time (less than 1 day)
- ☐ 1 ....Some or a little of the time (1 to 2 days)
- ☐ 2 ....A moderate amount of time (3 to 4 days)
- ☐ 3 ....Most of the time
- ☐ D ....Don't Know
- ☐ R ....Refused

P3. I felt that everything I did was an effort:

- ☐ 0 ....Rarely or none of the time (less than 1 day)
- ☐ 1 ....Some or a little of the time (1 to 2 days)
- ☐ 2 ....A moderate amount of time (3 to 4 days)
- ☐ 3 ....Most of the time
- ☐ D ....Don't Know
- ☐ R ....Refused

P4. I felt depressed:

- ☐ 0 ....Rarely or none of the time (less than 1 day)
- ☐ 1 ....Some or a little of the time (1 to 2 days)
- ☐ 2 ....A moderate amount of time (3 to 4 days)
- ☐ 3 ....Most of the time
- ☐ D ....Don't Know
- ☐ R ....Refused

P5. I felt hopeful about the future:

- ☐ 3 ....Rarely or none of the time (less than 1 day)
- ☐ 2 ....Some or a little of the time (1 to 2 days)
- ☐ 1 ....A moderate amount of time (3 to 4 days)
- ☐ 0 ....Most of the time
- ☐ D ....Don't Know
- ☐ R ....Refused

P6. I felt fearful:

- ☐ 0 ....Rarely or none of the time (less than 1 day)
- ☐ 1 ....Some or a little of the time (1 to 2 days)
- ☐ 2 ....A moderate amount of time (3 to 4 days)
- ☐ 3 ....Most of the time
- ☐ D ....Don't Know
- ☐ R ....Refused

P7. My sleep was restless:

- ☐ 0 ....Rarely or none of the time (less than 1 day)
- ☐ 1 ....Some or a little of the time (1 to 2 days)
- ☐ 2 ....A moderate amount of time (3 to 4 days)
- ☐ 3 ....Most of the time
- ☐ D ....Don't Know
- ☐ R ....Refused

P8. I was happy:

- ☐ 3 ....Rarely or none of the time (less than 1 day)
- ☐ 2 ....Some or a little of the time (1 to 2 days)
- ☐ 1 ....A moderate amount of time (3 to 4 days)
- ☐ 0 ....Most of the time
- ☐ D ....Don't Know
- ☐ R ....Refused

P9. I felt lonely:

- ☐ 0 ....Rarely or none of the time (less than 1 day)
- ☐ 1 ....Some or a little of the time (1 to 2 days)
- ☐ 2 ....A moderate amount of time (3 to 4 days)
- ☐ 3 ....Most of the time
- ☐ D ....Don't Know
- ☐ R ....Refused

P10. I could not get going:

- ☐ 0 ....Rarely or none of the time (less than 1 day)
- ☐ 1 ....Some or a little of the time (1 to 2 days)
- ☐ 2 ....A moderate amount of time (3 to 4 days)
- ☐ 3 ....Most of the time
- ☐ D ....Don't Know
- ☐ R ....Refused

NEO Five-Factor Inventory (N and C Items)

Participant Instructions

***Interviewer Script*: "Now I'm going to read some statements. Listen Carefully. For each statement, choose the response on this card that best represents your opinion.**

Proxy Instructions

***Interviewer Script*: "Now I'm going to read some statements. Listen Carefully. For each statement, choose the response on this card that best represents your opinion regarding [insert Name Here].**

Refer to Card in Appendix B

Choose:

- ***Strongly Disagree*** *if the statement is definitely false for you.*
- ***Disagree*** *if you feel the statement is mostly false.*
- ***Neutral*** *if you cannot decide.*
- ***Agree*** *if the statement is mostly true.*
- ***Strongly Agree*** *if the statement is definitely true for you.*

For example, if the statement was "I laugh easily," and this was definitely true for you, you would say "Strongly Agree". Provide only one response for each statement. Respond to all of the statements. Please rate yourself as you are today (not how you used to be).

***Examiner Note*: If the participant is not familiar with a word used in a statement, you may give them a synonym for the unfamiliar word. Appropriate substitutions are listed in the operations manual.**

P11. I am not a worrier.

☐1 ....Strongly Disagree
☐2 ....Disagree
☐3 ....Neutral
☐4 ....Agree
☐5 ....Strongly Agree

P12. I keep my belongings neat and clean.

☐1 ....Strongly Disagree
☐2 ....Disagree
☐3 ....Neutral
☐4 ....Agree
☐5 ....Strongly Agree

P13. I often feel inferior to others.

Interviewer: ***If respondent has difficulty understanding item 13, you may read the following:*** *"I often feel not as good as or less important than others."*

☐[1] ....Strongly Disagree
☐[2] ....Disagree
☐[3] ....Neutral
☐[4] ....Agree
☐[5] ....Strongly Agree

P14. I'm pretty good about pacing myself so as to get things done on time.

Interviewer: ***If respondent has difficulty understanding item 14, you may read the following:*** *"I'm pretty good about giving myself enough time or taking the right amount of time so as to get things done on time."*

☐[1] ....Strongly Disagree
☐[2] ....Disagree
☐[3] ....Neutral
☐[4] ....Agree
☐[5] ....Strongly Agree

P15. When I'm under a great deal of stress, sometimes I feel like I'm going to pieces.

☐[1] ....Strongly Disagree
☐[2] ....Disagree
☐[3] ....Neutral
☐[4] ....Agree
☐[5] ....Strongly Agree

P16. I am not a very methodical person.

Interviewer: ***If respondent has difficulty understanding item 16, you may read the following:*** *"I am not a very planful, orderly person."*

☐[1] ....Strongly Disagree
☐[2] ....Disagree
☐[3] ....Neutral
☐[4] ....Agree
☐[5] ....Strongly Agree

P17. I rarely feel lonely or blue.

- ☐[1] ....Strongly Disagree
- ☐[2] ....Disagree
- ☐[3] ....Neutral
- ☐[4] ....Agree
- ☐[5] ....Strongly Agree

P18. I try to perform all the tasks assigned to me conscientiously.

***Interviewer**: If respondent has difficulty understanding item 18, you may read the following:* *"I try to perform all the tasks assigned to me carefully."*

- ☐[1] ....Strongly Disagree
- ☐[2] ....Disagree
- ☐[3] ....Neutral
- ☐[4] ....Agree
- ☐[5] ....Strongly Agree

P19. I often feel tense and jittery.

- ☐[1] ....Strongly Disagree
- ☐[2] ....Disagree
- ☐[3] ....Neutral
- ☐[4] ....Agree
- ☐[5] ....Strongly Agree

P20. I have a clear set of goals and work toward them in an orderly fashion.

***Interviewer**: If respondent has difficulty understanding item 20, you may read the following:* *"I have a clear set of things I want to do, and I work toward them in a certain order."*

- ☐[1] ....Strongly Disagree
- ☐[2] ....Disagree
- ☐[3] ....Neutral
- ☐[4] ....Agree
- ☐[5] ....Strongly Agree

P21. Sometimes I feel completely worthless.

- ☐[1] ....Strongly Disagree
- ☐[2] ....Disagree
- ☐[3] ....Neutral
- ☐[4] ....Agree
- ☐[5] ....Strongly Agree

P22. I waste a lot of time before settling down to work.

- ☐ 1 ....Strongly Disagree
- ☐ 2 ....Disagree
- ☐ 3 ....Neutral
- ☐ 4 ....Agree
- ☐ 5 ....Strongly Agree

P23. I rarely feel fearful or anxious.

- ☐ 1 ....Strongly Disagree
- ☐ 2 ....Disagree
- ☐ 3 ....Neutral
- ☐ 4 ....Agree
- ☐ 5 ....Strongly Agree

P24. I work hard to accomplish my goals.

- ☐ 1 ....Strongly Disagree
- ☐ 2 ....Disagree
- ☐ 3 ....Neutral
- ☐ 4 ....Agree
- ☐ 5 ....Strongly Agree

P25. I often get angry at the way people treat me.

- ☐ 1 ....Strongly Disagree
- ☐ 2 ....Disagree
- ☐ 3 ....Neutral
- ☐ 4 ....Agree
- ☐ 5 ....Strongly Agree

P26. When I make a commitment, I can always be counted on to follow through.

Interviewer**:** ***If respondent has difficulty understanding item 26, you may read the following:*** *"When I say I'll do something, I do it."*

- ☐ 1 ....Strongly Disagree
- ☐ 2 ....Disagree
- ☐ 3 ....Neutral
- ☐ 4 ....Agree
- ☐ 5 ....Strongly Agree

P27. Too often, when things go wrong, I get discouraged and feel like giving up.

- ☐[1] ....Strongly Disagree
- ☐[2] ....Disagree
- ☐[3] ....Neutral
- ☐[4] ....Agree
- ☐[5] ....Strongly Agree

P28. Sometimes I'm not as dependable or reliable as I should be.

- ☐[1] ....Strongly Disagree
- ☐[2] ....Disagree
- ☐[3] ....Neutral
- ☐[4] ....Agree
- ☐[5] ....Strongly Agree

P29. I am seldom sad or depressed.

<u>Interviewer</u>**: *If respondent has difficulty understanding item 29, you may read the following:*** *"I am not often sad or depressed."*

- ☐[1] ....Strongly Disagree
- ☐[2] ....Disagree
- ☐[3] ....Neutral
- ☐[4] ....Agree
- ☐[5] ....Strongly Agree

P30. I am a productive person who always gets the job done.

- ☐[1] ....Strongly Disagree
- ☐[2] ....Disagree
- ☐[3] ....Neutral
- ☐[4] ....Agree
- ☐[5] ....Strongly Agree

P31. I often feel helpless and want someone else to solve my problems.

- ☐[1] ....Strongly Disagree
- ☐[2] ....Disagree
- ☐[3] ....Neutral
- ☐[4] ....Agree
- ☐[5] ....Strongly Agree

P32. I never seem to be able to get organized.

☐1 ....Strongly Disagree
☐2 ....Disagree
☐3 ....Neutral
☐4 ....Agree
☐5 ....Strongly Agree

P33. At times, I have been so ashamed I just wanted to hide.

☐1 ....Strongly Disagree
☐2 ....Disagree
☐3 ....Neutral
☐4 ....Agree
☐5 ....Strongly Agree

P34. I strive for excellence in everything I do.

☐1 ....Strongly Disagree
☐2 ....Disagree
☐3 ....Neutral
☐4 ....Agree
☐5 ....Strongly Agree

Alternative phrasings adapted from: Markey PM, Markey CN, Tinsley BJ, Ericksen AJ. A preliminary validation of preadolescents' self-reports using the Five-Factor Model of personality JOURNAL OF RESEARCH IN PERSONALITY 36 (2): 173-181 APR 2002

Reproduced with permission of
Psychological Assessment Resources (PAR)
P.O. Box 998, Odessa, FL 33556

LONG LIFE FAMILY STUDY

(Affix Label Here)

Participant ID: ____________________

Participant Name Code: _____________

Date Form Filled Out: ☐☐☐☐☐☐☐☐☐

d d M M M y y y y

(e.g., 10JUN2005)

Interviewer Code: ☐☐☐

Circle Field Center Location:

BU **CU** **DK** **UP**

Family Structure Worksheet

Public reporting burden for this collection of information is estimated to average 8 minutes per response, including the time for reviewing instructions, searching existing data sources, gathering and maintaining the data needed and completing and reviewing the collection of this information. Send comments regarding this burden estimate or any other aspect of this collection of information, including suggestions for reducing this burden, to: PHS Reports Clearance Officer, Rm. 721-B, Humphrey Building, 200 Independence Ave., SW, Washington DC 20201, Attn: PRA; and to the Office of Management and Budget, Paperwork Reduction Project (0925-0412), Washington, DC 20503. Do not return completed forms to either of these addresses.

KEY

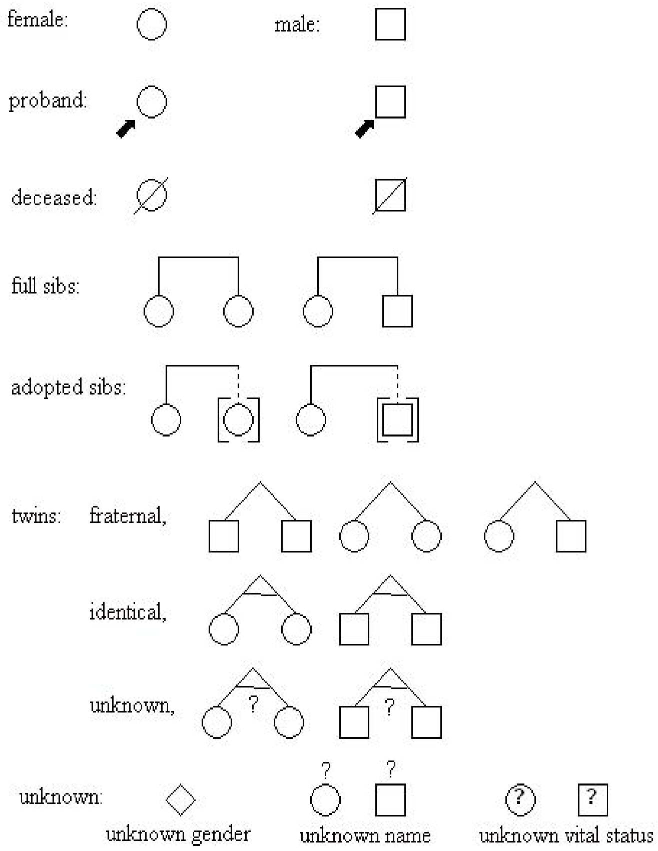

Name of Person who Completed the PIF Form:

Proband: ______________________________

Index Person: ___________________________

Comments: _____________________________

____

____

____

____

____

____

____

____

Participant ID: ______________________ Participant Name Code: ______________________

Family Structure Worksheet
Confidential

Generation

**I.**	
**II.**	
**III.**	

Comments: ____

____

____

____

LONG LIFE FAMILY STUDY

(Affix Label Here)

Participant ID: ____________________

Participant Name Code: ____________

Date Form Filled Out: ☐☐☐☐☐☐☐☐☐

d d M M M y y y y
(e.g., 10JUN2005)

Interviewer Code: ☐☐☐

Circle Field Center Location:

BU CU DK UP

Blood Collection

Venipuncture Form

INSTRUCTIONS: Verify the participant's name and ID before beginning the interview or procedure. If a number or response is entered incorrectly, mark through the incorrect entry with an “X”. Enter the correct entry clearly above the incorrect entry. Circle the correct response or clearly record the corrected value above the incorrect entry.

Special Instructions:

1. Be sure you have a frozen gel pack in the Styrofoam shipping box before leaving the Field Center.
2. Collect six (6) tubes provided in the following order ***(please write the date/time of blood collection on the label of the first tube)***:
   (a) #1 - blue/black topped CPT,
   (b) #2 - red/gray topped SST1,
   (c) #3 - lavender topped EDTA,
   (d) #4 - blue topped Sodium Citrate,
   (e) #5 - red topped PAXgene,
   (f) #6 - red/gray topped SST2
3. Be sure to hold the PAXgene tube vertically below the level of the participant’s arm during collection to avoid backflow from the tube.
4. Mix all tubes immediately after blood collection by gently inverting each tube eight times.
5. Return any totally unused tubes to the lab in the shipping container.
6. Check the Saturday Delivery box on the FedEx billable stamp (air bill) for shipments sent on Friday.

Proceed with the Screening Questions on Page 2 prior to beginning the blood draw.

Phlebotomy Screening

These phlebotomy screening questions **WILL NOT be entered into the DES System**

Date: ____ ____ / ____ ____ ____ / ____ ____ ____ ____

ID Number: ________________________________ Acrostic: ______________________________

(a) Have you ever had a radical mastectomy or other surgery where lymph nodes were removed from your armpits? ☐1 Yes ☐0 No

(b) Have you ever had a graft or shunt for kidney dialysis? ☐1 Yes ☐0 No

(c) Do you have a history of anemia? ☐1 Yes ☐0 No

- If "Yes", when were you told about this? ***[Interviewer Note:*** *The purpose of this question is to understand how long the participant has known this]* ________________________________
- Are you treated for the anemia? ☐1 Yes ☐0 No
- If "Yes", what treatment do you receive? For example, iron supplements, Vitamin B-12 shots, etc.? ________________________________

(d) Are you currently receiving chemotherapy? ☐1 Yes ☐0 No

- If "Yes", how often do you receive treatments? ________________________________
- What was the date of your last treatment? ___ ___ / ___ ___ ___ / ___ ___ ___ ___

(e) Have you had surgery in the past 3 months? ☐1 Yes ☐0 No

- If "Yes", what type of surgery? ________________________________
- Did you receive any transfusions? ☐1 Yes ☐0 No
- Did your doctor tell you that your blood count was low? ☐1 Yes ☐0 No
- If "Yes", are you currently receiving treatment? ☐1 Yes ☐0 No
- If "Yes", how are you being treated? ________________________________

A. BLOOD DRAWING

1. Do you have any bleeding disorders?

   ☐[1] ....Yes **If Yes, Review Special Precautions and Specify in Q17**
   ☐[0] ....No

2. On which day did you last eat or drink anything except water: today, yesterday, or the day before yesterday?

   ☐[1] ....Today
   ☐[2] ....Yesterday
   ☐[3] ....Before Yesterday

3. And at what time was that?

   ____ ____ **:** ____ ____ AM / PM **(Circle One)**

4. Number of venipuncture attempts: ____ ____

5. Time venipuncture ended?

   ____ ____ **:** ____ ____ AM / PM **(Circle One)**

6. Tubes collected: (X all that apply)

   ☐[1] ....CPT
   ☐[1] SST1
   ☐[1] ....EDTA
   ☐[1] ....Sodium Citrate
   ☐[1] ....PAXGene
   ☐[1] SST2

7. Code number of Phlebotomist: ____ ____ ____

B. BLOOD PROCESSING & SHIPPING

8. Is this a local health care provider blood collection by non-LLFS staff (i.e. following the instructions of the Local Health Care Provider Blood Collection protocol in Appendix 3 of Chapter 20 of the Manual of Procedures)?

   ☐[1] ....Yes
   ☐[0] ....No

9. Time at which SST1 and SST2 tubes were spun? **(Allow SST tubes to clot for 30-45 minutes before centrifuging at 1200 rcf.)**

____ ____ : ____ ____ AM / PM. **(Circle One)**

10. Date specimen tubes were shipped? ____ ____ / ____ ____ ____ / ____ ____ ____ ____
Day Month Year

11. Time specimen tubes were shipped?

____ ____ : ____ ____ AM / PM. **(Circle One)**

12. Code number of technician processing the blood: ____ ____ ____

C. BLOOD DRAWING INCIDENTS: This log is completed to document problems with the venipuncture. Place an "X" in the boxes corresponding to the tubes in which blood drawing problem(s) occurred. If a problem other than those listed below occurred, please indicate in Item 17 below.

		**TUBES**					
		**CPT**	**SST1**	**EDTA**	**Na Citrate**	**PAXGene**	**SST2**
13.	Sample Not Drawn						
14.	Partial Sample Drawn						
15.	Prolonged Tourniquet						
16.	Broken Tube						

17. Comments on blood drawing/centrifuging/shipping: ____

____

____

____

D. ALTERNATIVE DNA COLLECTION: This section is completed only if the Oragene collection cup is used for the DNA sample collection. The Oragene cup is used if only tubes #1 and #2 were collected or if blood collection attempts were unsuccessful.

18. If blood was not collected or insufficient blood was collected, was saliva collected in an Oragene collection cup?

☐ 1 ....Yes **Go to Q19**
☐ 0 ....No **End Interview**

19. Was at least 2 mL of saliva collected?

☐[1] ....Yes
☐[0] ....No

If no, estimate the volume saliva collected. ____________ mL

20. Date Oragene was shipped? ____ ____ / ____ ____ ____ / ____ ____ ____ ____
Day Month Year

21. Time Oragene was shipped?

____ ____ **:** ____ ____ AM / PM **(Circle One)**

LONG LIFE FAMILY STUDY	**(Affix Label Here)**<br><br>Participant ID: ______________<br><br>Participant Name Code: __________	Date Form Filled Out:<br>☐☐☐☐☐☐☐☐☐<br>d d M M M y y y y<br>(e.g., 10JUN2005)<br><br>Interviewer Code: ☐☐☐<br><br>Circle Field Center Location:<br>**BU CU DK UP**

LLFS Participant Contact Information

Interviewer Note: ***This form is to be kept in a confidential file, separate from data entry forms.***

1. What is your name? ____

   PREFIX FIRST NAME MI LAST NAME

2. What is your home address? (Street, City, State, Zip) ______________________________

____

____

3a. What is your home telephone number? ______________________________

3b. What is an alternate telephone number? ______________________________

4a. **US:** What is your Social Security Number? (*Check this box if refused to provide* ☐)

SSN: ___ ___ ___ -- ___ ___ -- ___ ___ ___ ___

4b. **DK:** What is your CPR (Civil Public Registry) Number? (*Check this box if refused to provide* ☐)

CPR: ___ ___ ___ ___ ___ ___ -- ___ ___ ___ ___

5. **US:** What is your Medicare Number? (*Check this box if refused to provide* ☐)

Medicare ID: ___ ___ ___ ___ ___ -- ___ ___ ___ ___ ___ ___ ___

6a. Please provide the name of the person who you would want us to ask to provide information and answer questions for you in the event that you are unable to answer for yourself.

Name: ____

PREFIX FIRST NAME MI LAST NAME

6b. Is this person a family member enrolled in LLFS?

☐1 ....Yes **Go to 7a**
☐0 ....No

6c. Address (Street, City, State, Zip) ____

____

____

Phone: _______________________________ (☐ Home ☐Work) Best day/time to call: ______________

E-Mail Address: _____________________________

6d. Relationship to You (i.e. spue, friend, etc.): ____

7a. Do you have a primary care physician or a specific location that you ***usually*** go to for health care or for advice about your health care?

- ☐ 1 ....Yes
- ☐ 0 ....No

***<u>Interviewer Note</u>*: Please read response options for 7b and check only one.**

7b. Where do you ***usually*** go for health care or advice about health care?

- ☐ 1 ....Private Doctor's Office (individual or group practice)
- ☐ 2 ....Public Clinic, such as a neighborhood health center
- ☐ 3 ....Health Maintenance Organization (HMO)
- ☐ 4 ....Hospital Outpatient Clinic
- ☐ 5 ....Emergency Room
- ☐ 6 ....Other (Please Specify)_________________________
- ☐ D ....Don't Know
- ☐ R ....Refused

7c. Please tell me the name, address and telephone number of the doctor or health care provider that you usually visit for health care needs.

Organization Name: ____

Physician Name: ____

PREFIX **FIRST NAME** **MI** **LAST NAME**

Address (Street, City, State, Zip): ____

____

____

Office Phone: _______________________________ Office Fax: ___________________________

E-Mail Address: ___________________________________

LLFS FORMS & TESTING CHECKLIST

(For Internal Use Only)

Key: IP=In-Person; T=Telephone; SA=Self-Administered

	If Completed:					If NOT Completed:	
**Screening and Visit Test & Procedure Instruments**	**How was Instrument Administered?**			**Date Started Date Completed**	**Time Started Time Completed**	**Place an "X" below**	**Please specify why this has not been completed**
	**IP**	**T**	**SA**				
**TS1**: Proband Telephone Screener				Date: _________<br>Date: _________	Time: ______ A/P<br>Time: ______ A/P		
**TS1a**: Family Member Telephone Screener				Date: _________<br>Date: _________	Time: ______ A/P<br>Time: ______ A/P		
**TS2**: Relative Contact Information Form				Date: _________<br>Date: _________	Time: ______ A/P<br>Time: ______ A/P		
**TS1a-c**: Telephone Screener Control Subjects				Date: _________<br>Date: _________	Time: ______ A/P<br>Time: ______ A/P		
**TS1c**: FLoSS Instrument Screener Control Subjects				Date: _________<br>Date: _________	Time: ______ A/P<br>Time: ______ A/P		
**TS3**: Pedigree Information Form				Date: _________<br>Date: _________	Time: ______ A/P<br>Time: ______ A/P		
**1.** Informed Consent & Interview Feasibility Form *(Panel 1)*				Date: _________<br>Date: _________	Time: ______ A/P<br>Time: ______ A/P		
**2.** Blood Collection/ Venipuncture Form *(Panel 13)*				Date: _________<br>Date: _________	Time: ______ A/P<br>Time: ______ A/P		
**3.** Performance Measures *(Panel 8)*				Date: _________<br>Date: _________	Time: ______ A/P<br>Time: ______ A/P		
**4.** Spirometry *(Panel 10)*				Date: _________<br>Date: _________	Time: ______ A/P<br>Time: ______ A/P		

	If Completed:					If NOT Completed:	
**Screening and Visit Test & Procedure Instruments**	**How was Instrument Administered?**			**Date Started Date Completed**	**Time Started Time Completed**	**Place an "X" below**	**Please specify why this has not been completed**
	**IP**	**T**	**SA**				
**5.** BP, Height, Weight, Knee Height, Waist Circumference *(Panel 9)*				Date: _________<br>Date: _________	Time: ______ A/P<br>Time: ______ A/P		
**6.** Medical History Form *(Panel 5)*				Date: _________<br>Date: _________	Time: ______ A/P<br>Time: ______ A/P		
**7.** Medication Inventory Form *(Panel 6)*				Date: _________<br>Date: _________	Time: ______ A/P<br>Time: ______ A/P		
**8.** Sociodemographic Information Form *(Panel 2: US or DK Version)*				Date: _________<br>Date: _________	Time: ______ A/P<br>Time: ______ A/P		
**9.** MMSE *(Panel 7)*				Date: _________<br>Date: _________	Time: ______ A/P<br>Time: ______ A/P		
**10.** Logical Memory 1A-Immediate *(Panel 7)*				Date: _________<br>Date: _________	Time: ______ A/P<br>Time: ______ A/P		
**11.** Digit Span Forward/Backward *(Panel 7)*				Date: _________<br>Date: _________	Time: ______ A/P<br>Time: ______ A/P		
**12.** Category Fluency Test *(Panel 7)*				Date: _________<br>Date: _________	Time: ______ A/P<br>Time: ______ A/P		
**13.** Digit Symbol Test *(Panel 7)*				Date: _________<br>Date: _________	Time: ______ A/P<br>Time: ______ A/P		
**14.** Logical Memory IIA-Delayed *(Panel 7)*				Date: _________<br>Date: _________	Time: ______ A/P<br>Time: ______ A/P		
**15.** Telephone Interview for Cognitive Status (TICS) *(Panel 7b)*							

	If Completed:					If NOT Completed:	
**Screening and Visit Test & Procedure Instruments**	**How was Instrument Administered?**			**Date Started Date Completed**	**Time Started Time Completed**	**Place an "X" below**	**Please specify why this has not been completed**
	**IP**	**T**	**SA**				
**16.** CES-D and NEO *(Panel 11)*				Date: _________<br>Date: _________	Time: ______ A/P<br>Time: ______ A/P		
**17.** Personal History Form *(Panel 4)*				Date: _________<br>Date: _________	Time: ______ A/P<br>Time: ______ A/P		
**18.** Physical Function and Activity Form *(Panel 3)*				Date: _________<br>Date: _________	Time: ______ A/P<br>Time: ______ A/P		
**19.** Family Structure Review Form *(Panel 12)*				Date: _________<br>Date: _________	Time: ______ A/P<br>Time: ______ A/P		

LONG LIFE FAMILY STUDY

(Affix Label Here)

Participant ID: ____________________

Participant Name Code: ____________

Date Form Filled Out: ☐☐☐☐☐☐☐☐☐
d d M M M y y y y
(e.g., 10JUN2005)

Interviewer Code: ☐☐☐

Circle Field Center Location:

BU CU DK UP

Annual Follow-Up Telephone Contact Questionnaire

***For Internal Use Only* – Please Mark the Appropriate Box Below:**

☐1 ....This Form was Administered via a DFR/Proxy
☐2 ....This Form was Administered via Telephone by Study Personnel
☐3 ....This Form was Mailed and Self-Administered by Participant

***Interviewer*: Please indicate which Follow-Up Contact this is:**

☐1 ....First Year Contact
☐2 ....Second Year Contact
☐3 ....Third Year Contact

1a. Is the participant deceased?

☐1 ....Yes **Go to Q1b**
☐0 ....No **Go to Q2**

1b. Date of Death: ____ ____ / ____ ____ ____ / ____ ____ ____ ____ **End Questionnaire**

2. In general, how would you say your health is?

☐5 ....Excellent
☐4 ....Very Good
☐3 ....Good
☐2 ....Fair
☐1 ....Poor
☐D ....Don't Know
☐R ....Refused

3a. Have you been hospitalized in the past year?

☐1 ....Yes **Go to Q3b**
☐0 ....No **Go to Q4a**

3b. How many times have you been hospitalized? ________________________

3c. For each hospitalization indicated in **Q3b**, please provide the following:

(1) Date of Hospitalization: ____ ____ / ____ ____ ____ / ____ ____ ____ ____

Reason for Hospitalization: ____

Study Personnel Only: Code: _______

(2) Date of Hospitalization: ____ ____ / ____ ____ ____ / ____ ____ ____ ____

Reason for Hospitalization: ____

Study Personnel Only: Code: ________

(3) Date of Hospitalization: ____ ____ / ____ ____ ____ / ____ ____ ____ ____

Reason for Hospitalization: ____

Study Personnel Only: Code: _______

For more than three (3) hospitalizations, please list on a separate sheet.

4a. Do you have any difficulty getting in and out of bed or chairs without help from another person or special equipment?

- ☐1 ....Yes
- ☐0 ....No **Go to Q5a**
- ☐D ....Don't Know **Go to Q5a**
- ☐R ....Refused **Go to Q5a**

4b. How much difficulty would you say you have? Would you say . . .

- ☐1 ....A little difficulty
- ☐2 ....Some difficulty
- ☐3 ....A lot of difficulty
- ☐0 ....I am unable to do it
- ☐D ....Don't Know

4c. Do you usually receive help from another person getting in and out of bed or chairs?

- ☐1 ....Yes
- ☐0 ....No

5a. Do you have any difficulty bathing or showering without help from another person or special equipment?

- ☐1 ....Yes
- ☐0 ....No **Go to Q6a**
- ☐D ....Don't Know **Go to Q6a**
- ☐R ....Refused **Go to Q6a**

5b. How much difficulty would you say you have? Would you say . . .

- ☐[1] ....A little difficulty
- ☐[2] ....Some difficulty
- ☐[3] ....A lot of difficulty
- ☐[0] ....I am unable to do it
- ☐[D] ....Don't Know

5c. Do you usually receive help from another person bathing or showering?

- ☐[1] ....Yes
- ☐[0] ....No

6a. Do you have any difficulty walking across a small room without help from another person or special equipment?

- ☐[1] ....Yes
- ☐[0] ....No **Go to Q7a**
- ☐[D] ....Don't Know **Go to Q7a**
- ☐[R] ....Refused **Go to Q7a**

6b. How much difficulty would you say you have? Would you say . . .

- ☐[1] ....A little difficulty
- ☐[2] ....Some difficulty
- ☐[3] ....A lot of difficulty
- ☐[0] ....I am unable to do it
- ☐[D] ....Don't Know

6c. Do you usually receive help from another person walking across a small room?

- ☐[1] ....Yes
- ☐[0] ....No

7a. Because of a health or physical problem, do you have any difficulty walking a quarter of a mile (2-3 blocks)?

- ☐[1] ....Yes
- ☐[0] ....No **Go to Q7d**

7b. How much difficulty would you say you have? Would you say . . .

- ☐[1] ....A little difficulty
- ☐[2] ....Some difficulty
- ☐[3] ....A lot of difficulty
- ☐[0] ....I am unable to do it on my own
- ☐[D] ....Don't Know

7c. Do you usually receive help from another person to walk a quarter of a mile (2-3 blocks)?

- ☐[1] ....Yes **Go to Q8a**
- ☐[0] ....No **Go to Q8a**
- ☐[D] ....Doesn't Do **Go to Q9a**

7d. How easy is it for you to walk for a quarter of a mile (2-3 blocks)? Would you say . . .

- ☐[1] ....Very easy
- ☐[2] ....Somewhat easy
- ☐[3] ....Not that easy
- ☐[D] ....Don't Know

8a. Because of a health or physical problem, do you have any difficulty walking a distance of one mile, that is about 8 to 12 blocks.

- ☐[1] ....Yes **Go to Q9a**
- ☐[0] ....No **Go to Q8b**

8b. How easy is it for you to walk one mile (about 8 to 12 blocks)? Would you say . . .

- ☐[1] ....Very easy
- ☐[2] ....Somewhat easy
- ☐[3] ....Not that easy
- ☐[D] ....Don't Know

9a. Because of a health or physical problem, do you have any difficulty walking up one flight of stairs (about 10 steps) without resting?

- ☐[1] ....Yes
- ☐[0] ....No **Go to Q9d**

9b. If yes, how much difficulty would you say you have? Would you say . . .

- ☐[1] ....A little difficulty
- ☐[2] ....Some difficulty
- ☐[3] ....A lot of difficulty
- ☐[0] ....I am unable to do it on my own
- ☐[D] ....Don't Know

9c. Do you usually receive help from another person to walk up one flight of stairs (about 10 steps)?

☐[1] ....Yes **Go to Q10a**
☐[0] ....No **Go to Q10a**
☐[D] ....Doesn't Do **Go to Q11a**

9d. How easy is it for you to walk up one flight of stairs (about 10 steps)? Would you say . . .

☐[1] ....Very easy
☐[2] ....Somewhat easy
☐[3] ....Not that easy
☐[D] ....Don't Know

10a. Because of a health or physical problem, do you have any difficulty walking up two flights of stairs (about 20 steps) without resting?

☐[1] ....Yes **Go to Q11a**
☐[0] ....No **Go to Q10b**

10b. How easy is it for you to walk up two flights of stairs (about 20 steps)? Would you say . . .

☐[1] ....Very easy
☐[2] ....Somewhat easy
☐[3] ....Not that easy
☐[D] ....Don't Know

11a. Please update (i.e., confirm/change) your address, phone number and E-Mail address ***(<u>Interviewer</u>: Please pre-populate)***:

Home Address: ____

____

____________________________________ ____________________ ____ ____ ____ ____ ____

City State Zip Code

Home Telephone Number: (____ ____ ____) ____ ____ ____ **-** ____ ____ ____ ____

E-Mail Address: ____

☐[1] ....Confirmed, this information is accurate
☐[2] ....Changed, this information is no longer accurate
☐[3] ....This information is accurate, but I am planning to move

11b. If this information has changed, please provide your new contact information:

Home Address: ____

____

_____________________________________ ____________________ ____ ____ ____ ____ ____

City State Zip Code

Home Telephone Number: (____ ____ ____) ____ ____ ____ **-** ____ ____ ____ ____

E-Mail Address: ____

Interviewer Script: *If you are planning to move, please call us at [Field Center Toll-Free Number] to update when you have this information.*

12a. Please update (i.e., confirm/change) the "Contact Person" information you provided to us at the time of enrollment ***(Interviewer: Please pre-populate)***:

Name: ____

PREFIX **FIRST NAME** **MI** **LAST NAME**

12b. Is this person a family member enrolled in LLFS?

☐ 1 ....Yes
☐ 0 ....No

12c. **Home Address (Street, City, State, Zip)**: ____

____

____

Phone: ________________________________ ☐ **Home** ☐ **Work**

Best day/time to call: ____

E-Mail Address: _______________________

12d. Relationship to You:

- ☐[1] ....Brother
- ☐[2] ....Sister
- ☐[3] ....Half Brother
- ☐[4] ....Half Sister
- ☐[5] ....Father
- ☐[6] ....Mother
- ☐[7] ....Stepfather
- ☐[8] ....Stepmother
- ☐[9] ....Husband
- ☐[10] ....Wife
- ☐[11] ....Son
- ☐[12] ....Daughter
- ☐[13] ....Stepson
- ☐[14] ....Stepdaughter
- ☐[15] ....Stepbrother
- ☐[16] ....Stepsister
- ☐[17] ....Uncle
- ☐[18] ....Aunt
- ☐[19] ....Nephew
- ☐[20] ....Niece
- ☐[21] ....Cousin
- ☐[22] ....In-Law
- ☐[23] ....Other (please specify): ____

***<u>Interviewer Note:</u>* The comments are not entered into the DES.**

13. Comments: ____

____

____

____

____

____

14a. Who is completing this form?

☐[1]	.................Study Participant	**Go to Q15**
☐[2]	.................Contact Person; Name: ______________________	**Go to Q14b**
☐[3]	.................Other; Name: ___________________________________	**Go to Q14b**

14b. What is your relationship to the Study Participant?

- ☐[1] ....Spouse
- ☐[2] ....Child (Daughter/Son)
- ☐[3] ....Sibling (Brother/Sister)
- ☐[4] ....Niece/Nephew
- ☐[5] ....Other: ____

14c. Please provide the reason that you are completing this form on behalf of or instead of the Study Participant? ***(Please X Only One)***

- ☐[1] ....Physically Ill
- ☐[2] ....Dementia
- ☐[3] ....Hearing Impairment
- ☐[4] ....Too Busy / Unavailable
- ☐[5] ....Nursing Home or Long-Term Care
- ☐[6] ....Unable to be Reached or Located
- ☐[7] ....Other: ____

15. At this time, is there any additional family member (brother/sister, aunt/uncle, niece/nephew, cousin) that you have spoken with that is now interested in participating in the LONG LIFE Family Study?

- ☐[1] ....Yes
- ☐[0] ....No

Interviewer Note: If "Yes", ask for Name and Contact Information and add to TS2. ***Note:*** If the answer to **Q15** is "Yes", and this questionnaire is being conducted via mail, a member of the LLFS Research Staff will be contacting you to obtain the contact information for the interested family members.

End Interview Script: *"Thank you very much for answering these questions. I enjoyed talking with you. Please remember to call us if you move or if your mailing address changes. I look forward to speaking with you again at approximately the same time next year. Again, thank you for your ongoing interest in our study."*
